# An underwater imagery identification guide for shallow, mesophotic and deep-sea benthos in Maldives

**DOI:** 10.3897/BDJ.12.e120128

**Published:** 2024-06-18

**Authors:** Farah Amjad, Mohamed Ahusan, Hana Amir, Nina M de Villiers, Erika Gress, Christopher L Mah, Shafiya Naeem, Nuria Rico-Seijo, Toufiek Samaai, Maryiam S Afzal, Lucy C Woodall, Paris V Stefanoudis

**Affiliations:** 1 Nekton Foundation, Oxford, United Kingdom Nekton Foundation Oxford United Kingdom; 2 Maldives Marine Research Institute, Male, Maldives Maldives Marine Research Institute Male Maldives; 3 Bangor University, Wales, United Kingdom Bangor University Wales United Kingdom; 4 James Cook University, Townsville, Australia James Cook University Townsville Australia; 5 Smithsonian Institution, Washington, United States of America Smithsonian Institution Washington United States of America; 6 Department of Forestry, Fisheries and Environment, Cape Town, South Africa Department of Forestry, Fisheries and Environment Cape Town South Africa; 7 University of the Ryukyus, Okinawa, Japan University of the Ryukyus Okinawa Japan; 8 University of Exeter, Exeter, United Kingdom University of Exeter Exeter United Kingdom

**Keywords:** coral reefs, mesophotic coral ecosystems, deep-sea, benthos, morphotype, Maldives, Indian Ocean

## Abstract

**Background:**

During the 2022 Nekton Maldives Mission, we deployed a variety of platforms (snorkelling, remotely-operated vehicles and manned submersibles) to conduct video surveys of the biodiversity and composition of shallow (< 30 m), mesophotic (30-150 m) and deep-sea (> 150 m) benthos found in the Maldives’ central and southern atolls. In total, ~ 80 hrs of stereo-video footage were collected during the benthic transect surveys, which were subsequently processed using annotation software in order to evaluate benthic biodiversity and community composition. Here, we present a photographic guide for the visual, *in situ* identification of reef benthos encountered, including corals, sponges and other invertebrates that inhabit Maldives’ nearshore habitats. We hope that this identification guide will aid future imagery-based surveys or observations of organisms during fieldwork.

**New information:**

A total of 283 morphotypes were identified, including those belonging to Octocorallia (61), Scleractinia (57), Porifera (38), Asteroidea (22), Antipatharia (15), Decapoda (13), Hydrozoa (12), Holothuroidea (10), Actiniaria (9), Echinoidea (8), Annelida (6), Chlorophyta (5), Gastropoda (4), Bivalvia (4), Ascidiacea (3), Crinoidea (3), Bryozoa (2), Cyanobacteria (2), Zoantharia (2), Cephalopoda (1), Ceriantharia (1), Corallimorpharia (1), Ctenophora (1), Ophiuroidea (1), Rhodophyta (1) and to an unknown category (1). Out of these, we identified 40 to species level, 120 to genus, 47 to family, 14 to order and suborder, 58 to class and subclass, two to phylum and one was of unknown phylum. This represents the first attempt to catalogue the mesophotic and deep-sea benthic megafaunal diversity in the Maldives using underwater imagery.

## Introduction

Coral reefs in the Maldives are of global significance due to their rich biodiversity (Spalding et al. 2001) and commercially important fisheries species that they support including groupers (Sattar et al. 2011), sea cucumbers (MEE 2017) and a variety of aquarium fish (Saleem and Islam 2008). Previous marine research and monitoring efforts in the Maldives have primarily focused on waters accessible by SCUBA (i.e. < 30 m) (MEE 2017), thus there is little information on deeper communities, including mesophotic coral ecosystems (30-150 m) and deep-sea (> 150 m) habitats.

These deeper environments provide a suite of benefits to humans, including coastal protection, fisheries and nursery grounds (Holstein et al. 2019). In addition, they have unique biodiversity and ecosystem functioning (Stefanoudis et al. 2023) and, hence, warrant protection in their own right. Despite being less accessible to most humans, these ecosystems are nevertheless affected by the consequences of anthropogenic activities, including plastic pollution (Pinheiro et al. 2023), thermal stress (Diaz et al. 2023a) and fishing (Soares et al. 2018).

Expanding knowledge of habitats below 30 m in the Maldives is crucial for making informed decisions about sustainable management. The Nekton Maldives Mission directly addressed that knowledge gap by investigating shallow (< 30 m), mesophotic coral ecosystems (30-150 m) and deeper habitats (> 150 m) in the Maldives, documenting biological communities, diversity and associated environmental conditions. From this exploratory work, this Field Identification Guide provides documentation and descriptions of a variety of benthic taxa that occur beyond the depths accessible to SCUBA divers.

## Materials and methods

**Figure 1.**
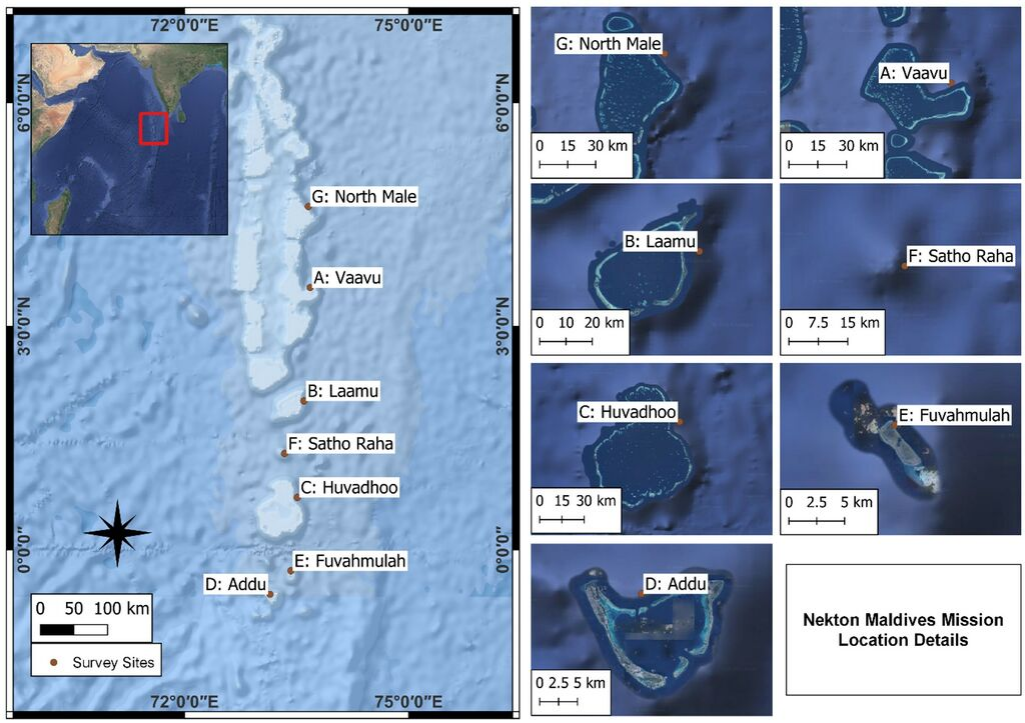
Survey sites investigated during the 2022 Nekton Maldives Mission (taken from Ahusan et al. 2023).

The Nekton Maldives Mission took place between 4 September and 6 October 2022 onboard the *RV Odyssey*. In total, six coral atolls and a seamount across the central and southern Maldives Archipelago were investigated (Fig. 1) using a combination of video surveys, biological sample collection, water chemistry measurements and bathymetry (Ahusan et al. 2023). Due to poor weather conditions, only a subset of surveys were made in one site (Fuvahmulah) and solely multibeam data and environmental DNA data in another site (seamount *Satho Raha*).

The video-based transect surveys, which are the focus of this contribution, were conducted by snorkellers (2 m), remotely-operated vehicles (ROVs) (~ 10 and 30 m) and manned submersibles (~ 60, 120, 250 and 500 m) using stereo-video camera systems to record benthic biodiversity. Paralenz Dive and Paralenz Vaquita cameras were used for surveys between 2 and 30 m and Teledyne Marine’s L3C-HD for deeper surveys, all recording at a minimum resolution of 1920 × 1080 pixels and a frame rate of 60 fps. Two to three replicate video transect surveys were conducted at each depth contour at each site (except for Fuvahmulah), each 250 m long. During all surveys, a constant altitude of 1-2 m above the seabed was aimed to allow for sufficient overlap between the stereo cameras yet staying close enough to the bottom to maximise the taxonomic resolution of the organisms observed.

Samples of the most common benthic organisms, typically octocorals, black corals or sponges, were collected to verify taxonomic identifications of video-based biodiversity surveys. Each sample was given a unique ID number and subsamples preserved in 98% ethanol were stored in fridges and subsamples in RNAlater stored at room temperature and later in the fridges.

All collected video footage was screened during and after the expedition to create image-based taxon lists. Together with the collected specimens, these were then reviewed during a taxonomic workshop that took place in the Maldives in February 2023 to verify identifications with taxonomic experts (Ahusan et al. 2023). Subsequent imagery annotation to derive biodiversity and other community metrics will comprise a separate publication (in prep).

## Data resources

### Authors' note

This guide has been designed to aid in the identification of benthic organisms as they are seen *in situ*. As such, it will be of great assistance to marine professionals observing these organisms in their natural habitat during fieldwork and to researchers annotating imagery-based datasets. Similar efforts for the Indian Ocean include field identification guides for Seychelles (Fassbender et al. 2021), Chagos (Diaz et al. 2023b) and Comoros (in prep.).

Given that some morphological features required for positive taxonomic identification are not visible through imagery alone and often require further *ex-situ* examination, assigning observed organisms to species level is not always possible. In fact, it is common practice to place observed organisms into ‘morphotypes’ or ‘morphospecies’ that are morphologically similar organisms that could represent a species, genus or higher-level classification (Howell et al. 2019, Fassbender et al. 2021).

Based on this work, we provide the lowest possible taxonomic identification from imagery alone and information on distribution, depth range and size (i.e. length across longest dimension) for each morphotype entry. Size represents the average length from all measured colonies/individuals during image annotation of collected data (Stefanoudis et al., in prep.). Note that size was not possible to estimate for some morphotypes that are difficult to count, such as some encrusting and stoloniferous forms (e.g. algae, some sponges). We also provide a short morphological description as observed from the video footage and some representative *in situ* images. Additional *ex situ* images of collected specimens are also provided where available, whose names comprise "MAL_" and a numerical number following the naming conventions during the Nekton Maldives Mission. These are typically identified to a lower taxonomic level (e.g. species or genus) than the morphotype entry they are assigned since they were examined in the laboratory post-expedition. Note that they are not assigned to a different entry since, from underwater footage alone, they are unlikely to have been identified to this fine taxonomic level.

To allow for inter-comparability of Indian Ocean benthic communities between locations, we use the same morphotype names for shared morphotypes found in the Seychelles (Fassbender et al. 2021) and Maldives.

### How to use the Guide

All morphotypes that have been observed are classified into 18 main classification groups, with categories ranging from phylum to order. The selection of the taxonomic level for each main group is in accordance with the classification widely used in the field by experts and the general public (e.g. Phylum Porifera for sponges or Order Antipatharia for black corals etc.). Individuals of each major group are categorised into the lowest taxonomic level possible and allocated morphotypes. Table 1 summarises all 281 morphotypes identified.

## Taxon treatments

### Cyanobacteria stet. sp. 1

F9632B1D-F693-575B-945C-DD1F6C524E68

#### Materials

- **Type status:** Other material. **Taxon:** scientificName: Cyanobacteria sp. 1; kingdom: Bacteria; phylum: Cyanobacteria; **Location:** waterBody: Indian Ocean; country: Maldives; locality: North Male’, Laamu, Huvadhu; minimumDepthInMeters: 2; maximumDepthInMeters: 30; locationRemarks: Nekton Maldives Mission; **Identification:** identifiedBy: Farah Amjad, Paris Stefanoudis; dateIdentified: 2022, 2023; identificationRemarks: Identified only from imagery; **Event:** samplingProtocol: Submersible OR Remotely Operated Vehicle OR Snorkel; **Record Level:** basisOfRecord: Human observation

### Cyanobacteria stet. sp. 2

D61CFF8D-4C63-58DA-BE07-9918787F3AE8

#### Materials

- **Type status:** Other material. **Taxon:** scientificName: Cyanobacteria sp. 2; kingdom: Bacteria; phylum: Cyanobacteria; **Location:** waterBody: Indian Ocean; country: Maldives; locality: North Male’, Huvadhu; minimumDepthInMeters: 30; maximumDepthInMeters: 30; locationRemarks: Nekton Maldives Mission; **Identification:** identifiedBy: Farah Amjad, Paris Stefanoudis; dateIdentified: 2022, 2023; identificationRemarks: Identified only from imagery; **Event:** samplingProtocol: Submersible OR Remotely Operated Vehicle OR Snorkel; **Record Level:** basisOfRecord: Human observation

### Caulerpa sp. indet. 1

669DBD9F-C3F1-505D-AA9E-16E14ADE6C6F

#### Materials

- **Type status:** Other material. **Taxon:** scientificName: *Caulerpa* sp. 1; kingdom: Plantae; phylum: Chlorophyta; class: Ulvophyceae; order: Bryopsidales; family: Caulerpaceae; genus: Caulerpa; **Location:** waterBody: Indian Ocean; country: Maldives; locality: North Male’; minimumDepthInMeters: 10; maximumDepthInMeters: 30; locationRemarks: Nekton Maldives Mission; **Identification:** identifiedBy: Farah Amjad, Paris Stefanoudis; dateIdentified: 2022, 2023; identificationRemarks: Identified only from imagery; **Event:** samplingProtocol: Submersible OR Remotely Operated Vehicle OR Snorkel; **Record Level:** basisOfRecord: Human observation

### Caulerpa serrulata

(Forsskål) J.Agardh, 1837

B4847A11-58C2-5DFE-B8F0-150DE040434C

#### Materials

- **Type status:** Other material. **Taxon:** scientificName: *Caulerpaserrulata*; kingdom: Plantae; phylum: Chlorophyta; class: Ulvophyceae; order: Bryopsidales; family: Caulerpaceae; genus: Caulerpa; scientificNameAuthorship: (Forsskål) J.Agardh, 1837; **Location:** waterBody: Indian Ocean; country: Maldives; locality: North Male’, Vaavu, Laamu, Huvadhu, Addu; minimumDepthInMeters: 2; maximumDepthInMeters: 30; locationRemarks: Nekton Maldives Mission; **Identification:** identifiedBy: Farah Amjad, Paris Stefanoudis; dateIdentified: 2022, 2023; identificationRemarks: Identified only from imagery; **Event:** samplingProtocol: Submersible OR Remotely Operated Vehicle OR Snorkel; **Record Level:** basisOfRecord: Human observation

### Halimeda sp. indet.

91F60809-DAF5-5CF7-9EB9-D49A75C77C94

#### Materials

- **Type status:** Other material. **Taxon:** scientificName: *Halimeda* sp.; kingdom: Plantae; phylum: Chlorophyta; class: Ulvophyceae; order: Bryopsidales; family: Halimedaceae; genus: Halimeda; **Location:** waterBody: Indian Ocean; country: Maldives; locality: TBD; minimumDepthInMeters: TBD; maximumDepthInMeters: TBD; locationRemarks: Nekton Maldives Mission; **Identification:** identifiedBy: Farah Amjad, Paris Stefanoudis; dateIdentified: 2022, 2023; identificationRemarks: Identified only from imagery; **Event:** samplingProtocol: Submersible OR Remotely Operated Vehicle OR Snorkel; **Record Level:** basisOfRecord: Human observation

### Halimeda micronesica

Yamada, 1941

BA2BD68B-965F-5263-987A-C6A8668A4348

#### Materials

- **Type status:** Other material. **Taxon:** scientificName: *Halimedamicronesica*; kingdom: Plantae; phylum: Chlorophyta; class: Ulvophyceae; order: Bryopsidales; family: Halimedaceae; genus: Halimeda; scientificNameAuthorship: Yamada, 1941; **Location:** waterBody: Indian Ocean; country: Maldives; locality: North Male’, Vaavu, Huvadhu, Laamu, Addu; minimumDepthInMeters: 10; maximumDepthInMeters: 30; locationRemarks: Nekton Maldives Mission; **Identification:** identifiedBy: Farah Amjad, Paris Stefanoudis; dateIdentified: 2022, 2023; identificationRemarks: Identified only from imagery; **Event:** samplingProtocol: Submersible OR Remotely Operated Vehicle OR Snorkel; **Record Level:** basisOfRecord: Human observation

### Tydemania expeditionis

Weber Bosse, 1901

CAD275E4-1A38-5897-A7A1-CE6B585A896B

#### Materials

- **Type status:** Other material. **Taxon:** scientificName: *Tydemaniaexpeditionis*; kingdom: Plantae; phylum: Chlorophyta; class: Ulvophyceae; order: Bryopsidales; family: Udoteaceae; genus: Tydemania; scientificNameAuthorship: Weber Bosse, 1901; **Location:** waterBody: Indian Ocean; country: Maldives; locality: North Male’, Vaavu, Laamu, Addu, Huvadhu; minimumDepthInMeters: 2; maximumDepthInMeters: 30; locationRemarks: Nekton Maldives Mission; **Identification:** identifiedBy: Farah Amjad, Paris Stefanoudis; dateIdentified: 2022, 2023; identificationRemarks: Identified only from imagery; **Event:** samplingProtocol: Submersible OR Remotely Operated Vehicle OR Snorkel; **Record Level:** basisOfRecord: Human observation

### Corallinales stet.

3F454CA4-B916-5DD0-A376-636B5E9F3456

#### Materials

- **Type status:** Other material. **Taxon:** scientificName: Corallinales; kingdom: Plantae; phylum: Rhodophyta; class: Florideophyceae; order: Corallinales; **Location:** waterBody: Indian Ocean; country: Maldives; locality: North Male’, Vaavu, Laamu, Huvadhu, Addu; minimumDepthInMeters: 2; maximumDepthInMeters: 121; locationRemarks: Nekton Maldives Mission; **Identification:** identifiedBy: Farah Amjad, Paris Stefanoudis; dateIdentified: 2022, 2023; identificationRemarks: Identified only from imagery; **Event:** samplingProtocol: Submersible OR Remotely Operated Vehicle OR Snorkel; **Record Level:** basisOfRecord: Human observation

### Spheciospongia sp. indet. 4

D8889C64-DBD4-56B2-9D9B-010F4E154589

#### Materials

- **Type status:** Other material. **Taxon:** scientificName: *Spheciospongia* sp. 4; kingdom: Animalia; phylum: Porifera; class: Demospongiae; order: Clionaida; family: Clionaidae; genus: Spheciospongia; **Location:** waterBody: Indian Ocean; country: Maldives; locality: Laamu, Huvadhu, Addu; minimumDepthInMeters: 30; maximumDepthInMeters: 62; locationRemarks: Nekton Maldives Mission; **Identification:** identifiedBy: Farah Amjad, Paris Stefanoudis, Toufiek Samaai; dateIdentified: 2022, 2023; identificationRemarks: Identified only from imagery; **Event:** samplingProtocol: Submersible OR Remotely Operated Vehicle OR Snorkel; **Record Level:** basisOfRecord: Human observation

### Spheciospongia sp. indet. 5

7BECD251-52FB-5B89-9586-8387FFFA85BF

#### Materials

- **Type status:** Other material. **Taxon:** scientificName: *Spheciospongia* sp. 5; kingdom: Animalia; phylum: Porifera; class: Demospongiae; order: Clionaida; family: Clionaidae; genus: Spheciospongia; **Location:** waterBody: Indian Ocean; country: Maldives; locality: North Male’, Vaavu, Laamu, Huvadhu, Addu; minimumDepthInMeters: 52; maximumDepthInMeters: 124; locationRemarks: Nekton Maldives Mission; **Identification:** identifiedBy: Farah Amjad, Paris Stefanoudis, Toufiek Samaai; dateIdentified: 2022, 2023; identificationRemarks: Identified only from imagery; **Event:** samplingProtocol: Submersible OR Remotely Operated Vehicle OR Snorkel; **Record Level:** basisOfRecord: Human observation

### Spheciospongia excentrica

(Burton, 1931)

3D5601AE-4158-59E8-BE36-590AD04933D4

#### Materials

- **Type status:** Other material. **Taxon:** scientificName: *Spheciospongiaexcentrica*; kingdom: Animalia; phylum: Porifera; class: Demospongiae; order: Clionaida; family: Clionaidae; genus: Spheciospongia; scientificNameAuthorship: (Burton, 1931); **Location:** waterBody: Indian Ocean; country: Maldives; locality: Addu; minimumDepthInMeters: 30; maximumDepthInMeters: 30; locationRemarks: Nekton Maldives Mission; **Identification:** identifiedBy: Farah Amjad, Paris Stefanoudis, Toufiek Samaai; dateIdentified: 2022, 2023; identificationRemarks: Identified only from imagery; **Event:** samplingProtocol: Submersible OR Remotely Operated Vehicle OR Snorkel; **Record Level:** basisOfRecord: Human observation

#### Notes

Large cup- to barrel-shaped sponges. Surface smooth, but undulating and somewhat slippery to the touch. Large sunken indentations visible on the outer surface. Oscules randomly scattered on the inner side of the vase. Approximately 46 cm across. Colour in life brown; in preservative beige (Fig. 12).

### Phyllospongia foliascens

(Pallas, 1766)

56DB0A91-60D6-5861-A731-E914F656C6E8

#### Materials

- **Type status:** Other material. **Taxon:** scientificName: *Phyllospongiafoliascens*; kingdom: Animalia; phylum: Porifera; class: Demospongiae; order: Dictyoceratida; family: Thorectidae; genus: Phyllospongia; scientificNameAuthorship: (Pallas, 1766); **Location:** waterBody: Indian Ocean; country: Maldives; locality: North Male’, Vaavu, Laamu, Huvadhu, Fuvahmulah, Addu; minimumDepthInMeters: 59; maximumDepthInMeters: 120; locationRemarks: Nekton Maldives Mission; **Identification:** identifiedBy: Farah Amjad, Paris Stefanoudis, Toufiek Samaai; dateIdentified: 2022, 2023; identificationRemarks: Identified only from imagery; **Event:** samplingProtocol: Submersible OR Remotely Operated Vehicle OR Snorkel; **Record Level:** basisOfRecord: Human observation

#### Notes

Oliaceous sponges attached to substratum by stalk. Usually made up of two concentric plates. The sponge is rubbery with ostia evenly scattered on the rim of the sponge. Surface smooth. Approximately 17 cm in longest dimension. Colouration light brown to pale grey with lighter shades of yellow (Fig. 13).

### Callyspongia sp. indet. 1

7C31D44D-76EB-5625-A3F2-C610B74DF3D7

#### Materials

- **Type status:** Other material. **Taxon:** scientificName: *Callyspongia* sp. 1; kingdom: Animalia; phylum: Porifera; class: Demospongiae; order: Haplosclerida; family: Callyspongiidae; genus: Callyspongia; **Location:** waterBody: Indian Ocean; country: Maldives; locality: North Male’, Vaavu; minimumDepthInMeters: 30; maximumDepthInMeters: 30; locationRemarks: Nekton Maldives Mission; **Identification:** identifiedBy: Farah Amjad, Paris Stefanoudis, Toufiek Samaai; dateIdentified: 2022, 2023; identificationRemarks: Identified only from imagery; **Event:** samplingProtocol: Submersible OR Remotely Operated Vehicle OR Snorkel; **Record Level:** basisOfRecord: Human observation

### Petrosiidae gen. indet. sp. 3

C89F4A54-1D40-5A88-BA09-1FF474477DA2

#### Materials

- **Type status:** Other material. **Taxon:** scientificName: Petrosiidae sp. 3; kingdom: Animalia; phylum: Porifera; class: Demospongiae; order: Haplosclerida; family: Petrosiidae; **Location:** waterBody: Indian Ocean; country: Maldives; locality: Huvadhu; minimumDepthInMeters: 30; maximumDepthInMeters: 30; locationRemarks: Nekton Maldives Mission; **Identification:** identifiedBy: Farah Amjad, Paris Stefanoudis, Toufiek Samaai; dateIdentified: 2022, 2023; identificationRemarks: Identified only from imagery; **Event:** samplingProtocol: Submersible OR Remotely Operated Vehicle OR Snorkel; **Record Level:** basisOfRecord: Human observation

### Petrosia sp. indet. 1

DF366647-CE2F-53E0-9E91-B6D055D79B21

#### Materials

- **Type status:** Other material. **Taxon:** scientificName: *Petrosia* sp. 1; kingdom: Animalia; phylum: Porifera; class: Demospongiae; order: Petrosiidae; family: Petrosiidae; genus: Petrosia; **Location:** waterBody: Indian Ocean; country: Maldives; locality: North Male’, Vaavu, Huvadhu; minimumDepthInMeters: 30; maximumDepthInMeters: 62; locationRemarks: Nekton Maldives Mission; **Identification:** identifiedBy: Farah Amjad, Paris Stefanoudis, Toufiek Samaai; dateIdentified: 2022, 2023; identificationRemarks: Identified only from imagery; **Event:** samplingProtocol: Submersible OR Remotely Operated Vehicle OR Snorkel; **Record Level:** basisOfRecord: Human observation

### Petrosia (Strongylophora) sp. indet. 2

DD452954-D320-5B55-A810-636E2470CE79

#### Materials

- **Type status:** Other material. **Taxon:** scientificName: Petrosia (Strongylophora) sp. 2; kingdom: Animalia; phylum: Porifera; class: Demospongiae; order: Petrosiidae; family: Petrosiidae; genus: Petrosia; **Location:** waterBody: Indian Ocean; country: Maldives; locality: North Male’, Huvadhu, Addu; minimumDepthInMeters: 2; maximumDepthInMeters: 10; locationRemarks: Nekton Maldives Mission; **Identification:** identifiedBy: Farah Amjad, Paris Stefanoudis, Toufiek Samaai; dateIdentified: 2022, 2023; identificationRemarks: Identified only from imagery; **Event:** samplingProtocol: Submersible OR Remotely Operated Vehicle OR Snorkel; **Record Level:** basisOfRecord: Human observation

### Haliclona sp. indet. 15

B0985FD8-617C-542C-A3B9-52176AA2380B

#### Materials

- **Type status:** Other material. **Taxon:** scientificName: *Haliclona* sp. 15; kingdom: Animalia; phylum: Porifera; class: Demospongiae; order: Haplosclerida; family: Chalinidae; genus: Haliclona; **Location:** waterBody: Indian Ocean; country: Maldives; locality: Vaavu, Laamu, Huvadhu; minimumDepthInMeters: 30; maximumDepthInMeters: 62; locationRemarks: Nekton Maldives Mission; **Identification:** identifiedBy: Farah Amjad, Paris Stefanoudis, Toufiek Samaai; dateIdentified: 2022, 2023; identificationRemarks: Identified only from imagery; **Event:** samplingProtocol: Submersible OR Remotely Operated Vehicle OR Snorkel; **Record Level:** basisOfRecord: Human observation

### Haliclona sp. indet. 16

C836BCBE-F61B-5280-8489-6E4263FF0A15

#### Materials

- **Type status:** Other material. **Taxon:** scientificName: *Haliclona* sp. 16; kingdom: Animalia; phylum: Porifera; class: Demospongiae; order: Haplosclerida; family: Chalinidae; genus: Haliclona; **Location:** waterBody: Indian Ocean; country: Maldives; locality: Huvadhu; minimumDepthInMeters: 61; maximumDepthInMeters: 62; locationRemarks: Nekton Maldives Mission; **Identification:** identifiedBy: Farah Amjad, Paris Stefanoudis, Toufiek Samaai; dateIdentified: 2022, 2023; identificationRemarks: Identified only from imagery; **Event:** samplingProtocol: Submersible OR Remotely Operated Vehicle OR Snorkel; **Record Level:** basisOfRecord: Human observation

### Haliclona sp. indet. 17

1DF5BC67-4363-5467-BA62-CD447A8B4913

#### Materials

- **Type status:** Other material. **Taxon:** scientificName: *Haliclona* sp. 17; kingdom: Animalia; phylum: Porifera; class: Demospongiae; order: Haplosclerida; family: Chalinidae; genus: Haliclona; **Location:** waterBody: Indian Ocean; country: Maldives; locality: North Male’, Addu; minimumDepthInMeters: 10; maximumDepthInMeters: 60; locationRemarks: Nekton Maldives Mission; **Identification:** identifiedBy: Farah Amjad, Paris Stefanoudis, Toufiek Samaai; dateIdentified: 2022, 2023; identificationRemarks: Identified only from imagery; **Event:** samplingProtocol: Submersible OR Remotely Operated Vehicle OR Snorkel; **Record Level:** basisOfRecord: Human observation

### Haliclona sp. indet. 18

90159F7F-ACBA-5F11-91BC-3AC2EA3BB10B

#### Materials

- **Type status:** Other material. **Taxon:** scientificName: *Haliclona* sp. 18; kingdom: Animalia; phylum: Porifera; class: Demospongiae; order: Haplosclerida; family: Chalinidae; genus: Haliclona; **Location:** waterBody: Indian Ocean; country: Maldives; locality: North Male’, Laamu, Huvadhu, Addu; minimumDepthInMeters: 10; maximumDepthInMeters: 55; locationRemarks: Nekton Maldives Mission; **Identification:** identifiedBy: Farah Amjad, Paris Stefanoudis, Toufiek Samaai; dateIdentified: 2022, 2023; identificationRemarks: Identified only from imagery; **Event:** samplingProtocol: Submersible OR Remotely Operated Vehicle OR Snorkel; **Record Level:** basisOfRecord: Human observation

### Haliclona sp. indet. 19

C8D61441-7CF6-5DF3-AB46-EFDEC66B1D8F

#### Materials

- **Type status:** Other material. **Taxon:** scientificName: *Haliclona* sp. 19; kingdom: Animalia; phylum: Porifera; class: Demospongiae; order: Haplosclerida; family: Chalinidae; genus: Haliclona; **Location:** waterBody: Indian Ocean; country: Maldives; locality: North Male’; minimumDepthInMeters: 115; maximumDepthInMeters: 124; locationRemarks: Nekton Maldives Mission; **Identification:** identifiedBy: Farah Amjad, Paris Stefanoudis, Toufiek Samaai; dateIdentified: 2022, 2023; identificationRemarks: Identified only from imagery; **Event:** samplingProtocol: Submersible OR Remotely Operated Vehicle OR Snorkel; **Record Level:** basisOfRecord: Human observation

### Suberites sp. indet. 3

8DA2DAF5-0CA8-5BB5-AB71-B008CB24CA5D

#### Materials

- **Type status:** Other material. **Taxon:** scientificName: *Suberites* sp. 3; kingdom: Animalia; phylum: Porifera; class: Demospongiae; order: Suberitida; family: Suberitidae; genus: Suberites; **Location:** waterBody: Indian Ocean; country: Maldives; locality: Laamu, Huvadhu; minimumDepthInMeters: 10; maximumDepthInMeters: 62; locationRemarks: Nekton Maldives Mission; **Identification:** identifiedBy: Farah Amjad, Paris Stefanoudis, Toufiek Samaai; dateIdentified: 2022, 2023; identificationRemarks: Identified only from imagery; **Event:** samplingProtocol: Submersible OR Remotely Operated Vehicle OR Snorkel; **Record Level:** basisOfRecord: Human observation

### Rhizaxinella ramulosa

(Ridley & Dendy, 1886)

AEE53D2B-5B89-54D2-B063-86E9850CF3C8

#### Materials

- **Type status:** Other material. **Taxon:** scientificName: *Rhizaxinellaramulosa*; kingdom: Animalia; phylum: Porifera; class: Demospongiae; order: Suberitida; family: Suberitidae; genus: Rhizaxinella; scientificNameAuthorship: Ridley & Dendy, 1886; **Location:** waterBody: Indian Ocean; country: Maldives; locality: North Male’, Addu; minimumDepthInMeters: 489; maximumDepthInMeters: 489; locationRemarks: Nekton Maldives Mission; **Identification:** identifiedBy: Farah Amjad, Paris Stefanoudis, Toufiek Samaai; dateIdentified: 2022, 2023; identificationRemarks: Identified only from imagery; **Event:** samplingProtocol: Submersible OR Remotely Operated Vehicle OR Snorkel; **Record Level:** basisOfRecord: Human observation

#### Description

Pedunculate sponges supported by a short stalk. Stalk is hard like bark. Surface hispid and undulating. One oscule present on the apex of peduncule. Approximately 6 cm tall. Colouration white to light yellow shades (Fig. 24).

### Plakortis sp. indet. 3

D525F337-B245-56F7-A7FB-3DAE6AD6804F

#### Materials

- **Type status:** Other material. **Taxon:** scientificName: *Plakortis* sp. 3; kingdom: Animalia; phylum: Porifera; class: Homoscleromorpha; order: Homosclerophorida; family: Plakinidae; genus: Plakortis; **Location:** waterBody: Indian Ocean; country: Maldives; locality: North Male’, Vaavu, Addu; minimumDepthInMeters: 30; maximumDepthInMeters: 120; locationRemarks: Nekton Maldives Mission; **Identification:** identifiedBy: Farah Amjad, Paris Stefanoudis, Toufiek Samaai; dateIdentified: 2022, 2023; identificationRemarks: Identified only from imagery; **Event:** samplingProtocol: Submersible OR Remotely Operated Vehicle OR Snorkel; **Record Level:** basisOfRecord: Human observation

### Iotrochota nigra

(Baer, 1906)

8461096F-5C82-5126-BA3D-DBAE248DA782

#### Materials

- **Type status:** Other material. **Taxon:** scientificName: *Iotrochotanigra*; kingdom: Animalia; phylum: Porifera; class: Demospongiae-Heteroscleromorpha; order: Poecilosclerida; family: Iotrochotidae; genus: Iotrochota; scientificNameAuthorship: (Baer, 1906); **Location:** waterBody: Indian Ocean; country: Maldives; locality: North Male’, Vaavu, Laamu, Huvadhu, Addu; minimumDepthInMeters: 10; maximumDepthInMeters: 62; locationRemarks: Nekton Maldives Mission; **Identification:** identifiedBy: Farah Amjad, Paris Stefanoudis, Toufiek Samaai; dateIdentified: 2022, 2023; identificationRemarks: Identified only from imagery; **Event:** samplingProtocol: Submersible OR Remotely Operated Vehicle OR Snorkel; **Record Level:** basisOfRecord: Human observation

#### Description

Thickly-encrusting growth forms with numerous ostia evenly scattered over the sponge surface. Approximately 25 cm across. Colouration in dark grey and black tones (Fig. 26).

### Clathria sp. indet. 1

5DAF54EE-311E-5F6B-A616-3662FE958A52

#### Materials

- **Type status:** Other material. **Taxon:** scientificName: *Clathria* sp. 1; kingdom: Animalia; phylum: Porifera; class: Demospongiae-Heteroscleromorpha; order: Poecilosclerida; family: Microcionidae; genus: Clathria; **Location:** waterBody: Indian Ocean; country: Maldives; locality: North Male’, Laamu, Addu; minimumDepthInMeters: 26; maximumDepthInMeters: 30; locationRemarks: Nekton Maldives Mission; **Identification:** identifiedBy: Farah Amjad, Paris Stefanoudis, Toufiek Samaai; dateIdentified: 2022, 2023; identificationRemarks: Identified only from imagery; **Event:** samplingProtocol: Submersible OR Remotely Operated Vehicle OR Snorkel; **Record Level:** basisOfRecord: Human observation

### Stylissa carteri

(Dendy, 1889)

D2627EF3-12E7-5F40-85FC-5894A77BA136

#### Materials

- **Type status:** Other material. **Taxon:** scientificName: *Stylissacarteri*; kingdom: Animalia; phylum: Porifera; class: Demospongiae-Heteroscleromorpha; order: Scopalinida; family: Scopalinidae; genus: Stylissa; scientificNameAuthorship: (Dendy, 1889); **Location:** waterBody: Indian Ocean; country: Maldives; locality: North Male’, Vaavu, Laamu, Huvadhu, Addu; minimumDepthInMeters: 30; maximumDepthInMeters: 62; locationRemarks: Nekton Maldives Mission; **Identification:** identifiedBy: Farah Amjad, Paris Stefanoudis, Toufiek Samaai; dateIdentified: 2022, 2023; identificationRemarks: Identified only from imagery; **Event:** samplingProtocol: Submersible OR Remotely Operated Vehicle OR Snorkel; **Record Level:** basisOfRecord: Human observation

#### Notes

Branching erect or thickly encrusting sponges. Branching sponges with irregular protrusions from the base. Surface conulose. Rugose and uneven surface. Approximately 21 cm in longest dimension. Colourations in tones of orange and brown (Fig. 28).

### Stelletta sp. indet. 2

**Figure 5.**
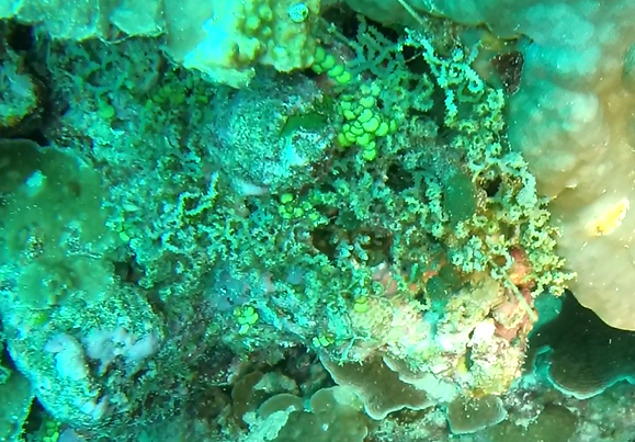
*Caulerpaserrulata* sp. inc., Addu, 30 m.

**Figure 12.**
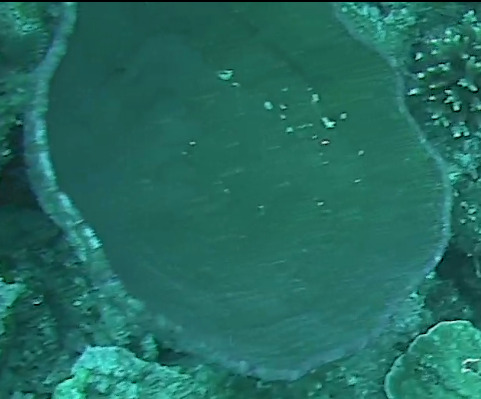
*Spheciospongiaexcentrica*, Addu, 30 m.

2D238AB3-0ABB-5CD0-80D1-CCC1EFD6645F

#### Materials

- **Type status:** Other material. **Taxon:** scientificName: *Stelletta* sp. 2; kingdom: Animalia; phylum: Porifera; class: Demospongiae-Heteroscleromorpha; order: Tetractinellida; family: Ancorinidae; genus: Stelletta; **Location:** waterBody: Indian Ocean; country: Maldives; locality: Addu; minimumDepthInMeters: 10; maximumDepthInMeters: 10; locationRemarks: Nekton Maldives Mission; **Identification:** identifiedBy: Farah Amjad, Paris Stefanoudis, Toufiek Samaai; dateIdentified: 2022, 2023; identificationRemarks: Identified only from imagery; **Event:** samplingProtocol: Submersible OR Remotely Operated Vehicle OR Snorkel; **Record Level:** basisOfRecord: Human observation

### Corallistes sp. indet. 2

0C80E71B-00E4-5B7B-B70D-08F511A6B939

#### Materials

- **Type status:** Other material. **Taxon:** scientificName: *Corallistes* sp. 2; kingdom: Animalia; phylum: Porifera; class: Demospongiae-Heteroscleromorpha; order: Tetractinellida; family: Corallistidae; genus: Corallistes; **Location:** waterBody: Indian Ocean; country: Maldives; locality: North Male’, Vaavu, Laamu, Huvadhu, Addu; minimumDepthInMeters: 52; maximumDepthInMeters: 491; locationRemarks: Nekton Maldives Mission; **Identification:** identifiedBy: Farah Amjad, Paris Stefanoudis, Toufiek Samaai; dateIdentified: 2022, 2023; identificationRemarks: Identified only from imagery; **Event:** samplingProtocol: Submersible OR Remotely Operated Vehicle OR Snorkel; **Record Level:** basisOfRecord: Human observation

### Geodia sp. indet. 3

D140D00B-7E66-5E6A-8144-75ADF4F0DD52

#### Materials

- **Type status:** Other material. **Taxon:** scientificName: *Geodia* sp. 3; kingdom: Animalia; phylum: Porifera; class: Demospongiae-Heteroscleromorpha; order: Tetractinellida; family: Geodiidae; genus: Geodia; **Location:** waterBody: Indian Ocean; country: Maldives; locality: North Male’, Laamu, Huvadhu, Addu; minimumDepthInMeters: 57; maximumDepthInMeters: 124; locationRemarks: Nekton Maldives Mission; **Identification:** identifiedBy: Farah Amjad, Paris Stefanoudis, Toufiek Samaai; dateIdentified: 2022, 2023; identificationRemarks: Identified only from imagery; **Event:** samplingProtocol: Submersible OR Remotely Operated Vehicle OR Snorkel; **Record Level:** basisOfRecord: Human observation

### Geodia sp. indet. 4

966A54B5-4A17-5481-836A-FEFBAD9382D5

#### Materials

- **Type status:** Other material. **Taxon:** scientificName: *Geodia* sp. 4; kingdom: Animalia; phylum: Porifera; class: Demospongiae-Heteroscleromorpha; order: Tetractinellida; family: Geodiidae; genus: Geodia; **Location:** waterBody: Indian Ocean; country: Maldives; locality: Vaavu, Huvadhu, Addu; minimumDepthInMeters: 59; maximumDepthInMeters: 119; locationRemarks: Nekton Maldives Mission; **Identification:** identifiedBy: Farah Amjad, Paris Stefanoudis, Toufiek Samaai; dateIdentified: 2022, 2023; identificationRemarks: Identified only from imagery; **Event:** samplingProtocol: Submersible OR Remotely Operated Vehicle OR Snorkel; **Record Level:** basisOfRecord: Human observation

### Pachastrella sp. indet. 1

0D075270-DFF7-5624-BFD8-DC96BB1C786A

#### Materials

- **Type status:** Other material. **Taxon:** scientificName: *Pachastrella* sp. 1; kingdom: Animalia; phylum: Porifera; class: Demospongiae-Heteroscleromorpha; order: Tetractinellida; family: Pachastrellidae; genus: Pachastrella; **Location:** waterBody: Indian Ocean; country: Maldives; locality: Vaavu, Laamu, Huvadhu, Addu; minimumDepthInMeters: 59; maximumDepthInMeters: 491; locationRemarks: Nekton Maldives Mission; **Identification:** identifiedBy: Farah Amjad, Paris Stefanoudis, Toufiek Samaai; dateIdentified: 2022, 2023; identificationRemarks: Identified only from imagery; **Event:** samplingProtocol: Submersible OR Remotely Operated Vehicle OR Snorkel; **Record Level:** basisOfRecord: Human observation

### Demospongiae ord. indet. sp. 1

1502088E-1DD0-5744-984E-1053614E4513

#### Materials

- **Type status:** Other material. **Taxon:** scientificName: Demospongiae sp. 1; kingdom: Animalia; phylum: Porifera; class: Demospongiae; **Location:** waterBody: Indian Ocean; country: Maldives; locality: North Male’, Vaavu, Laamu, Huvadhu, Addu; minimumDepthInMeters: 30; maximumDepthInMeters: 62; locationRemarks: Nekton Maldives Mission; **Identification:** identifiedBy: Farah Amjad, Paris Stefanoudis, Toufiek Samaai; dateIdentified: 2022, 2023; identificationRemarks: Identified only from imagery; **Event:** samplingProtocol: Submersible OR Remotely Operated Vehicle OR Snorkel; **Record Level:** basisOfRecord: Human observation

### Demospongiae ord. indet. sp. 2

96CD1425-7947-57C6-A386-7C905CA50B6D

#### Materials

- **Type status:** Other material. **Taxon:** scientificName: Demospongiae sp. 2; kingdom: Animalia; phylum: Porifera; class: Demospongiae; **Location:** waterBody: Indian Ocean; country: Maldives; locality: North Male’, Vaavu, Laamu, Huvadhu, Addu; minimumDepthInMeters: 2; maximumDepthInMeters: 490; locationRemarks: Nekton Maldives Mission; **Identification:** identifiedBy: Farah Amjad, Paris Stefanoudis, Toufiek Samaai; dateIdentified: 2022, 2023; identificationRemarks: Identified only from imagery; **Event:** samplingProtocol: Submersible OR Remotely Operated Vehicle OR Snorkel; **Record Level:** basisOfRecord: Human observation

### Demospongiae ord. indet. sp. 3

496176E3-96DC-578B-A9CA-EA9D52030E94

#### Materials

- **Type status:** Other material. **Taxon:** scientificName: Demospongiae sp. 3; kingdom: Animalia; phylum: Porifera; class: Demospongiae; scientificNameAuthorship: Sollas, 1885; **Location:** waterBody: Indian Ocean; country: Maldives; locality: North Male’, Vaavu, Laamu, Huvadhu, Addu; minimumDepthInMeters: 2; maximumDepthInMeters: 120; locationRemarks: Nekton Maldives Mission; **Identification:** identifiedBy: Farah Amjad, Paris Stefanoudis, Toufiek Samaai; dateIdentified: 2022, 2023; identificationRemarks: Identified only from imagery; **Event:** samplingProtocol: Submersible OR Remotely Operated Vehicle OR Snorkel; **Record Level:** basisOfRecord: Human observation

### Demospongiae ord. indet. sp. 4

1AE5BF90-B473-5BBE-97B4-F25EB88AF1C0

#### Materials

- **Type status:** Other material. **Taxon:** scientificName: Demospongiae sp. 4; kingdom: Animalia; phylum: Porifera; class: Demospongiae; scientificNameAuthorship: Sollas, 1885; **Location:** waterBody: Indian Ocean; country: Maldives; locality: North Male’, Vaavu, Laamu, Huvadhu, Addu; minimumDepthInMeters: 8; maximumDepthInMeters: 491; locationRemarks: Nekton Maldives Mission; **Identification:** identifiedBy: Farah Amjad, Paris Stefanoudis, Toufiek Samaai; dateIdentified: 2022, 2023; identificationRemarks: Identified only from imagery; **Event:** samplingProtocol: Submersible OR Remotely Operated Vehicle OR Snorkel; **Record Level:** basisOfRecord: Human observation

### Demospongiae ord. indet. sp. 16

Sollas, 1885

0F39D357-5EE5-51B4-A1C4-0C83AE2123BE

#### Materials

- **Type status:** Other material. **Taxon:** scientificName: Demospongiae sp. 16; kingdom: Animalia; phylum: Porifera; class: Demospongiae; **Location:** waterBody: Indian Ocean; country: Maldives; locality: Laamu, Huvadhu, Addu; minimumDepthInMeters: 57; maximumDepthInMeters: 124; locationRemarks: Nekton Maldives Mission; **Identification:** identifiedBy: Farah Amjad, Paris Stefanoudis, Toufiek Samaai; dateIdentified: 2022, 2023; identificationRemarks: Identified only from imagery; **Event:** samplingProtocol: Submersible OR Remotely Operated Vehicle OR Snorkel; **Record Level:** basisOfRecord: Human observation

### Demospongiae ord. indet. sp. 17

BA37F13B-B97B-5CC2-9AE3-D20384C91BAB

#### Materials

- **Type status:** Other material. **Taxon:** scientificName: Demospongiae sp. 17; kingdom: Animalia; phylum: Porifera; class: Demospongiae; **Location:** waterBody: Indian Ocean; country: Maldives; locality: Addu; minimumDepthInMeters: 59; maximumDepthInMeters: 60; locationRemarks: Nekton Maldives Mission; **Identification:** identifiedBy: Farah Amjad, Paris Stefanoudis, Toufiek Samaai; dateIdentified: 2022, 2023; identificationRemarks: Identified only from imagery; **Event:** samplingProtocol: Submersible OR Remotely Operated Vehicle OR Snorkel; **Record Level:** basisOfRecord: Human observation

### Demospongiae ord. indet. sp. 18

20B94C29-539F-596B-A701-A1178C36CF7C

#### Materials

- **Type status:** Other material. **Taxon:** scientificName: Demospongiae sp. 18; kingdom: Animalia; phylum: Porifera; class: Demospongiae; **Location:** waterBody: Indian Ocean; country: Maldives; locality: North Male’, Huvadhu; minimumDepthInMeters: 10; maximumDepthInMeters: 30; locationRemarks: Nekton Maldives Mission; **Identification:** identifiedBy: Farah Amjad, Paris Stefanoudis, Toufiek Samaai; dateIdentified: 2022, 2023; identificationRemarks: Identified only from imagery; **Event:** samplingProtocol: Submersible OR Remotely Operated Vehicle OR Snorkel; **Record Level:** basisOfRecord: Human observation

### Demospongiae ord. indet. sp. 19

6F9A02EE-E57F-5FE8-AB26-2E470504186A

#### Materials

- **Type status:** Other material. **Taxon:** scientificName: Demospongiae sp. 19; kingdom: Animalia; phylum: Porifera; class: Demospongiae; **Location:** waterBody: Indian Ocean; country: Maldives; locality: Huvadhu; minimumDepthInMeters: 489; maximumDepthInMeters: 489; locationRemarks: Nekton Maldives Mission; **Identification:** identifiedBy: Farah Amjad, Paris Stefanoudis, Toufiek Samaai; dateIdentified: 2022, 2023; identificationRemarks: Identified only from imagery; **Event:** samplingProtocol: Submersible OR Remotely Operated Vehicle OR Snorkel; **Record Level:** basisOfRecord: Human observation

### Hyalonema (Paradisconema) alcocki

Schulze, 1895

CEE25A94-6085-525A-BCF0-2475AD5E99B2

#### Materials

- **Type status:** Other material. **Taxon:** scientificName: Hyalonema (Paradisconema) alcocki; kingdom: Animalia; phylum: Porifera; class: Hexactinellida; order: Amphidiscosida; family: Hyalonematidae; genus: Hyalonema; scientificNameAuthorship: Schulze, 1895; **Location:** waterBody: Indian Ocean; country: Maldives; locality: Addu, Vaavu; minimumDepthInMeters: 248; maximumDepthInMeters: 497; locationRemarks: Nekton Maldives Mission; **Identification:** identifiedBy: Farah Amjad, Paris Stefanoudis, Toufiek Samaai; dateIdentified: 2022, 2023; identificationRemarks: Identified only from imagery; **Event:** samplingProtocol: Submersible OR Remotely Operated Vehicle OR Snorkel; **Record Level:** basisOfRecord: Human observation

#### Notes

Stalked with globular spherical-shaped sponges with varying length of stalk. Approximately 17 cm tall (Fig. 42).

### Semperella cucumis

Schulze, 1895

8B08C5E2-D8BE-5C8F-B36D-3B755BB59A20

#### Materials

- **Type status:** Other material. **Taxon:** scientificName: *Semperellacucumis*; kingdom: Animalia; phylum: Porifera; class: Hexactinellida; order: Amphidiscosida; family: Pheronematidae; genus: Semperella; scientificNameAuthorship: Schulze, 1895; **Location:** waterBody: Indian Ocean; country: Maldives; locality: Huvadhu; minimumDepthInMeters: 497; maximumDepthInMeters: 500; locationRemarks: Nekton Maldives Mission; **Identification:** identifiedBy: Farah Amjad, Paris Stefanoudis, Toufiek Samaai; dateIdentified: 2022, 2023; identificationRemarks: Identified only from imagery; **Event:** samplingProtocol: Submersible OR Remotely Operated Vehicle OR Snorkel; **Record Level:** basisOfRecord: Human observation

#### Notes

Elongated and columnar glass sponges with a short stalk and one central oscule at the tip. Approximately 17 cm tall. Collected specimen (Fig. 43).

### Farrea sp. indet. 1

2D1627B7-728B-50F3-BC51-0E471A23D467

#### Materials

- **Type status:** Other material. **Taxon:** scientificName: *Farrea* sp. 1; kingdom: Animalia; phylum: Porifera; class: Hexactinellida; order: Sceptrulophora; family: Farreidae; genus: Farrea; **Location:** waterBody: Indian Ocean; country: Maldives; locality: Vaavu; minimumDepthInMeters: 488; maximumDepthInMeters: 489; locationRemarks: Nekton Maldives Mission; **Identification:** identifiedBy: Farah Amjad, Paris Stefanoudis, Toufiek Samaai; dateIdentified: 2022, 2023; identificationRemarks: Identified only from imagery; **Event:** samplingProtocol: Submersible OR Remotely Operated Vehicle OR Snorkel; **Record Level:** basisOfRecord: Human observation

### Farrea sp. indet. 2

CCCC4004-83A9-5EB9-92A5-B6806FA89507

#### Materials

- **Type status:** Other material. **Occurrence:** occurrenceID: 941C7281-7FAE-5139-A7C7-24A0E3E5EC67; **Taxon:** scientificName: *Farrea* sp. 2; kingdom: Animalia; phylum: Porifera; class: Hexactinellida; order: Sceptrulophora; family: Farreidae; genus: Farrea; **Location:** waterBody: Indian Ocean; country: Maldives; locality: Laamu; minimumDepthInMeters: 490; maximumDepthInMeters: 491; locationRemarks: Nekton Maldives Mission; **Identification:** identifiedBy: Farah Amjad, Paris Stefanoudis, Toufiek Samaai; dateIdentified: 2022, 2023; identificationRemarks: Identified only from imagery; **Event:** samplingProtocol: Submersible OR Remotely Operated Vehicle OR Snorkel; **Record Level:** basisOfRecord: Human observation

### Pleurochorium annandalei

(Kirkpatrick, 1908)

4E4F269C-AD14-50CA-B0F1-A329F4732661

#### Materials

- **Type status:** Other material. **Taxon:** scientificName: *Pleurochoriumannandalei*; kingdom: Animalia; phylum: Porifera; class: Hexactinellida; order: Sceptrulophora; family: Euretidae; genus: Pleurochorium; scientificNameAuthorship: (Kirkpatrick, 1908); **Location:** waterBody: Indian Ocean; country: Maldives; locality: Huvadhu Fuvahmulah; minimumDepthInMeters: 489; maximumDepthInMeters: 489; locationRemarks: Nekton Maldives Mission; **Identification:** identifiedBy: Farah Amjad, Paris Stefanoudis, Toufiek Samaai; dateIdentified: 2022, 2023; identificationRemarks: Identified only from imagery; **Event:** samplingProtocol: Submersible OR Remotely Operated Vehicle OR Snorkel; **Record Level:** basisOfRecord: Human observation

#### Notes

Arborescent erect branching glass sponges with clear translucent-looking structure. Approximately 16 cm in the longest dimension. Collected specimen (Fig. 46).

### Hexactinellida ord. indet. sp. 1

4CBE2D68-DB7F-57DD-AB91-3A5ECB0EE617

#### Materials

- **Type status:** Other material. **Taxon:** scientificName: Hexactinellida sp. 1; kingdom: Animalia; phylum: Porifera; class: Hexactinellida; **Location:** waterBody: Indian Ocean; country: Maldives; locality: North Male’, Vaavu; minimumDepthInMeters: 488; maximumDepthInMeters: 489; locationRemarks: Nekton Maldives Mission; **Identification:** identifiedBy: Farah Amjad, Paris Stefanoudis, Toufiek Samaai; dateIdentified: 2022, 2023; identificationRemarks: Identified only from imagery; **Event:** samplingProtocol: Submersible OR Remotely Operated Vehicle OR Snorkel; **Record Level:** basisOfRecord: Human observation

### Radianthus magnifica

(Quoy & Gaimard, 1833)

6BD04466-F151-5359-A0CF-83FA4590D840

#### Materials

- **Type status:** Other material. **Taxon:** scientificName: *Radianthusmagnifica*; kingdom: Animalia; phylum: Cnidaria; class: Anthozoa-Hexacorallia; order: Actiniaria; family: Stichodactylidae; genus: Radianthus; scientificNameAuthorship: (Quoy & Gaimard, 1833); **Location:** waterBody: Indian Ocean; country: Maldives; locality: Addu, Fuvahmulah; minimumDepthInMeters: 10; maximumDepthInMeters: 30; locationRemarks: Nekton Maldives Mission; **Identification:** identifiedBy: Farah Amjad, Paris Stefanoudis; dateIdentified: 2022, 2023; identificationRemarks: Identified only from imagery; **Event:** samplingProtocol: Submersible OR Remotely Operated Vehicle OR Snorkel; **Record Level:** basisOfRecord: Human observation

#### Notes

Oral disc covered in densely packed long tentacles with blunt or slightly swollen tips. Column exposed with a flared look and bright coloured often in pink and purple shades (Fig. 48).

### Heteractis aurora

(Quoy & Gaimard, 1833)

F2F5A6A3-6691-51E9-A882-F68271A31C5A

#### Materials

- **Type status:** Other material. **Taxon:** scientificName: *Heteractisaurora*; kingdom: Animalia; phylum: Cnidaria; class: Anthozoa-Hexacorallia; order: Actiniaria; family: Heteractidae; genus: Heteractis; scientificNameAuthorship: (Quoy & Gaimard, 1833); **Location:** waterBody: Indian Ocean; country: Maldives; locality: Vaavu, Laamu, Huvadhu; minimumDepthInMeters: 30; maximumDepthInMeters: 30; locationRemarks: Nekton Maldives Mission; **Identification:** identifiedBy: Farah Amjad, Paris Stefanoudis; dateIdentified: 2022, 2023; identificationRemarks: Identified only from imagery; **Event:** samplingProtocol: Submersible OR Remotely Operated Vehicle OR Snorkel; **Record Level:** basisOfRecord: Human observation

#### Notes

This genus contains many species with similar morphology. Characterised by an undulated wide oral disc with short marginal tentacles on the outer edge. Diameter of oral disc ~ 12 cm. Colouration highly variable in brown and beige shades (Fig. 49).

### Stichodactyla mertensii

Brandt, 1835

1CC94A19-6B09-59D8-8DEF-1317519CDAC2

#### Materials

- **Type status:** Other material. **Taxon:** scientificName: *Stichodactylamertensii*; kingdom: Animalia; phylum: Cnidaria; class: Anthozoa-Hexacorallia; order: Actiniaria; family: Stichodactylidae; genus: Stichodactyla; scientificNameAuthorship: Brandt, 1835; **Location:** waterBody: Indian Ocean; country: Maldives; locality: Laamu; minimumDepthInMeters: 10; maximumDepthInMeters: 30; locationRemarks: Nekton Maldives Mission; **Identification:** identifiedBy: Farah Amjad, Paris Stefanoudis; dateIdentified: 2022, 2023; identificationRemarks: Identified only from imagery; **Event:** samplingProtocol: Submersible OR Remotely Operated Vehicle OR Snorkel; **Record Level:** basisOfRecord: Human observation

#### Notes

**Figure 50.**
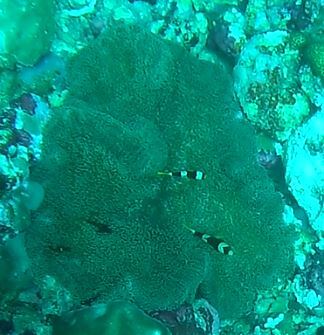
*Stichodactylamertensii* with host anemone fish *Amphiprionclarkii*, Laamu, 10 m.

Large anemone with densely packed short, finger-like or club-shaped tentacles. Slightly undulated ovular disc, growth sometimes following the profile of substratum and attached by adhesive verrucae. Average diameter of oral disc of measured individuals ~ 4 cm, although pcitured specimen was closer to 10 cm (Fig. 50).

### Actiniaria fam. indet. sp. 2

7419CEC1-5A40-5A6F-9EA4-4B307B516732

#### Materials

- **Type status:** Other material. **Taxon:** scientificName: Actiniaria sp. 2; kingdom: Animalia; phylum: Cnidaria; class: Anthozoa-Hexacorallia; order: Actiniaria; **Location:** waterBody: Indian Ocean; country: Maldives; locality: North Male’, Huvadhu, Addu; minimumDepthInMeters: 247; maximumDepthInMeters: 489; locationRemarks: Nekton Maldives Mission; **Identification:** identifiedBy: Farah Amjad, Paris Stefanoudis; dateIdentified: 2022, 2023; identificationRemarks: Identified only from imagery; **Event:** samplingProtocol: Submersible OR Remotely Operated Vehicle OR Snorkel; **Record Level:** basisOfRecord: Human observation

### Actiniaria fam. indet. sp. 4

7BF587B3-458D-50FC-8A15-A647F5318A57

#### Materials

- **Type status:** Other material. **Taxon:** scientificName: Actiniaria sp. 4; kingdom: Animalia; phylum: Cnidaria; class: Anthozoa-Hexacorallia; order: Actiniaria; **Location:** waterBody: Indian Ocean; country: Maldives; locality: North Male’, Vaavu, Addu; minimumDepthInMeters: 247; maximumDepthInMeters: 489; locationRemarks: Nekton Maldives Mission; **Identification:** identifiedBy: Farah Amjad, Paris Stefanoudis; dateIdentified: 2022, 2023; identificationRemarks: Identified only from imagery; **Event:** samplingProtocol: Submersible OR Remotely Operated Vehicle OR Snorkel; **Record Level:** basisOfRecord: Human observation

### Actiniaria fam. indet. sp. 5

1DEE2A57-780E-53CE-A172-98EC557A1AE8

#### Materials

- **Type status:** Other material. **Taxon:** scientificName: Actiniaria sp. 5; kingdom: Animalia; phylum: Cnidaria; class: Anthozoa-Hexacorallia; order: Actiniaria; **Location:** waterBody: Indian Ocean; country: Maldives; locality: North Male’, Addu; minimumDepthInMeters: 490; maximumDepthInMeters: 490; locationRemarks: Nekton Maldives Mission; **Identification:** identifiedBy: Farah Amjad, Paris Stefanoudis; dateIdentified: 2022, 2023; identificationRemarks: Identified only from imagery; **Event:** samplingProtocol: Submersible OR Remotely Operated Vehicle OR Snorkel; **Record Level:** basisOfRecord: Human observation

### Actiniaria fam. indet. sp. 6

95CFD28E-7063-5E73-988D-EA1443F61C08

#### Materials

- **Type status:** Other material. **Taxon:** scientificName: Actiniaria sp. 6; kingdom: Animalia; phylum: Cnidaria; class: Anthozoa-Hexacorallia; order: Actiniaria; **Location:** waterBody: Indian Ocean; country: Maldives; locality: North Male’, Vaavu, Addu; minimumDepthInMeters: 248; maximumDepthInMeters: 490; locationRemarks: Nekton Maldives Mission; **Identification:** identifiedBy: Farah Amjad, Paris Stefanoudis; dateIdentified: 2022, 2023; identificationRemarks: Identified only from imagery; **Event:** samplingProtocol: Submersible OR Remotely Operated Vehicle OR Snorkel; **Record Level:** basisOfRecord: Human observation

### Actiniaria fam. indet. sp. 7

7FC75BE2-F2EE-53B8-8EDB-8B8728794735

#### Materials

- **Type status:** Other material. **Taxon:** scientificName: Actiniaria sp. 7; kingdom: Animalia; phylum: Cnidaria; class: Anthozoa-Hexacorallia; order: Actiniaria; **Location:** waterBody: Indian Ocean; country: Maldives; locality: Addu; minimumDepthInMeters: 489; maximumDepthInMeters: 491; locationRemarks: Nekton Maldives Mission; **Identification:** identifiedBy: Farah Amjad, Paris Stefanoudis; dateIdentified: 2022, 2023; identificationRemarks: Identified only from imagery; **Event:** samplingProtocol: Submersible OR Remotely Operated Vehicle OR Snorkel; **Record Level:** basisOfRecord: Human observation

### Actiniaria fam. indet. sp. 8

D01EFAED-2F20-55D7-9152-9F87273F4F62

#### Materials

- **Type status:** Other material. **Taxon:** scientificName: Actiniaria sp. 8; kingdom: Animalia; phylum: Cnidaria; class: Anthozoa-Hexacorallia; order: Actiniaria; **Location:** waterBody: Indian Ocean; country: Maldives; locality: North Male’, Laamu; minimumDepthInMeters: 250; maximumDepthInMeters: 489; locationRemarks: Nekton Maldives Mission; **Identification:** identifiedBy: Farah Amjad, Paris Stefanoudis; dateIdentified: 2022, 2023; identificationRemarks: Identified only from imagery; **Event:** samplingProtocol: Submersible OR Remotely Operated Vehicle OR Snorkel; **Record Level:** basisOfRecord: Human observation

### Acropora sp. indet. 1

62EC6D5F-A114-5BDD-BC31-3B777F769EE2

#### Materials

- **Type status:** Other material. **Taxon:** scientificName: *Acropora* sp. 1; kingdom: Animalia; phylum: Cnidaria; class: Anthozoa-Hexacorallia; order: Scleractinia; family: Acroporidae; genus: Acropora; **Location:** waterBody: Indian Ocean; country: Maldives; locality: Vaavu, Laamu, Huvadhu; minimumDepthInMeters: 10; maximumDepthInMeters: 30; locationRemarks: Nekton Maldives Mission; **Identification:** identifiedBy: Farah Amjad, Paris Stefanoudis, Mariyam Shidha Afzal, Hana Amir; dateIdentified: 2022, 2023; identificationRemarks: Identified only from imagery; **Event:** samplingProtocol: Submersible OR Remotely Operated Vehicle OR Snorkel; **Record Level:** basisOfRecord: Human observation

### Acropora sp. indet. 2

88FB9D43-C496-5831-AA2D-ACF689B0C589

#### Materials

- **Type status:** Other material. **Taxon:** scientificName: *Acropora* sp. 2; kingdom: Animalia; phylum: Cnidaria; class: Anthozoa-Hexacorallia; order: Scleractinia; family: Acroporidae; genus: Acropora; **Location:** waterBody: Indian Ocean; country: Maldives; locality: North Male’, Vaavu, Laamu, Huvadhu, Addu; minimumDepthInMeters: 2; maximumDepthInMeters: 30; locationRemarks: Nekton Maldives Mission; **Identification:** identifiedBy: Farah Amjad, Paris Stefanoudis, Mariyam Shidha Afzal, Hana Amir; dateIdentified: 2022, 2023; identificationRemarks: Identified only from imagery; **Event:** samplingProtocol: Submersible OR Remotely Operated Vehicle OR Snorkel; **Record Level:** basisOfRecord: Human observation

### Acropora sp. indet. 3

08C0CA01-E694-5E7B-9BF4-20E4F2B41053

#### Materials

- **Type status:** Other material. **Taxon:** scientificName: Acropora sp. 3; kingdom: Animalia; phylum: Cnidaria; class: Anthozoa-Hexacorallia; order: Scleractinia; family: Acroporidae; genus: Acropora; scientificNameAuthorship: Oken, 1815; **Location:** waterBody: Indian Ocean; country: Maldives; locality: North Male’, Vaavu, Laamu, Huvadhu, Addu; minimumDepthInMeters: 2; maximumDepthInMeters: 30; locationRemarks: Nekton Maldives Mission; **Identification:** identifiedBy: Farah Amjad, Paris Stefanoudis, Mariyam Shidha Afzal, Hana Amir; dateIdentified: 2022, 2023; identificationRemarks: Identified only from imagery; **Event:** samplingProtocol: Submersible OR Remotely Operated Vehicle OR Snorkel; **Record Level:** basisOfRecord: Human observation

### Acropora sp. indet. 4

42858ABB-167D-5325-B994-9FA1D979AFB5

#### Materials

- **Type status:** Other material. **Taxon:** scientificName: *Acropora* sp. 4; kingdom: Animalia; phylum: Cnidaria; class: Anthozoa-Hexacorallia; order: Scleractinia; family: Acroporidae; genus: Acropora; **Location:** waterBody: Indian Ocean; country: Maldives; locality: North Male’, Vaavu, Laamu, Huvadhu, Addu; minimumDepthInMeters: 2; maximumDepthInMeters: 30; locationRemarks: Nekton Maldives Mission; **Identification:** identifiedBy: Farah Amjad, Paris Stefanoudis, Mariyam Shidha Afzal, Hana Amir; dateIdentified: 2022, 2023; identificationRemarks: Identified only from imagery; **Event:** samplingProtocol: Submersible OR Remotely Operated Vehicle OR Snorkel; **Record Level:** basisOfRecord: Human observation

### Acropora sp. indet. 5

81E1BB31-FFE7-5732-AB6A-F38938131CF4

#### Materials

- **Type status:** Other material. **Taxon:** scientificName: *Acropora* sp. 5; kingdom: Animalia; phylum: Cnidaria; class: Anthozoa-Hexacorallia; order: Scleractinia; family: Acroporidae; genus: Acropora; **Location:** waterBody: Indian Ocean; country: Maldives; locality: Laamu, Addu; minimumDepthInMeters: 10; maximumDepthInMeters: 30; locationRemarks: Nekton Maldives Mission; **Identification:** identifiedBy: Farah Amjad, Paris Stefanoudis, Mariyam Shidha Afzal, Hana Amir; dateIdentified: 2022, 2023; identificationRemarks: Identified only from imagery; **Event:** samplingProtocol: Submersible OR Remotely Operated Vehicle OR Snorkel; **Record Level:** basisOfRecord: Human observation

### Acropora sp. indet. 6

68206582-41BA-5B4A-9F06-52A481A8A2F4

#### Materials

- **Type status:** Other material. **Taxon:** scientificName: *Acropora* sp. 6; kingdom: Animalia; phylum: Cnidaria; class: Anthozoa-Hexacorallia; order: Scleractinia; family: Acroporidae; genus: Acropora; **Location:** waterBody: Indian Ocean; country: Maldives; locality: Laamu; minimumDepthInMeters: 10; maximumDepthInMeters: 10; locationRemarks: Nekton Maldives Mission; **Identification:** identifiedBy: Farah Amjad, Paris Stefanoudis, Mariyam Shidha Afzal, Hana Amir; dateIdentified: 2022, 2023; identificationRemarks: Identified only from imagery; **Event:** samplingProtocol: Submersible OR Remotely Operated Vehicle OR Snorkel; **Record Level:** basisOfRecord: Human observation

### Acropora sp. indet. 7

6653225C-B1F0-5D26-B683-92555A9E8844

#### Materials

- **Type status:** Other material. **Taxon:** scientificName: *Acropora* sp. 7; kingdom: Animalia; phylum: Cnidaria; class: Anthozoa-Hexacorallia; order: Scleractinia; family: Acroporidae; genus: Acropora; **Location:** waterBody: Indian Ocean; country: Maldives; locality: North Male’, Laamu, Huvadhu, Addu; minimumDepthInMeters: 2; maximumDepthInMeters: 10; locationRemarks: Nekton Maldives Mission; **Identification:** identifiedBy: Farah Amjad, Paris Stefanoudis, Mariyam Shidha Afzal, Hana Amir; dateIdentified: 2022, 2023; identificationRemarks: Identified only from imagery; **Event:** samplingProtocol: Submersible OR Remotely Operated Vehicle OR Snorkel; **Record Level:** basisOfRecord: Human observation

### Astreopora sp. indet.

9EC91037-0C38-59FC-BBF2-228638DE97D8

#### Materials

- **Type status:** Other material. **Taxon:** scientificName: *Astreopora* sp.; kingdom: Animalia; phylum: Cnidaria; class: Anthozoa-Hexacorallia; order: Scleractinia; family: Acroporidae; genus: Astreopora; **Location:** waterBody: Indian Ocean; country: Maldives; locality: North Male’, Vaavu, Laamu, Huvadhu, Addu; minimumDepthInMeters: 2; maximumDepthInMeters: 30; locationRemarks: Nekton Maldives Mission; **Identification:** identifiedBy: Farah Amjad, Paris Stefanoudis, Mariyam Shidha Afzal, Hana Amir; dateIdentified: 2022, 2023; identificationRemarks: Identified only from imagery; **Event:** samplingProtocol: Submersible OR Remotely Operated Vehicle OR Snorkel; **Record Level:** basisOfRecord: Human observation

### Montipora sp. indet. 1

3C63F3B7-9626-586C-82B8-30E443104D49

#### Materials

- **Type status:** Other material. **Taxon:** scientificName: *Montipora* sp. 1; kingdom: Animalia; phylum: Cnidaria; class: Anthozoa-Hexacorallia; order: Scleractinia; family: Acroporidae; genus: Montipora; **Location:** waterBody: Indian Ocean; country: Maldives; locality: North Male’, Vaavu, Laamu, Huvadhu, Addu; minimumDepthInMeters: 2; maximumDepthInMeters: 61; locationRemarks: Nekton Maldives Mission; **Identification:** identifiedBy: Farah Amjad, Paris Stefanoudis, Mariyam Shidha Afzal, Hana Amir; dateIdentified: 2022, 2023; identificationRemarks: Identified only from imagery; **Event:** samplingProtocol: Submersible OR Remotely Operated Vehicle OR Snorkel; **Record Level:** basisOfRecord: Human observation

### Montipora sp. indet. 2

09D05BC9-C20C-5238-AA1F-D9F0FE52B0C5

#### Materials

- **Type status:** Other material. **Taxon:** scientificName: *Montipora* sp. 2; kingdom: Animalia; phylum: Cnidaria; class: Anthozoa-Hexacorallia; order: Scleractinia; family: Acroporidae; genus: Montipora; **Location:** waterBody: Indian Ocean; country: Maldives; locality: North Male’, Vaavu, Laamu, Huvadhu, Addu, Fuvahmulah; minimumDepthInMeters: 2; maximumDepthInMeters: 61; locationRemarks: Nekton Maldives Mission; **Identification:** identifiedBy: Farah Amjad, Paris Stefanoudis, Mariyam Shidha Afzal, Hana Amir; dateIdentified: 2022, 2023; identificationRemarks: Identified only from imagery; **Event:** samplingProtocol: Submersible OR Remotely Operated Vehicle OR Snorkel; **Record Level:** basisOfRecord: Human observation

### Gardineroseris planulata

(Dana, 1846)

34AC6BFD-0415-5B21-906A-4999DF231FD9

#### Materials

- **Type status:** Other material. **Taxon:** scientificName: *Gardineroserisplanulata*; kingdom: Animalia; phylum: Cnidaria; class: Anthozoa-Hexacorallia; order: Scleractinia; family: Agariciidae; genus: Gardineroseris; scientificNameAuthorship: (Dana, 1846); **Location:** waterBody: Indian Ocean; country: Maldives; locality: Vaavu, Laamu, Huvadhu; minimumDepthInMeters: 10; maximumDepthInMeters: 30; locationRemarks: Nekton Maldives Mission; **Identification:** identifiedBy: Farah Amjad, Paris Stefanoudis, Mariyam Shidha Afzal, Hana Amir; dateIdentified: 2022, 2023; identificationRemarks: Identified only from imagery; **Event:** samplingProtocol: Submersible OR Remotely Operated Vehicle OR Snorkel; **Record Level:** basisOfRecord: Human observation

#### Notes

**Figure 67.**
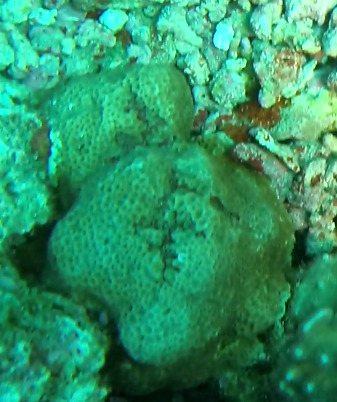
*Gardineroserisplanulata*, Laamu, 10 m.

Rounded colonies that are massive or sub-massive. Corallites lie in deep depressions with sharp-edged walls that give the colony a scalloped, honey-comb appearance. Deeper depressions of the corallites and lack of paliform lobes make it distinctive from *Coelastrea*. Colony size ~ 19 cm in the longest dimension. Colours range from yellowish to shades of light and dark brown. Officially, only this species of *Gardineroseris* has been officially recorded in Maldivian waters (Fig. 67).

### Leptoseris sp. indet.

2A189BEA-0AE7-5154-9627-62A87EA0FFB8

#### Materials

- **Type status:** Other material. **Taxon:** scientificName: *Leptoseris* sp.; kingdom: Animalia; phylum: Cnidaria; class: Anthozoa-Hexacorallia; order: Scleractinia; family: Agariciidae; genus: Leptoseris; **Location:** waterBody: Indian Ocean; country: Maldives; locality: North Male’, Vaavu, Laamu, Huvadhu, Addu; minimumDepthInMeters: 10; maximumDepthInMeters: 61; locationRemarks: Nekton Maldives Mission; **Identification:** identifiedBy: Farah Amjad, Paris Stefanoudis, Mariyam Shidha Afzal, Hana Amir; dateIdentified: 2022, 2023; identificationRemarks: Identified only from imagery; **Event:** samplingProtocol: Submersible OR Remotely Operated Vehicle OR Snorkel; **Record Level:** basisOfRecord: Human observation

### Pavona varians

(Verrill, 1864)

8A950D5B-6172-5901-8425-A991A856E1D7

#### Materials

- **Type status:** Other material. **Taxon:** scientificName: *Pavonavarians*; kingdom: Animalia; phylum: Cnidaria; class: Anthozoa-Hexacorallia; order: Scleractinia; family: Agariciidae; genus: Pavona; **Location:** waterBody: Indian Ocean; country: Maldives; locality: North Male’, Laamu, Huvadhu, Addu; minimumDepthInMeters: 10; maximumDepthInMeters: 30; locationRemarks: Nekton Maldives Mission; **Identification:** identifiedBy: Farah Amjad, Paris Stefanoudis, Mariyam Shidha Afzal, Hana Amir; dateIdentified: 2022, 2023; identificationRemarks: Identified only from imagery; **Event:** samplingProtocol: Submersible OR Remotely Operated Vehicle OR Snorkel; **Record Level:** basisOfRecord: Human observation

### Pavona sp. indet. 2

23698241-FF78-5805-806D-4608DB0F46D3

#### Materials

- **Type status:** Other material. **Taxon:** scientificName: *Pavona* sp. 2; kingdom: Animalia; phylum: Cnidaria; class: Anthozoa-Hexacorallia; order: Scleractinia; family: Agariciidae; genus: Pavona; **Location:** waterBody: Indian Ocean; country: Maldives; locality: North Male’, Vaavu, Laamu, Huvadhu, Addu; minimumDepthInMeters: 2; maximumDepthInMeters: 30; locationRemarks: Nekton Maldives Mission; **Identification:** identifiedBy: Farah Amjad, Paris Stefanoudis, Mariyam Shidha Afzal, Hana Amir; dateIdentified: 2022, 2023; identificationRemarks: Identified only from imagery; **Event:** samplingProtocol: Submersible OR Remotely Operated Vehicle OR Snorkel; **Record Level:** basisOfRecord: Human observation

### Dendrophyllia sp. indet.

39955BB5-E1B8-5A06-9AC1-BC73CF0B504E

#### Materials

- **Type status:** Other material. **Taxon:** scientificName: *Dendrophyllia* sp.; kingdom: Animalia; phylum: Cnidaria; class: Anthozoa-Hexacorallia; order: Scleractinia; family: Dendrophylliidae; genus: Dendrophyllia; **Location:** waterBody: Indian Ocean; country: Maldives; locality: North Male’, Vaavu, Laamu, Addu, Fuvahmulah; minimumDepthInMeters: 249; maximumDepthInMeters: 490; locationRemarks: Nekton Maldives Mission; **Identification:** identifiedBy: Farah Amjad, Paris Stefanoudis, Mariyam Shidha Afzal, Hana Amir; dateIdentified: 2022, 2023; identificationRemarks: Identified only from imagery; **Event:** samplingProtocol: Submersible OR Remotely Operated Vehicle OR Snorkel; **Record Level:** basisOfRecord: Human observation

### Tubastraea sp. indet.

21F8DDA9-6C0A-59A0-9265-02A50C9CB5D5

#### Materials

- **Type status:** Other material. **Occurrence:** occurrenceID: B; **Taxon:** scientificName: *Tubastraea* sp.; kingdom: Animalia; phylum: Cnidaria; class: Anthozoa-Hexacorallia; order: Scleractinia; family: Dendrophylliidae; genus: Tubastraea; **Location:** waterBody: Indian Ocean; country: Maldives; locality: North Male’, Vaavu, Huvadhu; minimumDepthInMeters: 10; maximumDepthInMeters: 62; locationRemarks: Nekton Maldives Mission; **Identification:** identifiedBy: Farah Amjad, Paris Stefanoudis, Mariyam Shidha Afzal, Hana Amir; dateIdentified: 2022, 2023; identificationRemarks: Identified only from imagery; **Event:** samplingProtocol: Submersible OR Remotely Operated Vehicle OR Snorkel; **Record Level:** basisOfRecord: Human observation

### Turbinaria sp. indet.

479D5BF7-31E4-544F-A4A7-297DCAD5FE09

#### Materials

- **Type status:** Other material. **Taxon:** scientificName: *Turbinaria* sp.; kingdom: Animalia; phylum: Cnidaria; class: Anthozoa-Hexacorallia; order: Scleractinia; family: Dendrophylliidae; genus: Turbinaria; **Location:** waterBody: Indian Ocean; country: Maldives; locality: Vaavu, Huvadhu; minimumDepthInMeters: 10; maximumDepthInMeters: 30; locationRemarks: Nekton Maldives Mission; **Identification:** identifiedBy: Farah Amjad, Paris Stefanoudis, Mariyam Shidha Afzal, Hana Amir; dateIdentified: 2022, 2023; identificationRemarks: Identified only from imagery; **Event:** samplingProtocol: Submersible OR Remotely Operated Vehicle OR Snorkel; **Record Level:** basisOfRecord: Human observation

### Duncanopsammia peltata

(Esper, 1790)

3D78D9AE-AA48-53F4-9902-49E0949D223E

#### Materials

- **Type status:** Other material. **Taxon:** scientificName: *Duncanopsammiapeltata*; kingdom: Animalia; phylum: Cnidaria; class: Anthozoa-Hexacorallia; order: Scleractinia; family: Dendrophylliidae; genus: Duncanopsammia; scientificNameAuthorship: (Esper, 1790); **Location:** waterBody: Indian Ocean; country: Maldives; locality: Huvadhu; minimumDepthInMeters: 30; maximumDepthInMeters: 30; locationRemarks: Nekton Maldives Mission; **Identification:** identifiedBy: Farah Amjad, Paris Stefanoudis, Mariyam Shidha Afzal, Hana Amir; dateIdentified: 2022, 2023; identificationRemarks: Identified only from imagery; **Event:** samplingProtocol: Submersible OR Remotely Operated Vehicle OR Snorkel; **Record Level:** basisOfRecord: Human observation

#### Notes

**Figure 74.**
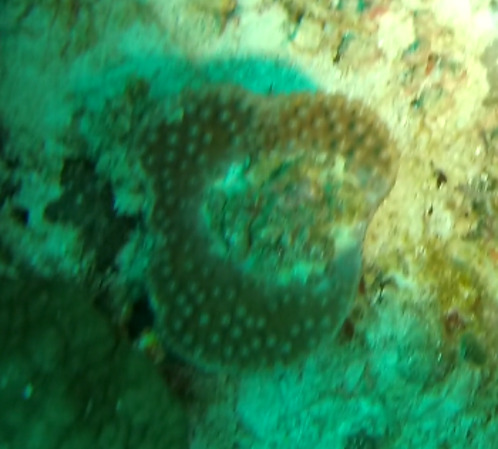
*Duncanopsammiapeltata*, Huvadhu, 30 m.

Large immersed corallites often forming plates. Previously under coral genera Turbinaria. Only one species is recorded under this genera from Maldives (Fig. 74).

### Balanophyllia sp. indet.

135DE6F2-177E-5910-B446-735317D6BCDF

#### Materials

- **Type status:** Other material. **Taxon:** scientificName: Balanophyllia sp.; kingdom: Animalia; phylum: Cnidaria; class: Anthozoa-Hexacorallia; order: Scleractinia; family: Dendrophylliidae; genus: Balanophyllia; **Location:** waterBody: Indian Ocean; country: Maldives; locality: Huvadhu; minimumDepthInMeters: TBD; maximumDepthInMeters: TBD; locationRemarks: Nekton Maldives Mission; **Identification:** identifiedBy: Farah Amjad, Paris Stefanoudis, Mariyam Shidha Afzal, Hana Amir; dateIdentified: 2022, 2023; identificationRemarks: Identified only from imagery; **Event:** samplingProtocol: Submersible OR Remotely Operated Vehicle OR Snorkel; **Record Level:** basisOfRecord: Human observation

### Diploastrea heliopora

Matthai, 1914

E0CAC469-DC10-52EF-980C-E1431E4179F9

#### Materials

- **Type status:** Other material. **Occurrence:** occurrenceID: 0B4AA070-88E8-58B6-BBCF-EC9C1E66B304; **Taxon:** scientificName: *Diploastreaheliopora*; kingdom: Animalia; phylum: Cnidaria; class: Anthozoa; order: Scleractinia; family: Diploastraeidae; genus: Diploastrea; scientificNameAuthorship: Matthai, 1914; **Location:** waterBody: Indian Ocean; country: Maldives; locality: Laamu, Huvadhu, Vaavu, North Male’; minimumDepthInMeters: 2; maximumDepthInMeters: 30; locationRemarks: Nekton Maldives Mission; **Identification:** identifiedBy: Farah Amjad, Paris Stefanoudis, Mariyam Shidha Afzal, Hana Amir; dateIdentified: 2022, 2023; identificationRemarks: Identified only from imagery; **Event:** samplingProtocol: Submersible OR Remotely Operated Vehicle OR Snorkel; **Record Level:** basisOfRecord: Human observation

### Galaxea sp. indet.

7E74EAF6-BBB7-568C-A413-376AB2980778

#### Materials

- **Type status:** Other material. **Taxon:** scientificName: *Galaxea* sp.; kingdom: Animalia; phylum: Cnidaria; class: Anthozoa-Hexacorallia; order: Scleractinia; family: Euphyllidae; genus: Galaxea; **Location:** waterBody: Indian Ocean; country: Maldives; locality: North Male’, Vaavu, Huvadhu; minimumDepthInMeters: 2; maximumDepthInMeters: 30; locationRemarks: Nekton Maldives Mission; **Identification:** identifiedBy: Farah Amjad, Paris Stefanoudis, Mariyam Shidha Afzal, Hana Amir; dateIdentified: 2022, 2023; identificationRemarks: Identified only from imagery; **Event:** samplingProtocol: Submersible OR Remotely Operated Vehicle OR Snorkel; **Record Level:** basisOfRecord: Human observation

### Dipsastraea sp. indet.

3FA44724-2C74-5B79-A875-33FB7B00246D

#### Materials

- **Type status:** Other material. **Taxon:** scientificName: *Dipsastraea* sp.; kingdom: Animalia; phylum: Cnidaria; class: Anthozoa-Hexacorallia; order: Scleractinia; family: Faviidae; genus: Dipsastraea; **Location:** waterBody: Indian Ocean; country: Maldives; locality: North Male’, Vaavu, Laamu, Huvadhu, Addu, Fuvahmulah; minimumDepthInMeters: 2; maximumDepthInMeters: 30; locationRemarks: Nekton Maldives Mission; **Identification:** identifiedBy: Farah Amjad, Paris Stefanoudis, Mariyam Shidha Afzal, Hana Amir; dateIdentified: 2022, 2023; identificationRemarks: Identified only from imagery; **Event:** samplingProtocol: Submersible OR Remotely Operated Vehicle OR Snorkel; **Record Level:** basisOfRecord: Human observation

### Heliofungia actiniformis

(Quoy & Gaimard, 1833)

8A42B620-E06A-5296-808A-9955BD978E61

#### Materials

- **Type status:** Other material. **Occurrence:** occurrenceID: E5D74ABD-DDE5-598F-A0B6-28C02670828A; **Taxon:** scientificName: *Heliofungiaactiniformis*; kingdom: Animalia; phylum: Cnidaria; class: Anthozoa-Hexacorallia; order: Scleractinia; family: Fungiidae; genus: Heliofungia; scientificNameAuthorship: (Quoy & Gaimard, 1833); **Location:** waterBody: Indian Ocean; country: Maldives; locality: Laamu; minimumDepthInMeters: 30; maximumDepthInMeters: 30; locationRemarks: Nekton Maldives Mission; **Identification:** identifiedBy: Farah Amjad, Paris Stefanoudis, Mariyam Shidha Afzal, Hana Amir; dateIdentified: 2022, 2023; identificationRemarks: Identified only from imagery; **Event:** samplingProtocol: Submersible OR Remotely Operated Vehicle OR Snorkel; **Record Level:** basisOfRecord: Human observation

#### Notes

**Figure 79.**
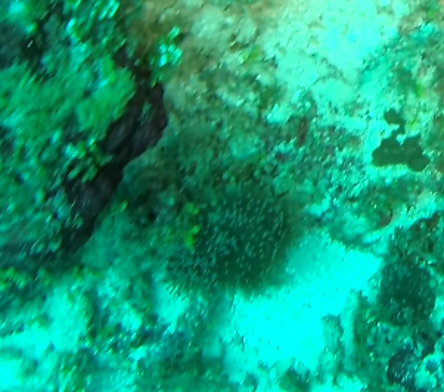
*Heliofungiaactiniformis*, Laamu, 30 m.

Solitary, free living corals. Juvenile colonies may remain attached to the substrate. Septa are granulate and continue to the underside of the corallum as fine ridges known as costae. Polyp is thick and fleshy and has a single mouth surrounded by thick tentacles with knobs on the end. Colony size ~ 15 cm in the longest dimension. Resembles a large anemone as it extends its tentacles during the daytime. Officially, only one species of *Heliofungia* has been recorded in Maldivian waters (Fig. 79).

### Fungiidae gen. indet. sp. 1

6B5432CB-FF4F-536B-9894-0C564F553AF6

#### Materials

- **Type status:** Other material. **Taxon:** scientificName: Fungiidae sp. 1; kingdom: Animalia; phylum: Cnidaria; class: Anthozoa-Hexacorallia; order: Scleractinia; family: Fungiidae; **Location:** waterBody: Indian Ocean; country: Maldives; locality: North Male’, Vaavu, Laamu, Huvadhu, Addu; minimumDepthInMeters: 2; maximumDepthInMeters: 30; locationRemarks: Nekton Maldives Mission; **Identification:** identifiedBy: Farah Amjad, Paris Stefanoudis, Mariyam Shidha Afzal, Hana Amir; dateIdentified: 2022, 2023; identificationRemarks: Identified only from imagery; **Event:** samplingProtocol: Submersible OR Remotely Operated Vehicle OR Snorkel; **Record Level:** basisOfRecord: Human observation

### Herpolitha sp. indet.

3EA032F0-2854-5F61-A9E0-7BD6C64C55C0

#### Materials

- **Type status:** Other material. **Taxon:** scientificName: *Herpolitha* sp.; kingdom: Animalia; phylum: Cnidaria; class: Anthozoa-Hexacorallia; order: Scleractinia; family: Fungiidae; genus: Herpolitha; **Location:** waterBody: Indian Ocean; country: Maldives; locality: Vaavu, Laamu, Addu; minimumDepthInMeters: 8; maximumDepthInMeters: 30; locationRemarks: Nekton Maldives Mission; **Identification:** identifiedBy: Farah Amjad, Paris Stefanoudis, Mariyam Shidha Afzal, Hana Amir; dateIdentified: 2022, 2023; identificationRemarks: Identified only from imagery; **Event:** samplingProtocol: Submersible OR Remotely Operated Vehicle OR Snorkel; **Record Level:** basisOfRecord: Human observation

### Halomitra sp. indet.

E7B20F54-F3EC-5D84-966C-8ED957752B65

#### Materials

- **Type status:** Other material. **Taxon:** scientificName: *Halomitra* sp.; kingdom: Animalia; phylum: Cnidaria; class: Anthozoa-Hexacorallia; order: Scleractinia; family: Fungiidae; genus: Halomitra; **Location:** waterBody: Indian Ocean; country: Maldives; locality: North Male’, Laamu, Huvadhu, Addu; minimumDepthInMeters: 10; maximumDepthInMeters: 30; locationRemarks: Nekton Maldives Mission; **Identification:** identifiedBy: Farah Amjad, Paris Stefanoudis, Mariyam Shidha Afzal, Hana Amir; dateIdentified: 2022, 2023; identificationRemarks: Identified only from imagery; **Event:** samplingProtocol: Submersible OR Remotely Operated Vehicle OR Snorkel; **Record Level:** basisOfRecord: Human observation

### Lithophyllon undulatum

Rehberg, 1892

1AE0CEEB-8BF2-5FC6-ADAD-DA4730EA9A97

#### Materials

- **Type status:** Other material. **Taxon:** scientificName: *Lithophyllonundulatum*; kingdom: Animalia; phylum: Cnidaria; class: Anthozoa-Hexacorallia; order: Scleractinia; family: Fungiidae; genus: Lithophyllon; scientificNameAuthorship: Rehberg, 1892; **Location:** waterBody: Indian Ocean; country: Maldives; locality: Vaavu; minimumDepthInMeters: 65; maximumDepthInMeters: 65; locationRemarks: Nekton Maldives Mission; **Identification:** identifiedBy: Farah Amjad, Paris Stefanoudis, Mariyam Shidha Afzal, Hana Amir; dateIdentified: 2022, 2023; identificationRemarks: Identified only from imagery; **Event:** samplingProtocol: Submersible OR Remotely Operated Vehicle OR Snorkel; **Record Level:** basisOfRecord: Human observation

#### Notes

Encrusting colonies which form a flat lamina with lobed margins. Size ~ 10 cm in the longest dimension. Brownish-red in colour with protruding septo-costae (Fig. 83).

### Pachyseris rugosa

(Lamarck, 1801)

A75FE459-1ED3-5551-9977-7C998276F9E3

#### Materials

- **Type status:** Other material. **Taxon:** scientificName: *Pachyserisrugosa*; kingdom: Animalia; phylum: Cnidaria; class: Anthozoa-Hexacorallia; order: Scleractinia; family: Incerta saedis; genus: Pachyseris; scientificNameAuthorship: (Lamarck, 1801); **Location:** waterBody: Indian Ocean; country: Maldives; locality: North Male’, Vaavu, Laamu, Huvadhu; minimumDepthInMeters: 8; maximumDepthInMeters: 30; locationRemarks: Nekton Maldives Mission; **Identification:** identifiedBy: Farah Amjad, Paris Stefanoudis, Mariyam Shidha Afzal, Hana Amir; dateIdentified: 2022, 2023; identificationRemarks: Identified only from imagery; **Event:** samplingProtocol: Submersible OR Remotely Operated Vehicle OR Snorkel; **Record Level:** basisOfRecord: Human observation

#### Notes

Colonies encrusting, with surface covered in a series of concentric ridges that are either parallel to colony margins or contorted. Colony size ~ 14 cm in the longest dimension (Fig. 84).

### Pachyseris speciosa

(Dana, 1846)

79755B3D-EBB9-54F9-8C8E-281C380CD89F

#### Materials

- **Type status:** Other material. **Occurrence:** occurrenceID: 3E30EDD5-32FA-55B9-8449-638953ADC254; **Taxon:** scientificName: *Pachyserisspeciosa*; kingdom: Animalia; phylum: Cnidaria; class: Anthozoa-Hexacorallia; order: Scleractinia; family: Incerta saedis; genus: Pachyseris; scientificNameAuthorship: (Dana, 1846); **Location:** waterBody: Indian Ocean; country: Maldives; locality: Vaavu, Laamu, Huvadhu, Addu; minimumDepthInMeters: 8; maximumDepthInMeters: 30; locationRemarks: Nekton Maldives Mission; **Identification:** identifiedBy: Farah Amjad, Paris Stefanoudis, Mariyam Shidha Afzal, Hana Amir; dateIdentified: 2022, 2023; identificationRemarks: Identified only from imagery; **Event:** samplingProtocol: Submersible OR Remotely Operated Vehicle OR Snorkel; **Record Level:** basisOfRecord: Human observation

#### Notes

Colony laminar and unifacial. Surface shows a more ridged appearance with corallite in between ridges. Colony size ~ 25 cm in the longest dimension. Darker brown with paler margins (Fig. 85).

### Physogyra lichtensteini

(Milne Edwards & Haime, 1851)

A6E4EFDB-4C88-587E-B6A2-B3E168F46945

#### Materials

- **Type status:** Other material. **Taxon:** scientificName: *Physogyralichtensteini*; kingdom: Animalia; phylum: Cnidaria; class: Anthozoa-Hexacorallia; order: Scleractinia; family: Incerta saedis; genus: Physogyra; scientificNameAuthorship: (Milne Edwards & Haime, 1851); **Location:** waterBody: Indian Ocean; country: Maldives; locality: Addu; minimumDepthInMeters: 10; maximumDepthInMeters: 30; locationRemarks: Nekton Maldives Mission; **Identification:** identifiedBy: Farah Amjad, Paris Stefanoudis, Mariyam Shidha Afzal, Hana Amir; dateIdentified: 2022, 2023; identificationRemarks: Identified only from imagery; **Event:** samplingProtocol: Submersible OR Remotely Operated Vehicle OR Snorkel; **Record Level:** basisOfRecord: Human observation

#### Notes

Colonies are massive or thickly encrusting. Colony surface is entirely covered in bubble-like, teardrop-shaped vesicles giving a fuzzy appearance. Vesicles paler white than the rest of the colony. When vesicles are constricted, shared walls of corallites that form valleys can be observed. Colony size ~ 20 cm in the longest dimension (Fig. 86).

### Plerogyra sinuosa

(Dana, 1846)

14E3F3D1-2D53-5D71-889C-855A60FF133D

#### Materials

- **Type status:** Other material. **Taxon:** scientificName: *Plerogyrasinuosa*; kingdom: Animalia; phylum: Cnidaria; class: Anthozoa-Hexacorallia; order: Scleractinia; family: Incerta saedis; genus: Plerogyra; scientificNameAuthorship: (Dana, 1846); **Location:** waterBody: Indian Ocean; country: Maldives; locality: Addu; minimumDepthInMeters: 26; maximumDepthInMeters: 30; locationRemarks: Nekton Maldives Mission; **Identification:** identifiedBy: Farah Amjad, Paris Stefanoudis, Mariyam Shidha Afzal, Hana Amir; dateIdentified: 2022, 2023; identificationRemarks: Identified only from imagery; **Event:** samplingProtocol: Submersible OR Remotely Operated Vehicle OR Snorkel; **Record Level:** basisOfRecord: Human observation

#### Notes

**Figure 87.**
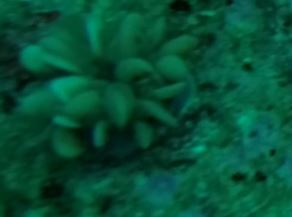
*Plerogyrasinuosa*, Addu, 30 m.

Colonies are flabello-meandroid with valleys thinly connected by a light blistery coenosteum. Has a bubbly appearance. Colony size ~ 17 cm in the longest dimension. Cream to bluish-grey in colour (Fig. 87).

### Plesiastrea versipora

(Lamarck, 1816)

76A95638-1D90-5677-ABE4-200E163708F1

#### Materials

- **Type status:** Other material. **Taxon:** scientificName: *Plesiastreaversipora*; kingdom: Animalia; phylum: Cnidaria; class: Anthozoa-Hexacorallia; order: Scleractinia; family: Incerta saedis; genus: Plesiastrea; scientificNameAuthorship: (Lamarck, 1816); **Location:** waterBody: Indian Ocean; country: Maldives; locality: North Male’, Vaavu, Laamu, Huvadhu; minimumDepthInMeters: 10; maximumDepthInMeters: 30; locationRemarks: Nekton Maldives Mission; **Identification:** identifiedBy: Farah Amjad, Paris Stefanoudis, Mariyam Shidha Afzal, Hana Amir; dateIdentified: 2022, 2023; identificationRemarks: Identified only from imagery; **Event:** samplingProtocol: Submersible OR Remotely Operated Vehicle OR Snorkel; **Record Level:** basisOfRecord: Human observation

#### Notes

Massive encrusting colonies. Multiple colour variants mostly beige to brown-greens. Paliform lobes form a neat circle around small columellae. Colony size ~ 11 cm in the longest dimension. Only this species of *Plesiastrea* has been officially recorded in Maldivian waters (Fig. 88).

### Echinophyllia sp. indet.

D837850B-36E3-5519-83E6-BF535A326998

#### Materials

- **Type status:** Other material. **Taxon:** scientificName: *Echinophyllia* sp.; kingdom: Animalia; phylum: Cnidaria; class: Anthozoa-Hexacorallia; order: Scleractinia; family: Lobophyllidae; genus: Echinophyllia; **Location:** waterBody: Indian Ocean; country: Maldives; locality: North Male’, Vaavu, Huvadhu, Addu; minimumDepthInMeters: 10; maximumDepthInMeters: 30; locationRemarks: Nekton Maldives Mission; **Identification:** identifiedBy: Farah Amjad, Paris Stefanoudis, Mariyam Shidha Afzal, Hana Amir; dateIdentified: 2022, 2023; identificationRemarks: Identified only from imagery; **Event:** samplingProtocol: Submersible OR Remotely Operated Vehicle OR Snorkel; **Record Level:** basisOfRecord: Human observation

### Lobophyllia sp. indet.

2462399E-897E-5AA0-BA11-464CE94EF1C7

#### Materials

- **Type status:** Other material. **Taxon:** scientificName: *Lobophyllia* sp.; kingdom: Animalia; phylum: Cnidaria; class: Anthozoa-Hexacorallia; order: Scleractinia; family: Lobophyllidae; genus: Lobophyllia; **Location:** waterBody: Indian Ocean; country: Maldives; locality: North Male’, Vaavu, Laamu, Huvadhu, Addu; minimumDepthInMeters: 2; maximumDepthInMeters: 30; locationRemarks: Nekton Maldives Mission; **Identification:** identifiedBy: Farah Amjad, Paris Stefanoudis, Mariyam Shidha Afzal, Hana Amir; dateIdentified: 2022, 2023; identificationRemarks: Identified only from imagery; **Event:** samplingProtocol: Submersible OR Remotely Operated Vehicle OR Snorkel; **Record Level:** basisOfRecord: Human observation

### Oxypora crassispinosa

Nemenzo, 1979

A14376B7-23D3-5D86-8617-C2E36A3765A5

#### Materials

- **Type status:** Other material. **Taxon:** scientificName: *Oxyporacrassispinosa*; kingdom: Animalia; phylum: Cnidaria; class: Anthozoa-Hexacorallia; order: Scleractinia; family: Lobophyllidae; genus: Oxypora; scientificNameAuthorship: Nemenzo, 1979; **Location:** waterBody: Indian Ocean; country: Maldives; locality: Vaavu, Addu; minimumDepthInMeters: 26; maximumDepthInMeters: 30; locationRemarks: Nekton Maldives Mission; **Identification:** identifiedBy: Farah Amjad, Paris Stefanoudis, Mariyam Shidha Afzal, Hana Amir; dateIdentified: 2022, 2023; identificationRemarks: Identified only from imagery; **Event:** samplingProtocol: Submersible OR Remotely Operated Vehicle OR Snorkel; **Record Level:** basisOfRecord: Human observation

#### Notes

Laminar colonies, forming thin plates, sometimes forming tiers. Colony size ~ 32 cm in the longest dimension. With thin-walled, meandering valleys, some branch towards the centre. Colour from light brown to grey or green (Fig. 91).

### Coelastrea sp. indet.

4F8E294E-CAEE-5A27-8DF3-0AE09492A2C6

#### Materials

- **Type status:** Other material. **Taxon:** scientificName: *Coelastrea* sp.; kingdom: Animalia; phylum: Cnidaria; class: Anthozoa-Hexacorallia; order: Scleractinia; family: Merulinidae; genus: Coelastrea; **Location:** waterBody: Indian Ocean; country: Maldives; locality: North Male’, Huvadhu, Addu, Fuvahmulah; minimumDepthInMeters: 2; maximumDepthInMeters: 10; locationRemarks: Nekton Maldives Mission; **Identification:** identifiedBy: Farah Amjad, Paris Stefanoudis, Mariyam Shidha Afzal, Hana Amir; dateIdentified: 2022, 2023; identificationRemarks: Identified only from imagery; **Event:** samplingProtocol: Submersible OR Remotely Operated Vehicle OR Snorkel; **Record Level:** basisOfRecord: Human observation

### Echinopora sp. indet.

DAEBBE4C-1E59-5C54-9377-89F8C6F1DCFC

#### Materials

- **Type status:** Other material. **Taxon:** scientificName: *Echinopora* sp.; kingdom: Animalia; phylum: Cnidaria; class: Anthozoa-Hexacorallia; order: Scleractinia; family: Merulinidae; genus: Echinopora; **Location:** waterBody: Indian Ocean; country: Maldives; locality: North Male’, Vaavu, Laamu, Huvadhu, Addu; minimumDepthInMeters: 10; maximumDepthInMeters: 62; locationRemarks: Nekton Maldives Mission; **Identification:** identifiedBy: Farah Amjad, Paris Stefanoudis, Mariyam Shidha Afzal, Hana Amir; dateIdentified: 2022, 2023; identificationRemarks: Identified only from imagery; **Event:** samplingProtocol: Submersible OR Remotely Operated Vehicle OR Snorkel; **Record Level:** basisOfRecord: Human observation

### Favites sp. indet.

8D8D9DCF-07C6-516F-99E5-70428F8D5566

#### Materials

- **Type status:** Other material. **Taxon:** scientificName: *Favites* sp.; kingdom: Animalia; phylum: Cnidaria; class: Anthozoa-Hexacorallia; order: Scleractinia; family: Merulinidae; genus: Favites; **Location:** waterBody: Indian Ocean; country: Maldives; locality: North Male’, Vaavu, Laamu, Huvadhu, Addu; minimumDepthInMeters: 2; maximumDepthInMeters: 30; locationRemarks: Nekton Maldives Mission; **Identification:** identifiedBy: Farah Amjad, Paris Stefanoudis, Mariyam Shidha Afzal, Hana Amir; dateIdentified: 2022, 2023; identificationRemarks: Identified only from imagery; **Event:** samplingProtocol: Submersible OR Remotely Operated Vehicle OR Snorkel; **Record Level:** basisOfRecord: Human observation

### Goniastrea sp. indet.

842DA3BE-8D88-5F1E-B86C-ED0D707B070E

#### Materials

- **Type status:** Other material. **Taxon:** scientificName: *Goniastrea* sp.; kingdom: Animalia; phylum: Cnidaria; class: Anthozoa-Hexacorallia; order: Scleractinia; family: Merulinidae; genus: Goniastrea; **Location:** waterBody: Indian Ocean; country: Maldives; locality: North Male’, Vaavu, Laamu, Huvadhu, Addu; minimumDepthInMeters: 2; maximumDepthInMeters: 30; locationRemarks: Nekton Maldives Mission; **Identification:** identifiedBy: Farah Amjad, Paris Stefanoudis, Mariyam Shidha Afzal, Hana Amir; dateIdentified: 2022, 2023; identificationRemarks: Identified only from imagery; **Event:** samplingProtocol: Submersible OR Remotely Operated Vehicle OR Snorkel; **Record Level:** basisOfRecord: Human observation

### Hydnophora sp. indet.

33562320-59C5-5C20-AFF8-C64FA4088AE8

#### Materials

- **Type status:** Other material. **Taxon:** scientificName: *Hydnophora* sp.; kingdom: Animalia; phylum: Cnidaria; class: Anthozoa-Hexacorallia; order: Scleractinia; family: Merulinidae; genus: Hydnophora; **Location:** waterBody: Indian Ocean; country: Maldives; locality: North Male’, Vaavu; minimumDepthInMeters: 10; maximumDepthInMeters: 30; locationRemarks: Nekton Maldives Mission; **Identification:** identifiedBy: Farah Amjad, Paris Stefanoudis, Mariyam Shidha Afzal, Hana Amir; dateIdentified: 2022, 2023; identificationRemarks: Identified only from imagery; **Event:** samplingProtocol: Submersible OR Remotely Operated Vehicle OR Snorkel; **Record Level:** basisOfRecord: Human observation

### Leptoria sp. indet.

DD69F112-7710-59D0-AE27-5F2703770E23

#### Materials

- **Type status:** Other material. **Taxon:** scientificName: *Leptoria* sp.; kingdom: Animalia; phylum: Cnidaria; class: Anthozoa-Hexacorallia; order: Scleractinia; family: Merulinidae; genus: Leptoria; **Location:** waterBody: Indian Ocean; country: Maldives; locality: North Male’, Vaavu, Laamu; minimumDepthInMeters: 2; maximumDepthInMeters: 10; locationRemarks: Nekton Maldives Mission; **Identification:** identifiedBy: Farah Amjad, Paris Stefanoudis, Mariyam Shidha Afzal, Hana Amir; dateIdentified: 2022, 2023; identificationRemarks: Identified only from imagery; **Event:** samplingProtocol: Submersible OR Remotely Operated Vehicle OR Snorkel; **Record Level:** basisOfRecord: Human observation

### Merulina sp. indet.

06F6FFC2-5DD6-5ACE-8C04-9FC9C65A88BA

#### Materials

- **Type status:** Other material. **Taxon:** scientificName: *Merulina* sp.; kingdom: Animalia; phylum: Cnidaria; class: Anthozoa-Hexacorallia; order: Scleractinia; family: Merulinidae; genus: Merulina; **Location:** waterBody: Indian Ocean; country: Maldives; locality: Vaavu, Addu; minimumDepthInMeters: 10; maximumDepthInMeters: 30; locationRemarks: Nekton Maldives Mission; **Identification:** identifiedBy: Farah Amjad, Paris Stefanoudis, Mariyam Shidha Afzal, Hana Amir; dateIdentified: 2022, 2023; identificationRemarks: Identified only from imagery; **Event:** samplingProtocol: Submersible OR Remotely Operated Vehicle OR Snorkel; **Record Level:** basisOfRecord: Human observation

### Mycedium sp. indet.

D3DDC9B6-6E8F-5999-851B-14AA00469B1D

#### Materials

- **Type status:** Other material. **Taxon:** scientificName: *Mycedium* sp.; kingdom: Animalia; phylum: Cnidaria; class: Anthozoa-Hexacorallia; order: Scleractinia; family: Merulinidae; genus: Mycedium; **Location:** waterBody: Indian Ocean; country: Maldives; locality: North Male’, Vaavu, Laamu, Huvadhu, Addu; minimumDepthInMeters: 10; maximumDepthInMeters: 30; locationRemarks: Nekton Maldives Mission; **Identification:** identifiedBy: Farah Amjad, Paris Stefanoudis, Mariyam Shidha Afzal, Hana Amir; dateIdentified: 2022, 2023; identificationRemarks: Identified only from imagery; **Event:** samplingProtocol: Submersible OR Remotely Operated Vehicle OR Snorkel; **Record Level:** basisOfRecord: Human observation

### Oulophyllia sp. indet.

DFC27D63-AAD2-53B6-94CF-8DA8E3F154E4

#### Materials

- **Type status:** Other material. **Taxon:** scientificName: *Oulophyllia* sp.; kingdom: Animalia; phylum: Cnidaria; class: Anthozoa-Hexacorallia; order: Scleractinia; family: Merulinidae; genus: Oulophyllia; **Location:** waterBody: Indian Ocean; country: Maldives; locality: Laamu; minimumDepthInMeters: 8; maximumDepthInMeters: 10; locationRemarks: Nekton Maldives Mission; **Identification:** identifiedBy: Farah Amjad, Paris Stefanoudis, Mariyam Shidha Afzal, Hana Amir; dateIdentified: 2022, 2023; identificationRemarks: Identified only from imagery; **Event:** samplingProtocol: Submersible OR Remotely Operated Vehicle OR Snorkel; **Record Level:** basisOfRecord: Human observation

### Paragoniastrea russelli

(Wells, 1954)

F0F44443-C7FB-5B5B-B32F-B1BA7C2372F6

#### Materials

- **Type status:** Other material. **Taxon:** scientificName: *Paragoniastrearusselli*; kingdom: Animalia; phylum: Cnidaria; class: Anthozoa-Hexacorallia; order: Scleractinia; family: Merulinidae; genus: Paragoniastrea; scientificNameAuthorship: (Wells, 1954); **Location:** waterBody: Indian Ocean; country: Maldives; locality: Laamu; minimumDepthInMeters: 30; maximumDepthInMeters: 30; locationRemarks: Nekton Maldives Mission; **Identification:** identifiedBy: Farah Amjad, Paris Stefanoudis, Mariyam Shidha Afzal, Hana Amir; dateIdentified: 2022, 2023; identificationRemarks: Identified only from imagery; **Event:** samplingProtocol: Submersible OR Remotely Operated Vehicle OR Snorkel; **Record Level:** basisOfRecord: Human observation

#### Notes

Thick crusting massive colonies with deep meandering valleys. Previously grouped with *Goniastrea* and exhibits long valleys that appear with more regularly-shaped septa than *Platygyra* (Fig. 101).

### Platygyra sp. indet.

54B03EE3-0ECD-5276-81B1-F62958509D86

#### Materials

- **Type status:** Other material. **Taxon:** scientificName: *Platygyra* sp.; kingdom: Animalia; phylum: Cnidaria; class: Anthozoa-Hexacorallia; order: Scleractinia; family: Merulinidae; genus: Platygyra; **Location:** waterBody: Indian Ocean; country: Maldives; locality: North Male’, Vaavu, Laamu, Huvadhu, Addu; minimumDepthInMeters: 2; maximumDepthInMeters: 30; locationRemarks: Nekton Maldives Mission; **Identification:** identifiedBy: Farah Amjad, Paris Stefanoudis, Mariyam Shidha Afzal, Hana Amir; dateIdentified: 2022, 2023; identificationRemarks: Identified only from imagery; **Event:** samplingProtocol: Submersible OR Remotely Operated Vehicle OR Snorkel; **Record Level:** basisOfRecord: Human observation

### Madracis sp. indet.

7B97C920-048A-5F1E-B0F0-D51FA581AF8B

#### Materials

- **Type status:** Other material. **Taxon:** scientificName: *Madracis* sp.; kingdom: Animalia; phylum: Cnidaria; class: Anthozoa-Hexacorallia; order: Scleractinia; family: Pocilloporidae; genus: Madracis; **Location:** waterBody: Indian Ocean; country: Maldives; locality: Laamu, Huvadhu, Addu, Fuvahmulah; minimumDepthInMeters: 57; maximumDepthInMeters: 120; locationRemarks: Nekton Maldives Mission; **Identification:** identifiedBy: Farah Amjad, Paris Stefanoudis, Mariyam Shidha Afzal, Hana Amir; dateIdentified: 2022, 2023; identificationRemarks: Identified only from imagery; **Event:** samplingProtocol: Submersible OR Remotely Operated Vehicle OR Snorkel; **Record Level:** basisOfRecord: Human observation

### Pocillopora sp. indet. 1

11C2F5B5-2119-5D58-B7A6-1D0A277443A1

#### Materials

- **Type status:** Other material. **Taxon:** scientificName: *Pocillopora* sp. 1; kingdom: Animalia; phylum: Cnidaria; class: Anthozoa-Hexacorallia; order: Scleractinia; family: Pocilloporidae; genus: Pocillopora; **Location:** waterBody: Indian Ocean; country: Maldives; locality: Vaavu, Laamu, Huvadhu, Addu; minimumDepthInMeters: 2.5; maximumDepthInMeters: 30; locationRemarks: Nekton Maldives Mission; **Identification:** identifiedBy: Farah Amjad, Paris Stefanoudis, Mariyam Shidha Afzal, Hana Amir; dateIdentified: 2022, 2023; identificationRemarks: Identified only from imagery; **Event:** samplingProtocol: Submersible OR Remotely Operated Vehicle OR Snorkel; **Record Level:** basisOfRecord: Human observation

### Pocillopora sp. indet. 2

BD7CB66C-18A9-5B88-90B2-2C933D381EA7

#### Materials

- **Type status:** Other material. **Taxon:** scientificName: Pocillopora sp. 2; kingdom: Animalia; phylum: Cnidaria; class: Anthozoa-Hexacorallia; order: Scleractinia; family: Pocilloporidae; genus: Pocillopora; **Location:** waterBody: Indian Ocean; country: Maldives; locality: Vaavu, Laamu; minimumDepthInMeters: 10; maximumDepthInMeters: 10; locationRemarks: Nekton Maldives Mission; **Identification:** identifiedBy: Farah Amjad, Paris Stefanoudis, Mariyam Shidha Afzal, Hana Amir; dateIdentified: 2022, 2023; identificationRemarks: Identified only from imagery; **Event:** samplingProtocol: Submersible OR Remotely Operated Vehicle OR Snorkel; **Record Level:** basisOfRecord: Human observation

### Pocillopora sp. indet. 3

98B62F91-F736-5465-8C0F-7268EADA639A

#### Materials

- **Type status:** Other material. **Taxon:** scientificName: *Pocillopora* sp. 3; kingdom: Animalia; phylum: Cnidaria; class: Anthozoa-Hexacorallia; order: Scleractinia; family: Pocilloporidae; genus: Pocillopora; **Location:** waterBody: Indian Ocean; country: Maldives; locality: Vaavu, Laamu, Huvadhu; minimumDepthInMeters: 8; maximumDepthInMeters: 30; locationRemarks: Nekton Maldives Mission; **Identification:** identifiedBy: Farah Amjad, Paris Stefanoudis, Mariyam Shidha Afzal, Hana Amir; dateIdentified: 2022, 2023; identificationRemarks: Identified only from imagery; **Event:** samplingProtocol: Submersible OR Remotely Operated Vehicle OR Snorkel; **Record Level:** basisOfRecord: Human observation

### Pocillopora sp. indet. 4

0ACB871D-99E7-5D12-B718-6CF17C8276BE

#### Materials

- **Type status:** Other material. **Taxon:** scientificName: *Pocillopora* sp. 4; kingdom: Animalia; phylum: Cnidaria; class: Anthozoa-Hexacorallia; order: Scleractinia; family: Pocilloporidae; genus: Pocillopora; **Location:** waterBody: Indian Ocean; country: Maldives; locality: Laamu, Huvadhu; minimumDepthInMeters: 2; maximumDepthInMeters: 10; locationRemarks: Nekton Maldives Mission; **Identification:** identifiedBy: Farah Amjad, Paris Stefanoudis, Mariyam Shidha Afzal, Hana Amir; dateIdentified: 2022, 2023; identificationRemarks: Identified only from imagery; **Event:** samplingProtocol: Submersible OR Remotely Operated Vehicle OR Snorkel; **Record Level:** basisOfRecord: Human observation

### Goniopora sp. indet.

7FDD918A-178A-56B3-A491-36F41E37FFA6

#### Materials

- **Type status:** Other material. **Taxon:** scientificName: *Goniopora* sp.; kingdom: Animalia; phylum: Cnidaria; class: Anthozoa-Hexacorallia; order: Scleractinia; family: Poritidae; genus: Goniopora; **Location:** waterBody: Indian Ocean; country: Maldives; locality: North Male’, Vaavu, Laamu, Huvadhu, Addu, Fuvahmulah; minimumDepthInMeters: 10; maximumDepthInMeters: 60; locationRemarks: Nekton Maldives Mission; **Identification:** identifiedBy: Farah Amjad, Paris Stefanoudis, Mariyam Shidha Afzal, Hana Amir; dateIdentified: 2022, 2023; identificationRemarks: Identified only from imagery; **Event:** samplingProtocol: Submersible OR Remotely Operated Vehicle OR Snorkel; **Record Level:** basisOfRecord: Human observation

### Porites rus

117CAB09-13F8-5557-98EF-4A47655860FE

#### Materials

- **Type status:** Other material. **Taxon:** scientificName: *Poritesrus*; kingdom: Animalia; phylum: Cnidaria; class: Anthozoa-Hexacorallia; order: Scleractinia; family: Poritidae; genus: Porites; scientificNameAuthorship: (Forskål, 1775); **Location:** waterBody: Indian Ocean; country: Maldives; locality: North Male’, Vaavu, Laamu, Huvadhu, Addu, Fuvahmulah; minimumDepthInMeters: 2; maximumDepthInMeters: 30; locationRemarks: Nekton Maldives Mission; **Identification:** identifiedBy: Farah Amjad, Paris Stefanoudis, Mariyam Shidha Afzal, Hana Amir; dateIdentified: 2022, 2023; identificationRemarks: Identified only from imagery; **Event:** samplingProtocol: Submersible OR Remotely Operated Vehicle OR Snorkel; **Record Level:** basisOfRecord: Human observation

#### Notes

Individual colonies can take on multiple morphologies shifting from encrusting, plating to branching. Branching portions of the colonies form short, stubby, contorted branches. Corallites are small and frequently hidden within the structure of the colony. Colony size ~ 29 cm in the longest dimension. Pale cream and yellowish-brown with tips of branches appearing more white (Fig. 109).

### Porites sp. indet. 1

CC2772F7-2441-51A3-B5D5-C49F0C40E3F8

#### Materials

- **Type status:** Other material. **Taxon:** scientificName: *Porites* sp. 1; kingdom: Animalia; phylum: Cnidaria; class: Anthozoa-Hexacorallia; order: Scleractinia; family: Poritidae; genus: Porites; **Location:** waterBody: Indian Ocean; country: Maldives; locality: North Male’, Vaavu, Laamu, Huvadhu, Addu, Fuvahmulah; minimumDepthInMeters: 2; maximumDepthInMeters: 55; locationRemarks: Nekton Maldives Mission; **Identification:** identifiedBy: Farah Amjad, Paris Stefanoudis, Mariyam Shidha Afzal, Hana Amir; dateIdentified: 2022, 2023; identificationRemarks: Identified only from imagery; **Event:** samplingProtocol: Submersible OR Remotely Operated Vehicle OR Snorkel; **Record Level:** basisOfRecord: Human observation

### Porites sp. indet. 2

48CB3166-364D-5C9F-BBB8-45632E9E0477

#### Materials

- **Type status:** Other material. **Taxon:** scientificName: *Porites* sp. 2; kingdom: Animalia; phylum: Cnidaria; class: Anthozoa-Hexacorallia; order: Scleractinia; family: Poritidae; genus: Porites; **Location:** waterBody: Indian Ocean; country: Maldives; locality: North Male’, Vaavu, Laamu, Huvadhu, Addu; minimumDepthInMeters: 2; maximumDepthInMeters: 30; locationRemarks: Nekton Maldives Mission; **Identification:** identifiedBy: Farah Amjad, Paris Stefanoudis, Mariyam Shidha Afzal, Hana Amir; dateIdentified: 2022, 2023; identificationRemarks: Identified only from imagery; **Event:** samplingProtocol: Submersible OR Remotely Operated Vehicle OR Snorkel; **Record Level:** basisOfRecord: Human observation

### Porites sp. indet. 3

7E2DD6A0-F478-5C8F-821D-9FD9B0BCC386

#### Materials

- **Type status:** Other material. **Taxon:** scientificName: *Porites* sp. 3; kingdom: Animalia; phylum: Cnidaria; class: Anthozoa-Hexacorallia; order: Scleractinia; family: Poritidae; genus: Porites; **Location:** waterBody: Indian Ocean; country: Maldives; locality: Vaavu, Huvadhu, Addu; minimumDepthInMeters: 26; maximumDepthInMeters: 30; locationRemarks: Nekton Maldives Mission; **Identification:** identifiedBy: Farah Amjad, Paris Stefanoudis, Mariyam Shidha Afzal, Hana Amir; dateIdentified: 2022, 2023; identificationRemarks: Identified only from imagery; **Event:** samplingProtocol: Submersible OR Remotely Operated Vehicle OR Snorkel; **Record Level:** basisOfRecord: Human observation

### Psammocora sp. indet.

201F3DAC-1692-5C35-ACC6-22694B594F60

#### Materials

- **Type status:** Other material. **Taxon:** scientificName: *Psammocora* sp.; kingdom: Animalia; phylum: Cnidaria; class: Anthozoa-Hexacorallia; order: Scleractinia; family: Psammocoridae; genus: Psammocora; **Location:** waterBody: Indian Ocean; country: Maldives; locality: Huvadhu; minimumDepthInMeters: 10; maximumDepthInMeters: 30; locationRemarks: Nekton Maldives Mission; **Identification:** identifiedBy: Farah Amjad, Paris Stefanoudis, Mariyam Shidha Afzal, Hana Amir; dateIdentified: 2022, 2023; identificationRemarks: Identified only from imagery; **Event:** samplingProtocol: Submersible OR Remotely Operated Vehicle OR Snorkel; **Record Level:** basisOfRecord: Human observation

### Palythoa tuberculosa

(Esper, 1805)

139D1B79-7910-51FF-A41E-BE5FA80F94BA

#### Materials

- **Type status:** Other material. **Occurrence:** occurrenceID: 6F19890C-8840-5778-982A-3043C90B5A17; **Taxon:** scientificName: *Palythoatuberculosa*; kingdom: Animalia; phylum: Cnidaria; class: Anthozoa-Hexacorallia; order: Zoantharia; family: Sphenopidae; genus: Palythoa; scientificNameAuthorship: (Esper, 1805); **Location:** waterBody: Indian Ocean; country: Maldives; locality: North Male’, Vaavu, Laamu, Huvadhu, Addu; minimumDepthInMeters: 2; maximumDepthInMeters: 30; locationRemarks: Nekton Maldives Mission; **Identification:** identifiedBy: Farah Amjad, Paris Stefanoudis; dateIdentified: 2022, 2023; identificationRemarks: Identified only from imagery; **Event:** samplingProtocol: Submersible OR Remotely Operated Vehicle OR Snorkel; **Record Level:** basisOfRecord: Human observation

### Palythoa sp. indet. 2

E79C6EE8-836F-5BD5-A3CB-927412895110

#### Materials

- **Type status:** Other material. **Taxon:** scientificName: *Palythoa* sp. 2; kingdom: Animalia; phylum: Cnidaria; class: Anthozoa; order: Zoantharia; family: Sphenopidae; genus: Palythoa; **Location:** waterBody: Indian Ocean; country: Maldives; locality: Vaavu, Addu; minimumDepthInMeters: 10; maximumDepthInMeters: 30; locationRemarks: Nekton Maldives Mission; **Identification:** identifiedBy: Farah Amjad, Paris Stefanoudis; dateIdentified: 2022, 2023; identificationRemarks: Identified only from imagery; **Event:** samplingProtocol: Submersible OR Remotely Operated Vehicle OR Snorkel; **Record Level:** basisOfRecord: Human observation

### Arachnopathes sp. indet.

ED5F13E8-B5D0-5F99-A11D-5538153A83C9

#### Materials

- **Type status:** Other material. **Taxon:** scientificName: *Arachnopathes* sp.; kingdom: Animalia; phylum: Cnidaria; class: Anthozoa-Hexacorallia; order: Antipatharia; family: Antipathidae; genus: Arachnopathes; **Location:** waterBody: Indian Ocean; country: Maldives; locality: Vaavu, Huvadhu, Addu; minimumDepthInMeters: 30; maximumDepthInMeters: 60; locationRemarks: Nekton Maldives Mission; **Identification:** identifiedBy: Farah Amjad, Paris Stefanoudis, Erika Gress; dateIdentified: 2022, 2023; identificationRemarks: Identified only from imagery; **Event:** samplingProtocol: Submersible OR Remotely Operated Vehicle OR Snorkel; **Record Level:** basisOfRecord: Human observation

### Antipathes nilanduensis

Cooper, 1903

85C75771-2AD1-5E18-B2BB-93013097C963

#### Materials

- **Type status:** Other material. **Taxon:** scientificName: *Antipathesnilanduensis*; kingdom: Animalia; phylum: Cnidaria; class: Anthozoa-Hexacorallia; order: Antipatharia; family: Antipathidae; genus: Antipathes; scientificNameAuthorship: Cooper, 1903; **Location:** waterBody: Indian Ocean; country: Maldives; locality: North Male’; minimumDepthInMeters: 60; maximumDepthInMeters: 60; locationRemarks: Nekton Maldives Mission; **Identification:** identifiedBy: Farah Amjad, Paris Stefanoudis, Erika Gress; dateIdentified: 2022, 2023; identificationRemarks: Identified only from imagery; **Event:** samplingProtocol: Submersible OR Remotely Operated Vehicle OR Snorkel; **Record Level:** basisOfRecord: Human observation

#### Notes

Colonies are branching and mostly grow in a single pane with densely packed, fine branches. Colony height ~ 13 cm (Fig. 117).

### Antipathes sp. indet. 2

4F8FAF05-F055-5CC6-9EFA-40016CD41B32

#### Materials

- **Type status:** Other material. **Taxon:** scientificName: *Antipathes* sp. 2; kingdom: Animalia; phylum: Cnidaria; class: Anthozoa-Hexacorallia; order: Antipatharia; family: Antipathidae; genus: Antipathes; scientificNameAuthorship: Pallas, 1766; **Location:** waterBody: Indian Ocean; country: Maldives; locality: Vaavu, Laamu, Huvadhu, Addu; minimumDepthInMeters: 30; maximumDepthInMeters: 121; locationRemarks: Nekton Maldives Mission; **Identification:** identifiedBy: Farah Amjad, Paris Stefanoudis, Erika Gress; dateIdentified: 2022, 2023; identificationRemarks: Identified only from imagery; **Event:** samplingProtocol: Submersible OR Remotely Operated Vehicle OR Snorkel; **Record Level:** basisOfRecord: Human observation

### Antipathes sp. indet. 3

9401DC7C-283F-5087-A724-637B786ECCFD

#### Materials

- **Type status:** Other material. **Taxon:** scientificName: *Antipathes* sp. 3; kingdom: Animalia; phylum: Cnidaria; class: Anthozoa-Hexacorallia; order: Antipatharia; family: Antipathidae; genus: Antipathes; **Location:** waterBody: Indian Ocean; country: Maldives; locality: Addu; minimumDepthInMeters: 59; maximumDepthInMeters: 120; locationRemarks: Nekton Maldives Mission; **Identification:** identifiedBy: Farah Amjad, Paris Stefanoudis, Erika Gress; dateIdentified: 2022, 2023; identificationRemarks: Identified only from imagery; **Event:** samplingProtocol: Submersible OR Remotely Operated Vehicle OR Snorkel; **Record Level:** basisOfRecord: Human observation

### Antipathes sp. indet. 4

FED56C07-5F19-5595-9400-6B265FEF5FCB

#### Materials

- **Type status:** Other material. **Taxon:** scientificName: *Antipathes* sp. 4; kingdom: Animalia; phylum: Cnidaria; class: Anthozoa-Hexacorallia; order: Antipatharia; family: Antipathidae; genus: Antipathes; **Location:** waterBody: Indian Ocean; country: Maldives; locality: Vaavu, Huvadhu; minimumDepthInMeters: 63; maximumDepthInMeters: 121; locationRemarks: Nekton Maldives Mission; **Identification:** identifiedBy: Farah Amjad, Paris Stefanoudis, Erika Gress; dateIdentified: 2022, 2023; identificationRemarks: Identified only from imagery; **Event:** samplingProtocol: Submersible OR Remotely Operated Vehicle OR Snorkel; **Record Level:** basisOfRecord: Human observation

### Asteriopathes sp. indet.

84AB19D0-C877-5A6F-9189-567AA4BF8928

#### Materials

- **Type status:** Other material. **Taxon:** scientificName: *Asteriopathes* sp.; kingdom: Animalia; phylum: Cnidaria; class: Anthozoa-Hexacorallia; order: Antipatharia; family: Aphanipathidae; genus: Asteriopathes; **Location:** waterBody: Indian Ocean; country: Maldives; locality: Vaavu, Laamu, Huvadhu, Addu; minimumDepthInMeters: 116; maximumDepthInMeters: 121; locationRemarks: Nekton Maldives Mission; **Identification:** identifiedBy: Farah Amjad, Paris Stefanoudis, Erika Gress; dateIdentified: 2022, 2023; identificationRemarks: Identified only from imagery; **Event:** samplingProtocol: Submersible OR Remotely Operated Vehicle OR Snorkel; **Record Level:** basisOfRecord: Human observation

### Tetrapathes sp. indet.

727AD32A-1B41-596C-8281-C90F81F91CEC

#### Materials

- **Type status:** Other material. **Taxon:** scientificName: *Tetrapathes* sp.; kingdom: Animalia; phylum: Cnidaria; class: Anthozoa; order: Antipatharia; family: Aphanipathidae; genus: Tetrapathes; **Location:** waterBody: Indian Ocean; country: Maldives; locality: North Male’, Laamu, Huvadhu, Fuvahmulah, Addu; minimumDepthInMeters: 57; maximumDepthInMeters: 489; locationRemarks: Nekton Maldives Mission; **Identification:** identifiedBy: Farah Amjad, Paris Stefanoudis, Erika Gress; **Event:** samplingProtocol: Submersible OR Remotely Operated Vehicle OR Snorkel; **Record Level:** basisOfRecord: Human observation

### Bathypathes sp. indet.

D96E961D-2889-54C4-B266-8FBD8FF01343

#### Materials

- **Type status:** Other material. **Taxon:** scientificName: *Bathypathes* sp.; kingdom: Animalia; phylum: Cnidaria; class: Anthozoa-Hexacorallia; order: Antipatharia; family: Schizopathidae; genus: Bathypathes; **Location:** waterBody: Indian Ocean; country: Maldives; locality: Huvadhu, Fuvahmulah; minimumDepthInMeters: 249; maximumDepthInMeters: 253; locationRemarks: Nekton Maldives Mission; **Identification:** identifiedBy: Farah Amjad, Paris Stefanoudis, Erika Gress; dateIdentified: 2022, 2023; identificationRemarks: Identified only from imagery; **Event:** samplingProtocol: Submersible OR Remotely Operated Vehicle OR Snorkel; **Record Level:** basisOfRecord: Human observation

### Cupressopathes sp. indet.

8AB9A81D-7304-51DF-8C03-6ADC4165C9C6

#### Materials

- **Type status:** Other material. **Taxon:** scientificName: *Cupressopathes* sp.; kingdom: Animalia; phylum: Cnidaria; class: Anthozoa-Hexacorallia; order: Antipatharia; family: Myriopathidae; genus: Cupressopathes; **Location:** waterBody: Indian Ocean; country: Maldives; locality: North Male’, Vaavu, Addu; minimumDepthInMeters: 53; maximumDepthInMeters: 121; locationRemarks: Nekton Maldives Mission; **Identification:** identifiedBy: Farah Amjad, Paris Stefanoudis, Erika Gress; dateIdentified: 2022, 2023; identificationRemarks: Identified only from imagery; **Event:** samplingProtocol: Submersible OR Remotely Operated Vehicle OR Snorkel; **Record Level:** basisOfRecord: Human observation

### Myriopathes sp. indet. 1

B68C2375-3290-5345-A873-A270518B8E94

#### Materials

- **Type status:** Other material. **Taxon:** scientificName: *Myriopathes* sp. 1; kingdom: Animalia; phylum: Cnidaria; class: Anthozoa-Hexacorallia; order: Antipatharia; family: Myriopathidae; genus: Myriopathes; **Location:** waterBody: Indian Ocean; country: Maldives; locality: Laamu, Addu; minimumDepthInMeters: 60; maximumDepthInMeters: 250; locationRemarks: Nekton Maldives Mission; **Identification:** identifiedBy: Farah Amjad, Paris Stefanoudis, Erika Gress; dateIdentified: 2022, 2023; identificationRemarks: Identified only from imagery; **Event:** samplingProtocol: Submersible OR Remotely Operated Vehicle OR Snorkel; **Record Level:** basisOfRecord: Human observation

### Pteridopathes sp. indet.

5315B455-3C91-59D0-8F51-626F69C1F5DC

#### Materials

- **Type status:** Other material. **Taxon:** scientificName: *Pteridopathes* sp.; kingdom: Animalia; phylum: Cnidaria; class: Anthozoa-Hexacorallia; order: Antipatharia; family: Aphanipathidae; genus: Pteridopathes; **Location:** waterBody: Indian Ocean; country: Maldives; locality: North Male’, Laamu, Huvadhu; minimumDepthInMeters: 120; maximumDepthInMeters: 120; locationRemarks: Nekton Maldives Mission; **Identification:** identifiedBy: Farah Amjad, Paris Stefanoudis, Erika Gress; dateIdentified: 2022, 2023; identificationRemarks: Identified only from imagery; **Event:** samplingProtocol: Submersible OR Remotely Operated Vehicle OR Snorkel; **Record Level:** basisOfRecord: Human observation

### Stylopathes sp. indet.

47846B1A-11D9-5025-BB6D-53D5F83C434B

#### Materials

- **Type status:** Other material. **Taxon:** scientificName: *Stylopathes* sp.; kingdom: Animalia; phylum: Cnidaria; class: Anthozoa-Hexacorallia; order: Antipatharia; family: Stylopathidae; genus: Stylopathes; **Location:** waterBody: Indian Ocean; country: Maldives; locality: North Male’, Addu; minimumDepthInMeters: 115; maximumDepthInMeters: 253; locationRemarks: Nekton Maldives Mission; **Identification:** identifiedBy: Farah Amjad, Paris Stefanoudis, Erika Gress; dateIdentified: 2022, 2023; identificationRemarks: Identified only from imagery; **Event:** samplingProtocol: Submersible OR Remotely Operated Vehicle OR Snorkel; **Record Level:** basisOfRecord: Human observation

### Parantipathes sp. indet.

3439D426-3A82-5836-B8F5-5C6E7DB19CEB

#### Materials

- **Type status:** Other material. **Taxon:** scientificName: *Parantipathes* sp.; kingdom: Animalia; phylum: Cnidaria; class: Anthozoa-Hexacorallia; order: Antipatharia; family: Schizopathidae; genus: Parantipathes; **Location:** waterBody: Indian Ocean; country: Maldives; locality: Vaavu; minimumDepthInMeters: 121; maximumDepthInMeters: 489; locationRemarks: Nekton Maldives Mission; **Identification:** identifiedBy: Farah Amjad, Paris Stefanoudis, Erika Gress; dateIdentified: 2022, 2023; identificationRemarks: Identified only from imagery; **Event:** samplingProtocol: Submersible OR Remotely Operated Vehicle OR Snorkel; **Record Level:** basisOfRecord: Human observation

### Umbellapathes sp. indet.

19359EC2-9E4B-5956-A3D1-7480F5384FA9

#### Materials

- **Type status:** Other material. **Taxon:** scientificName: *Umbellapathes* sp.; kingdom: Animalia; phylum: Cnidaria; class: Anthozoa-Hexacorallia; order: Antipatharia; family: Schizopathidae; genus: Umbellapathes; **Location:** waterBody: Indian Ocean; country: Maldives; locality: Fuvahmulah; minimumDepthInMeters: 488; maximumDepthInMeters: 495; locationRemarks: Nekton Maldives Mission; **Identification:** identifiedBy: Farah Amjad, Paris Stefanoudis, Erika Gress; dateIdentified: 2022, 2023; identificationRemarks: Identified only from imagery; **Event:** samplingProtocol: Submersible OR Remotely Operated Vehicle OR Snorkel; **Record Level:** basisOfRecord: Human observation

### Antipatharia fam. indet. sp. 7

44198877-D447-587C-B2E9-4625D8336AD5

#### Materials

- **Type status:** Other material. **Occurrence:** occurrenceID: BB0ED6B0-0B6D-57D5-A724-76DC628F6A2E; **Taxon:** scientificName: Antipatharia sp. 7; kingdom: Animalia; phylum: Cnidaria; class: Anthozoa-Hexacorallia; order: Antipatharia; **Location:** waterBody: Indian Ocean; country: Maldives; locality: North Male’, Vaavu, Huvadhu, Addu; minimumDepthInMeters: 30; maximumDepthInMeters: 119; locationRemarks: Nekton Maldives Mission; **Identification:** identifiedBy: Farah Amjad, Paris Stefanoudis, Erika Gress; dateIdentified: 2022, 2023; identificationRemarks: Identified only from imagery; **Event:** samplingProtocol: Submersible OR Remotely Operated Vehicle OR Snorkel; **Record Level:** basisOfRecord: Human observation

### Rhodactis sp. indet.

26D0799C-64B9-597B-AF7B-014728F80F6F

#### Materials

- **Type status:** Other material. **Taxon:** scientificName: *Rhodactis* sp. indet.; kingdom: Animalia; phylum: Cnidaria; class: Anthozoa-Hexacorallia; order: Corallimorpharia; family: Discosomidae; genus: Rhodactis; **Location:** waterBody: Indian Ocean; country: Maldives; locality: North Male’, Huvadhu; minimumDepthInMeters: 10; maximumDepthInMeters: 10; locationRemarks: Nekton Maldives Mission; **Identification:** identifiedBy: Farah Amjad, Paris Stefanoudis, Erika Gress; dateIdentified: 2022, 2023; identificationRemarks: Identified only from imagery; **Event:** samplingProtocol: Submersible OR Remotely Operated Vehicle OR Snorkel; **Record Level:** basisOfRecord: Human observation

### Astrogorgia sp. indet.

662E566C-DD11-5B50-AD7F-ED6319594C34

#### Materials

- **Type status:** Other material. **Occurrence:** occurrenceID: F79BE385-60DD-57CF-8E64-2A1B735DBBF0; **Taxon:** scientificName: *Astrogorgia* sp.; kingdom: Animalia; phylum: Cnidaria; class: Anthozoa-Octocorallia; order: Malacalcyonacea; family: Astrogorgiidae; genus: Astrogorgia; **Location:** waterBody: Indian Ocean; country: Maldives; locality: Laamu, Huvadhu, Addu; minimumDepthInMeters: 30; maximumDepthInMeters: 60; locationRemarks: Nekton Maldives Mission; **Identification:** identifiedBy: Farah Amjad, Paris Stefanoudis; dateIdentified: 2022, 2023; identificationRemarks: Identified only from imagery; **Event:** samplingProtocol: Submersible OR Remotely Operated Vehicle OR Snorkel; **Record Level:** basisOfRecord: Human observation

### Melithaea sp. indet. 1

171FE032-9A86-5083-BA90-0747CF377C34

#### Materials

- **Type status:** Other material. **Occurrence:** occurrenceID: 060F5248-564A-55CD-B34E-568391657C50; **Taxon:** scientificName: *Melithaea* sp. 1; kingdom: Animalia; phylum: Cnidaria; class: Anthozoa-Octocorallia; order: Malacalcyonacea; family: Melithaeidae; genus: Melithaea; **Location:** waterBody: Indian Ocean; country: Maldives; locality: North Male’, Vaavu, Laamu, Addu; minimumDepthInMeters: 30; maximumDepthInMeters: 61; locationRemarks: Nekton Maldives Mission; **Identification:** identifiedBy: Farah Amjad, Paris Stefanoudis; dateIdentified: 2022, 2023; identificationRemarks: Identified only from imagery; **Event:** samplingProtocol: Submersible OR Remotely Operated Vehicle OR Snorkel; **Record Level:** basisOfRecord: Human observation

### Melithaea sp. indet. 2

E44DD05A-360B-57F3-997E-774545CB3D04

#### Materials

- **Type status:** Other material. **Occurrence:** occurrenceID: 9A0BD323-7824-53BB-BDAB-0D81AEDD908D; **Taxon:** scientificName: *Melithaea* sp. 2; kingdom: Animalia; phylum: Cnidaria; class: Anthozoa-Octocorallia; order: Malacalcyonacea; family: Melithaeidae; genus: Melithaea; **Location:** waterBody: Indian Ocean; country: Maldives; locality: Vaavu, Laamu, Huvadhu; minimumDepthInMeters: 8; maximumDepthInMeters: 61; locationRemarks: Nekton Maldives Mission; **Identification:** identifiedBy: Farah Amjad, Paris Stefanoudis; dateIdentified: 2022, 2023; identificationRemarks: Identified only from imagery; **Event:** samplingProtocol: Submersible OR Remotely Operated Vehicle OR Snorkel; **Record Level:** basisOfRecord: Human observation

### Dendronephthya sp. indet. 1

D239BFDB-CBF6-5559-94D2-57D837972C4D

#### Materials

- **Type status:** Other material. **Occurrence:** occurrenceID: D99A7691-6267-57AA-958E-AD984999210C; **Taxon:** scientificName: *Dendronephthya* sp. 1; kingdom: Animalia; phylum: Cnidaria; class: Anthozoa-Octocorallia; order: Malacalcyonacea; family: Nephtheidae; genus: Dendronephthya; **Location:** waterBody: Indian Ocean; country: Maldives; locality: Huvadhu; minimumDepthInMeters: 30; maximumDepthInMeters: 30; locationRemarks: Nekton Maldives Mission; **Identification:** identifiedBy: Farah Amjad, Paris Stefanoudis; dateIdentified: 2022, 2023; identificationRemarks: Identified only from imagery; **Event:** samplingProtocol: Submersible OR Remotely Operated Vehicle OR Snorkel; **Record Level:** basisOfRecord: Human observation

### Dendronephthya sp. indet. 4

77FF116D-FA6A-592B-86EF-7EF64EB8F86A

#### Materials

- **Type status:** Other material. **Occurrence:** occurrenceID: 6294C380-6FC3-5593-9618-006D404A329C; **Taxon:** scientificName: *Dendronephthya* sp. 4; kingdom: Animalia; phylum: Cnidaria; class: Anthozoa-Octocorallia; order: Malacalcyonacea; family: Nephtheidae; genus: Dendronephthya; **Location:** waterBody: Indian Ocean; country: Maldives; locality: Addu; minimumDepthInMeters: 60; maximumDepthInMeters: 60; locationRemarks: Nekton Maldives Mission; **Identification:** identifiedBy: Farah Amjad, Paris Stefanoudis; dateIdentified: 2022, 2023; identificationRemarks: Identified only from imagery; **Event:** samplingProtocol: Submersible OR Remotely Operated Vehicle OR Snorkel; **Record Level:** basisOfRecord: Human observation

### Nephtheidae gen. indet. sp. 5

EDFC3453-4C23-5771-A834-66A9501BD74E

#### Materials

- **Type status:** Other material. **Occurrence:** occurrenceID: 0A87234F-AE8F-538A-84A0-1C0A628BC6CE; **Taxon:** scientificName: Nephtheidae sp. 5; kingdom: Animalia; phylum: Cnidaria; class: Anthozoa-Octocorallia; order: Malacalcyonacea; family: Nephtheidae; **Location:** waterBody: Indian Ocean; country: Maldives; locality: Laamu, Huvadhu, Addu; minimumDepthInMeters: 30; maximumDepthInMeters: 121; locationRemarks: Nekton Maldives Mission; **Identification:** identifiedBy: Farah Amjad, Paris Stefanoudis; dateIdentified: 2022, 2023; identificationRemarks: Identified only from imagery; **Event:** samplingProtocol: Submersible OR Remotely Operated Vehicle OR Snorkel; **Record Level:** basisOfRecord: Human observation

### Scleronephthya sp. indet. 2

0D8C5958-37E9-5254-9806-DAFB95D8F187

#### Materials

- **Type status:** Other material. **Occurrence:** occurrenceID: 4A721492-64E6-5FB8-9487-C70851B1CF4B; **Taxon:** scientificName: *Scleronephthya* sp. 2; kingdom: Animalia; phylum: Cnidaria; class: Octocorallia; order: Malacalcyonacea; family: Nephtheidae; genus: Scleronephthya; scientificNameAuthorship: Studer, 1887; **Location:** waterBody: Indian Ocean; country: Maldives; locality: Huvadhu; minimumDepthInMeters: 60; maximumDepthInMeters: 60; locationRemarks: Nekton Maldives Mission; **Identification:** identifiedBy: Farah Amjad, Paris Stefanoudis; dateIdentified: 2022, 2023; identificationRemarks: Identified only from imagery; **Event:** samplingProtocol: Submersible OR Remotely Operated Vehicle OR Snorkel; **Record Level:** basisOfRecord: Human observation

### Umbellulifera sp. indet.

6B8A1EDF-3DA3-5CD5-AB6D-D73EFCF66CB3

#### Materials

- **Type status:** Other material. **Occurrence:** occurrenceID: 26BB05B8-5C69-5EAB-9A5E-5EEABAC65B1E; **Taxon:** scientificName: *Umbellulifera* sp.; kingdom: Animalia; phylum: Cnidaria; class: Anthozoa-Octocorallia; order: Malacalcyonacea; family: Nephtheidae; genus: Umbellulifera; **Location:** waterBody: Indian Ocean; country: Maldives; locality: North Male'; minimumDepthInMeters: 53; maximumDepthInMeters: 124; locationRemarks: Nekton Maldives Mission; **Identification:** identifiedBy: Farah Amjad, Paris Stefanoudis; dateIdentified: 2022, 2023; identificationRemarks: Identified only from imagery; **Event:** samplingProtocol: Submersible OR Remotely Operated Vehicle OR Snorkel; **Record Level:** basisOfRecord: Human observation

### Paramuriceidae gen. indet. sp. 1

A9BAAC0B-85CC-5DDD-B1BB-99C5E5414803

#### Materials

- **Type status:** Other material. **Occurrence:** occurrenceID: D82C4A71-A840-5137-9266-1247EDAE06B6; **Taxon:** scientificName: Paramuriceidae sp. 1; kingdom: Animalia; phylum: Cnidaria; class: Anthozoa-Octocorallia; order: Malacalcyonacea; family: Paramuriceidae; **Location:** waterBody: Indian Ocean; country: Maldives; locality: Vaavu, Fuvahmulah; minimumDepthInMeters: 118; maximumDepthInMeters: 120; locationRemarks: Nekton Maldives Mission; **Identification:** identifiedBy: Farah Amjad, Paris Stefanoudis; dateIdentified: 2022, 2023; identificationRemarks: Identified only from imagery; **Event:** samplingProtocol: Submersible OR Remotely Operated Vehicle OR Snorkel; **Record Level:** basisOfRecord: Human observation

### Acanthogorgia sp. indet.

1EA13EB4-2B7A-5B1B-B26B-092639DF26EB

#### Materials

- **Type status:** Other material. **Occurrence:** occurrenceID: 1DFDEFBB-A449-55D3-9AB8-0861F7931F97; **Taxon:** scientificName: *Acanthogorgia* sp.; kingdom: Animalia; phylum: Cnidaria; class: Anthozoa-Octocorallia; order: Malacalcyonacea; family: Paramuriceidae; genus: Acanthogorgia; **Location:** waterBody: Indian Ocean; country: Maldives; locality: Fuvahmulah; minimumDepthInMeters: 250; maximumDepthInMeters: 250; locationRemarks: Nekton Maldives Mission; **Identification:** identifiedBy: Farah Amjad, Paris Stefanoudis; dateIdentified: 2022, 2023; identificationRemarks: Identified only from imagery; **Event:** samplingProtocol: Submersible OR Remotely Operated Vehicle OR Snorkel; **Record Level:** basisOfRecord: Human observation

### Plexauridae gen. indet. sp. 2

9A4D0018-6DB0-5EB1-99AE-0806BE1021D2

#### Materials

- **Type status:** Other material. **Occurrence:** occurrenceID: B8F8B8C2-29F7-5A33-B33E-C91A3905E42A; **Taxon:** scientificName: Plexauridae sp. 2; kingdom: Animalia; phylum: Cnidaria; class: Anthozoa-Octocorallia; order: Malacalcyonacea; family: Plexauridae; **Location:** waterBody: Indian Ocean; country: Maldives; locality: North Male’, Vaavu, Laamu, Huvadhu, Addu, Fuvahmulah; minimumDepthInMeters: 10; maximumDepthInMeters: 121; locationRemarks: Nekton Maldives Mission; **Identification:** identifiedBy: Farah Amjad, Paris Stefanoudis; dateIdentified: 2022, 2023; identificationRemarks: Identified only from imagery; **Event:** samplingProtocol: Submersible OR Remotely Operated Vehicle OR Snorkel; **Record Level:** basisOfRecord: Human observation

### Plexauridae gen. indet. sp. 9

86CC2A03-9D4E-5532-B794-E3BCA4660512

#### Materials

- **Type status:** Other material. **Occurrence:** occurrenceID: 25F2F451-1578-5786-8F3F-BA79DAD3EA08; **Taxon:** scientificName: Plexauridae sp. 9; kingdom: Animalia; phylum: Cnidaria; class: Anthozoa-Octocorallia; order: Malacalcyonacea; family: Plexauridae; **Location:** waterBody: Indian Ocean; country: Maldives; locality: Addu; minimumDepthInMeters: 60; maximumDepthInMeters: 60; locationRemarks: Nekton Maldives Mission; **Identification:** identifiedBy: Farah Amjad, Paris Stefanoudis; dateIdentified: 2022, 2023; identificationRemarks: Identified only from imagery; **Event:** samplingProtocol: Submersible OR Remotely Operated Vehicle OR Snorkel; **Record Level:** basisOfRecord: Human observation

### Lobophytum sp. indet.

19AFDC12-AD97-561A-8663-56E54BF6BF91

#### Materials

- **Type status:** Other material. **Occurrence:** occurrenceID: B5340C84-399C-51F8-A409-997D89A10DC3; **Taxon:** scientificName: *Lobophytum* sp.; kingdom: Animalia; phylum: Cnidaria; class: Anthozoa-Octocorallia; order: Malacalcyonacea; family: Sarcophytidae; genus: Lobophytum; **Location:** waterBody: Indian Ocean; country: Maldives; locality: North Male’, Vaavu, Laamu, Huvadhu, Addu, Fuvahmulah; minimumDepthInMeters: 2; maximumDepthInMeters: 60; locationRemarks: Nekton Maldives Mission; **Identification:** identifiedBy: Farah Amjad, Paris Stefanoudis; dateIdentified: 2022, 2023; identificationRemarks: Identified only from imagery; **Event:** samplingProtocol: Submersible OR Remotely Operated Vehicle OR Snorkel; **Record Level:** basisOfRecord: Human observation

### Sarcophyton sp. indet.

261F4EF1-04E0-5A13-B9B7-C4D43C4B4C91

#### Materials

- **Type status:** Other material. **Occurrence:** occurrenceID: 89789312-D42C-5BF3-88B8-AF7B3D1E1BBA; **Taxon:** scientificName: *Sarcophyton* sp.; kingdom: Animalia; phylum: Cnidaria; class: Anthozoa-Octocorallia; order: Malacalcyonacea; family: Sarcophytidae; genus: Sarcophyton; **Location:** waterBody: Indian Ocean; country: Maldives; locality: North Male’, Vaavu, Laamu, Huvadhu, Addu; minimumDepthInMeters: 2; maximumDepthInMeters: 60; locationRemarks: Nekton Maldives Mission; **Identification:** identifiedBy: Farah Amjad, Paris Stefanoudis; dateIdentified: 2022, 2023; identificationRemarks: Identified only from imagery; **Event:** samplingProtocol: Submersible OR Remotely Operated Vehicle OR Snorkel; **Record Level:** basisOfRecord: Human observation

### Sinulariidae gen. indet. sp. 1

DB8E7C47-DFA7-5878-8733-A7FA95CBFB4F

#### Materials

- **Type status:** Other material. **Occurrence:** occurrenceID: E294CBE2-CCA3-503D-859B-9B45122C2D2F; **Taxon:** scientificName: Sinulariidae sp. 1; kingdom: Animalia; phylum: Cnidaria; class: Anthozoa-Octocorallia; order: Malacalcyonacea; family: Sinulariidae; **Location:** waterBody: Indian Ocean; country: Maldives; locality: North Male’, Vaavu, Laamu, Huvadhu; minimumDepthInMeters: 2; maximumDepthInMeters: 30; locationRemarks: Nekton Maldives Mission; **Identification:** identifiedBy: Farah Amjad, Paris Stefanoudis; dateIdentified: 2022, 2023; identificationRemarks: Identified only from imagery; **Event:** samplingProtocol: Submersible OR Remotely Operated Vehicle OR Snorkel; **Record Level:** basisOfRecord: Human observation

### Sinulariidae gen. indet. sp. 2

254EFC2E-6174-5C79-BD30-B822962CE0B4

#### Materials

- **Type status:** Other material. **Occurrence:** occurrenceID: 7D93116C-3280-599C-B73F-BFCA6EF0C558; **Taxon:** scientificName: Sinulariidae sp. 2; kingdom: Animalia; phylum: Cnidaria; class: Anthozoa-Octocorallia; order: Malacalcyonacea; family: Sinulariidae; **Location:** waterBody: Indian Ocean; country: Maldives; locality: Laamu, Huvadhu, Addu; minimumDepthInMeters: 2; maximumDepthInMeters: 30; locationRemarks: Nekton Maldives Mission; **Identification:** identifiedBy: Farah Amjad, Paris Stefanoudis; dateIdentified: 2022, 2023; identificationRemarks: Identified only from imagery; **Event:** samplingProtocol: Submersible OR Remotely Operated Vehicle OR Snorkel; **Record Level:** basisOfRecord: Human observation

### Sinulariidae gen. indet. sp. 3

DED9482E-8ADA-5F92-A6B7-A63DEE6E769D

#### Materials

- **Type status:** Other material. **Occurrence:** occurrenceID: BF5094EB-22B6-5B35-89BA-757FCCD23501; **Taxon:** scientificName: Sinulariidae sp. 3; kingdom: Animalia; phylum: Cnidaria; class: Anthozoa-Octocorallia; order: Malacalcyonacea; family: Sinulariidae; **Location:** waterBody: Indian Ocean; country: Maldives; locality: Laamu, Huvadhu, Addu; minimumDepthInMeters: 30; maximumDepthInMeters: 30; locationRemarks: Nekton Maldives Mission; **Identification:** identifiedBy: Farah Amjad, Paris Stefanoudis; dateIdentified: 2022, 2023; identificationRemarks: Identified only from imagery; **Event:** samplingProtocol: Submersible OR Remotely Operated Vehicle OR Snorkel; **Record Level:** basisOfRecord: Human observation

### Sinulariidae gen. indet. sp. 4

017CD7AE-5588-5D0A-A3BE-9658DF152884

#### Materials

- **Type status:** Other material. **Occurrence:** occurrenceID: 6C3F16EC-D175-5744-A3BA-5BFD50C627FA; **Taxon:** scientificName: Sinulariidae sp. 4; kingdom: Animalia; phylum: Cnidaria; class: Anthozoa-Octocorallia; order: Malacalcyonacea; family: Sinulariidae; **Location:** waterBody: Indian Ocean; country: Maldives; locality: North Male’, Vaavu, Addu; minimumDepthInMeters: 10; maximumDepthInMeters: 30; locationRemarks: Nekton Maldives Mission; **Identification:** identifiedBy: Farah Amjad, Paris Stefanoudis; dateIdentified: 2022, 2023; identificationRemarks: Identified only from imagery; **Event:** samplingProtocol: Submersible OR Remotely Operated Vehicle OR Snorkel; **Record Level:** basisOfRecord: Human observation

### Chironephthya sp. indet. 2

2BB26600-5537-58DE-A5DB-9C8922EB5623

#### Materials

- **Type status:** Other material. **Occurrence:** occurrenceID: E8B2CD37-3FEA-5722-81E8-F67CC9E48D48; **Taxon:** scientificName: *Chironephthya* sp. 2; kingdom: Animalia; phylum: Cnidaria; class: Anthozoa-Octocorallia; order: Malacalcyonacea; family: Siphonogorgiidae; genus: Chironephthya; **Location:** waterBody: Indian Ocean; country: Maldives; locality: Laamu, Addu; minimumDepthInMeters: 57; maximumDepthInMeters: 61; locationRemarks: Nekton Maldives Mission; **Identification:** identifiedBy: Farah Amjad, Paris Stefanoudis; dateIdentified: 2022, 2023; identificationRemarks: Identified only from imagery; **Event:** samplingProtocol: Submersible OR Remotely Operated Vehicle OR Snorkel; **Record Level:** basisOfRecord: Human observation

### Subergorgiidae gen. indet. sp. 1

A1382335-8B9A-5521-B6A6-70A0676FD16C

#### Materials

- **Type status:** Other material. **Occurrence:** occurrenceID: 4BC3D7CE-849E-5D5C-9586-58FA4A4526F7; **Taxon:** scientificName: Subergorgiidae sp. 1; kingdom: Animalia; phylum: Cnidaria; class: Anthozoa-Octocorallia; order: Malacalcyonacea; family: Subergorgiidae; **Location:** waterBody: Indian Ocean; country: Maldives; locality: North Male’, Laamu, Huvadhu, Addu; minimumDepthInMeters: 26; maximumDepthInMeters: 62; locationRemarks: Nekton Maldives Mission; **Identification:** identifiedBy: Farah Amjad, Paris Stefanoudis; dateIdentified: 2022, 2023; identificationRemarks: Identified only from imagery; **Event:** samplingProtocol: Submersible OR Remotely Operated Vehicle OR Snorkel; **Record Level:** basisOfRecord: Human observation

### Annella sp. indet.

2485E33A-55F4-5A78-A272-D03E35F51406

#### Materials

- **Type status:** Other material. **Occurrence:** occurrenceID: A251E1E2-CE17-5021-9E5F-421CF6E95EB6; **Taxon:** scientificName: *Annella* sp.; kingdom: Animalia; phylum: Cnidaria; class: Anthozoa-Octocorallia; order: Malacalcyonacea; family: Subergorgiidae; genus: Annella; **Location:** waterBody: Indian Ocean; country: Maldives; locality: North Male’, Vaavu, Laamu, Huvadhu, Addu, Fuvahmulah; minimumDepthInMeters: 53; maximumDepthInMeters: 121; locationRemarks: Nekton Maldives Mission; **Identification:** identifiedBy: Farah Amjad, Paris Stefanoudis; dateIdentified: 2022, 2023; identificationRemarks: Identified only from imagery; **Event:** samplingProtocol: Submersible OR Remotely Operated Vehicle OR Snorkel; **Record Level:** basisOfRecord: Human observation

### Subergorgia sp. indet. 2

904AE9F1-4440-5A57-BF6B-203E4F6E9764

#### Materials

- **Type status:** Other material. **Occurrence:** occurrenceID: A84DAD3B-C65F-53FA-806E-4C7AD5203852; **Taxon:** scientificName: *Subergorgia* sp. 2; kingdom: Animalia; phylum: Cnidaria; class: Anthozoa-Octocorallia; order: Malacalcyonacea; family: Subergorgiidae; genus: Subergorgia; **Location:** waterBody: Indian Ocean; country: Maldives; locality: Vaavu; minimumDepthInMeters: 118; maximumDepthInMeters: 120; locationRemarks: Nekton Maldives Mission; **Identification:** identifiedBy: Farah Amjad, Paris Stefanoudis; dateIdentified: 2022, 2023; identificationRemarks: Identified only from imagery; **Event:** samplingProtocol: Submersible OR Remotely Operated Vehicle OR Snorkel; **Record Level:** basisOfRecord: Human observation

### Malacalcyonacea fam. indet. sp.

EEE2ED3D-019B-53EF-8066-064A42AA4CFF

#### Materials

- **Type status:** Other material. **Occurrence:** occurrenceID: 2582E107-2A4A-5BF9-B495-2EBC4C5DC46D; **Taxon:** scientificName: Malacalcyonacea sp.; kingdom: Animalia; phylum: Cnidaria; class: Anthozoa-Octocorallia; order: Malacalcyonacea; **Location:** waterBody: Indian Ocean; country: Maldives; locality: North Male’, Vaavu, Huvadhu, Addu; minimumDepthInMeters: 30; maximumDepthInMeters: 62; locationRemarks: Nekton Maldives Mission; **Identification:** identifiedBy: Farah Amjad, Paris Stefanoudis; dateIdentified: 2022, 2023; identificationRemarks: Identified only from imagery; **Event:** samplingProtocol: Submersible OR Remotely Operated Vehicle OR Snorkel; **Record Level:** basisOfRecord: Human observation

### Ellisella sp. indet.

BBE68BDC-20CF-5ADC-800A-9DFE81CE5F5D

#### Materials

- **Type status:** Other material. **Occurrence:** occurrenceID: FE179DBC-7177-569F-9589-060816A41A01; **Taxon:** scientificName: *Ellisella* sp.; kingdom: Animalia; phylum: Cnidaria; class: Anthozoa-Octocorallia; order: Scleralcyonacea; family: Ellisellidae; genus: Ellisella; **Location:** waterBody: Indian Ocean; country: Maldives; locality: North Male’, Vaavu, Laamu, Huvadhu, Addu; minimumDepthInMeters: 30; maximumDepthInMeters: 124; locationRemarks: Nekton Maldives Mission; **Identification:** identifiedBy: Farah Amjad, Paris Stefanoudis; dateIdentified: 2022, 2023; identificationRemarks: Identified only from imagery; **Event:** samplingProtocol: Submersible OR Remotely Operated Vehicle OR Snorkel; **Record Level:** basisOfRecord: Human observation

### Nicella sp. indet.

CD22D1CF-C994-5570-842A-D6A7530FC57E

#### Materials

- **Type status:** Other material. **Occurrence:** occurrenceID: 803536AC-D39A-5F66-89DC-8CD59B64BBAA; **Taxon:** scientificName: *Nicella* sp.; kingdom: Animalia; phylum: Cnidaria; class: Anthozoa-Octocorallia; order: Scleralcyonacea; family: Ellisellidae; genus: Nicella; **Location:** waterBody: Indian Ocean; country: Maldives; locality: Laamu, Huvadhu, Addu; minimumDepthInMeters: 57; maximumDepthInMeters: 124; locationRemarks: Nekton Maldives Mission; **Identification:** identifiedBy: Farah Amjad, Paris Stefanoudis; dateIdentified: 2022, 2023; identificationRemarks: Identified only from imagery; **Event:** samplingProtocol: Submersible OR Remotely Operated Vehicle OR Snorkel; **Record Level:** basisOfRecord: Human observation

### Ellisellidae gen. indet. sp. 2

0C69E607-B96A-5EC8-85CB-C08A6EAD8D45

Ellisellidae gen. indet. sp. 2

#### Materials

- **Type status:** Other material. **Occurrence:** occurrenceID: 46508A3B-6A5E-5F14-A654-4230F3F6E66F; **Taxon:** scientificName: Ellisellidae sp. 2; kingdom: Animalia; phylum: Cnidaria; class: Anthozoa-Octocorallia; order: Scleralcyonacea; family: Ellisellidae; **Location:** waterBody: Indian Ocean; country: Maldives; locality: North Male’, Vaavu, Laamu, Huvadhu, Addu; minimumDepthInMeters: 10; maximumDepthInMeters: 124; locationRemarks: Nekton Maldives Mission; **Identification:** identifiedBy: Farah Amjad, Paris Stefanoudis; dateIdentified: 2022, 2023; identificationRemarks: Identified only from imagery; **Event:** samplingProtocol: Submersible OR Remotely Operated Vehicle OR Snorkel; **Record Level:** basisOfRecord: Human observation

### Ellisellidae gen. indet. sp. 4

AB5E4909-6398-50A5-A60B-7C06664A18B1

#### Materials

- **Type status:** Other material. **Occurrence:** occurrenceID: C2A1407A-86D9-5581-8624-2255E17A2031; **Taxon:** scientificName: Ellisellidae sp. 4; kingdom: Animalia; phylum: Cnidaria; class: Anthozoa-Octocorallia; order: Scleralcyonacea; family: Ellisellidae; **Location:** waterBody: Indian Ocean; country: Maldives; locality: North Male’, Vaavu, Huvadhu, Addu, Laamu; minimumDepthInMeters: 30; maximumDepthInMeters: 124; locationRemarks: Nekton Maldives Mission; **Identification:** identifiedBy: Farah Amjad, Paris Stefanoudis; dateIdentified: 2022, 2023; identificationRemarks: Identified only from imagery; **Event:** samplingProtocol: Submersible OR Remotely Operated Vehicle OR Snorkel; **Record Level:** basisOfRecord: Human observation

### Ellisellidae gen. indet. sp. 5

00E47021-4006-58B4-A201-CB729655883E

#### Materials

- **Type status:** Other material. **Occurrence:** occurrenceID: E8B070B9-0AC2-58E2-B29E-8E0DE0E3ED3B; **Taxon:** scientificName: Ellisellidae sp. 5; kingdom: Animalia; phylum: Cnidaria; class: Anthozoa-Octocorallia; order: Scleralcyonacea; family: Ellisellidae; **Location:** waterBody: Indian Ocean; country: Maldives; locality: North Male’, Huvadhu, Addu, Fuvahmulah; minimumDepthInMeters: 116; maximumDepthInMeters: 490; locationRemarks: Nekton Maldives Mission; **Identification:** identifiedBy: Farah Amjad, Paris Stefanoudis; dateIdentified: 2022, 2023; identificationRemarks: Identified only from imagery; **Event:** samplingProtocol: Submersible OR Remotely Operated Vehicle OR Snorkel; **Record Level:** basisOfRecord: Human observation

### Ellisellidae gen. indet. sp. 6

15B9B333-3CD8-55CB-8C1D-32B4E48171B6

#### Materials

- **Type status:** Other material. **Occurrence:** occurrenceID: A76BAF06-CE33-59E1-ABD3-84098DB797F8; **Taxon:** scientificName: Ellisellidae sp. 6; kingdom: Animalia; phylum: Cnidaria; class: Anthozoa-Octocorallia; order: Scleralcyonacea; family: Ellisellidae; **Location:** waterBody: Indian Ocean; country: Maldives; locality: North Male’, Vaavu, Laamu, Huvadhu, Addu; minimumDepthInMeters: 52; maximumDepthInMeters: 121; locationRemarks: Nekton Maldives Mission; **Identification:** identifiedBy: Farah Amjad, Paris Stefanoudis; dateIdentified: 2022, 2023; identificationRemarks: Identified only from imagery; **Event:** samplingProtocol: Submersible OR Remotely Operated Vehicle OR Snorkel; **Record Level:** basisOfRecord: Human observation

### Ellisellidae gen. indet. sp. 8

8BF82BCB-E58E-5F22-BC26-6480CAE215A8

#### Materials

- **Type status:** Other material. **Occurrence:** occurrenceID: 4713C20B-2856-58EC-BBA4-DB071D223E5C; **Taxon:** scientificName: Ellisellidae sp. 8; kingdom: Animalia; phylum: Cnidaria; class: Anthozoa-Octocorallia; order: Scleralcyonacea; family: Ellisellidae; **Location:** waterBody: Indian Ocean; country: Maldives; locality: Huvadhu, Addu; minimumDepthInMeters: 60; maximumDepthInMeters: 120; locationRemarks: Nekton Maldives Mission; **Identification:** identifiedBy: Farah Amjad, Paris Stefanoudis; dateIdentified: 2022, 2023; identificationRemarks: Identified only from imagery; **Event:** samplingProtocol: Submersible OR Remotely Operated Vehicle OR Snorkel; **Record Level:** basisOfRecord: Human observation

### Ellisellidae gen. indet. sp. 9

E466A3D3-C6A2-591E-9B05-39036F6EE79A

#### Materials

- **Type status:** Other material. **Taxon:** scientificName: Ellisellidae sp. 9; kingdom: Animalia; phylum: Cnidaria; class: Anthozoa-Octocorallia; order: Scleralcyonacea; family: Ellisellidae; **Location:** waterBody: Indian Ocean; country: Maldives; locality: North Male'; minimumDepthInMeters: 250; maximumDepthInMeters: 253; locationRemarks: Nekton Maldives Mission; **Identification:** identifiedBy: Farah Amjad, Paris Stefanoudis; dateIdentified: 2022, 2023; identificationRemarks: Identified only from imagery; **Event:** samplingProtocol: Submersible OR Remotely Operated Vehicle OR Snorkel; **Record Level:** basisOfRecord: Human observation

### Heliopora sp. indet.

452D4E5F-C3A7-55E8-9E04-C95E09A18A7D

#### Materials

- **Type status:** Other material. **Taxon:** scientificName: *Heliopora* sp.; kingdom: Animalia; phylum: Cnidaria; class: Anthozoa-Octocorallia; order: Scleralcyonacea; family: Helioporidae; genus: Heliopora; **Location:** waterBody: Indian Ocean; country: Maldives; locality: North Male’, Vaavu, Laamu, Addu; minimumDepthInMeters: 2; maximumDepthInMeters: 30; locationRemarks: Nekton Maldives Mission; **Identification:** identifiedBy: Farah Amjad, Paris Stefanoudis; dateIdentified: 2022, 2023; identificationRemarks: Identified only from imagery; **Event:** samplingProtocol: Submersible OR Remotely Operated Vehicle OR Snorkel; **Record Level:** basisOfRecord: Human observation

### Pennatuloidea gen. indet. sp.

60095B8C-7AF3-5356-A8F9-6DE33E5FDB1D

#### Materials

- **Type status:** Other material. **Taxon:** scientificName: Pennatuloidea sp.; kingdom: Animalia; phylum: Cnidaria; class: Anthozoa-Octocorallia; order: Scleralcyonacea; family: Pennatuloidea; **Location:** waterBody: Indian Ocean; country: Maldives; locality: Vaavu, Fuvahmulah; minimumDepthInMeters: 489; maximumDepthInMeters: 489; locationRemarks: Nekton Maldives Mission; **Identification:** identifiedBy: Farah Amjad, Paris Stefanoudis; dateIdentified: 2022, 2024; identificationRemarks: Identified only from imagery; **Event:** samplingProtocol: Submersible OR Remotely Operated Vehicle OR Snorkel; **Record Level:** basisOfRecord: Human observation

### Primnoidae gen. indet. sp. 1

89C6727E-1E90-56BD-9072-E2651C16A98D

#### Materials

- **Type status:** Other material. **Occurrence:** occurrenceID: DFBCDC4A-E3A6-5CDB-858A-5FB32A9173E6; **Taxon:** scientificName: Primnoidae sp. 1; kingdom: Animalia; phylum: Cnidaria; class: Anthozoa-Octocorallia; order: Scleralcyonacea; family: Primnoidae; **Location:** waterBody: Indian Ocean; country: Maldives; locality: Vaavu, Laamu, Fuvahmulah; minimumDepthInMeters: 53; maximumDepthInMeters: 491; locationRemarks: Nekton Maldives Mission; **Identification:** identifiedBy: Farah Amjad, Paris Stefanoudis; dateIdentified: 2022, 2025; identificationRemarks: Identified only from imagery; **Event:** samplingProtocol: Submersible OR Remotely Operated Vehicle OR Snorkel; **Record Level:** basisOfRecord: Human observation

### Primnoidae gen. indet. sp. 2

1E669403-3D1A-5FE8-A94C-29F70785A5E2

#### Materials

- **Type status:** Other material. **Occurrence:** occurrenceID: 276EEE10-9A48-5BE1-8CE7-A80B3D080EA6; **Taxon:** scientificName: Primnoidae sp. 2; kingdom: Animalia; phylum: Cnidaria; class: Anthozoa-Octocorallia; order: Scleralcyonacea; family: Primnoidae; **Location:** waterBody: Indian Ocean; country: Maldives; locality: Laamu; minimumDepthInMeters: 30; maximumDepthInMeters: 60; locationRemarks: Nekton Maldives Mission; **Identification:** identifiedBy: Farah Amjad, Paris Stefanoudis; dateIdentified: 2022, 2026; identificationRemarks: Identified only from imagery; **Event:** samplingProtocol: Submersible OR Remotely Operated Vehicle OR Snorkel; **Record Level:** basisOfRecord: Human observation

### Primnoidae gen. indet. sp. 3

622A1BEC-125F-5ADC-8474-BB371F7621D2

#### Materials

- **Type status:** Other material. **Occurrence:** occurrenceID: C2A171E6-BDF5-51D9-8C9D-A9E5203E1673; **Taxon:** scientificName: Primnoidae sp. 3; kingdom: Animalia; phylum: Cnidaria; class: Anthozoa-Octocorallia; order: Scleralcyonacea; family: Primnoidae; **Location:** waterBody: Indian Ocean; country: Maldives; locality: Huvadhu; minimumDepthInMeters: 248; maximumDepthInMeters: 253; locationRemarks: Nekton Maldives Mission; **Identification:** identifiedBy: Farah Amjad, Paris Stefanoudis; dateIdentified: 2022, 2027; identificationRemarks: Identified only from imagery; **Event:** samplingProtocol: Submersible OR Remotely Operated Vehicle OR Snorkel; **Record Level:** basisOfRecord: Human observation

### Primnoidae gen. indet. sp. 4

19BDA55E-9998-5CE6-850E-37E14EC09BE1

#### Materials

- **Type status:** Other material. **Occurrence:** occurrenceID: 683B729C-E284-5246-BF3A-AA949A59F960; **Taxon:** scientificName: Primnoidae sp. 4; kingdom: Animalia; phylum: Cnidaria; class: Anthozoa-Octocorallia; order: Scleralcyonacea; family: Primnoidae; **Location:** waterBody: Indian Ocean; country: Maldives; locality: Laamu; minimumDepthInMeters: 488; maximumDepthInMeters: 490; locationRemarks: Nekton Maldives Mission; **Identification:** identifiedBy: Farah Amjad, Paris Stefanoudis; dateIdentified: 2022, 2028; identificationRemarks: Identified only from imagery; **Event:** samplingProtocol: Submersible OR Remotely Operated Vehicle OR Snorkel; **Record Level:** basisOfRecord: Human observation

### Primnoidae gen. indet. sp. 5

BD78F716-E4D8-53F8-AF30-200C9B4BA5A8

#### Materials

- **Type status:** Other material. **Occurrence:** occurrenceID: 937DF3BC-FD43-53BA-A246-D33D664A7BC7; **Taxon:** scientificName: Primnoidae sp. 5; kingdom: Animalia; phylum: Cnidaria; class: Anthozoa-Octocorallia; order: Scleralcyonacea; family: Primnoidae; **Location:** waterBody: Indian Ocean; country: Maldives; locality: Huvadhu; minimumDepthInMeters: 489; maximumDepthInMeters: 489; locationRemarks: Nekton Maldives Mission; **Identification:** identifiedBy: Farah Amjad, Paris Stefanoudis; dateIdentified: 2022, 2029; identificationRemarks: Identified only from imagery; **Event:** samplingProtocol: Submersible OR Remotely Operated Vehicle OR Snorkel; **Record Level:** basisOfRecord: Human observation

### Octocorallia ord. indet. sp. 3

0223EE9A-F076-5872-801F-A7B4B54BB07F

#### Materials

- **Type status:** Other material. **Occurrence:** occurrenceID: 6FED4268-A55F-593A-B20C-D320AFC81C2D; **Taxon:** scientificName: Octocorallia sp. 3; kingdom: Animalia; phylum: Cnidaria; class: Anthozoa-Octocorallia; **Location:** waterBody: Indian Ocean; country: Maldives; locality: Vaavu, Laamu, Huvadhu, Addu; minimumDepthInMeters: 30; maximumDepthInMeters: 121; locationRemarks: Nekton Maldives Mission; **Identification:** identifiedBy: Farah Amjad, Paris Stefanoudis; dateIdentified: 2022, 2030; identificationRemarks: Identified only from imagery; **Event:** samplingProtocol: Submersible OR Remotely Operated Vehicle OR Snorkel; **Record Level:** basisOfRecord: Human observation

### Octocorallia ord. indet. sp. 4

820B6434-64B2-5BEA-ACC1-20BD0F07C969

#### Materials

- **Type status:** Other material. **Taxon:** scientificName: Octocorallia sp. 4; kingdom: Animalia; phylum: Cnidaria; class: Anthozoa-Octocorallia; **Location:** waterBody: Indian Ocean; country: Maldives; locality: North Male’, Vaavu, Huvadhu, Addu, Fuvahmulah; minimumDepthInMeters: 59; maximumDepthInMeters: 124; locationRemarks: Nekton Maldives Mission; **Identification:** identifiedBy: Farah Amjad, Paris Stefanoudis; dateIdentified: 2022, 2031; identificationRemarks: Identified only from imagery; **Event:** samplingProtocol: Submersible OR Remotely Operated Vehicle OR Snorkel; **Record Level:** basisOfRecord: Human observation

### Octocorallia ord. indet. sp. 5

A9D04972-0A62-5B70-A4EC-41AFF7B39F87

#### Materials

- **Type status:** Other material. **Taxon:** scientificName: Octocorallia sp. 5; kingdom: Animalia; phylum: Cnidaria; class: Anthozoa-Octocorallia; **Location:** waterBody: Indian Ocean; country: Maldives; locality: Vaavu, Laamu, Addu; minimumDepthInMeters: 57; maximumDepthInMeters: 124; locationRemarks: Nekton Maldives Mission; **Identification:** identifiedBy: Farah Amjad, Paris Stefanoudis; dateIdentified: 2022, 2032; identificationRemarks: Identified only from imagery; **Event:** samplingProtocol: Submersible OR Remotely Operated Vehicle OR Snorkel; **Record Level:** basisOfRecord: Human observation

### Octocorallia ord. indet. sp. 6

381FBA46-3636-549A-9BB0-E6856A5672C2

#### Materials

- **Type status:** Other material. **Taxon:** scientificName: Octocorallia sp. 6; kingdom: Animalia; phylum: Cnidaria; class: Anthozoa-Octocorallia; **Location:** waterBody: Indian Ocean; country: Maldives; locality: North Male’, Vaavu, Huvadhu; minimumDepthInMeters: 11; maximumDepthInMeters: 124; locationRemarks: Nekton Maldives Mission; **Identification:** identifiedBy: Farah Amjad, Paris Stefanoudis; dateIdentified: 2022, 2033; identificationRemarks: Identified only from imagery; **Event:** samplingProtocol: Submersible OR Remotely Operated Vehicle OR Snorkel; **Record Level:** basisOfRecord: Human observation

### Octocorallia ord. indet. sp. 7

3A3FF843-4E4E-5C51-837B-8BD23FEEF245

#### Materials

- **Type status:** Other material. **Taxon:** scientificName: Octocorallia sp. 7; kingdom: Animalia; phylum: Cnidaria; class: Anthozoa-Octocorallia; **Location:** waterBody: Indian Ocean; country: Maldives; locality: North Male’, Vaavu, Laamu, Fuvahmulah, Addu; minimumDepthInMeters: 53; maximumDepthInMeters: 124; locationRemarks: Nekton Maldives Mission; **Identification:** identifiedBy: Farah Amjad, Paris Stefanoudis; dateIdentified: 2022, 2034; identificationRemarks: Identified only from imagery; **Event:** samplingProtocol: Submersible OR Remotely Operated Vehicle OR Snorkel; **Record Level:** basisOfRecord: Human observation

### Octocorallia ord. indet. sp. 8

55C6E2E3-7C9F-5A1A-B54C-E036FAAF0208

#### Materials

- **Type status:** Other material. **Taxon:** scientificName: Octocorallia sp. 8; kingdom: Animalia; phylum: Cnidaria; class: Anthozoa-Octocorallia; **Location:** waterBody: Indian Ocean; country: Maldives; locality: North Male’, Laamu; minimumDepthInMeters: 60; maximumDepthInMeters: 60; locationRemarks: Nekton Maldives Mission; **Identification:** identifiedBy: Farah Amjad, Paris Stefanoudis; dateIdentified: 2022, 2035; identificationRemarks: Identified only from imagery; **Event:** samplingProtocol: Submersible OR Remotely Operated Vehicle OR Snorkel; **Record Level:** basisOfRecord: Human observation

### Octocorallia ord. indet. sp. 9

EF4CAA5E-177A-560D-B452-3D89BEF773A0

#### Materials

- **Type status:** Other material. **Taxon:** scientificName: Octocorallia sp. 9; kingdom: Animalia; phylum: Cnidaria; class: Anthozoa-Octocorallia; **Location:** waterBody: Indian Ocean; country: Maldives; locality: North Male’, Vaavu, Laamu; minimumDepthInMeters: 53; maximumDepthInMeters: 124; locationRemarks: Nekton Maldives Mission; **Identification:** identifiedBy: Farah Amjad, Paris Stefanoudis; dateIdentified: 2022, 2036; identificationRemarks: Identified only from imagery; **Event:** samplingProtocol: Submersible OR Remotely Operated Vehicle OR Snorkel; **Record Level:** basisOfRecord: Human observation

### Octocorallia ord. indet. sp. 10

199EB0B1-428A-55D9-A383-D8ADF056289F

#### Materials

- **Type status:** Other material. **Taxon:** scientificName: Octocorallia sp. 10; kingdom: Animalia; phylum: Cnidaria; class: Anthozoa-Octocorallia; **Location:** waterBody: Indian Ocean; country: Maldives; locality: Addu, Fuvahmulah; minimumDepthInMeters: 118; maximumDepthInMeters: 121; locationRemarks: Nekton Maldives Mission; **Identification:** identifiedBy: Farah Amjad, Paris Stefanoudis; dateIdentified: 2022, 2037; identificationRemarks: Identified only from imagery; **Event:** samplingProtocol: Submersible OR Remotely Operated Vehicle OR Snorkel; **Record Level:** basisOfRecord: Human observation

### Octocorallia ord. indet. sp. 11

EBA98AC6-508C-55C9-8798-1C424F49881B

#### Materials

- **Type status:** Other material. **Taxon:** scientificName: Octocorallia sp. 11; kingdom: Animalia; phylum: Cnidaria; class: Anthozoa-Octocorallia; **Location:** waterBody: Indian Ocean; country: Maldives; locality: Fuvahmulah; minimumDepthInMeters: 490; maximumDepthInMeters: 490; locationRemarks: Nekton Maldives Mission; **Identification:** identifiedBy: Farah Amjad, Paris Stefanoudis; dateIdentified: 2022, 2038; identificationRemarks: Identified only from imagery; **Event:** samplingProtocol: Submersible OR Remotely Operated Vehicle OR Snorkel; **Record Level:** basisOfRecord: Human observation

### Octocorallia ord. indet. sp. 12

A6B032D5-4D10-5E43-86C9-E16D5BFCE22C

#### Materials

- **Type status:** Other material. **Taxon:** scientificName: Octocorallia sp. 12; kingdom: Animalia; phylum: Cnidaria; class: Anthozoa-Octocorallia; **Location:** waterBody: Indian Ocean; country: Maldives; locality: Huvadhu; minimumDepthInMeters: 489; maximumDepthInMeters: 489; locationRemarks: Nekton Maldives Mission; **Identification:** identifiedBy: Farah Amjad, Paris Stefanoudis; dateIdentified: 2022, 2039; identificationRemarks: Identified only from imagery; **Event:** samplingProtocol: Submersible OR Remotely Operated Vehicle OR Snorkel; **Record Level:** basisOfRecord: Human observation

### Octocorallia ord. indet. sp. 13

9D65C92D-CD6C-5414-9A88-C358123EBBFC

#### Materials

- **Type status:** Other material. **Taxon:** scientificName: Octocorallia sp. 13; kingdom: Animalia; phylum: Cnidaria; class: Anthozoa-Octocorallia; **Location:** waterBody: Indian Ocean; country: Maldives; locality: Fuvahmulah; minimumDepthInMeters: 120; maximumDepthInMeters: 120; locationRemarks: Nekton Maldives Mission; **Identification:** identifiedBy: Farah Amjad, Paris Stefanoudis; dateIdentified: 2022, 2040; identificationRemarks: Identified only from imagery; **Event:** samplingProtocol: Submersible OR Remotely Operated Vehicle OR Snorkel; **Record Level:** basisOfRecord: Human observation

### Octocorallia ord. indet. sp. 14

C1627432-04DE-5675-9586-09C83ED79845

#### Materials

- **Type status:** Other material. **Taxon:** scientificName: Octocorallia sp. 14; kingdom: Animalia; phylum: Cnidaria; class: Anthozoa-Octocorallia; **Location:** waterBody: Indian Ocean; country: Maldives; locality: Addu; minimumDepthInMeters: 248; maximumDepthInMeters: 250; locationRemarks: Nekton Maldives Mission; **Identification:** identifiedBy: Farah Amjad, Paris Stefanoudis; dateIdentified: 2022, 2041; identificationRemarks: Identified only from imagery; **Event:** samplingProtocol: Submersible OR Remotely Operated Vehicle OR Snorkel; **Record Level:** basisOfRecord: Human observation

### Octocorallia ord. indet. sp. 15

E54D8347-2986-5F34-BA2C-EA8540563934

#### Materials

- **Type status:** Other material. **Taxon:** scientificName: Octocorallia sp. 15; kingdom: Animalia; phylum: Cnidaria; class: Anthozoa-Octocorallia; **Location:** waterBody: Indian Ocean; country: Maldives; locality: Laamu; minimumDepthInMeters: 59; maximumDepthInMeters: 60; locationRemarks: Nekton Maldives Mission; **Identification:** identifiedBy: Farah Amjad, Paris Stefanoudis; dateIdentified: 2022, 2042; identificationRemarks: Identified only from imagery; **Event:** samplingProtocol: Submersible OR Remotely Operated Vehicle OR Snorkel; **Record Level:** basisOfRecord: Human observation

### Octocorallia ord. indet. sp. 18

8BBC2B63-C4CA-5BC7-A40D-3AAC839C0DD7

#### Materials

- **Type status:** Other material. **Taxon:** scientificName: Octocorallia sp. 18; kingdom: Animalia; phylum: Cnidaria; class: Anthozoa-Octocorallia; **Location:** waterBody: Indian Ocean; country: Maldives; locality: Vaavu, Laamu; minimumDepthInMeters: 53; maximumDepthInMeters: 61; locationRemarks: Nekton Maldives Mission; **Identification:** identifiedBy: Farah Amjad, Paris Stefanoudis; dateIdentified: 2022, 2044; identificationRemarks: Identified only from imagery; **Event:** samplingProtocol: Submersible OR Remotely Operated Vehicle OR Snorkel; **Record Level:** basisOfRecord: Human observation

### Octocorallia ord. indet. sp. 19

C8786BC7-C433-5940-952C-74E4B8ED8791

#### Materials

- **Type status:** Other material. **Taxon:** scientificName: Octocorallia sp. 19; kingdom: Animalia; phylum: Cnidaria; class: Anthozoa-Octocorallia; **Location:** waterBody: Indian Ocean; country: Maldives; locality: Fuvahmulah; minimumDepthInMeters: 490; maximumDepthInMeters: 490; locationRemarks: Nekton Maldives Mission; **Identification:** identifiedBy: Farah Amjad, Paris Stefanoudis; dateIdentified: 2022, 2045; identificationRemarks: Identified only from imagery; **Event:** samplingProtocol: Submersible OR Remotely Operated Vehicle OR Snorkel; **Record Level:** basisOfRecord: Human observation

### Octocorallia ord. indet. sp. 20

40D40832-1571-568B-8572-96098CE5B51C

#### Materials

- **Type status:** Other material. **Taxon:** scientificName: Octocorallia sp. 20; kingdom: Animalia; phylum: Cnidaria; class: Anthozoa-Octocorallia; **Location:** waterBody: Indian Ocean; country: Maldives; locality: North Male’, Laamu; minimumDepthInMeters: 30; maximumDepthInMeters: 124; locationRemarks: Nekton Maldives Mission; **Identification:** identifiedBy: Farah Amjad, Paris Stefanoudis; dateIdentified: 2022, 2046; identificationRemarks: Identified only from imagery; **Event:** samplingProtocol: Submersible OR Remotely Operated Vehicle OR Snorkel; **Record Level:** basisOfRecord: Human observation

### Octocorallia ord. indet. sp. 21

14FEC0AA-B344-57E9-8322-23672E3DDA55

#### Materials

- **Type status:** Other material. **Taxon:** scientificName: Octocorallia sp. 21; kingdom: Animalia; phylum: Cnidaria; class: Anthozoa-Octocorallia; **Location:** waterBody: Indian Ocean; country: Maldives; locality: Fuvahmulah; minimumDepthInMeters: 120; maximumDepthInMeters: 120; locationRemarks: Nekton Maldives Mission; **Identification:** identifiedBy: Farah Amjad, Paris Stefanoudis; dateIdentified: 2022, 2047; identificationRemarks: Identified only from imagery; **Event:** samplingProtocol: Submersible OR Remotely Operated Vehicle OR Snorkel; **Record Level:** basisOfRecord: Human observation

### Octocorallia ord. indet. sp. 22

540244A8-0E6C-58C8-8EE8-C4BAC00A74CC

#### Materials

- **Type status:** Other material. **Taxon:** scientificName: Octocorallia sp. 22; kingdom: Animalia; phylum: Cnidaria; class: Anthozoa-Octocorallia; **Location:** waterBody: Indian Ocean; country: Maldives; locality: Laamu, Fuvahmulah; minimumDepthInMeters: 57; maximumDepthInMeters: 61; locationRemarks: Nekton Maldives Mission; **Identification:** identifiedBy: Farah Amjad, Paris Stefanoudis; dateIdentified: 2022, 2048; identificationRemarks: Identified only from imagery; **Event:** samplingProtocol: Submersible OR Remotely Operated Vehicle OR Snorkel; **Record Level:** basisOfRecord: Human observation

### Octocorallia ord. indet. sp. 24

A8464F8C-9278-5D39-A27A-08FA0F124EE6

#### Materials

- **Type status:** Other material. **Taxon:** scientificName: Octocorallia sp. 24; kingdom: Animalia; phylum: Cnidaria; class: Anthozoa-Octocorallia; **Location:** waterBody: Indian Ocean; country: Maldives; locality: North Male’, Vaavu, Huvadhu, Addu; minimumDepthInMeters: 26; maximumDepthInMeters: 124; locationRemarks: Nekton Maldives Mission; **Identification:** identifiedBy: Farah Amjad, Paris Stefanoudis; dateIdentified: 2022, 2049; identificationRemarks: Identified only from imagery; **Event:** samplingProtocol: Submersible OR Remotely Operated Vehicle OR Snorkel; **Record Level:** basisOfRecord: Human observation

### Octocorallia ord. indet. sp. 25

4C76B92B-B9BB-5C60-BE54-6208D1C65665

#### Materials

- **Type status:** Other material. **Taxon:** scientificName: Octocorallia sp. 25; kingdom: Animalia; phylum: Cnidaria; class: Anthozoa-Octocorallia; **Location:** waterBody: Indian Ocean; country: Maldives; locality: North Male’, Laamu, Huvadhu, Addu; minimumDepthInMeters: 53; maximumDepthInMeters: 120; locationRemarks: Nekton Maldives Mission; **Identification:** identifiedBy: Farah Amjad, Paris Stefanoudis; dateIdentified: 2022, 2050; identificationRemarks: Identified only from imagery; **Event:** samplingProtocol: Submersible OR Remotely Operated Vehicle OR Snorkel; **Record Level:** basisOfRecord: Human observation

### Octocorallia ord. indet. sp. 27

069CF2C7-FE68-5AF5-BC3E-07A8E4F5A37B

#### Materials

- **Type status:** Other material. **Taxon:** scientificName: Octocorallia sp. 27; kingdom: Animalia; phylum: Cnidaria; class: Anthozoa-Octocorallia; **Location:** waterBody: Indian Ocean; country: Maldives; locality: Huvadhu; minimumDepthInMeters: 30; maximumDepthInMeters: 62; locationRemarks: Nekton Maldives Mission; **Identification:** identifiedBy: Farah Amjad, Paris Stefanoudis; dateIdentified: 2022, 2051; identificationRemarks: Identified only from imagery; **Event:** samplingProtocol: Submersible OR Remotely Operated Vehicle OR Snorkel; **Record Level:** basisOfRecord: Human observation

### Octocorallia ord. indet. sp. 29

23ECBBEB-DB74-51C3-A99E-3A6EF204CCA9

#### Materials

- **Type status:** Other material. **Taxon:** scientificName: Octocorallia sp. 29; kingdom: Animalia; phylum: Cnidaria; class: Anthozoa-Octocorallia; **Location:** waterBody: Indian Ocean; country: Maldives; locality: Huvadhu, Addu; minimumDepthInMeters: 59; maximumDepthInMeters: 121; locationRemarks: Nekton Maldives Mission; **Identification:** identifiedBy: Farah Amjad, Paris Stefanoudis; dateIdentified: 2022, 2052; identificationRemarks: Identified only from imagery; **Event:** samplingProtocol: Submersible OR Remotely Operated Vehicle OR Snorkel; **Record Level:** basisOfRecord: Human observation

### Octocorallia ord. indet. sp. 31

BE2B74F7-E861-525E-A9CE-508DFDE22F6E

#### Materials

- **Type status:** Other material. **Taxon:** scientificName: Octocorallia sp. 31; kingdom: Animalia; phylum: Cnidaria; class: Anthozoa-Octocorallia; **Location:** waterBody: Indian Ocean; country: Maldives; locality: North Male’, Vaavu, Huvadhu, Addu; minimumDepthInMeters: 115; maximumDepthInMeters: 120; locationRemarks: Nekton Maldives Mission; **Identification:** identifiedBy: Farah Amjad, Paris Stefanoudis; dateIdentified: 2022, 2053; identificationRemarks: Identified only from imagery; **Event:** samplingProtocol: Submersible OR Remotely Operated Vehicle OR Snorkel; **Record Level:** basisOfRecord: Human observation

### Ceriantharia stet.

B692A471-C056-5E5F-9003-1431E3B19899

#### Materials

- **Type status:** Other material. **Taxon:** scientificName: Ceriantharia; kingdom: Animalia; phylum: Cnidaria; class: Anthozoa-Ceriantharia; **Location:** waterBody: Indian Ocean; country: Maldives; locality: North Male’; minimumDepthInMeters: 247; maximumDepthInMeters: 251; locationRemarks: Nekton Maldives Mission; **Identification:** identifiedBy: Farah Amjad, Paris Stefanoudis; dateIdentified: 2022, 2023; identificationRemarks: Identified only from imagery; **Event:** samplingProtocol: Submersible OR Remotely Operated Vehicle OR Snorkel; **Record Level:** basisOfRecord: Human observation

### Millepora sp. indet. 1

5921B515-D599-5E01-A483-A06A677A2709

#### Materials

- **Type status:** Other material. **Taxon:** scientificName: *Millepora* sp. 1; kingdom: Animalia; phylum: Cnidaria; class: Hydrozoa; order: Anthoathecata; family: Milleporidae; genus: Millepora; **Location:** waterBody: Indian Ocean; country: Maldives; locality: North Male’, Vaavu, Laamu, Addu; minimumDepthInMeters: 2; maximumDepthInMeters: 30; locationRemarks: Nekton Maldives Mission; **Identification:** identifiedBy: Farah Amjad, Paris Stefanoudis; dateIdentified: 2022, 2023; identificationRemarks: Identified only from imagery; **Event:** samplingProtocol: Submersible OR Remotely Operated Vehicle OR Snorkel; **Record Level:** basisOfRecord: Human observation

### Millepora sp. indet. 2

893E478B-B2D2-58D9-A821-95DF6CBEA6A5

#### Materials

- **Type status:** Other material. **Taxon:** scientificName: *Millepora* sp. 2; kingdom: Animalia; phylum: Cnidaria; class: Hydrozoa; order: Anthoathecata; family: Milleporidae; genus: Millepora; **Location:** waterBody: Indian Ocean; country: Maldives; locality: North Male’, Laamu, Huvadhu; minimumDepthInMeters: 8; maximumDepthInMeters: 30; locationRemarks: Nekton Maldives Mission; **Identification:** identifiedBy: Farah Amjad, Paris Stefanoudis; dateIdentified: 2022, 2023; identificationRemarks: Identified only from imagery; **Event:** samplingProtocol: Submersible OR Remotely Operated Vehicle OR Snorkel; **Record Level:** basisOfRecord: Human observation

### Millepora sp. indet. 3

AD1BAEAD-C4D4-548E-819A-34B300A47122

#### Materials

- **Type status:** Other material. **Taxon:** scientificName: *Millepora* sp. 3; kingdom: Animalia; phylum: Cnidaria; class: Hydrozoa; order: Anthoathecata; family: Milleporidae; genus: Millepora; **Location:** waterBody: Indian Ocean; country: Maldives; locality: North Male’, Vaavu, Laamu, Huvadhu, Addu; minimumDepthInMeters: 2; maximumDepthInMeters: 30; locationRemarks: Nekton Maldives Mission; **Identification:** identifiedBy: Farah Amjad, Paris Stefanoudis; dateIdentified: 2022, 2023; identificationRemarks: Identified only from imagery; **Event:** samplingProtocol: Submersible OR Remotely Operated Vehicle OR Snorkel; **Record Level:** basisOfRecord: Human observation

### Crypthelia gen. indet. sp.

D5AF1800-1C8D-5B78-BCFE-7A4CE8DA7F79

#### Materials

- **Type status:** Other material. **Occurrence:** occurrenceID: 1ED3B82C-D15B-5BD6-B9F1-300C9FF2036A; **Taxon:** scientificName: *Crypthelia* sp.; kingdom: Animalia; phylum: Cnidaria; class: Hydrozoa; order: Anthoathecata; family: Stylasteridae; genus: Crypthelia; **Location:** waterBody: Indian Ocean; country: Maldives; locality: Laamu, Huvadhu; minimumDepthInMeters: 249; maximumDepthInMeters: 487; locationRemarks: Nekton Maldives Mission; **Identification:** identifiedBy: Farah Amjad, Paris Stefanoudis; dateIdentified: 2022, 2023; identificationRemarks: Identified only from imagery; **Event:** samplingProtocol: Submersible OR Remotely Operated Vehicle OR Snorkel; **Record Level:** basisOfRecord: Human observation

### Stylasteridae gen. indet. sp. 4

4F194AFB-671D-5209-A221-A9197008C670

#### Materials

- **Type status:** Other material. **Taxon:** scientificName: Stylasteridae sp. 4; kingdom: Animalia; phylum: Cnidaria; class: Hydrozoa; order: Anthoathecata; family: Stylasteridae; **Location:** waterBody: Indian Ocean; country: Maldives; locality: Vaavu, Laamu, Huvadhu, Addu, Fuvahmulah; minimumDepthInMeters: 116; maximumDepthInMeters: 490; locationRemarks: Nekton Maldives Mission; **Identification:** identifiedBy: Farah Amjad, Paris Stefanoudis; dateIdentified: 2022, 2023; identificationRemarks: Identified only from imagery; **Event:** samplingProtocol: Submersible OR Remotely Operated Vehicle OR Snorkel; **Record Level:** basisOfRecord: Human observation

### Stylasteridae gen. indet. sp. 5

B253EF0B-E533-538B-AB36-22D80D116157

#### Materials

- **Type status:** Other material. **Taxon:** scientificName: Stylasteridae sp. 5; kingdom: Animalia; phylum: Cnidaria; class: Hydrozoa; order: Anthoathecata; family: Stylasteridae; **Location:** waterBody: Indian Ocean; country: Maldives; locality: Huvadhu, Addu; minimumDepthInMeters: 248; maximumDepthInMeters: 251; locationRemarks: Nekton Maldives Mission; **Identification:** identifiedBy: Farah Amjad, Paris Stefanoudis; dateIdentified: 2022, 2023; identificationRemarks: Identified only from imagery; **Event:** samplingProtocol: Submersible OR Remotely Operated Vehicle OR Snorkel; **Record Level:** basisOfRecord: Human observation

### Stylasteridae gen. indet. sp. 6

048BE030-94F2-5D9D-B815-BC0B7A897576

#### Materials

- **Type status:** Other material. **Taxon:** scientificName: Stylasteridae sp. 6; kingdom: Animalia; phylum: Cnidaria; class: Hydrozoa; order: Anthoathecata; family: Stylasteridae; **Location:** waterBody: Indian Ocean; country: Maldives; locality: North Male’, Vaavu, Laamu; minimumDepthInMeters: 488; maximumDepthInMeters: 490; locationRemarks: Nekton Maldives Mission; **Identification:** identifiedBy: Farah Amjad, Paris Stefanoudis; dateIdentified: 2022, 2023; identificationRemarks: Identified only from imagery; **Event:** samplingProtocol: Submersible OR Remotely Operated Vehicle OR Snorkel; **Record Level:** basisOfRecord: Human observation

### Stylasteridae gen. indet. sp. 7

503F469C-9A56-5B23-8C9F-593ACC5D303C

#### Materials

- **Type status:** Other material. **Taxon:** scientificName: Stylasteridae sp. 7; kingdom: Animalia; phylum: Cnidaria; class: Hydrozoa; order: Anthoathecata; family: Stylasteridae; **Location:** waterBody: Indian Ocean; country: Maldives; locality: Vaavu, Huvadhu, Addu; minimumDepthInMeters: 248; maximumDepthInMeters: 489; locationRemarks: Nekton Maldives Mission; **Identification:** identifiedBy: Farah Amjad, Paris Stefanoudis; dateIdentified: 2022, 2023; identificationRemarks: Identified only from imagery; **Event:** samplingProtocol: Submersible OR Remotely Operated Vehicle OR Snorkel; **Record Level:** basisOfRecord: Human observation

### Stylasteridae gen. indet. sp. 8

D3DA95AF-7FFE-52D7-B2C3-4599696C0C4A

#### Materials

- **Type status:** Other material. **Taxon:** scientificName: Stylasteridae sp. 8; kingdom: Animalia; phylum: Cnidaria; class: Hydrozoa; order: Anthoathecata; family: Stylasteridae; **Location:** waterBody: Indian Ocean; country: Maldives; locality: Huvadhu, Addu; minimumDepthInMeters: 116; maximumDepthInMeters: 121; locationRemarks: Nekton Maldives Mission; **Identification:** identifiedBy: Farah Amjad, Paris Stefanoudis; dateIdentified: 2022, 2023; identificationRemarks: Identified only from imagery; **Event:** samplingProtocol: Submersible OR Remotely Operated Vehicle OR Snorkel; **Record Level:** basisOfRecord: Human observation

### Hydrozoa ord. indet. sp. 1

9A2793FA-2AEC-5A2E-B9D0-31F5B51F589D

#### Materials

- **Type status:** Other material. **Taxon:** scientificName: Hydrozoa sp. 1; kingdom: Animalia; phylum: Cnidaria; class: Hydrozoa; **Location:** waterBody: Indian Ocean; country: Maldives; locality: North Male’, Addu; minimumDepthInMeters: 10; maximumDepthInMeters: 10; locationRemarks: Nekton Maldives Mission; **Identification:** identifiedBy: Farah Amjad, Paris Stefanoudis; dateIdentified: 2022, 2023; identificationRemarks: Identified only from imagery; **Event:** samplingProtocol: Submersible OR Remotely Operated Vehicle OR Snorkel; **Record Level:** basisOfRecord: Human observation

### Hydrozoa ord. indet. sp. 4

FD835159-E865-5AB3-9A0F-230F7E7A72B0

#### Materials

- **Type status:** Other material. **Taxon:** scientificName: Hydrozoa sp. 4; kingdom: Animalia; phylum: Cnidaria; class: Hydrozoa; **Location:** waterBody: Indian Ocean; country: Maldives; locality: North Male’; minimumDepthInMeters: 489; maximumDepthInMeters: 489; locationRemarks: Nekton Maldives Mission; **Identification:** identifiedBy: Farah Amjad, Paris Stefanoudis; dateIdentified: 2022, 2023; identificationRemarks: Identified only from imagery; **Event:** samplingProtocol: Submersible OR Remotely Operated Vehicle OR Snorkel; **Record Level:** basisOfRecord: Human observation

### Hydrozoa ord. indet. sp. 5

B442E874-56F7-5680-A1DD-8809DF80F974

#### Materials

- **Type status:** Other material. **Taxon:** scientificName: Hydrozoa sp. 5; kingdom: Animalia; phylum: Cnidaria; class: Hydrozoa; scientificNameAuthorship: Owen, 1843; **Location:** waterBody: Indian Ocean; country: Maldives; locality: Vaavu, Addu; minimumDepthInMeters: 489; maximumDepthInMeters: 490; locationRemarks: Nekton Maldives Mission; **Identification:** identifiedBy: Farah Amjad, Paris Stefanoudis; dateIdentified: 2022, 2023; identificationRemarks: Identified only from imagery; **Event:** samplingProtocol: Submersible OR Remotely Operated Vehicle OR Snorkel; **Record Level:** basisOfRecord: Human observation

### Lyrocteis sp. indet.

D7162F89-2C1E-54D6-A125-9522B9485863

#### Materials

- **Type status:** Other material. **Taxon:** scientificName: *Lyrocteis* sp.; kingdom: Animalia; phylum: Ctenophora; class: Tentaculata; order: Platyctenida; family: Lyroctenidae; genus: Lyrocteis; **Location:** waterBody: Indian Ocean; country: Maldives; locality: TBD; minimumDepthInMeters: TBD; maximumDepthInMeters: TBD; locationRemarks: Nekton Maldives Mission; **Identification:** identifiedBy: Farah Amjad, Paris Stefanoudis; dateIdentified: 2022, 2023; identificationRemarks: Identified only from imagery; **Event:** samplingProtocol: Submersible OR Remotely Operated Vehicle OR Snorkel; **Record Level:** basisOfRecord: Human observation

### Tridacna sp. indet.

3010926A-0C64-582C-8081-88DDD2E055A2

#### Materials

- **Type status:** Other material. **Taxon:** scientificName: *Tridacna* sp.; kingdom: Animalia; phylum: Mollusca; class: Bivalvia; order: Cardiida; family: Cardiidae; genus: Tridacna; **Location:** waterBody: Indian Ocean; country: Maldives; locality: North Male’, Vaavu, Laamu, Addu; minimumDepthInMeters: 2; maximumDepthInMeters: 10; locationRemarks: Nekton Maldives Mission; **Identification:** identifiedBy: Farah Amjad, Paris Stefanoudis; dateIdentified: 2022, 2023; identificationRemarks: Identified only from imagery; **Event:** samplingProtocol: Submersible OR Remotely Operated Vehicle OR Snorkel; **Record Level:** basisOfRecord: Human observation

### Bivalvia ord. indet. sp. 1

AC6D300D-3AEF-57AC-AB47-226DEEB4B45F

#### Materials

- **Type status:** Other material. **Taxon:** scientificName: Bivalvia sp. 1; kingdom: Animalia; phylum: Mollusca; class: Bivalvia; **Location:** waterBody: Indian Ocean; country: Maldives; locality: North Male’, Laamu, Huvadhu, Fuvahmulah; minimumDepthInMeters: 120; maximumDepthInMeters: 487; locationRemarks: Nekton Maldives Mission; **Identification:** identifiedBy: Farah Amjad, Paris Stefanoudis; dateIdentified: 2022, 2023; identificationRemarks: Identified only from imagery; **Event:** samplingProtocol: Submersible OR Remotely Operated Vehicle OR Snorkel; **Record Level:** basisOfRecord: Human observation

### Bivalvia ord. indet. sp. 2

395464ED-5B82-564E-A301-43E52BCF82D6

#### Materials

- **Type status:** Other material. **Taxon:** scientificName: Bivalvia sp. 2; kingdom: Animalia; phylum: Mollusca; class: Bivalvia; **Location:** waterBody: Indian Ocean; country: Maldives; locality: Laamu, Huvadhu; minimumDepthInMeters: 485; maximumDepthInMeters: 489; locationRemarks: Nekton Maldives Mission; **Identification:** identifiedBy: Farah Amjad, Paris Stefanoudis; dateIdentified: 2022, 2023; identificationRemarks: Identified only from imagery; **Event:** samplingProtocol: Submersible OR Remotely Operated Vehicle OR Snorkel; **Record Level:** basisOfRecord: Human observation

### Bivalvia ord. indet. sp. 3

1DF05540-7D24-57FA-B675-223855DCD05E

#### Materials

- **Type status:** Other material. **Taxon:** scientificName: Bivalvia sp. 3; kingdom: Animalia; phylum: Mollusca; class: Bivalvia; **Location:** waterBody: Indian Ocean; country: Maldives; locality: Laamu, Addu; minimumDepthInMeters: 60; maximumDepthInMeters: 251; locationRemarks: Nekton Maldives Mission; **Identification:** identifiedBy: Farah Amjad, Paris Stefanoudis; dateIdentified: 2022, 2023; identificationRemarks: Identified only from imagery; **Event:** samplingProtocol: Submersible OR Remotely Operated Vehicle OR Snorkel; **Record Level:** basisOfRecord: Human observation

### Octopoda fam. indet. sp.

86169F75-E2B0-5E8A-80F0-7701FE6A34E1

#### Materials

- **Type status:** Other material. **Taxon:** scientificName: Octopoda sp.; kingdom: Animalia; phylum: Mollusca; class: Cephalopoda; order: Octopoda; **Location:** waterBody: Indian Ocean; country: Maldives; locality: Vaavu, Fuvahmulah; minimumDepthInMeters: 248; maximumDepthInMeters: 495; locationRemarks: Nekton Maldives Mission; **Identification:** identifiedBy: Farah Amjad, Paris Stefanoudis; dateIdentified: 2022, 2023; identificationRemarks: Identified only from imagery; **Event:** samplingProtocol: Submersible OR Remotely Operated Vehicle OR Snorkel; **Record Level:** basisOfRecord: Human observation

### Strombidae gen. indet. sp. 1

322E66A4-C117-59B8-80BF-7FAB81085981

#### Materials

- **Type status:** Other material. **Taxon:** scientificName: Strombidae sp. 1; kingdom: Animalia; phylum: Mollusca; class: Gastropoda; order: Littorinimorpha; family: Strombidae; **Location:** waterBody: Indian Ocean; country: Maldives; locality: Huvadhu Addu;; minimumDepthInMeters: 248; maximumDepthInMeters: 489; locationRemarks: Nekton Maldives Mission; **Identification:** identifiedBy: Farah Amjad, Paris Stefanoudis; dateIdentified: 2022, 2023; identificationRemarks: Identified only from imagery; **Event:** samplingProtocol: Submersible OR Remotely Operated Vehicle OR Snorkel; **Record Level:** basisOfRecord: Human observation

### Strombidae gen. indet. sp. 2

71FD0F66-A183-5E09-B95A-4628AD0666BA

#### Materials

- **Type status:** Other material. **Taxon:** scientificName: Strombidae sp. 2; kingdom: Animalia; phylum: Mollusca; class: Gastropoda; order: Littorinimorpha; family: Strombidae; **Location:** waterBody: Indian Ocean; country: Maldives; locality: Huvadhu; minimumDepthInMeters: 120; maximumDepthInMeters: 251; locationRemarks: Nekton Maldives Mission; **Identification:** identifiedBy: Farah Amjad, Paris Stefanoudis; dateIdentified: 2022, 2023; identificationRemarks: Identified only from imagery; **Event:** samplingProtocol: Submersible OR Remotely Operated Vehicle OR Snorkel; **Record Level:** basisOfRecord: Human observation

### Conoidea gen. indet. sp.

FCBAFE48-C27C-5503-B08A-5F491933B87E

#### Materials

- **Type status:** Other material. **Taxon:** scientificName: Conoidea sp.; kingdom: Animalia; phylum: Mollusca; class: Gastropoda; order: Neogastropoda; family: Conoidea; **Location:** waterBody: Indian Ocean; country: Maldives; locality: North Male’, Laamu, Huvadhu, Addu; minimumDepthInMeters: 248; maximumDepthInMeters: 491; locationRemarks: Nekton Maldives Mission; **Identification:** identifiedBy: Farah Amjad, Paris Stefanoudis; dateIdentified: 2022, 2023; identificationRemarks: Identified only from imagery; **Event:** samplingProtocol: Submersible OR Remotely Operated Vehicle OR Snorkel; **Record Level:** basisOfRecord: Human observation

### Drupella sp. indet.

A5A7DBA7-A667-562B-AEBB-25FF6BA8476B

#### Materials

- **Type status:** Other material. **Taxon:** scientificName: *Drupella* sp. indet.; kingdom: Animalia; phylum: Mollusca; class: Gastropoda; order: Neogastropoda; family: Muricidae; genus: Drupella; **Location:** waterBody: Indian Ocean; country: Maldives; locality: North Male’; minimumDepthInMeters: 10; maximumDepthInMeters: 10; locationRemarks: Nekton Maldives Mission; **Identification:** identifiedBy: Farah Amjad, Paris Stefanoudis; dateIdentified: 2022, 2023; identificationRemarks: Identified only from imagery; **Event:** samplingProtocol: Submersible OR Remotely Operated Vehicle OR Snorkel; **Record Level:** basisOfRecord: Human observation

### Polychaeta ord. indet. sp. 1

89B8ECAC-D43E-5450-A155-B78F77F3E833

#### Materials

- **Type status:** Other material. **Taxon:** scientificName: Polychaeta sp. 1; kingdom: Animalia; phylum: Annelida; class: Polychaeta; **Location:** waterBody: Indian Ocean; country: Maldives; locality: North Male’, Laamu, Addu; minimumDepthInMeters: 247; maximumDepthInMeters: 251; locationRemarks: Nekton Maldives Mission; **Identification:** identifiedBy: Farah Amjad, Paris Stefanoudis; dateIdentified: 2022, 2023; identificationRemarks: Identified only from imagery; **Event:** samplingProtocol: Submersible OR Remotely Operated Vehicle OR Snorkel; **Record Level:** basisOfRecord: Human observation

### Polychaeta ord. indet. sp. 2

2F9CAD47-3726-5776-A8C5-D97E7111E476

#### Materials

- **Type status:** Other material. **Taxon:** scientificName: Polychaeta sp. 2; kingdom: Animalia; phylum: Annelida; class: Polychaeta; **Location:** waterBody: Indian Ocean; country: Maldives; locality: North Male’, Laamu, Huvadhu, Addu; minimumDepthInMeters: 249; maximumDepthInMeters: 491; locationRemarks: Nekton Maldives Mission; **Identification:** identifiedBy: Farah Amjad, Paris Stefanoudis; dateIdentified: 2022, 2023; identificationRemarks: Identified only from imagery; **Event:** samplingProtocol: Submersible OR Remotely Operated Vehicle OR Snorkel; **Record Level:** basisOfRecord: Human observation

### Polychaeta ord. indet. sp. 3

6A6938C0-1A67-5A4A-97BD-15C57221EFAC

#### Materials

- **Type status:** Other material. **Taxon:** scientificName: Polychaeta sp. 3; kingdom: Animalia; phylum: Annelida; class: Polychaeta; **Location:** waterBody: Indian Ocean; country: Maldives; locality: Laamu; minimumDepthInMeters: 488; maximumDepthInMeters: 491; locationRemarks: Nekton Maldives Mission; **Identification:** identifiedBy: Farah Amjad, Paris Stefanoudis; dateIdentified: 2022, 2023; identificationRemarks: Identified only from imagery; **Event:** samplingProtocol: Submersible OR Remotely Operated Vehicle OR Snorkel; **Record Level:** basisOfRecord: Human observation

### Polychaeta ord. indet. sp. 4

6C1A5903-3691-5347-872E-DF9310A36322

#### Materials

- **Type status:** Other material. **Taxon:** scientificName: Polychaeta sp. 4; kingdom: Animalia; phylum: Annelida; class: Polychaeta; **Location:** waterBody: Indian Ocean; country: Maldives; locality: Laamu; minimumDepthInMeters: 485; maximumDepthInMeters: 491; locationRemarks: Nekton Maldives Mission; **Identification:** identifiedBy: Farah Amjad, Paris Stefanoudis; dateIdentified: 2022, 2023; identificationRemarks: Identified only from imagery; **Event:** samplingProtocol: Submersible OR Remotely Operated Vehicle OR Snorkel; **Record Level:** basisOfRecord: Human observation

### Polychaeta ord. indet. sp. 5

8A7EF3DD-B941-5DFC-B6F4-CA1E9E5F6A4D

#### Materials

- **Type status:** Other material. **Occurrence:** occurrenceID: DB931A3D-037A-5A5E-96F0-0057103577F7; **Taxon:** scientificName: Polychaeta sp. 5; kingdom: Animalia; phylum: Annelida; class: Polychaeta; **Location:** waterBody: Indian Ocean; country: Maldives; locality: North Male'; minimumDepthInMeters: 250; maximumDepthInMeters: 253; locationRemarks: Nekton Maldives Mission; **Identification:** identifiedBy: Farah Amjad, Paris Stefanoudis; dateIdentified: 2022, 2023; identificationRemarks: Identified only from imagery; **Event:** samplingProtocol: Submersible OR Remotely Operated Vehicle OR Snorkel; **Record Level:** basisOfRecord: Human observation

### Echiura ord. indet. sp.

669C7C29-3884-560F-87E1-59A3FEA04070

#### Materials

- **Type status:** Other material. **Taxon:** scientificName: Echiura sp.; kingdom: Animalia; phylum: Annelida; class: Polychaeta-Echiura; **Location:** waterBody: Indian Ocean; country: Maldives; locality: North Male’, Vaavu; minimumDepthInMeters: 247; maximumDepthInMeters: 490; locationRemarks: Nekton Maldives Mission; **Identification:** identifiedBy: Farah Amjad, Paris Stefanoudis; dateIdentified: 2022, 2023; identificationRemarks: Identified only from imagery; **Event:** samplingProtocol: Submersible OR Remotely Operated Vehicle OR Snorkel; **Record Level:** basisOfRecord: Human observation

### Galatheoidea gen. indet. sp.

78A04152-2FBA-5F6A-A13B-1703972FF29F

#### Materials

- **Type status:** Other material. **Taxon:** scientificName: Galatheoidea sp.; kingdom: Animalia; phylum: Arthropoda; class: Malacostraca; order: Decapoda; family: Galatheoidea; **Location:** waterBody: Indian Ocean; country: Maldives; locality: Fuvahmulah, Addu; minimumDepthInMeters: 248; maximumDepthInMeters: 249; locationRemarks: Nekton Maldives Mission; **Identification:** identifiedBy: Farah Amjad, Paris Stefanoudis; dateIdentified: 2022, 2023; identificationRemarks: Identified only from imagery; **Event:** samplingProtocol: Submersible OR Remotely Operated Vehicle OR Snorkel; **Record Level:** basisOfRecord: Human observation

### Chirostyloidea gen. indet. sp. 1

A80240F7-DD28-589B-9F6E-7B2BAABFE565

#### Materials

- **Type status:** Other material. **Taxon:** scientificName: Chirostyloidea sp. 1; kingdom: Animalia; phylum: Arthropoda; class: Malacostraca; order: Decapoda; family: Chirostyloidea; **Location:** waterBody: Indian Ocean; country: Maldives; locality: North Male’, Laamu; minimumDepthInMeters: 246; maximumDepthInMeters: 251; locationRemarks: Nekton Maldives Mission; **Identification:** identifiedBy: Farah Amjad, Paris Stefanoudis; dateIdentified: 2022, 2023; identificationRemarks: Identified only from imagery; **Event:** samplingProtocol: Submersible OR Remotely Operated Vehicle OR Snorkel; **Record Level:** basisOfRecord: Human observation

### Chirostyloidea gen. indet. sp. 2

FBF8DC2D-6429-5915-AA2F-1350B797101A

#### Materials

- **Type status:** Other material. **Taxon:** scientificName: Chirostyloidea sp. 2; kingdom: Animalia; phylum: Arthropoda; class: Malacostraca; order: Decapoda; family: Chirostyloidea; **Location:** waterBody: Indian Ocean; country: Maldives; locality: Vaavu; minimumDepthInMeters: 250; maximumDepthInMeters: 250; locationRemarks: Nekton Maldives Mission; **Identification:** identifiedBy: Farah Amjad, Paris Stefanoudis; dateIdentified: 2022, 2023; identificationRemarks: Identified only from imagery; **Event:** samplingProtocol: Submersible OR Remotely Operated Vehicle OR Snorkel; **Record Level:** basisOfRecord: Human observation

### Leucosiidae gen. indet. sp.

3374A436-2B58-53E3-AECB-678A0A5D335E

#### Materials

- **Type status:** Other material. **Taxon:** scientificName: Leucosiidae sp.; kingdom: Animalia; phylum: Arthropoda; class: Malacostraca; order: Decapoda; family: Leucosiidae; **Location:** waterBody: Indian Ocean; country: Maldives; locality: Vaavu; minimumDepthInMeters: 489; maximumDepthInMeters: 489; locationRemarks: Nekton Maldives Mission; **Identification:** identifiedBy: Farah Amjad, Paris Stefanoudis; dateIdentified: 2022, 2023; identificationRemarks: Identified only from imagery; **Event:** samplingProtocol: Submersible OR Remotely Operated Vehicle OR Snorkel; **Record Level:** basisOfRecord: Human observation

### Munidopsidae gen. indet. sp.

B8A4E04A-EDB0-5BBE-970B-C47983F1D496

#### Materials

- **Type status:** Other material. **Taxon:** scientificName: Munidopsidae sp.; kingdom: Animalia; phylum: Arthropoda; class: Malacostraca; order: Decapoda; family: Munidopsidae; **Location:** waterBody: Indian Ocean; country: Maldives; locality: Laamu, Vaavu, Huvadhu, North Male’, Addu; minimumDepthInMeters: 247; maximumDepthInMeters: 490; locationRemarks: Nekton Maldives Mission; **Identification:** identifiedBy: Farah Amjad, Paris Stefanoudis; dateIdentified: 2022, 2023; identificationRemarks: Identified only from imagery; **Event:** samplingProtocol: Submersible OR Remotely Operated Vehicle OR Snorkel; **Record Level:** basisOfRecord: Human observation

### Mithracidae gen. indet. sp.

FCD257A0-4DA4-58FF-965B-719CF8E4763A

#### Materials

- **Type status:** Other material. **Taxon:** scientificName: Mithracidae sp.; kingdom: Animalia; phylum: Arthropoda; class: Malacostraca; order: Decapoda; family: Mithracidae; **Location:** waterBody: Indian Ocean; country: Maldives; locality: North Male’; minimumDepthInMeters: 489; maximumDepthInMeters: 489; locationRemarks: Nekton Maldives Mission; **Identification:** identifiedBy: Farah Amjad, Paris Stefanoudis; dateIdentified: 2022, 2023; identificationRemarks: Identified only from imagery; **Event:** samplingProtocol: Submersible OR Remotely Operated Vehicle OR Snorkel; **Record Level:** basisOfRecord: Human observation

### Homolidae gen. indet. sp.

41CD9F17-9559-563E-9952-BD1DA632C164

#### Materials

- **Type status:** Other material. **Taxon:** scientificName: Homolidae sp.; kingdom: Animalia; phylum: Arthropoda; class: Malacostraca; order: Decapoda; family: Homolidae; **Location:** waterBody: Indian Ocean; country: Maldives; locality: Laamu; minimumDepthInMeters: 491; maximumDepthInMeters: 491; locationRemarks: Nekton Maldives Mission; **Identification:** identifiedBy: Farah Amjad, Paris Stefanoudis; dateIdentified: 2022, 2023; identificationRemarks: Identified only from imagery; **Event:** samplingProtocol: Submersible OR Remotely Operated Vehicle OR Snorkel; **Record Level:** basisOfRecord: Human observation

### Paguropsis confusa

Lemaitre, Rahayu & Komai, 2018

6E82AAE9-B941-519C-8B77-07542B43F22C

#### Materials

- **Type status:** Other material. **Taxon:** scientificName: *Paguropsisconfusa*; kingdom: Animalia; phylum: Arthropoda; class: Malacostraca; order: Decapoda; family: Paguroidea; genus: Paguropsis; scientificNameAuthorship: Lemaitre, Rahayu & Komai, 2018; **Location:** waterBody: Indian Ocean; country: Maldives; locality: North Male’; minimumDepthInMeters: 247; maximumDepthInMeters: 251; locationRemarks: Nekton Maldives Mission; **Identification:** identifiedBy: Farah Amjad, Paris Stefanoudis; dateIdentified: 2022, 2023; identificationRemarks: Identified only from imagery; **Event:** samplingProtocol: Submersible OR Remotely Operated Vehicle OR Snorkel; **Record Level:** basisOfRecord: Human observation

#### Notes

A blanket-hermit crab with a sub-rectangular-shaped body. Has specialised chelate legs similar to ice-block tongs in shape. Approximately ~ 7 cm in the longest dimension. Colouration white with orange patches (Fig. 229).

### Aristeidae gen. indet. sp.

256EDDD8-CFDE-5E74-A0E9-84FB47A34715

#### Materials

- **Type status:** Other material. **Taxon:** scientificName: Aristeidae sp.; kingdom: Animalia; phylum: Arthropoda; class: Malacostraca; order: Decapoda; family: Aristeidae; **Location:** waterBody: Indian Ocean; country: Maldives; locality: North Male’, Vaavu, Huvadhu; minimumDepthInMeters: 247; maximumDepthInMeters: 490; locationRemarks: Nekton Maldives Mission; **Identification:** identifiedBy: Farah Amjad, Paris Stefanoudis; dateIdentified: 2022, 2023; identificationRemarks: Identified only from imagery; **Event:** samplingProtocol: Submersible OR Remotely Operated Vehicle OR Snorkel; **Record Level:** basisOfRecord: Human observation

### Xanthidae gen. indet. sp.

3127CD7A-BBC1-57F1-98F8-2A1B332E6833

#### Materials

- **Type status:** Other material. **Taxon:** scientificName: Xanthidae sp.; kingdom: Animalia; phylum: Arthropoda; class: Malacostraca; order: Decapoda-Brachyura; family: Xanthidae; **Location:** waterBody: Indian Ocean; country: Maldives; locality: Huvadhu; minimumDepthInMeters: 490; maximumDepthInMeters: 491; locationRemarks: Nekton Maldives Mission; **Identification:** identifiedBy: Farah Amjad, Paris Stefanoudis; dateIdentified: 2022, 2023; identificationRemarks: Identified only from imagery; **Event:** samplingProtocol: Submersible OR Remotely Operated Vehicle OR Snorkel; **Record Level:** basisOfRecord: Human observation

### Calappidae gen. indet. sp.

6D6CCC25-C9ED-5A2D-8985-0FA00B5C034F

#### Materials

- **Type status:** Other material. **Taxon:** scientificName: Calappidae sp.; kingdom: Animalia; phylum: Arthropoda; class: Malacostraca; order: Decapoda-Brachyura; family: Calappidae; **Location:** waterBody: Indian Ocean; country: Maldives; locality: North Male’, Vaavu, Addu; minimumDepthInMeters: 60; maximumDepthInMeters: 250; locationRemarks: Nekton Maldives Mission; **Identification:** identifiedBy: Farah Amjad, Paris Stefanoudis; dateIdentified: 2022, 2023; identificationRemarks: Identified only from imagery; **Event:** samplingProtocol: Submersible OR Remotely Operated Vehicle OR Snorkel; **Record Level:** basisOfRecord: Human observation

### Geryonidae gen. indet. sp.

A04622D5-8CFE-5BCC-883C-03D309455665

#### Materials

- **Type status:** Other material. **Taxon:** scientificName: Geryonidae sp.; kingdom: Animalia; phylum: Arthropoda; class: Malacostraca; order: Decapoda-Brachyura; family: Geryonidae; **Location:** waterBody: Indian Ocean; country: Maldives; locality: Huvadhu; minimumDepthInMeters: 490; maximumDepthInMeters: 490; locationRemarks: Nekton Maldives Mission; **Identification:** identifiedBy: Farah Amjad, Paris Stefanoudis; dateIdentified: 2022, 2023; identificationRemarks: Identified only from imagery; **Event:** samplingProtocol: Submersible OR Remotely Operated Vehicle OR Snorkel; **Record Level:** basisOfRecord: Human observation

### Caridea

fam. indet. sp.

D5916DCF-1984-5B87-9A94-CFD036F296AB

#### Materials

- **Type status:** Other material. **Taxon:** scientificName: Caridea sp.; kingdom: Animalia; phylum: Arthropoda; class: Malacostraca; order: Decapoda-Caridea; **Location:** waterBody: Indian Ocean; country: Maldives; locality: Huvadhu; minimumDepthInMeters: 489; maximumDepthInMeters: 489; locationRemarks: Nekton Maldives Mission; **Identification:** identifiedBy: Farah Amjad, Paris Stefanoudis; dateIdentified: 2022, 2024; identificationRemarks: Identified only from imagery; **Event:** samplingProtocol: Submersible OR Remotely Operated Vehicle OR Snorkel; **Record Level:** basisOfRecord: Human observation

### Bryozoa clas. indet. sp. 1

8A8DDB9C-724D-59B2-8A86-03B84BC382B7

#### Materials

- **Type status:** Other material. **Taxon:** scientificName: Bryozoa sp. 1; kingdom: Animalia; phylum: Bryozoa; scientificNameAuthorship: Not documented; **Location:** waterBody: Indian Ocean; country: Maldives; locality: Vaavu, Adhu; minimumDepthInMeters: 60; maximumDepthInMeters: 250; locationRemarks: Nekton Maldives Mission; **Identification:** identifiedBy: Farah Amjad, Paris Stefanoudis; dateIdentified: 2022, 2023; identificationRemarks: Identified only from imagery; **Event:** samplingProtocol: Submersible OR Remotely Operated Vehicle OR Snorkel; **Record Level:** basisOfRecord: Human observation

### Cellaria sp. indet.

F1FAC01E-E1F8-5798-BD7A-787B70293AB8

#### Materials

- **Type status:** Other material. **Taxon:** scientificName: *Cellaria* sp.; kingdom: Animalia; phylum: Bryozoa; class: Gymnolaemata; order: Cheilostomatida; family: Cellariidae; genus: Cellaria; **Location:** waterBody: Indian Ocean; country: Maldives; locality: Laamu, Huvadhu; minimumDepthInMeters: 10; maximumDepthInMeters: 30; locationRemarks: Nekton Maldives Mission; **Identification:** identifiedBy: Farah Amjad, Paris Stefanoudis; dateIdentified: 2022, 2023; identificationRemarks: Identified only from imagery; **Event:** samplingProtocol: Submersible OR Remotely Operated Vehicle OR Snorkel; **Record Level:** basisOfRecord: Human observation

### Brisingidae gen. indet. sp.

A543BAA8-C6CE-56D4-A9F6-06D0FD07F533

#### Materials

- **Type status:** Other material. **Taxon:** scientificName: Brisingidae sp.; kingdom: Animalia; phylum: Echinodermata; class: Asteroidea; order: Brisingida; family: Brisingidae; **Location:** waterBody: Indian Ocean; country: Maldives; locality: Vaavu; minimumDepthInMeters: 250; maximumDepthInMeters: 251; locationRemarks: Nekton Maldives Mission; **Identification:** identifiedBy: Farah Amjad, Paris Stefanoudis, Christopher Mah; dateIdentified: 2022, 2023; identificationRemarks: Identified only from imagery; **Event:** samplingProtocol: Submersible OR Remotely Operated Vehicle OR Snorkel; **Record Level:** basisOfRecord: Human observation

### Sclerasterias sp. indet.

01D0AF26-BA12-56B4-9DD9-ABBC909119C0

#### Materials

- **Type status:** Other material. **Taxon:** scientificName: *Sclerasterias* sp.; kingdom: Animalia; phylum: Echinodermata; class: Asteroidea; order: Forcipulatida; family: Asteriidae; genus: Sclerasterias; **Location:** waterBody: Indian Ocean; country: Maldives; locality: Laamu, Fuvahmulah, Addu; minimumDepthInMeters: 60; maximumDepthInMeters: 488; locationRemarks: Nekton Maldives Mission; **Identification:** identifiedBy: Farah Amjad, Paris Stefanoudis, Christopher Mah; dateIdentified: 2022, 2023; identificationRemarks: Identified only from imagery; **Event:** samplingProtocol: Submersible OR Remotely Operated Vehicle OR Snorkel; **Record Level:** basisOfRecord: Human observation

### Persephonaster sp. indet.

E2DCB62F-6F13-50EE-9008-F1392C6054B0

#### Materials

- **Type status:** Other material. **Taxon:** scientificName: *Persephonaster* sp.; kingdom: Animalia; phylum: Echinodermata; class: Asteroidea; order: Paxillosida; family: Astropectinidae; genus: Persephonaster; **Location:** waterBody: Indian Ocean; country: Maldives; locality: Vaavu; minimumDepthInMeters: 488; maximumDepthInMeters: 494; locationRemarks: Nekton Maldives Mission; **Identification:** identifiedBy: Farah Amjad, Paris Stefanoudis, Christopher Mah; dateIdentified: 2022, 2024; identificationRemarks: Identified only from imagery; **Event:** samplingProtocol: Submersible OR Remotely Operated Vehicle OR Snorkel; **Record Level:** basisOfRecord: Human observation

### Astropectinidae gen. indet. sp. 1

CED3B3A6-B7C5-5225-9F4E-9A3D32EEDFB0

#### Materials

- **Type status:** Other material. **Taxon:** scientificName: Astropectinidae sp. 1; kingdom: Animalia; phylum: Echinodermata; class: Asteroidea; order: Paxillosida; family: Astropectinidae; **Location:** waterBody: Indian Ocean; country: Maldives; locality: Vaavu, Addu; minimumDepthInMeters: 248; maximumDepthInMeters: 250; locationRemarks: Nekton Maldives Mission; **Identification:** identifiedBy: Farah Amjad, Paris Stefanoudis, Christopher Mah; dateIdentified: 2022, 2023; identificationRemarks: Identified only from imagery; **Event:** samplingProtocol: Submersible OR Remotely Operated Vehicle OR Snorkel; **Record Level:** basisOfRecord: Human observation

### Astropectinidae gen. indet. sp. 2

1123D244-C926-57FF-BF8E-256D712F1007

#### Materials

- **Type status:** Other material. **Taxon:** scientificName: Astropectinidae sp. 2; kingdom: Animalia; phylum: Echinodermata; class: Asteroidea; order: Paxillosida; family: Astropectinidae; **Location:** waterBody: Indian Ocean; country: Maldives; locality: North Male’, Vaavu, Addu; minimumDepthInMeters: 248; maximumDepthInMeters: 255; locationRemarks: Nekton Maldives Mission; **Identification:** identifiedBy: Farah Amjad, Paris Stefanoudis, Christopher Mah; dateIdentified: 2022, 2023; identificationRemarks: Identified only from imagery; **Event:** samplingProtocol: Submersible OR Remotely Operated Vehicle OR Snorkel; **Record Level:** basisOfRecord: Human observation

### Echinaster luzonicus

(Gray, 1840)

67A03B40-915F-5CF5-B48E-0559F8C85760

#### Materials

- **Type status:** Other material. **Taxon:** scientificName: *Echinasterluzonicus*; kingdom: Animalia; phylum: Echinodermata; class: Asteroidea; order: Spinulosida; family: Echinasteridae; genus: Echinaster; scientificNameAuthorship: (Gray, 1840); **Location:** waterBody: Indian Ocean; country: Maldives; locality: Huvadhu; minimumDepthInMeters: 10; maximumDepthInMeters: 30; locationRemarks: Nekton Maldives Mission; **Identification:** identifiedBy: Farah Amjad, Paris Stefanoudis, Christopher Mah; dateIdentified: 2022, 2023; identificationRemarks: Identified only from imagery; **Event:** samplingProtocol: Submersible OR Remotely Operated Vehicle OR Snorkel; **Record Level:** basisOfRecord: Human observation

#### Notes

**Figure 242.**
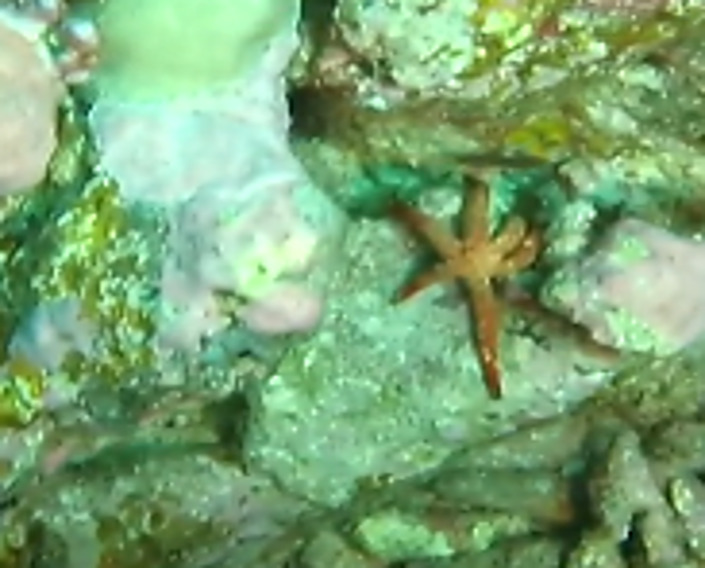
*Echinasterluzonicus*, Huvadhu, 10 m.

Slender tapered arms with pointy tip and inconspicuous central disc. Approximately 8 cm in the longest dimension. Colouration lighter shades of orange at the centre with gradual darkening towards the tips of the arms (Fig. 242).

### Anseropoda sp. indet.

276B6B00-1B30-567A-8BBC-EAF379FE5B48

#### Materials

- **Type status:** Other material. **Taxon:** scientificName: *Anseropoda* sp.; kingdom: Animalia; phylum: Echinodermata; class: Asteroidea; order: Valvatida; family: Asterinidae; genus: Anseropoda; **Location:** waterBody: Indian Ocean; country: Maldives; locality: North Male'; minimumDepthInMeters: 489; maximumDepthInMeters: 489; locationRemarks: Nekton Maldives Mission; **Identification:** identifiedBy: Farah Amjad, Paris Stefanoudis, Christopher Mah; dateIdentified: 2022, 2023; identificationRemarks: Identified only from imagery; **Event:** samplingProtocol: Submersible OR Remotely Operated Vehicle OR Snorkel; **Record Level:** basisOfRecord: Human observation

### Paranepanthia sp. indet.

D8A61D8C-3E64-56EC-A606-EEE2D6773CD1

#### Materials

- **Type status:** Other material. **Taxon:** scientificName: *Paranepanthia* sp.; kingdom: Animalia; phylum: Echinodermata; class: Asteroidea; order: Valvatida; family: Asterinidae; **Location:** waterBody: Indian Ocean; country: Maldives; locality: North Male’, Vaavu; minimumDepthInMeters: 247; maximumDepthInMeters: 489; locationRemarks: Nekton Maldives Mission; **Identification:** identifiedBy: Farah Amjad, Paris Stefanoudis, Christopher Mah; dateIdentified: 2022, 2023; identificationRemarks: Identified only from imagery; **Event:** samplingProtocol: Submersible OR Remotely Operated Vehicle OR Snorkel; **Record Level:** basisOfRecord: Human observation

### Tremaster mirabilis

Verrill, 1880

5BFCD22E-7494-5733-99D3-B60662BE3735

#### Materials

- **Type status:** Other material. **Taxon:** scientificName: *Tremastermirabilis*; kingdom: Animalia; phylum: Echinodermata; class: Asteroidea; order: Valvatida; family: Asterinidae; genus: Tremaster; scientificNameAuthorship: Verrill, 1880; **Location:** waterBody: Indian Ocean; country: Maldives; locality: Fuvahmulah; minimumDepthInMeters: 488; maximumDepthInMeters: 499; locationRemarks: Nekton Maldives Mission; **Identification:** identifiedBy: Farah Amjad, Paris Stefanoudis, Christopher Mah; dateIdentified: 2022, 2023; identificationRemarks: Identified only from imagery; **Event:** samplingProtocol: Submersible OR Remotely Operated Vehicle OR Snorkel; **Record Level:** basisOfRecord: Human observation

#### Notes

Outline pentagonal-shaped and inflated centrally with large central disc and thin body edges. Approximately 9 cm in the longest dimension. Collected specimen (Fig. 245).

### Asterinidae gen. indet. sp.

961F5702-EC0D-59E9-BBCF-5B2E9699042A

#### Materials

- **Type status:** Other material. **Taxon:** scientificName: Asterinidae sp.; kingdom: Animalia; phylum: Echinodermata; class: Asteroidea; order: Valvatida; family: Asterinidae; **Location:** waterBody: Indian Ocean; country: Maldives; locality: Vaavu, Laamu; minimumDepthInMeters: 248; maximumDepthInMeters: 251; locationRemarks: Nekton Maldives Mission; **Identification:** identifiedBy: Farah Amjad, Paris Stefanoudis, Christopher Mah; dateIdentified: 2022, 2023; identificationRemarks: Identified only from imagery; **Event:** samplingProtocol: Submersible OR Remotely Operated Vehicle OR Snorkel; **Record Level:** basisOfRecord: Human observation

### Mediaster sp. indet.

48AF9F44-98A0-58D1-B2CE-68700F235975

#### Materials

- **Type status:** Other material. **Taxon:** scientificName: *Mediaster* sp.; kingdom: Animalia; phylum: Echinodermata; class: Asteroidea; order: Valvatida; family: Goniasteridae; genus: Mediaster; **Location:** waterBody: Indian Ocean; country: Maldives; locality: North Male’, Laamu, Huvadhu; minimumDepthInMeters: 485; maximumDepthInMeters: 492; locationRemarks: Nekton Maldives Mission; **Identification:** identifiedBy: Farah Amjad, Paris Stefanoudis, Christopher Mah; dateIdentified: 2022, 2023; identificationRemarks: Identified only from imagery; **Event:** samplingProtocol: Submersible OR Remotely Operated Vehicle OR Snorkel; **Record Level:** basisOfRecord: Human observation

### Ceramaster sp. indet.

D8312C4B-DCF9-5AE4-92FD-A9EB8433EC2F

#### Materials

- **Type status:** Other material. **Taxon:** scientificName: *Ceramaster* sp.; kingdom: Animalia; phylum: Echinodermata; class: Asteroidea; order: Valvatida; family: Goniasteridae; genus: Ceramaster; scientificNameAuthorship: , 1899; **Location:** waterBody: Indian Ocean; country: Maldives; locality: Vaavu, Huvadhu; minimumDepthInMeters: 248; maximumDepthInMeters: 253; locationRemarks: Nekton Maldives Mission; **Identification:** identifiedBy: Farah Amjad, Paris Stefanoudis, Christopher Mah; dateIdentified: 2022, 2023; identificationRemarks: Identified only from imagery; **Event:** samplingProtocol: Submersible OR Remotely Operated Vehicle OR Snorkel; **Record Level:** basisOfRecord: Human observation

### Sphaeriodiscus sp. indet.

4BB4209C-3738-5953-912C-2CEB5B44A432

#### Materials

- **Type status:** Other material. **Taxon:** scientificName: *Sphaeriodiscus* sp.; kingdom: Animalia; phylum: Echinodermata; class: Asteroidea; order: Valvatida; family: Goniasteridae; genus: Sphaeriodiscus; **Location:** waterBody: Indian Ocean; country: Maldives; locality: TBC; minimumDepthInMeters: TBC; maximumDepthInMeters: TBC; locationRemarks: Nekton Maldives Mission; **Identification:** identifiedBy: Farah Amjad, Paris Stefanoudis, Christopher Mah; dateIdentified: 2022, 2023; identificationRemarks: Identified only from imagery; **Event:** samplingProtocol: Submersible OR Remotely Operated Vehicle OR Snorkel; **Record Level:** basisOfRecord: Human observation

### Nymphaster sp. indet.

8BEC85EF-AB51-5E6E-A7FC-F53E0B0FCB73

#### Materials

- **Type status:** Other material. **Taxon:** scientificName: *Nymphaster* sp.; kingdom: Animalia; phylum: Echinodermata; class: Asteroidea; order: Valvatida; family: Goniasteridae; genus: Nymphaster; **Location:** waterBody: Indian Ocean; country: Maldives; locality: Vaavu; minimumDepthInMeters: 489; maximumDepthInMeters: 489; locationRemarks: Nekton Maldives Mission; **Identification:** identifiedBy: Farah Amjad, Paris Stefanoudis, Christopher Mah; dateIdentified: 2022, 2023; identificationRemarks: Identified only from imagery; **Event:** samplingProtocol: Submersible OR Remotely Operated Vehicle OR Snorkel; **Record Level:** basisOfRecord: Human observation

### Goniasteridae gen. indet. sp. 4

6468E6F7-B772-5DFF-A132-DE426ECC5403

#### Materials

- **Type status:** Other material. **Taxon:** scientificName: Goniasteridae sp. 4; kingdom: Animalia; phylum: Echinodermata; class: Asteroidea; order: Valvatida; family: Goniasteridae; **Location:** waterBody: Indian Ocean; country: Maldives; locality: Huvadhu; minimumDepthInMeters: 489; maximumDepthInMeters: 489; locationRemarks: Nekton Maldives Mission; **Identification:** identifiedBy: Farah Amjad, Paris Stefanoudis, Christopher Mah; dateIdentified: 2022, 2023; identificationRemarks: Identified only from imagery; **Event:** samplingProtocol: Submersible OR Remotely Operated Vehicle OR Snorkel; **Record Level:** basisOfRecord: Human observation

### Fromia monilis

(Perrier, 1869)

98A2D184-C1C5-5B7E-9754-05992BF88B3C

#### Materials

- **Type status:** Other material. **Occurrence:** occurrenceID: D1A131B2-BF70-5FDA-9246-EE36F7AD26D8; **Taxon:** scientificName: *Fromiamonilis*; kingdom: Animalia; phylum: Echinodermata; class: Asteroidea; order: Valvatida; family: Goniasteridae; genus: Fromia; scientificNameAuthorship: (Perrier, 1869); **Location:** waterBody: Indian Ocean; country: Maldives; locality: North Male’, Vaavu, Addu; minimumDepthInMeters: 2; maximumDepthInMeters: 61; locationRemarks: Nekton Maldives Mission; **Identification:** identifiedBy: Farah Amjad, Paris Stefanoudis, Christopher Mah; dateIdentified: 2022, 2023; identificationRemarks: Identified only from imagery; **Event:** samplingProtocol: Submersible OR Remotely Operated Vehicle OR Snorkel; **Record Level:** basisOfRecord: Human observation

#### Notes

Five long tapered arms with small central disc. Marginal plates distinct and well developed with lighter cream colouration. Pattern on mid-line of arms in similar colour to the marginal plates. Approximately 7 cm in the longest dimension. Colouration of body and central disc bright to deep red tones (Fig. 252).

### Astrosarkus idipi

Mah, 2003

FDD74F4A-842F-519E-BC52-3ECD8F895DB3

#### Materials

- **Type status:** Other material. **Taxon:** scientificName: *Astrosarkusidipi*; kingdom: Animalia; phylum: Echinodermata; class: Asteroidea; order: Valvatida; family: Oreasteridae; genus: Astrosarkus; scientificNameAuthorship: Mah, 2003; **Location:** waterBody: Indian Ocean; country: Maldives; locality: Addu; minimumDepthInMeters: 83; maximumDepthInMeters: 91; locationRemarks: Nekton Maldives Mission; **Identification:** identifiedBy: Farah Amjad, Paris Stefanoudis, Christopher Mah; dateIdentified: 2022, 2023; identificationRemarks: Identified only from imagery; **Event:** samplingProtocol: Submersible OR Remotely Operated Vehicle OR Snorkel; **Record Level:** basisOfRecord: Human observation

#### Notes

Body heavily inflated with large central disc and short arms. Arms with rounded margins and more pointy at the tips. Approximately 32 cm in the longest dimension. Colouration in red tones and peach with patchy pattern throughout the surface. This is a colour variant of the species described in more detail in Mah 2023 (Fig. 253).

### Choriaster granulatus

Lütken, 1869

2160D2FC-F90E-56D2-A240-827F90D4B02C

#### Materials

- **Type status:** Other material. **Taxon:** scientificName: *Choriastergranulatus*; kingdom: Animalia; phylum: Echinodermata; class: Asteroidea; order: Valvatida; family: Oreasteridae; genus: Choriaster; scientificNameAuthorship: Lütken, 1869; **Location:** waterBody: Indian Ocean; country: Maldives; locality: Vaavu, Huvadhu, Fuvahmulah, Addu; minimumDepthInMeters: 26; maximumDepthInMeters: 60; locationRemarks: Nekton Maldives Mission; **Identification:** identifiedBy: Farah Amjad, Paris Stefanoudis, Christopher Mah; dateIdentified: 2022, 2023; identificationRemarks: Identified only from imagery; **Event:** samplingProtocol: Submersible OR Remotely Operated Vehicle OR Snorkel; **Record Level:** basisOfRecord: Human observation

#### Notes

Large inflated central disc with short compact and cylindrical arms. Arms rounded and cylindrical at the tips. Approximately 24 cm in the longest dimension. Colouration pink, orange and peach tones with distinctly lighter colouration tips of arms (Fig. 254).

### Culcita schmideliana

(Bruzelius, 1805)

B2D62EF6-CCE3-5EBF-B7F4-4BDF5182B0F9

#### Materials

- **Type status:** Other material. **Taxon:** scientificName: *Culcitaschmideliana*; kingdom: Animalia; phylum: Echinodermata; class: Asteroidea; order: Valvatida; family: Oreasteridae; genus: Culcita; scientificNameAuthorship: (Bruzelius, 1805); **Location:** waterBody: Indian Ocean; country: Maldives; locality: North Male’, Vaavu; minimumDepthInMeters: 2; maximumDepthInMeters: 5; locationRemarks: Nekton Maldives Mission; **Identification:** identifiedBy: Farah Amjad, Paris Stefanoudis, Christopher Mah; dateIdentified: 2022, 2023; identificationRemarks: Identified only from imagery; **Event:** samplingProtocol: Submersible OR Remotely Operated Vehicle OR Snorkel; **Record Level:** basisOfRecord: Human observation

#### Notes

**Figure 255.**
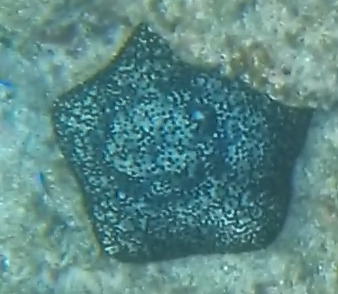
*Culcitaschmideliana*, Vaavu, 2 m.

Five short arms with a large central disc. Body shape pentagonal with a heavily inflated appearance. Pentagonal-shaped with minimal concaves on edge. Inflated body with compact arms and a large central disc. Spinelets evident. Approximately 20 cm in the longest dimension. Colouration appears blotchy with dark and light grey tones (Fig. 255).

### Linckia sp. indet.

1443CAB1-FB07-5F3B-9372-F6F2FA87DDED

#### Materials

- **Type status:** Other material. **Taxon:** scientificName: *Linckia* sp.; kingdom: Animalia; phylum: Echinodermata; class: Asteroidea; order: Valvatida; family: Ophidiasteridae; genus: Linckia; **Location:** waterBody: Indian Ocean; country: Maldives; locality: Vaavu, Huvadhu, Addu; minimumDepthInMeters: 10; maximumDepthInMeters: 62; locationRemarks: Nekton Maldives Mission; **Identification:** identifiedBy: Farah Amjad, Paris Stefanoudis, Christopher Mah; dateIdentified: 2022, 2023; identificationRemarks: Identified only from imagery; **Event:** samplingProtocol: Submersible OR Remotely Operated Vehicle OR Snorkel; **Record Level:** basisOfRecord: Human observation

### Asteroidea ord. indet. sp. 5

6B115C33-F3D0-5621-9F3D-C117475764F1

#### Materials

- **Type status:** Other material. **Taxon:** scientificName: Asteroidea sp. 5; kingdom: Animalia; phylum: Echinodermata; class: Asteroidea; **Location:** waterBody: Indian Ocean; country: Maldives; locality: Addu; minimumDepthInMeters: 491; maximumDepthInMeters: 491; locationRemarks: Nekton Maldives Mission; **Identification:** identifiedBy: Farah Amjad, Paris Stefanoudis, Christopher Mah; dateIdentified: 2022, 2023; identificationRemarks: Identified only from imagery; **Event:** samplingProtocol: Submersible OR Remotely Operated Vehicle OR Snorkel; **Record Level:** basisOfRecord: Human observation

### Asteroidea ord. indet. sp. 6

77A85E0C-BA89-579B-83F8-E3FACAA3D3ED

#### Materials

- **Type status:** Other material. **Taxon:** scientificName: Asteroidea sp. 6; kingdom: Animalia; phylum: Echinodermata; class: Asteroidea; **Location:** waterBody: Indian Ocean; country: Maldives; locality: Addu; minimumDepthInMeters: 248; maximumDepthInMeters: 250; locationRemarks: Nekton Maldives Mission; **Identification:** identifiedBy: Farah Amjad, Paris Stefanoudis, Christopher Mah; dateIdentified: 2022, 2023; identificationRemarks: Identified only from imagery; **Event:** samplingProtocol: Submersible OR Remotely Operated Vehicle OR Snorkel; **Record Level:** basisOfRecord: Human observation

### Ophiuroidea stet.

AC7960A6-A9E5-5C42-B984-892C2C2D66C0

#### Materials

- **Type status:** Other material. **Taxon:** scientificName: Ophiuroidea sp.; kingdom: Animalia; phylum: Echinodermata; class: Ophiuroidea; **Location:** waterBody: Indian Ocean; country: Maldives; locality: North Male’, Vaavu, Huvadhu, Addu; minimumDepthInMeters: 250; maximumDepthInMeters: 490; locationRemarks: Nekton Maldives Mission; **Identification:** identifiedBy: Farah Amjad, Paris Stefanoudis; dateIdentified: 2022, 2023; identificationRemarks: Identified only from imagery; **Event:** samplingProtocol: Submersible OR Remotely Operated Vehicle OR Snorkel; **Record Level:** basisOfRecord: Human observation

### Crinoidea ord. indet. sp. 1

FCEB8347-EE6A-57F6-9558-1C4A9DC242F8

#### Materials

- **Type status:** Other material. **Taxon:** scientificName: Crinoidea sp. 1; kingdom: Animalia; phylum: Echinodermata; class: Crinoidea; **Location:** waterBody: Indian Ocean; country: Maldives; locality: North Male’, Vaavu, Huvadhu, Addu; minimumDepthInMeters: 115; maximumDepthInMeters: 253; locationRemarks: Nekton Maldives Mission; **Identification:** identifiedBy: Farah Amjad, Paris Stefanoudis; dateIdentified: 2022, 2023; identificationRemarks: Identified only from imagery; **Event:** samplingProtocol: Submersible OR Remotely Operated Vehicle OR Snorkel; **Record Level:** basisOfRecord: Human observation

### Crinoidea ord. indet. sp. 2

87D86B83-6031-5C7E-8C94-6788D90EAF8F

Crinoidea ord. indet. sp. 2

#### Materials

- **Type status:** Other material. **Taxon:** scientificName: Crinoidea sp. 2; kingdom: Animalia; phylum: Echinodermata; class: Crinoidea; **Location:** waterBody: Indian Ocean; country: Maldives; locality: North Male’, Vaavu; minimumDepthInMeters: 489; maximumDepthInMeters: 489; locationRemarks: Nekton Maldives Mission; **Identification:** identifiedBy: Farah Amjad, Paris Stefanoudis; dateIdentified: 2022, 2023; identificationRemarks: Identified only from imagery; **Event:** samplingProtocol: Submersible OR Remotely Operated Vehicle OR Snorkel; **Record Level:** basisOfRecord: Human observation

### Crinoidea ord. indet. sp. 3

B28EDFDA-07B7-5FF3-B0E8-7532459F51FC

#### Materials

- **Type status:** Other material. **Taxon:** scientificName: Crinoidea sp. 3; kingdom: Animalia; phylum: Echinodermata; class: Crinoidea; **Location:** waterBody: Indian Ocean; country: Maldives; locality: North Male’, Huvadhu, Fuvahmulah; minimumDepthInMeters: 115; maximumDepthInMeters: 489; locationRemarks: Nekton Maldives Mission; **Identification:** identifiedBy: Farah Amjad, Paris Stefanoudis; dateIdentified: 2022, 2023; identificationRemarks: Identified only from imagery; **Event:** samplingProtocol: Submersible OR Remotely Operated Vehicle OR Snorkel; **Record Level:** basisOfRecord: Human observation

### Micropyga sp. indet.

74B7C56C-C0EA-5710-B5D3-3F78E19C1520

#### Materials

- **Type status:** Other material. **Taxon:** scientificName: *Micropyga* sp.; kingdom: Animalia; phylum: Echinodermata; class: Echinoidea; order: Micropygoida; family: Micropygidae; genus: Micropyga; **Location:** waterBody: Indian Ocean; country: Maldives; locality: Laamu, Huvadhu, Addu; minimumDepthInMeters: 246; maximumDepthInMeters: 490; locationRemarks: Nekton Maldives Mission; **Identification:** identifiedBy: Farah Amjad, Paris Stefanoudis; dateIdentified: 2022, 2023; identificationRemarks: Identified only from imagery; **Event:** samplingProtocol: Submersible OR Remotely Operated Vehicle OR Snorkel; **Record Level:** basisOfRecord: Human observation

### Cidaroida fam. indet. sp. 1

CFF199EE-2E78-5CEE-82CD-68F20E68D311

#### Materials

- **Type status:** Other material. **Taxon:** scientificName: Cidaroida sp. 1; kingdom: Animalia; phylum: Echinodermata; class: Echinoidea; order: Cidaroida; **Location:** waterBody: Indian Ocean; country: Maldives; locality: North Male’, Vaavu, Laamu, Huvadhu, Addu; minimumDepthInMeters: 115; maximumDepthInMeters: 490; locationRemarks: Nekton Maldives Mission; **Identification:** identifiedBy: Farah Amjad, Paris Stefanoudis; dateIdentified: 2022, 2023; identificationRemarks: Identified only from imagery; **Event:** samplingProtocol: Submersible OR Remotely Operated Vehicle OR Snorkel; **Record Level:** basisOfRecord: Human observation

### Cidaroida fam. indet. sp. 2

93188898-F247-562E-A542-0CED47FB98DC

#### Materials

- **Type status:** Other material. **Taxon:** scientificName: Cidaroida sp. 2; kingdom: Animalia; phylum: Echinodermata; class: Echinoidea; order: Cidaroida; **Location:** waterBody: Indian Ocean; country: Maldives; locality: Vaavu, Fuvahmulah; minimumDepthInMeters: 250; maximumDepthInMeters: 253; locationRemarks: Nekton Maldives Mission; **Identification:** identifiedBy: Farah Amjad, Paris Stefanoudis; dateIdentified: 2022, 2023; identificationRemarks: Identified only from imagery; **Event:** samplingProtocol: Submersible OR Remotely Operated Vehicle OR Snorkel; **Record Level:** basisOfRecord: Human observation

### Clypeaster sp. indet.

4AE5A99D-8069-5054-8575-0035866E2CAC

#### Materials

- **Type status:** Other material. **Taxon:** scientificName: *Clypeaster* sp.; kingdom: Animalia; phylum: Echinodermata; class: Echinoidea; order: Clypeasteroida; family: Clypeasteridae; genus: Clypeaster; **Location:** waterBody: Indian Ocean; country: Maldives; locality: Vaavu, Addu; minimumDepthInMeters: 26; maximumDepthInMeters: 251; locationRemarks: Nekton Maldives Mission; **Identification:** identifiedBy: Farah Amjad, Paris Stefanoudis; dateIdentified: 2022, 2023; identificationRemarks: Identified only from imagery; **Event:** samplingProtocol: Submersible OR Remotely Operated Vehicle OR Snorkel; **Record Level:** basisOfRecord: Human observation

### Echinothrix diadema

(Linnaeus, 1758)

60474D88-AA78-5818-A921-48213111C2EA

#### Materials

- **Type status:** Other material. **Taxon:** scientificName: *Echinothrixdiadema*; kingdom: Animalia; phylum: Echinodermata; class: Echinoidea; order: Diadematoida; family: Diadematidae; genus: Echinothrix; scientificNameAuthorship: (Linnaeus, 1758); **Location:** waterBody: Indian Ocean; country: Maldives; locality: Laamu; minimumDepthInMeters: 2; maximumDepthInMeters: 5; locationRemarks: Nekton Maldives Mission; **Identification:** identifiedBy: Farah Amjad, Paris Stefanoudis; dateIdentified: 2022, 2023; identificationRemarks: Identified only from imagery; **Event:** samplingProtocol: Submersible OR Remotely Operated Vehicle OR Snorkel; **Record Level:** basisOfRecord: Human observation

#### Notes

**Figure 267.**
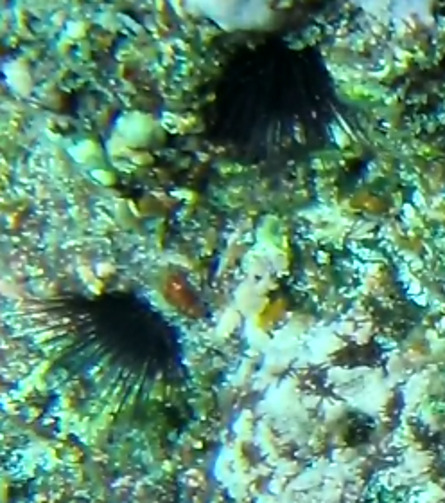
*Echinothrixdiadema*, Laamu, 2 m.

Small test size compared to spine length, with numerous fine spines throughout the aboral surface. Spines and test appear dark, almost black in colour. *Diadema* spp. look similar but tend to have longer spines relative to the test (Fig. 267).

### Echinoidea ord. indet. sp. 1

FF564C15-071D-5745-BFD6-449C23B058EA

#### Materials

- **Type status:** Other material. **Taxon:** scientificName: Echinoidea sp. 1; kingdom: Animalia; phylum: Echinodermata; class: Echinoidea; **Location:** waterBody: Indian Ocean; country: Maldives; locality: Vaavu, Huvadhu, Addu; minimumDepthInMeters: 248; maximumDepthInMeters: 490; locationRemarks: Nekton Maldives Mission; **Identification:** identifiedBy: Farah Amjad, Paris Stefanoudis; dateIdentified: 2022, 2023; identificationRemarks: Identified only from imagery; **Event:** samplingProtocol: Submersible OR Remotely Operated Vehicle OR Snorkel; **Record Level:** basisOfRecord: Human observation

### Echinoidea ord. indet. sp. 2

D8C1EB1B-C8BB-514D-9109-AABE8779E79D

#### Materials

- **Type status:** Other material. **Taxon:** scientificName: Echinoidea sp. 2; kingdom: Animalia; phylum: Echinodermata; class: Echinoidea; **Location:** waterBody: Indian Ocean; country: Maldives; locality: Laamu; minimumDepthInMeters: 488; maximumDepthInMeters: 490; locationRemarks: Nekton Maldives Mission; **Identification:** identifiedBy: Farah Amjad, Paris Stefanoudis; dateIdentified: 2022, 2023; identificationRemarks: Identified only from imagery; **Event:** samplingProtocol: Submersible OR Remotely Operated Vehicle OR Snorkel; **Record Level:** basisOfRecord: Human observation

### Spatangoida fam. indet. sp.

3F809423-44F8-5896-B53F-69C0031B1B87

#### Materials

- **Type status:** Other material. **Taxon:** scientificName: Spatangoida sp.; kingdom: Animalia; phylum: Echinodermata; class: Echinoidea; order: Spatangoida; **Location:** waterBody: Indian Ocean; country: Maldives; locality: North Male’; minimumDepthInMeters: 115; maximumDepthInMeters: 124; locationRemarks: Nekton Maldives Mission; **Identification:** identifiedBy: Farah Amjad, Paris Stefanoudis, Christopher Mah; dateIdentified: 2022, 2023; identificationRemarks: Identified only from imagery; **Event:** samplingProtocol: Submersible OR Remotely Operated Vehicle OR Snorkel; **Record Level:** basisOfRecord: Human observation

### Holothuria atra

Jaeger, 1833

DC525516-CE5D-572C-9970-B6B36747BB9A

#### Materials

- **Type status:** Other material. **Taxon:** scientificName: *Holothuriaatra*; kingdom: Animalia; phylum: Echinodermata; class: Holothuroidea; order: Holothuriida; family: Holothuriidae; genus: Holothuria; scientificNameAuthorship: Jaeger, 1833; **Location:** waterBody: Indian Ocean; country: Maldives; locality: Vaavu, Laamu, Addu; minimumDepthInMeters: 2; maximumDepthInMeters: 30; locationRemarks: Nekton Maldives Mission; **Identification:** identifiedBy: Farah Amjad, Paris Stefanoudis; dateIdentified: 2022, 2023; identificationRemarks: Identified only from imagery; **Event:** samplingProtocol: Submersible OR Remotely Operated Vehicle OR Snorkel; **Record Level:** basisOfRecord: Human observation

#### Notes

Cylindrical body with smooth surface, sometimes covered in sediment. Some individuals have a more stout body shape. Black and dark green in colour. Approximately 36 cm long (Fig. 271).

### Holothuria edulis

Lesson, 1830

43CE17A8-524B-537C-BB48-2C6D20E90EE4

#### Materials

- **Type status:** Other material. **Taxon:** scientificName: *Holothuriaedulis*; kingdom: Animalia; phylum: Echinodermata; class: Holothuroidea; order: Holothuriida; family: Holothuriidae; genus: Holothuria; scientificNameAuthorship: Lesson, 1830; **Location:** waterBody: Indian Ocean; country: Maldives; locality: Laamu; minimumDepthInMeters: 57; maximumDepthInMeters: 61; locationRemarks: Nekton Maldives Mission; **Identification:** identifiedBy: Farah Amjad, Paris Stefanoudis; dateIdentified: 2022, 2023; identificationRemarks: Identified only from imagery; **Event:** samplingProtocol: Submersible OR Remotely Operated Vehicle OR Snorkel; **Record Level:** basisOfRecord: Human observation

#### Notes

**Figure 272.**
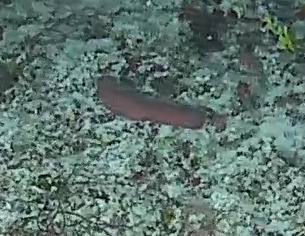
*Holothuriaedulis*, Laamu, 60 m.

Cylindrical elongated shape, surface appears smooth. Approximately 23 cm long. Pink with grey and black in colour (Fig. 272).

### Pearsonothuria graeffei

(Semper, 1868)

20B55C7F-C90F-5DF2-8A6C-C7B3800F7ED2

#### Materials

- **Type status:** Other material. **Occurrence:** occurrenceID: 659AFA28-F272-52E1-89A6-A4707F2258F4; **Taxon:** scientificName: *Pearsonothuriagraeffei*; kingdom: Animalia; phylum: Echinodermata; class: Holothuroidea; order: Holothuriida; family: Holothuriidae; genus: Pearsonothuria; scientificNameAuthorship: (Semper, 1868); **Location:** waterBody: Indian Ocean; country: Maldives; locality: North Male’, Addu; minimumDepthInMeters: 10; maximumDepthInMeters: 10; locationRemarks: Nekton Maldives Mission; **Identification:** identifiedBy: Farah Amjad, Paris Stefanoudis; dateIdentified: 2022, 2023; identificationRemarks: Identified only from imagery; **Event:** samplingProtocol: Submersible OR Remotely Operated Vehicle OR Snorkel; **Record Level:** basisOfRecord: Human observation

#### Notes

**Figure 273.**
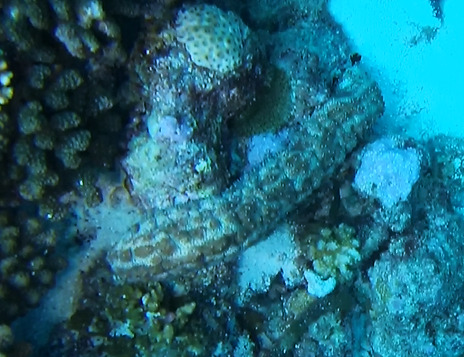
*Pearsonothuriagraeffei*, North Male’, ~ 10 m.

Elongated cylindrical body with bumpy papillae present throughout the surface. Approximately 31 cm long. Feeding tentacles extended from the body. Light brown to cream in colour with dark brown patches and black markings on the body (Fig. 273).

### Holothuroidea ord. indet. sp. 3

AB9D0C88-B632-50A7-8323-4EFD910F1D4D

#### Materials

- **Type status:** Other material. **Taxon:** scientificName: Holothuroidea sp. 3; kingdom: Animalia; phylum: Echinodermata; class: Holothuroidea; **Location:** waterBody: Indian Ocean; country: Maldives; locality: Vaavu, Huvadhu; minimumDepthInMeters: 247; maximumDepthInMeters: 489; locationRemarks: Nekton Maldives Mission; **Identification:** identifiedBy: Farah Amjad, Paris Stefanoudis; dateIdentified: 2022, 2023; identificationRemarks: Identified only from imagery; **Event:** samplingProtocol: Submersible OR Remotely Operated Vehicle OR Snorkel; **Record Level:** basisOfRecord: Human observation

### Holothuroidea ord. indet. sp. 4

03559D72-0901-5FD8-BD40-9AC54CB22769

#### Materials

- **Type status:** Other material. **Taxon:** scientificName: Holothuroidea sp. 4; kingdom: Animalia; phylum: Echinodermata; class: Holothuroidea; **Location:** waterBody: Indian Ocean; country: Maldives; locality: North Male’, Vaavu; minimumDepthInMeters: 233; maximumDepthInMeters: 490; locationRemarks: Nekton Maldives Mission; **Identification:** identifiedBy: Farah Amjad, Paris Stefanoudis; dateIdentified: 2022, 2023; identificationRemarks: Identified only from imagery; **Event:** samplingProtocol: Submersible OR Remotely Operated Vehicle OR Snorkel; **Record Level:** basisOfRecord: Human observation

### Holothuroidea ord. indet. sp. 5

50AFA789-01EC-5466-BA67-6B7D39E70C7B

#### Materials

- **Type status:** Other material. **Taxon:** scientificName: Holothuroidea sp. 5; kingdom: Animalia; phylum: Echinodermata; class: Holothuroidea; **Location:** waterBody: Indian Ocean; country: Maldives; locality: Vaavu; minimumDepthInMeters: 496; maximumDepthInMeters: 496; locationRemarks: Nekton Maldives Mission; **Identification:** identifiedBy: Farah Amjad, Paris Stefanoudis; dateIdentified: 2022, 2023; identificationRemarks: Identified only from imagery; **Event:** samplingProtocol: Submersible OR Remotely Operated Vehicle OR Snorkel; **Record Level:** basisOfRecord: Human observation

### Holothuroidea ord. indet. sp. 6

6C9DB984-5889-59CE-9019-C4F9C7F4D57F

#### Materials

- **Type status:** Other material. **Taxon:** scientificName: Holothuroidea sp. 6; kingdom: Animalia; phylum: Echinodermata; class: Holothuroidea; **Location:** waterBody: Indian Ocean; country: Maldives; locality: North Male’, Vaavu; minimumDepthInMeters: 250; maximumDepthInMeters: 489; locationRemarks: Nekton Maldives Mission; **Identification:** identifiedBy: Farah Amjad, Paris Stefanoudis; dateIdentified: 2022, 2023; identificationRemarks: Identified only from imagery; **Event:** samplingProtocol: Submersible OR Remotely Operated Vehicle OR Snorkel; **Record Level:** basisOfRecord: Human observation

### Holothuroidea ord. indet. sp. 7

D97102BD-F528-5E01-BD82-F13B738BC78B

#### Materials

- **Type status:** Other material. **Taxon:** scientificName: Holothuroidea sp. 7; kingdom: Animalia; phylum: Echinodermata; class: Holothuroidea; **Location:** waterBody: Indian Ocean; country: Maldives; locality: North Male’; minimumDepthInMeters: 489; maximumDepthInMeters: 489; locationRemarks: Nekton Maldives Mission; **Identification:** identifiedBy: Farah Amjad, Paris Stefanoudis; dateIdentified: 2022, 2023; identificationRemarks: Identified only from imagery; **Event:** samplingProtocol: Submersible OR Remotely Operated Vehicle OR Snorkel; **Record Level:** basisOfRecord: Human observation

### Holothuroidea ord. indet. sp. 8

F4ECA26B-8054-5028-9D92-42A5489EB292

#### Materials

- **Type status:** Other material. **Taxon:** scientificName: Holothuroidea sp. 8; kingdom: Animalia; phylum: Echinodermata; class: Holothuroidea; **Location:** waterBody: Indian Ocean; country: Maldives; locality: Vaavu; minimumDepthInMeters: 489; maximumDepthInMeters: 489; locationRemarks: Nekton Maldives Mission; **Identification:** identifiedBy: Farah Amjad, Paris Stefanoudis; dateIdentified: 2022, 2023; identificationRemarks: Identified only from imagery; **Event:** samplingProtocol: Submersible OR Remotely Operated Vehicle OR Snorkel; **Record Level:** basisOfRecord: Human observation

### Holothuroidea ord. indet. sp. 9

2045CA5E-CC5C-511A-9080-FCFAB58EFE97

#### Materials

- **Type status:** Other material. **Taxon:** scientificName: Holothuroidea sp. 9; kingdom: Animalia; phylum: Echinodermata; class: Holothuroidea; **Location:** waterBody: Indian Ocean; country: Maldives; locality: Fuvahmulah; minimumDepthInMeters: 489; maximumDepthInMeters: 492; locationRemarks: Nekton Maldives Mission; **Identification:** identifiedBy: Farah Amjad, Paris Stefanoudis; dateIdentified: 2022, 2023; identificationRemarks: Identified only from imagery; **Event:** samplingProtocol: Submersible OR Remotely Operated Vehicle OR Snorkel; **Record Level:** basisOfRecord: Human observation

### Didemnum molle

(Herdman, 1886)

DBAEFBA9-9078-53AD-89D4-34D98248B378

#### Materials

- **Type status:** Other material. **Taxon:** scientificName: *Didemnummolle*; kingdom: Animalia; phylum: Chordata-Tunicata; class: Ascidiacea; order: Aplousobranchia; family: Didemnidae; genus: Didemnum; scientificNameAuthorship: (Herdman, 1886); **Location:** waterBody: Indian Ocean; country: Maldives; locality: Vaavu; minimumDepthInMeters: 2; maximumDepthInMeters: 489; locationRemarks: Nekton Maldives Mission; **Identification:** identifiedBy: Farah Amjad, Paris Stefanoudis; dateIdentified: 2022, 2023; identificationRemarks: Identified only from imagery; **Event:** samplingProtocol: Submersible OR Remotely Operated Vehicle OR Snorkel; **Record Level:** basisOfRecord: Human observation

#### Notes

Forms urn-shaped structures with linked zooids that are anchored to the substratum. Approximately 14 cm in the longest dimension. Contains cyanobacteria in its tissue. Similar to *Haliclona* sp. (Fig. 281).

### Ascidiacea ord. indet. sp. 1

7934DE47-D907-57F0-BED4-190C882909C7

#### Materials

- **Type status:** Other material. **Taxon:** scientificName: Ascidiacea sp. 1; kingdom: Animalia; phylum: Chordata-Tunicata; class: Ascidiacea; **Location:** waterBody: Indian Ocean; country: Maldives; locality: Fuvahmulah; minimumDepthInMeters: 120; maximumDepthInMeters: 121; locationRemarks: Nekton Maldives Mission; **Identification:** identifiedBy: Farah Amjad, Paris Stefanoudis; dateIdentified: 2022, 2023; identificationRemarks: Identified only from imagery; **Event:** samplingProtocol: Submersible OR Remotely Operated Vehicle OR Snorkel; **Record Level:** basisOfRecord: Human observation

### Ascidiacea ord. indet. sp. 2

2113BA16-FBB2-5C2B-B7B1-D8026E024D3B

#### Materials

- **Type status:** Other material. **Taxon:** scientificName: Ascidiacea sp. 2; kingdom: Animalia; phylum: Chordata-Tunicata; class: Ascidiacea; **Location:** waterBody: Indian Ocean; country: Maldives; locality: Addu; minimumDepthInMeters: 10; maximumDepthInMeters: 10; locationRemarks: Nekton Maldives Mission; **Identification:** identifiedBy: Farah Amjad, Paris Stefanoudis; dateIdentified: 2022, 2023; identificationRemarks: Identified only from imagery; **Event:** samplingProtocol: Submersible OR Remotely Operated Vehicle OR Snorkel; **Record Level:** basisOfRecord: Human observation

### Unknown sp. indet. 1

1685FCB6-A9F3-5F3B-A264-6E44A038BDB3

#### Materials

- **Type status:** Other material. **Taxon:** scientificName: Unknown sp. indet. 1; **Location:** waterBody: Indian Ocean; country: Maldives; locality: North Male’, Vaavu, Laamu, Huvadhu, Addu; minimumDepthInMeters: 53; maximumDepthInMeters: 124; locationRemarks: Nekton Maldives Mission; **Identification:** identifiedBy: Farah Amjad, Paris Stefanoudis; dateIdentified: 2022, 2023; identificationRemarks: Identified only from imagery; **Event:** samplingProtocol: Submersible OR Remotely Operated Vehicle OR Snorkel; **Record Level:** basisOfRecord: Human observation

## Figures and Tables

**Figure 2.**
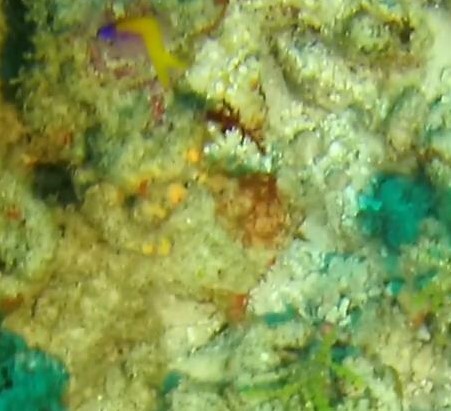
Cyanobacteria stet. sp. 1, Laamu, 30 m.

**Figure 3.**
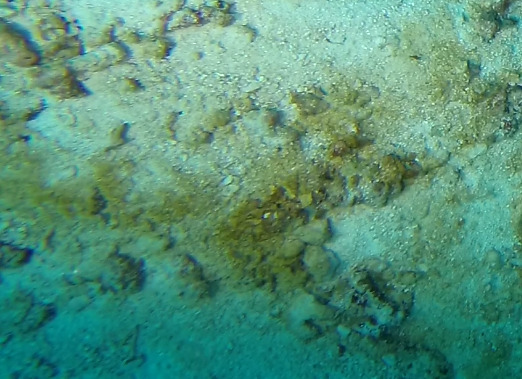
Cyanobacteria stet. sp. 2, North Male’, 30 m.

**Figure 4a.**
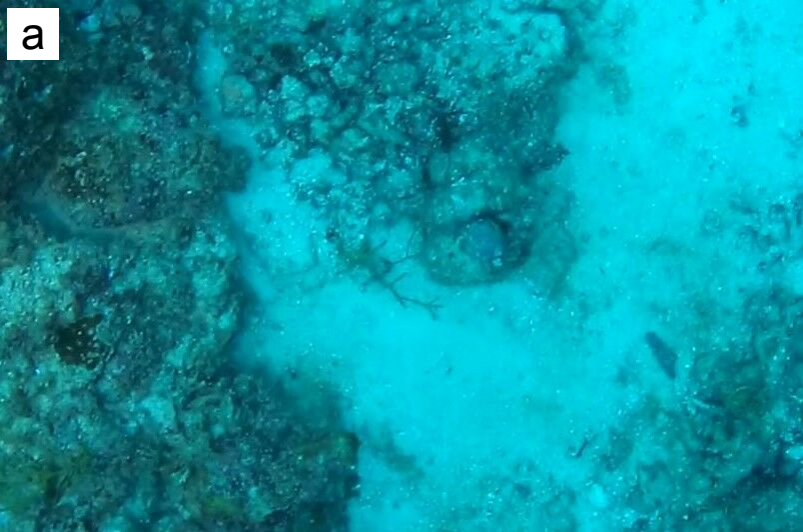
North Male’, 30 m;

**Figure 4b.**
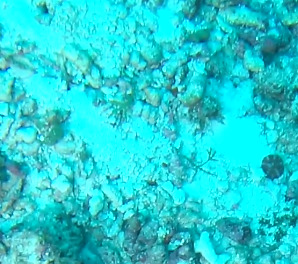
North Male’, 30 m.

**Figure 6a.**
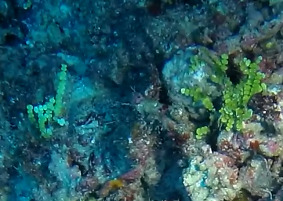
Vaavu, 30 m;

**Figure 6b.**
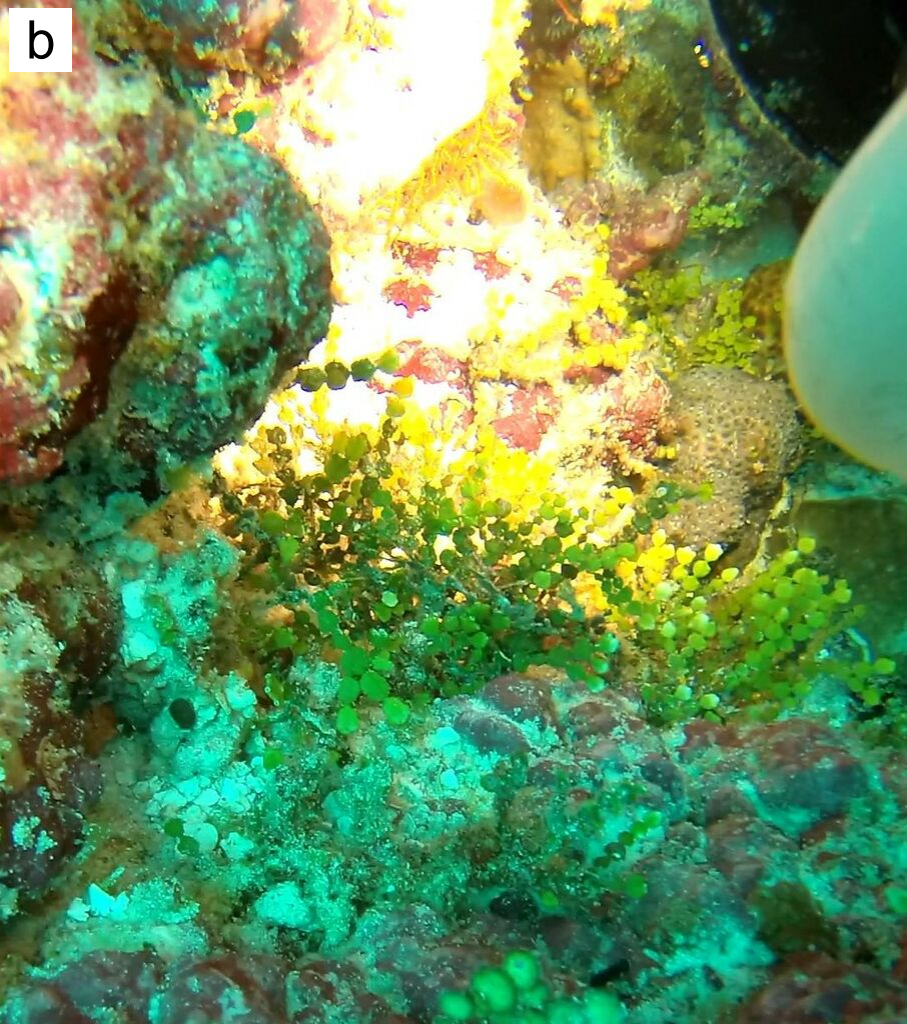
Laamu, 30 m.

**Figure 7a.**
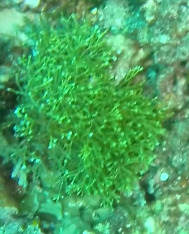
North Male’, 10 m;

**Figure 7b.**
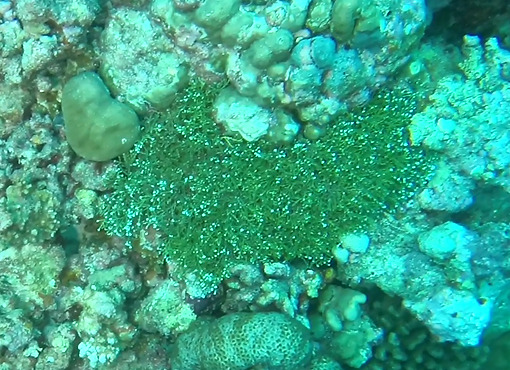
North Male’, 10 m.

**Figure 8a.**
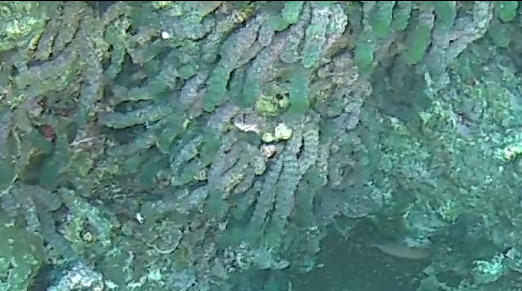
Addu, 30 m;

**Figure 8b.**
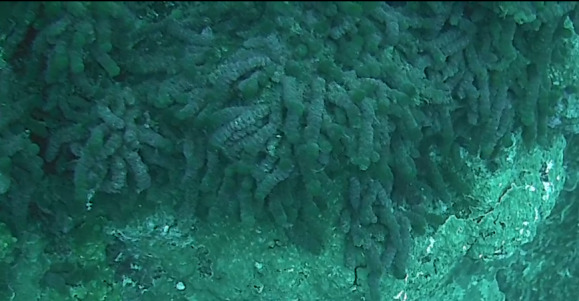
Addu, 30 m.

**Figure 9a.**
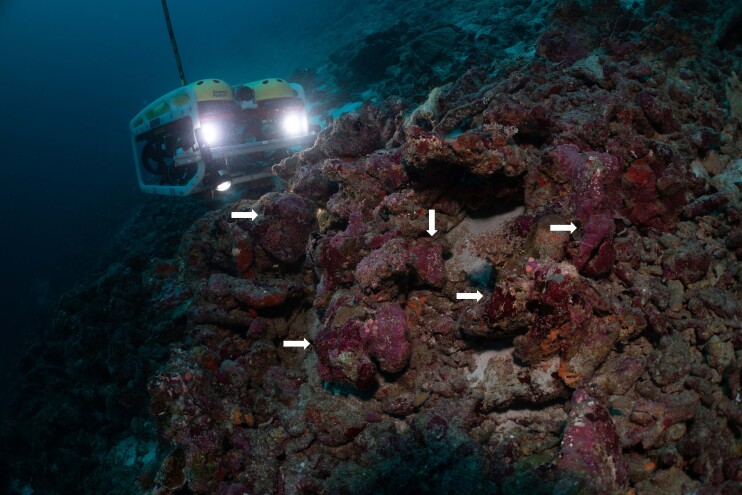
Huvadhu, 10-30 m;

**Figure 9b.**
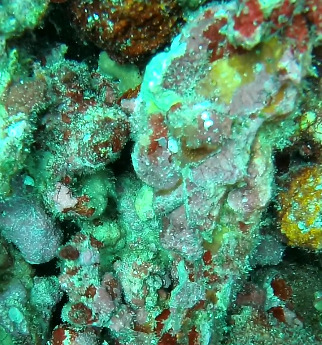
North Male’, 10 m;

**Figure 9c.**
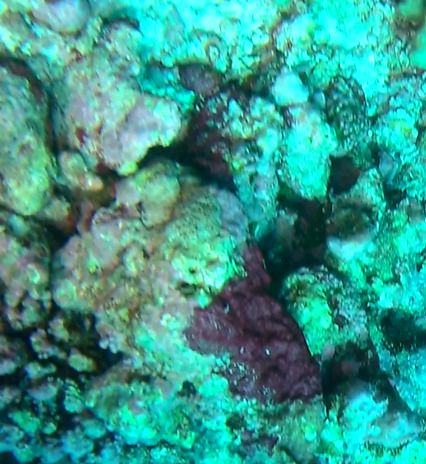
Laamu, 10 m.

**Figure 10a.**
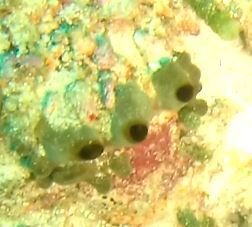
Huvadhu, 30 m;

**Figure 10b.**
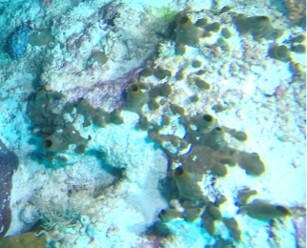
Huvadhu, 30 m.

**Figure 11a.**
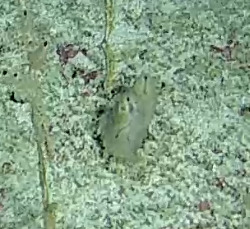
North Male’, 120 m;

**Figure 11b.**
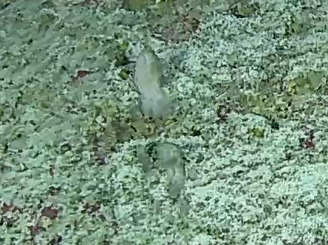
North Male’, 120 m.

**Figure 13a.**
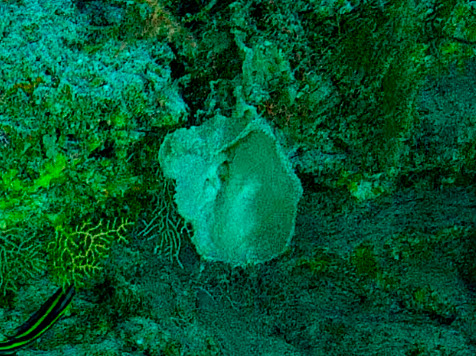
Fuvahmulah, 120 m;

**Figure 13b.**
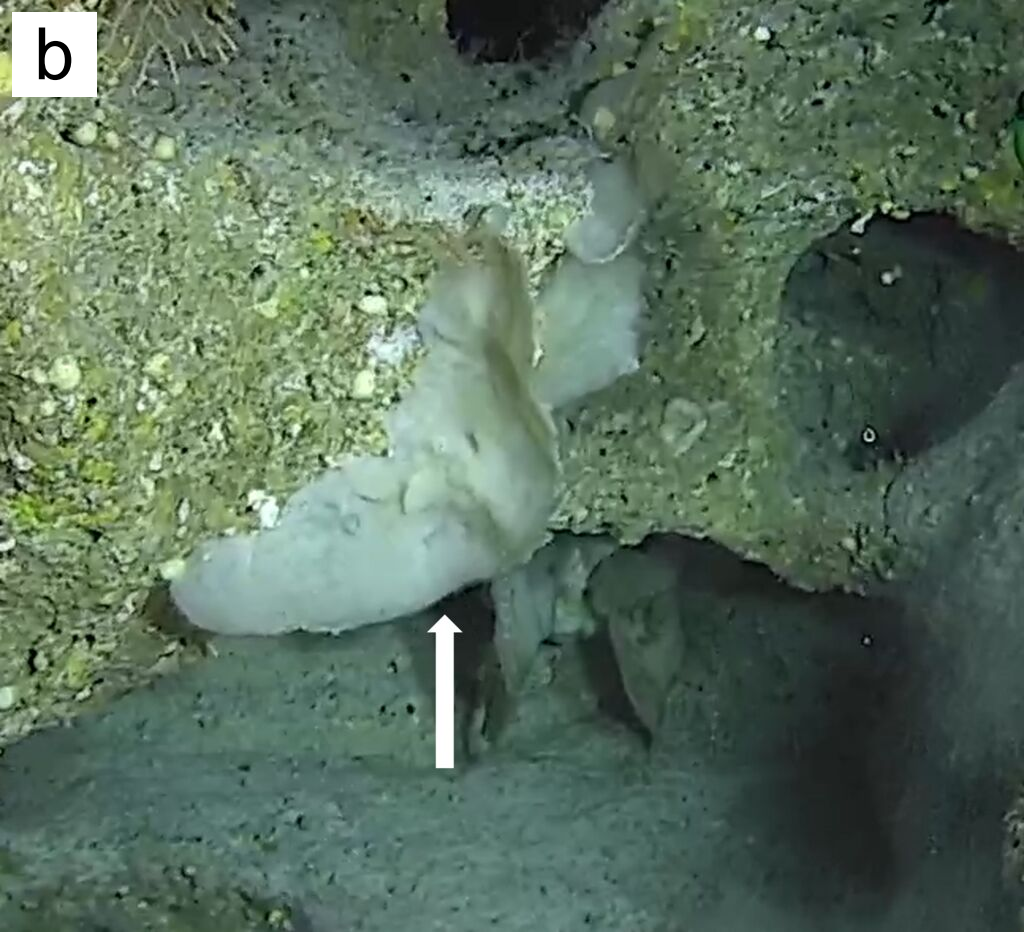
Huvadhu, 120 m;

**Figure 13c.**
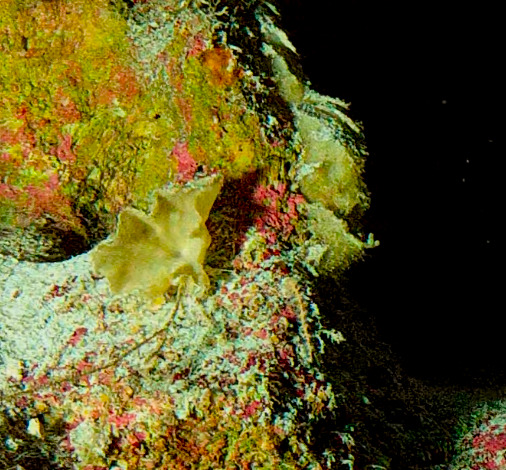
Laamu, 116 m, *in situ* photo of collected specimen MAL1_550;

**Figure 13d.**
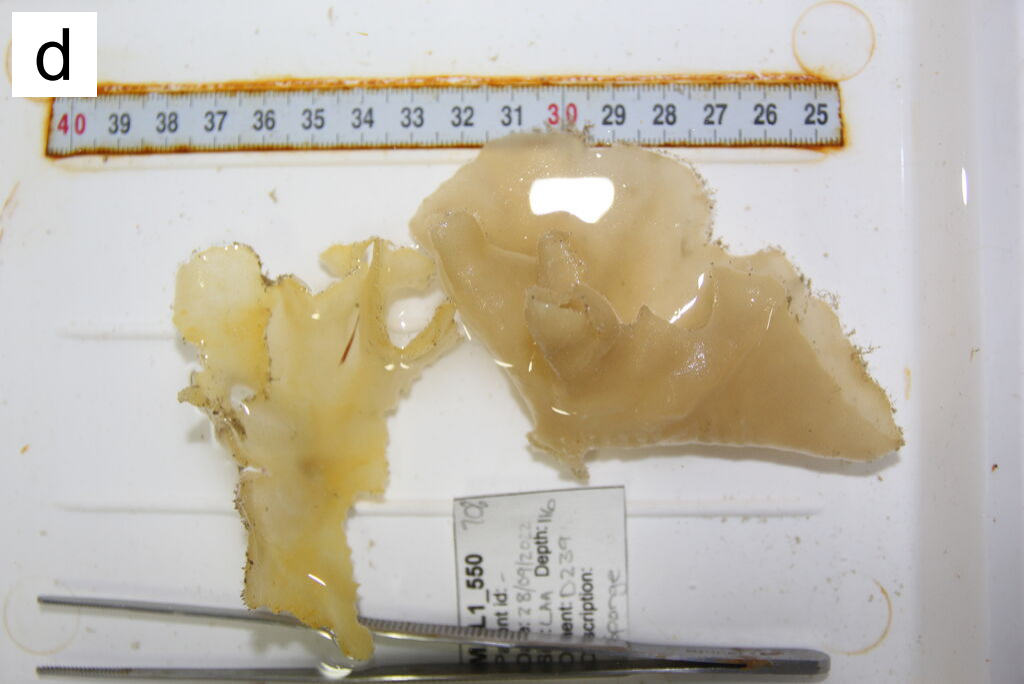
Laamu, 116 m, collected specimen MAL1_550.

**Figure 14a.**
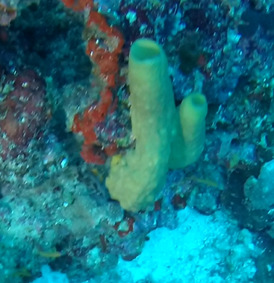
North Male’, 30 m;

**Figure 14b.**
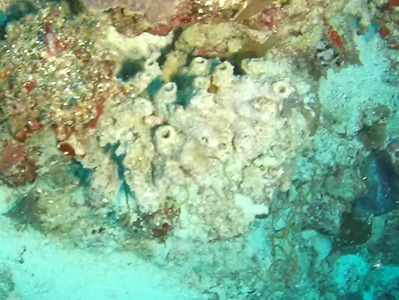
Vaavu, 30 m.

**Figure 15.**
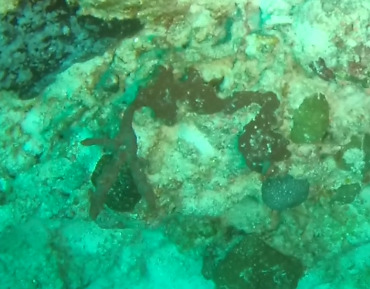
Petrosiidae gen. indet. sp. 3, Huvadhu, 30 m.

**Figure 16a.**
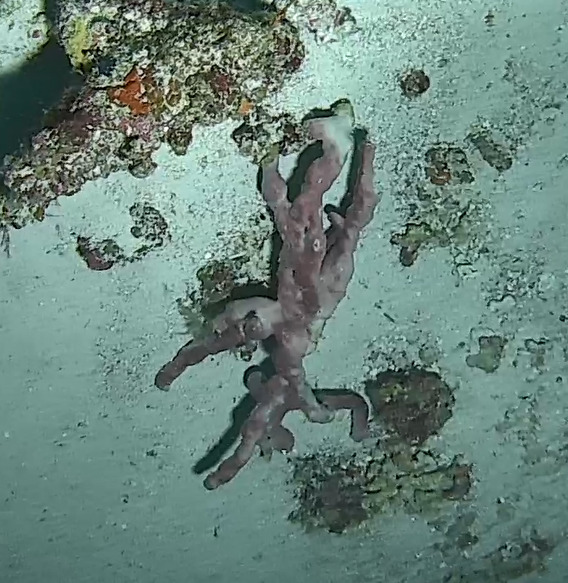
North Male’, 60 m;

**Figure 16b.**
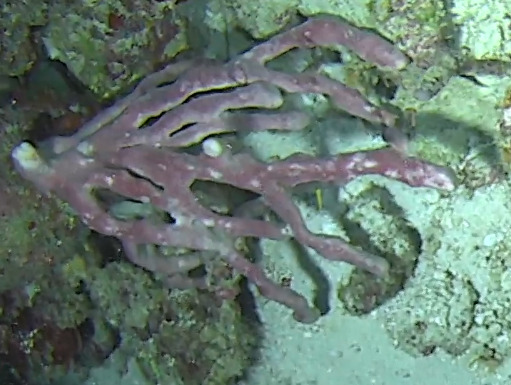
North Male’, 60 m;

**Figure 16c.**
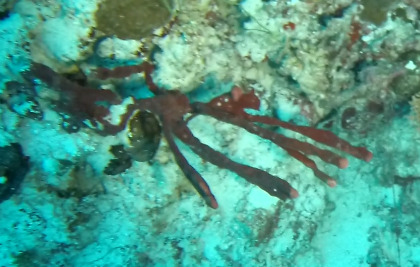
Vaavu, 30 m;

**Figure 16d.**
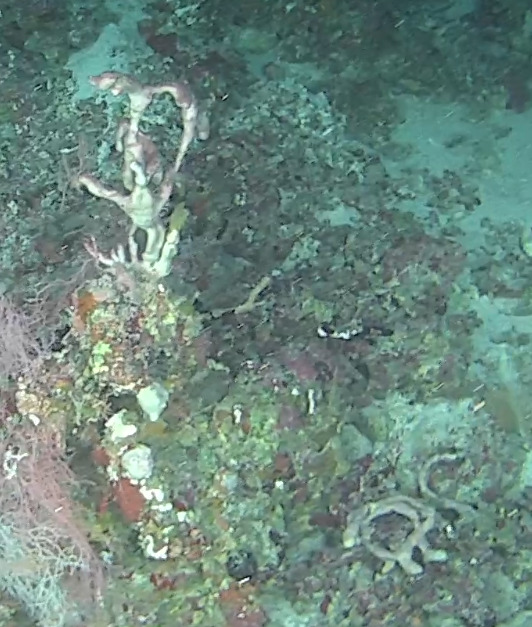
Vaavu, 60 m.

**Figure 17a.**
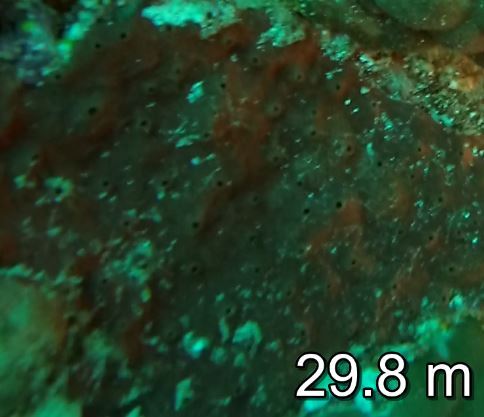
Huvadhu, 30 m;

**Figure 17b.**
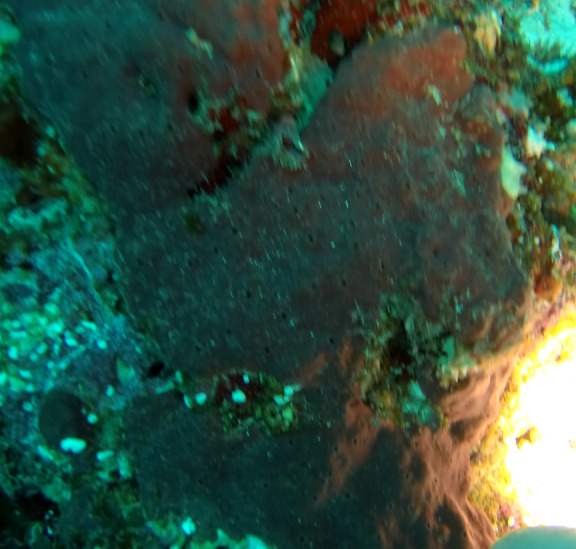
North Male’, 30 m.

**Figure 18a.**
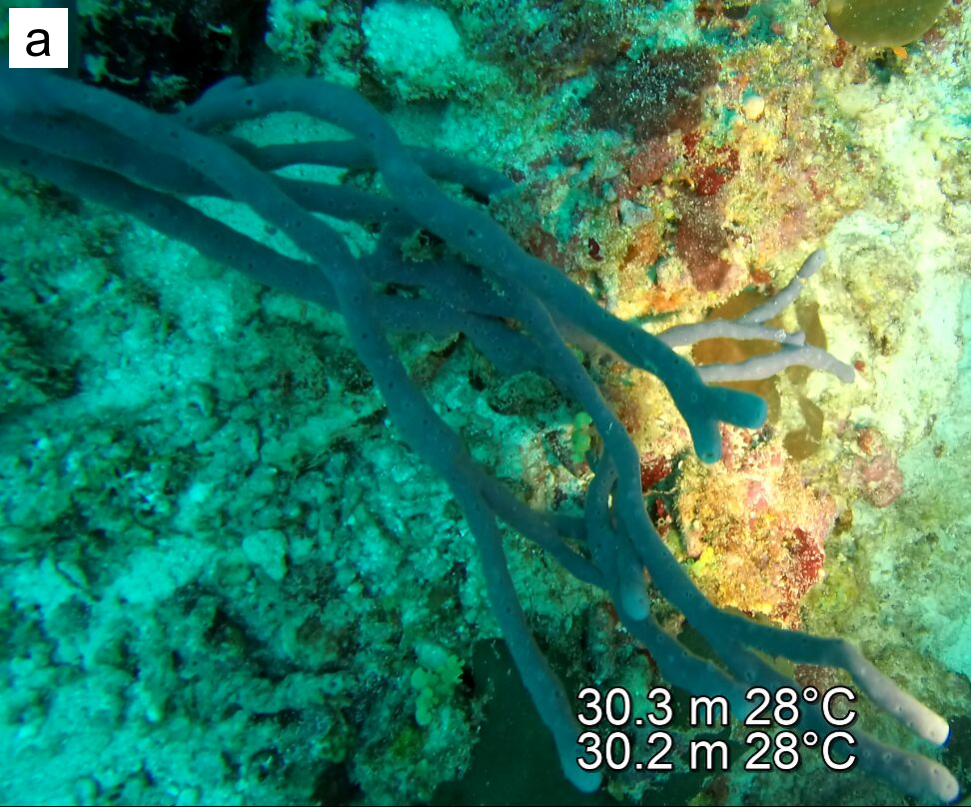
North Male’, 30 m;

**Figure 18b.**
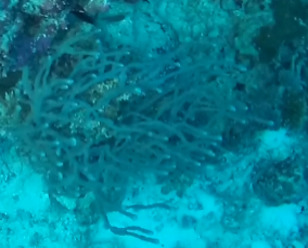
Huvadhu, 30 m.

**Figure 19a.**
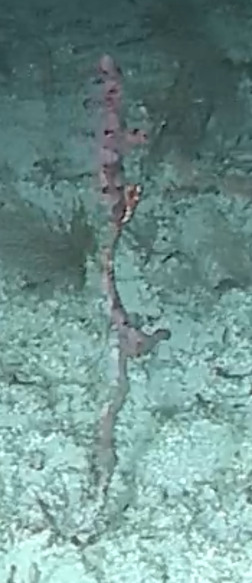
Laamu, 60 m;

**Figure 19b.**
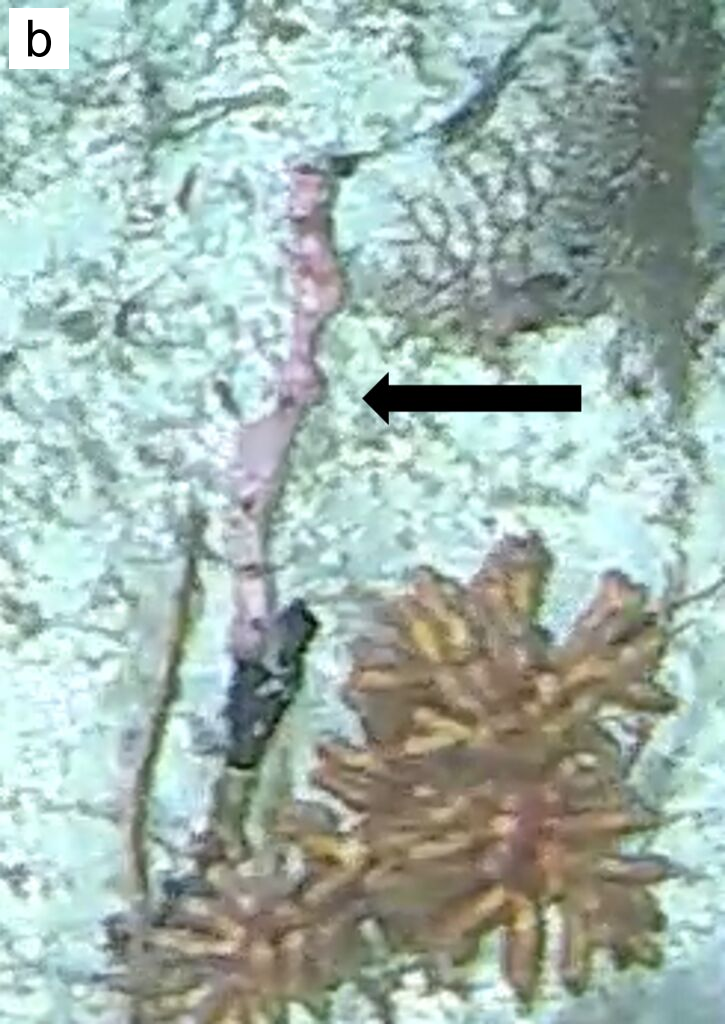
Laamu, 60 m.

**Figure 20a.**
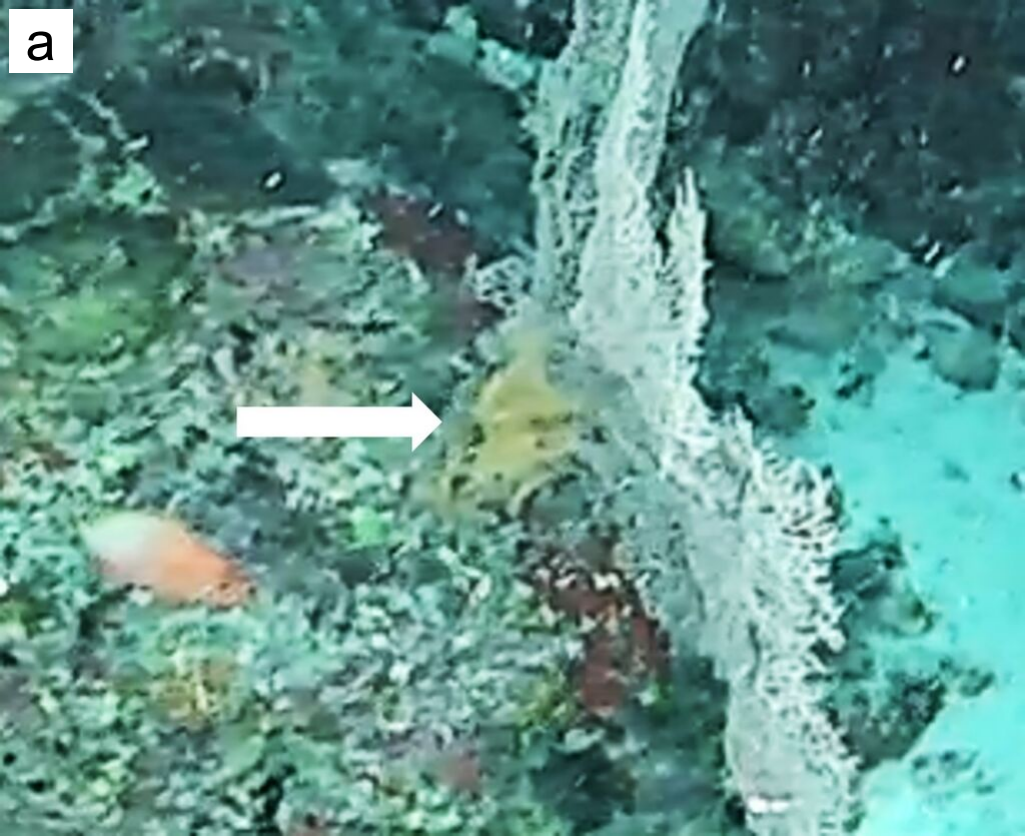
North Male’, 60 m;

**Figure 20b.**
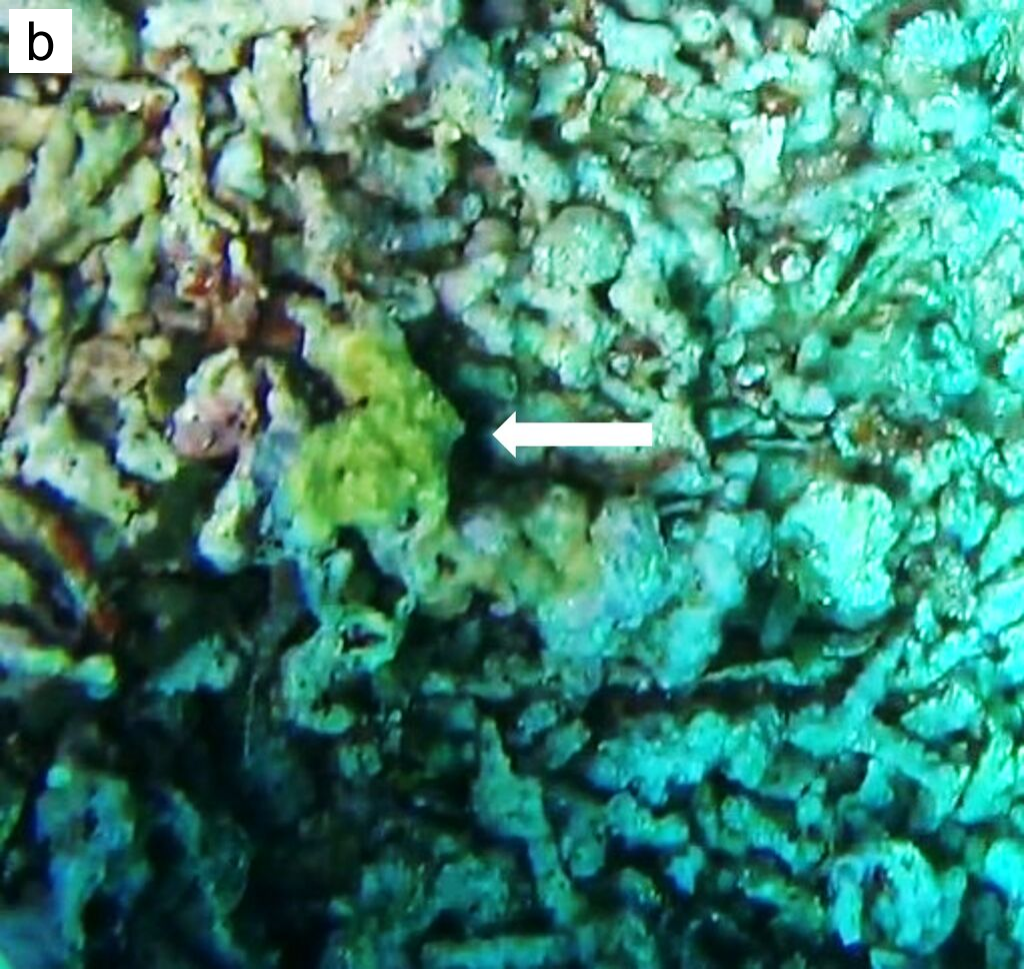
Addu, 10 m.

**Figure 21a.**
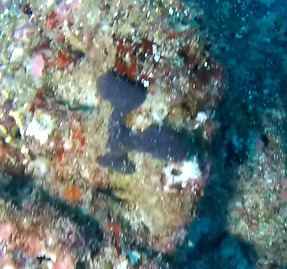
North Male’, 30 m;

**Figure 21b.**
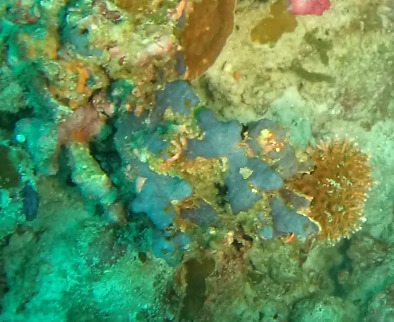
Huvadhu, 30 m.

**Figure 22a.**
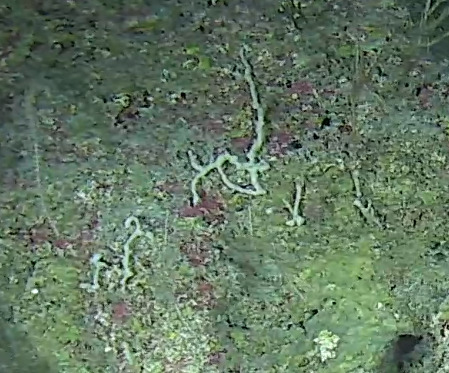
North Male’, 120 m;

**Figure 22b.**
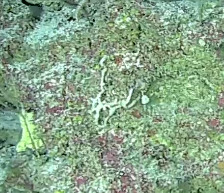
North Male’, 120 m.

**Figure 23.**
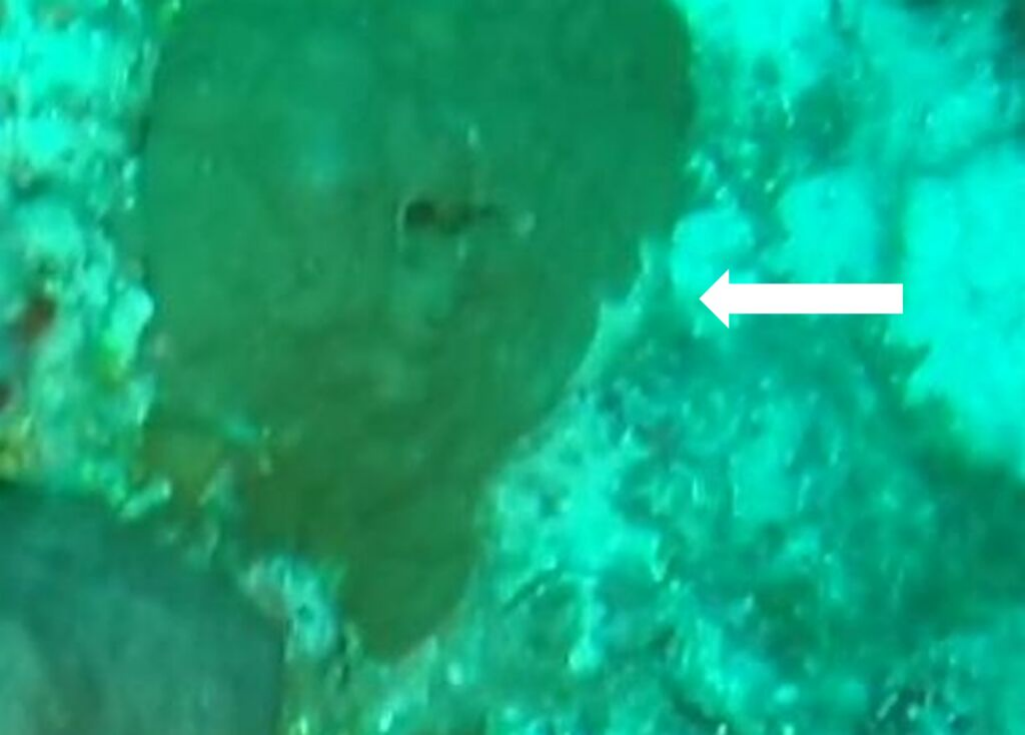
*Suberites* sp. indet. 3, Laamu, 10 m.

**Figure 24a.**
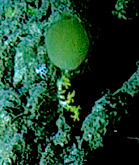
Addu, 250 m, *in situ* photo of collected specimen MAL1_292;

**Figure 24b.**
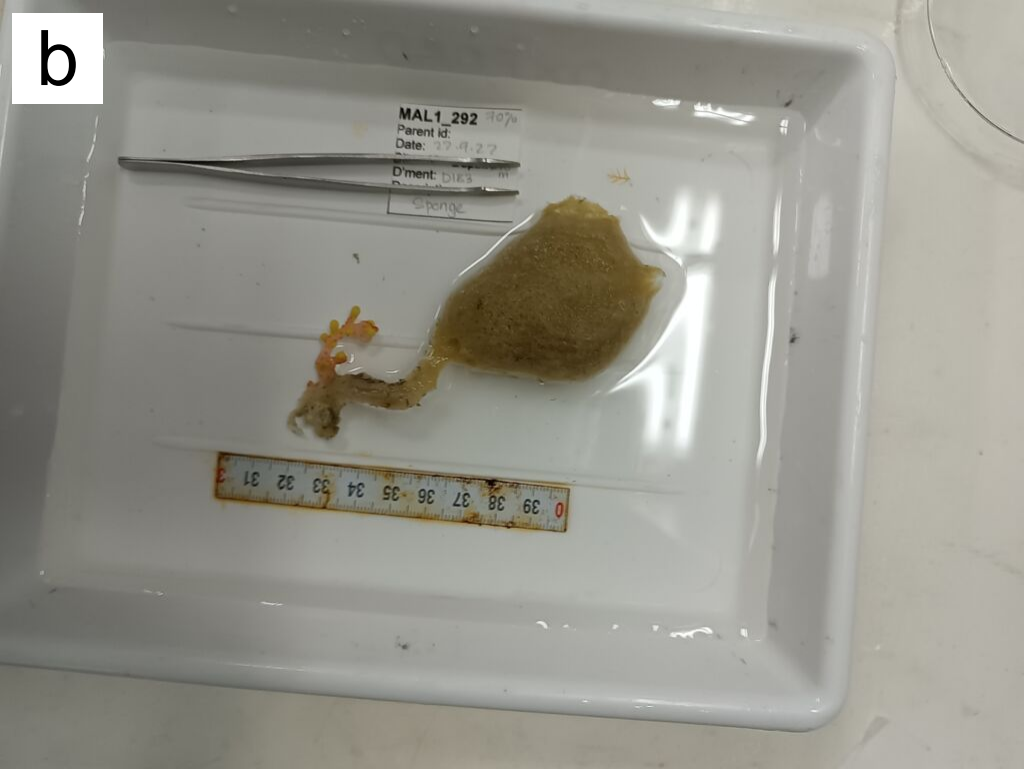
Addu, 250 m, collected specimen MAL1_292.

**Figure 25a.**
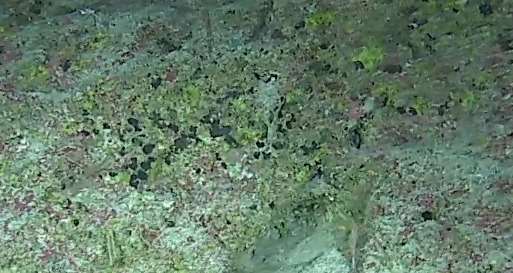
North Male’, 120 m

**Figure 25b.**
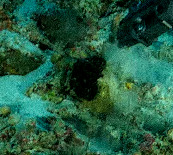
Vaavu, 62 m, *in situ* photo of collected specimen MAL1_635;

**Figure 25c.**
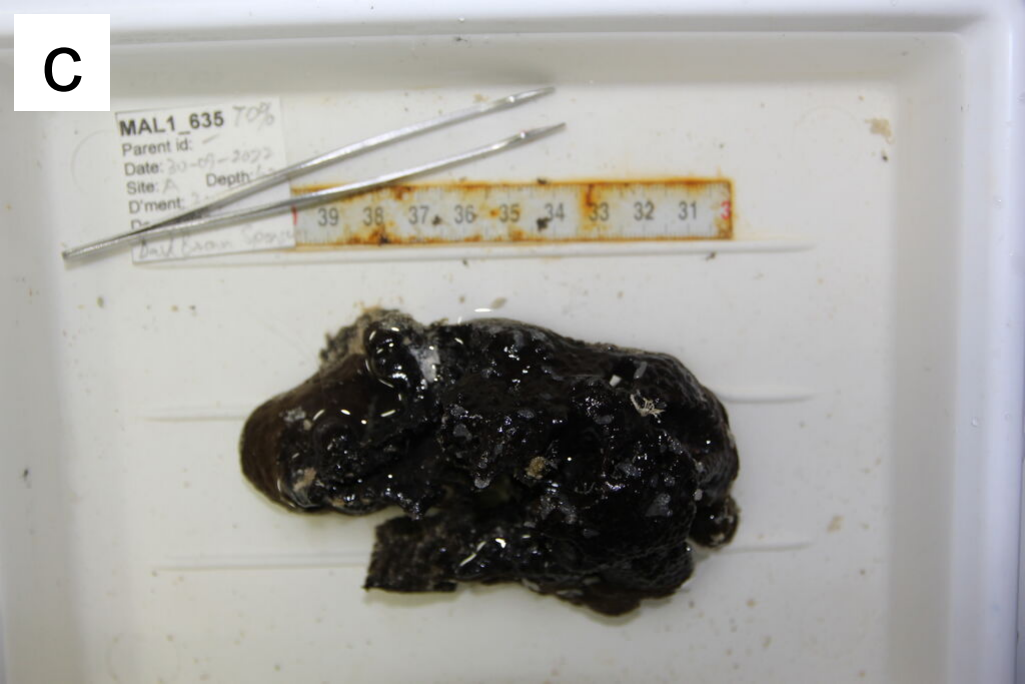
Vaavu, 62 m, collected specimen MAL1_635;

**Figure 25d.**
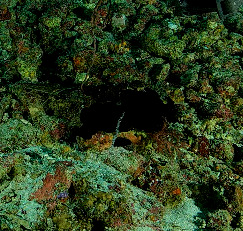
North Male’, 67 m, *in situ* photo of collected specimen MAL1_724;

**Figure 25e.**
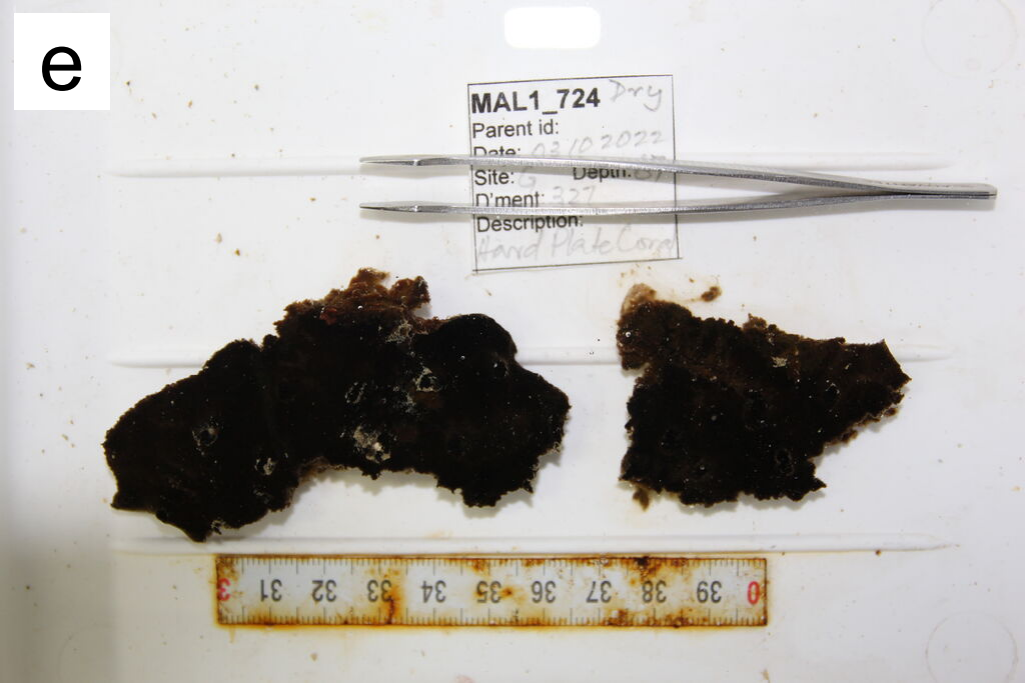
North Male’, 67 m, collected specimen MAL1_724.

**Figure 26a.**
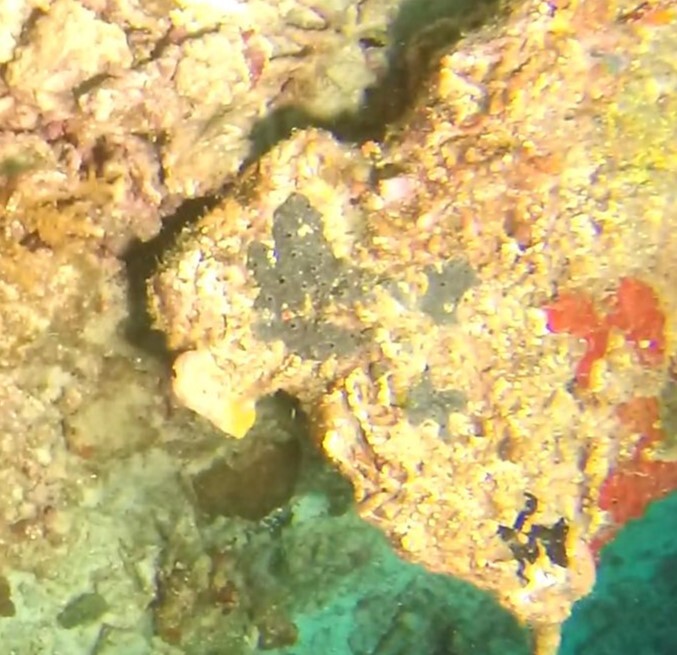
Addu, 30 m;

**Figure 26b.**
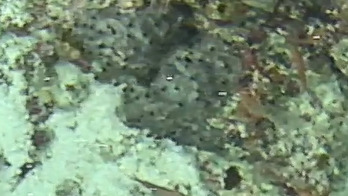
Vaavu, 60 m.

**Figure 27.**
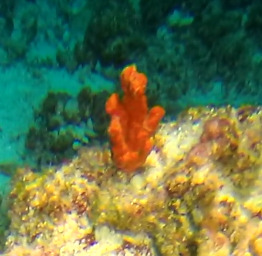
*Clathria* sp. indet. 1, North Male’, 30 m.

**Figure 28a.**
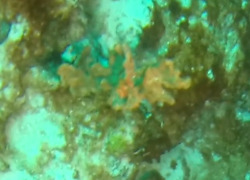
Huvadhu, 30 m;

**Figure 28b.**
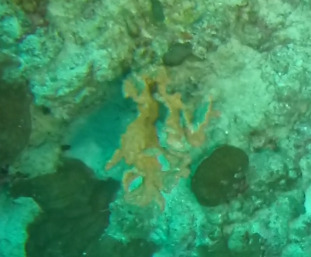
Huvadhu, 30 m;

**Figure 28c.**
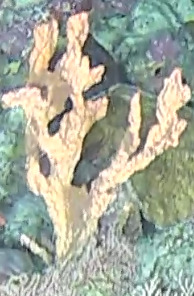
Huvadhu, 60 m.

**Figure 29.**
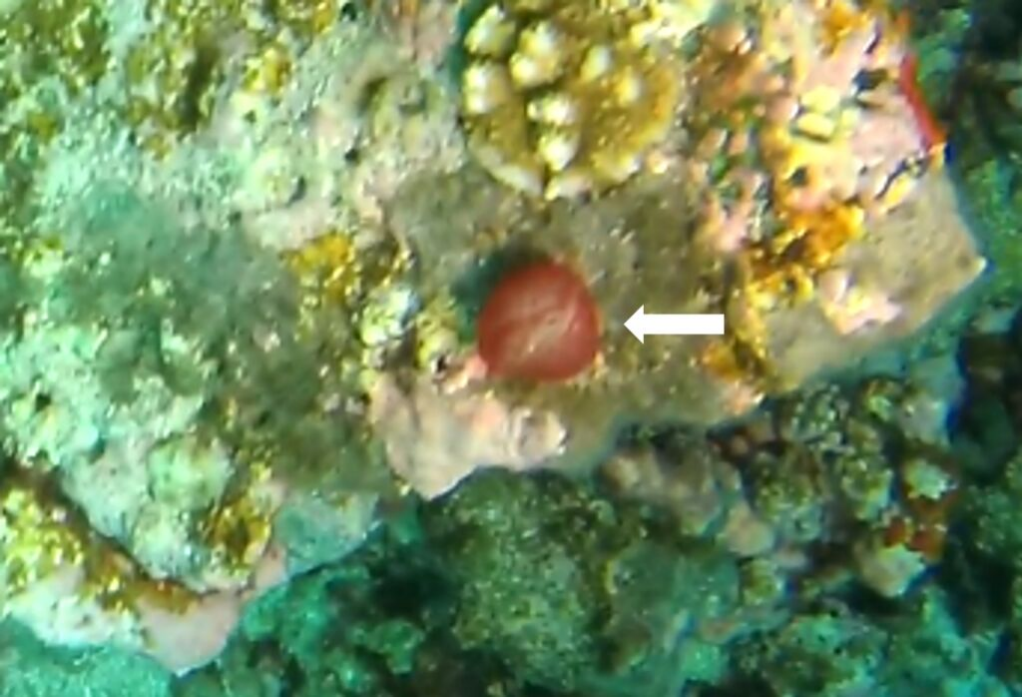
*Stelletta* sp. indet. 2, Addu, 10 m.

**Figure 30a.**
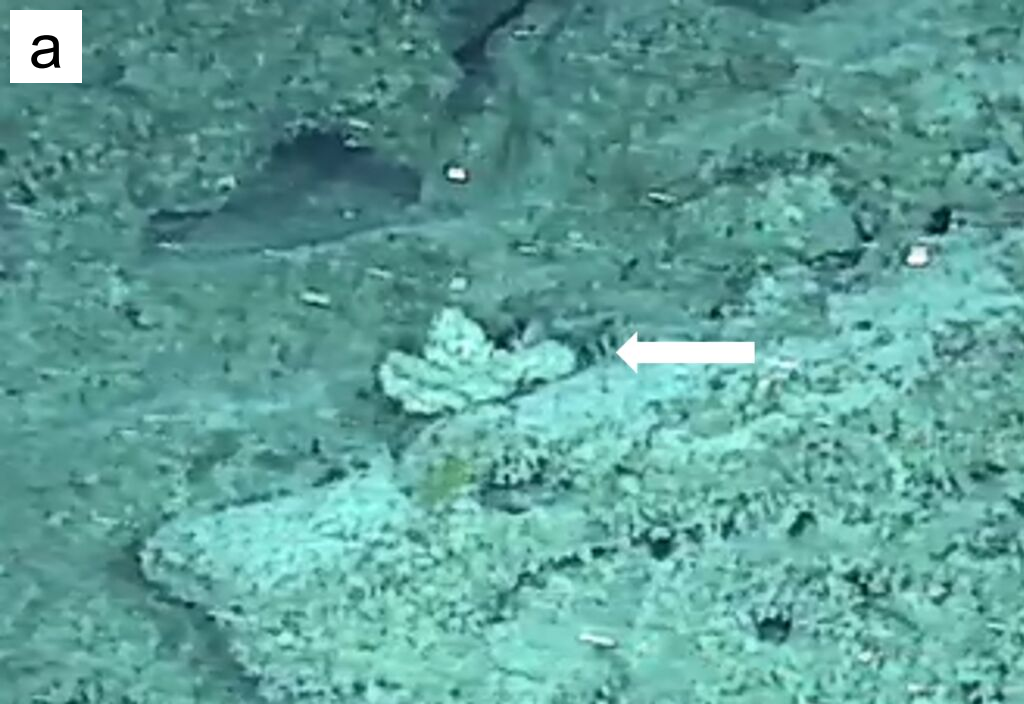
Vaavu, 120 m;

**Figure 30b.**
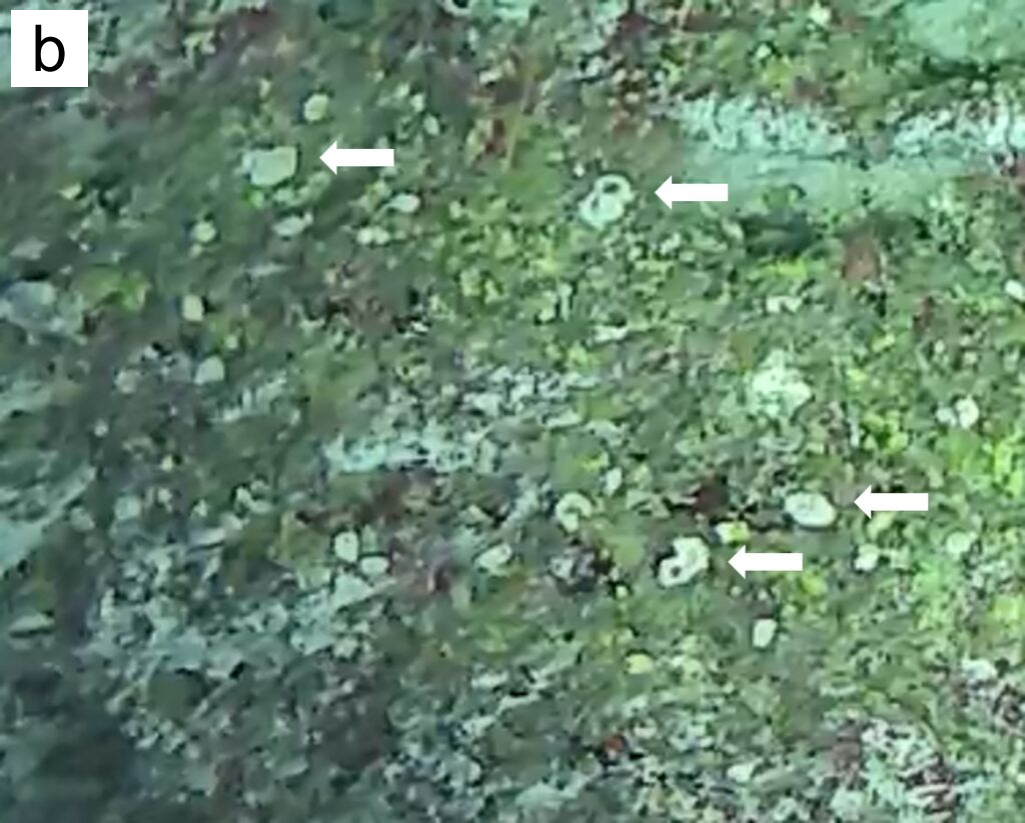
Huvadhu, 120 m;

**Figure 30c.**
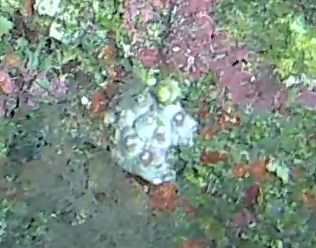
Addu, 60 m;

**Figure 30d.**
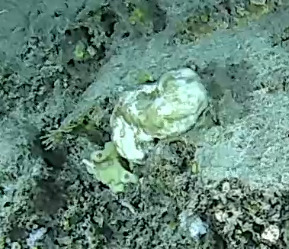
Laamu, 490 m.

**Figure 31a.**
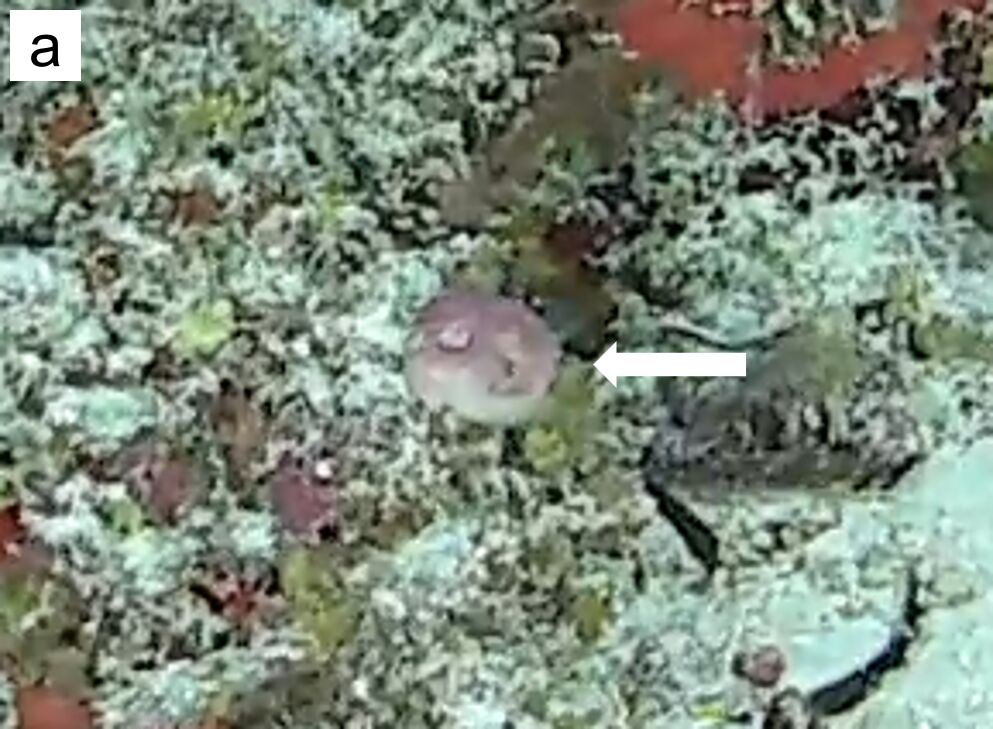
Laamu, 60 m;

**Figure 31b.**
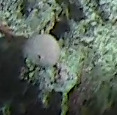
Huvadhu, 120 m.

**Figure 32.**
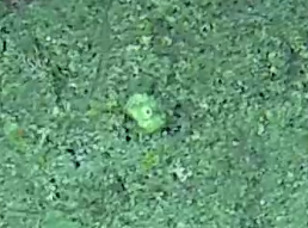
*Geodia* sp. indet. 4, Vaavu, 120 m.

**Figure 33a.**
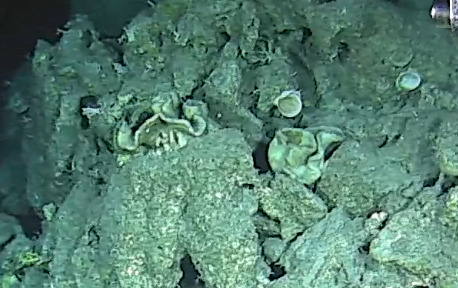
Addu, 250 m;

**Figure 33b.**
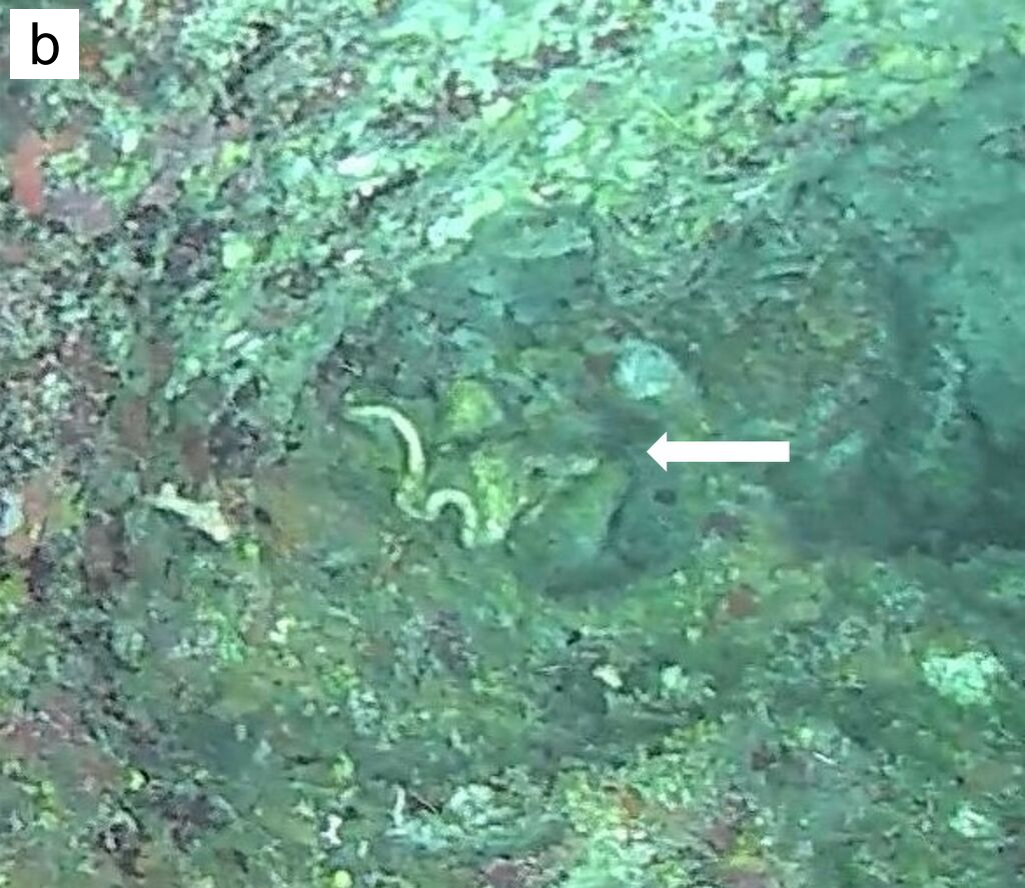
Addu, 60 m;

**Figure 33c.**
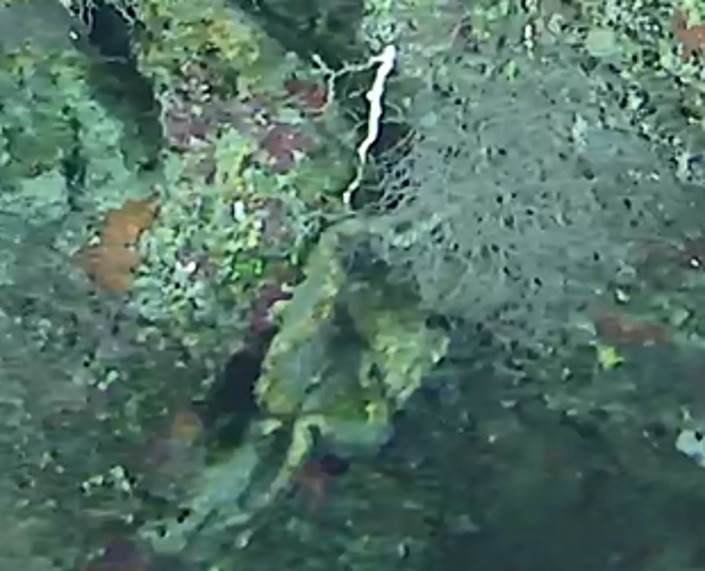
Addu, 60 m.

**Figure 34a.**
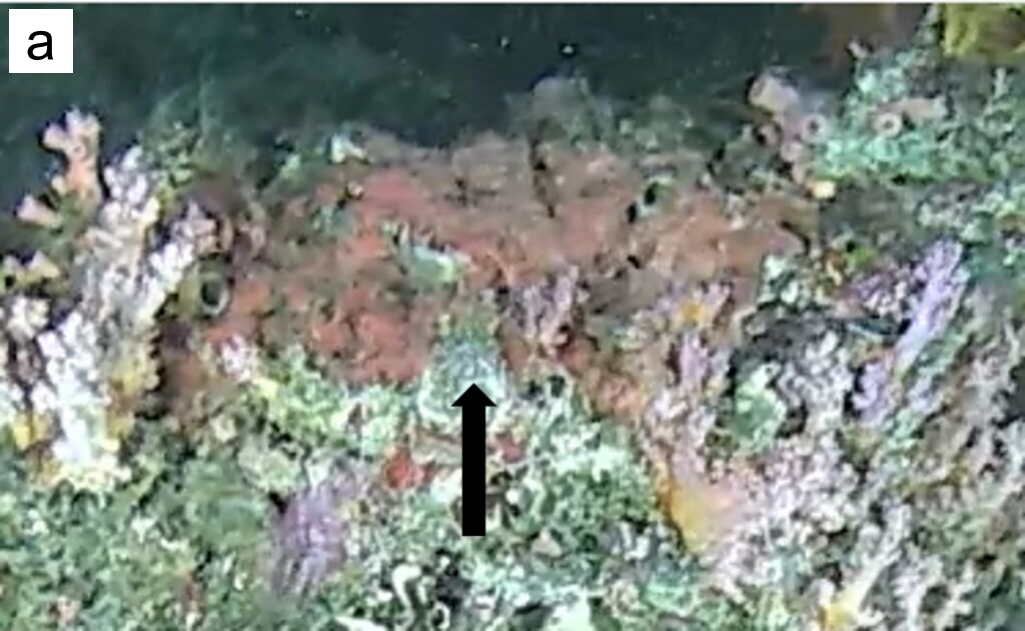
Huvadhu, 60 m;

**Figure 34b.**
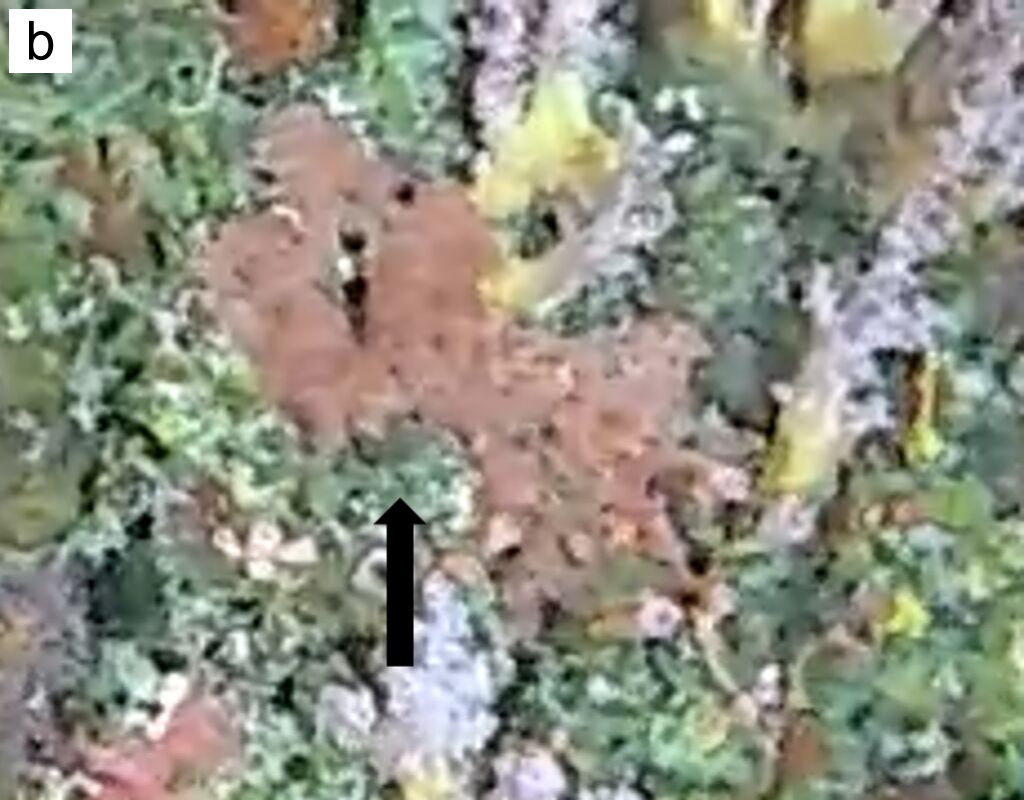
Huvadhu, 60 m.

**Figure 35a.**
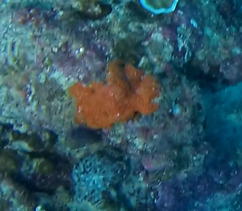
North Male, 30 m, *Biemna* sp. indet.;

**Figure 35b.**
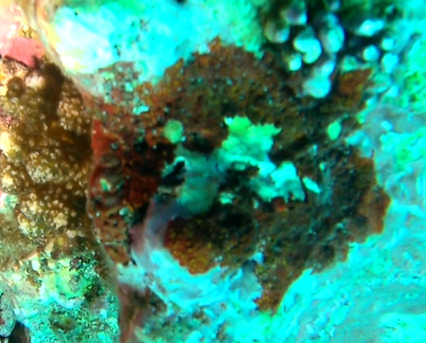
Laamu, 10 m, *Biemna* sp. indet.;

**Figure 35c.**
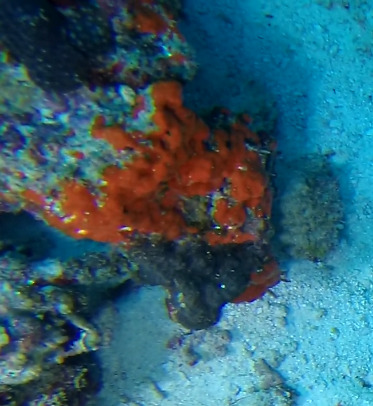
North Male, 30 m.

**Figure 36a.**
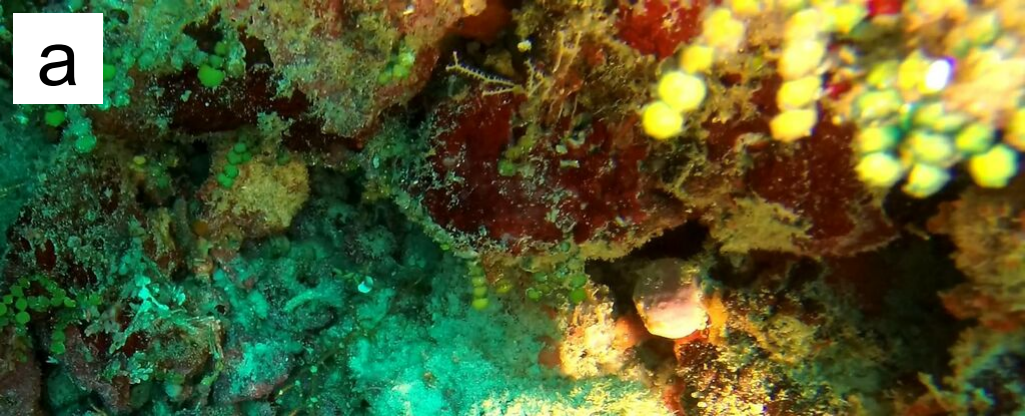
Laamu, 30 m;

**Figure 36b.**
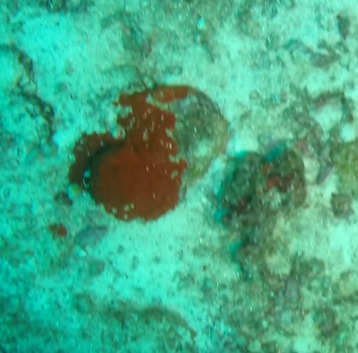
North Male’, 30 m, *Clathria* sp. indet.;

**Figure 36c.**
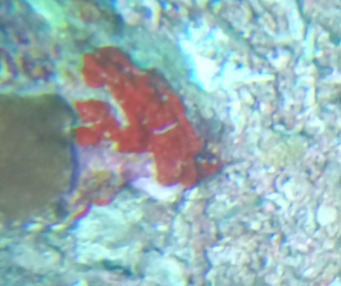
Addu, 30 m, *Clathria* sp. indet.

**Figure 37a.**
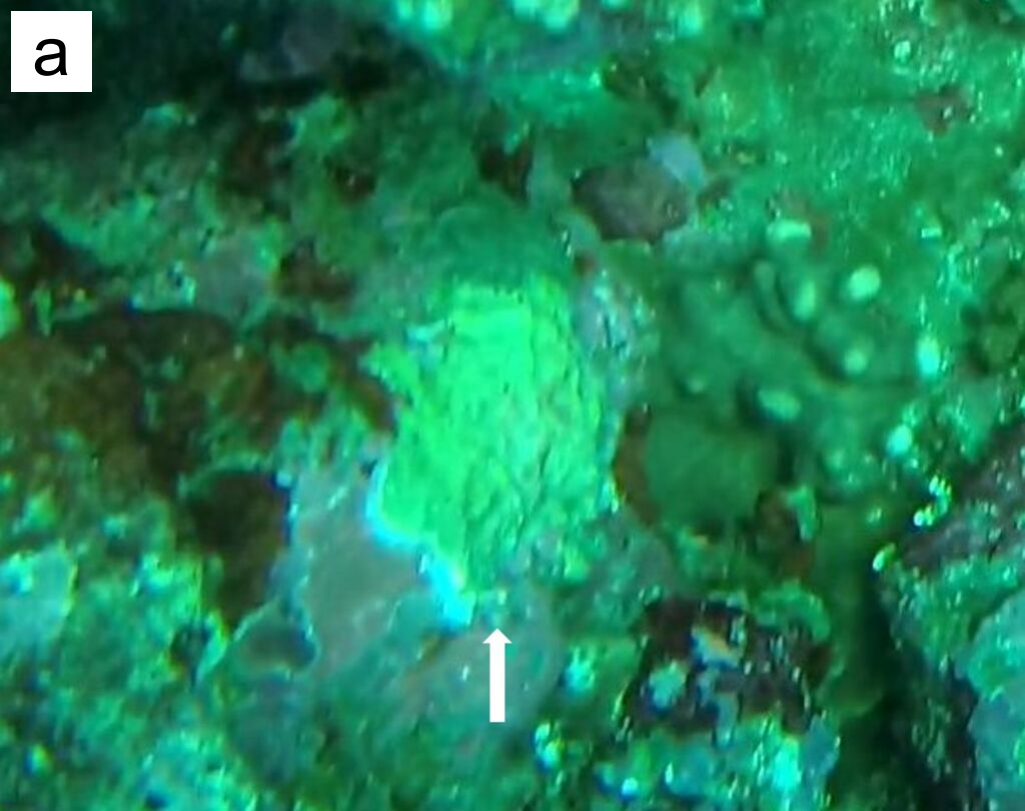
Laamu, 10 m, *Pseudoceratinapurpurea* sp. inc.;

**Figure 37b.**
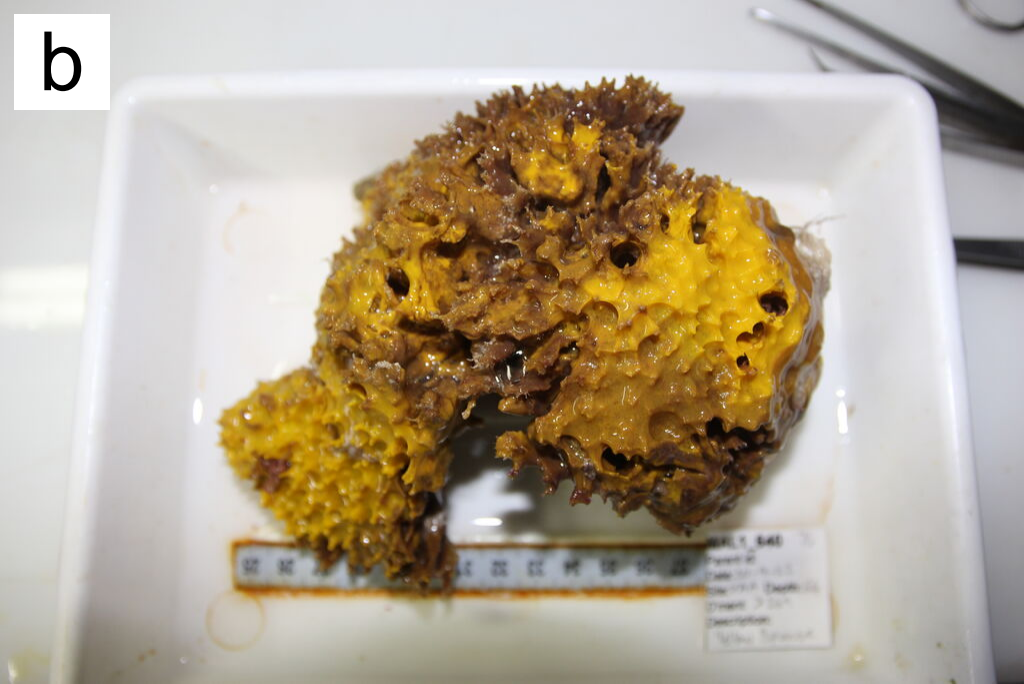
Vaavu, 64 m, collected specimen MAL1_640;

**Figure 37c.**
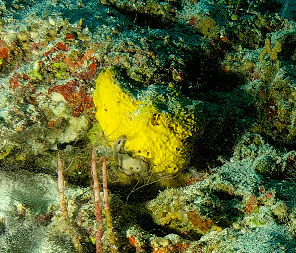
Vaavu, 64 m, *in situ* photo of collected specimen MAL1_640;

**Figure 37d.**
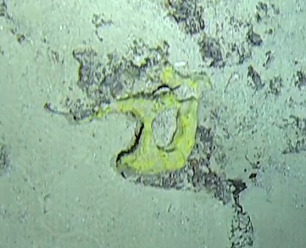
Huvadhu, 490 m, *Aplysillasulfurea* sp. inc.;

**Figure 37e.**
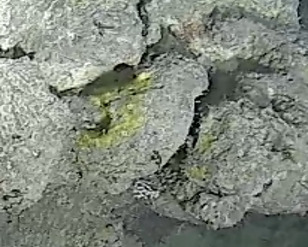
Huvadhu, 250 m, *Aplysillasulfurea* sp. inc.

**Figure 38a.**
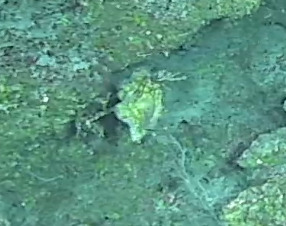
Addu, 120 m;

**Figure 38b.**
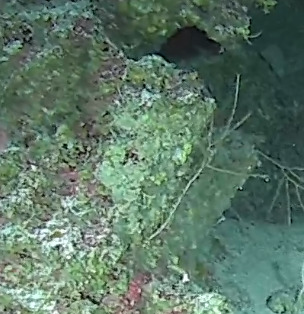
Laamu, 120 m.

**Figure 39.**
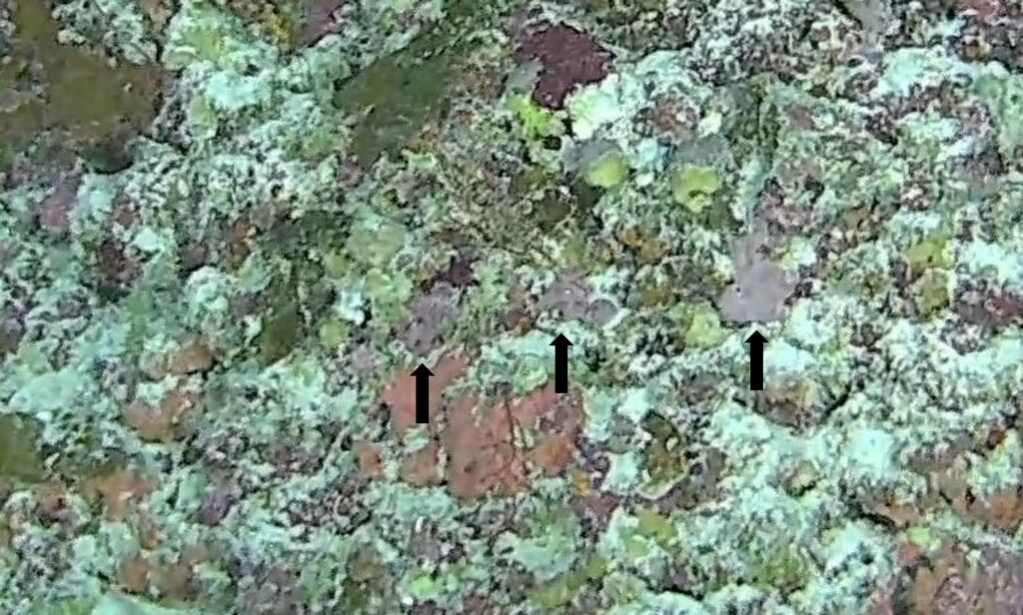
Demospongiae ord. indet. sp. 17, Addu, 60 m.

**Figure 40.**
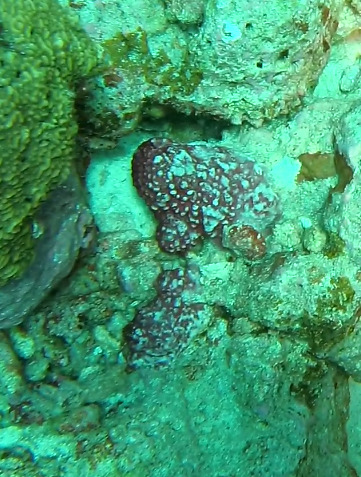
Demospongiae ord. indet. sp. 18, Huvadhu, 10 m.

**Figure 41a.**
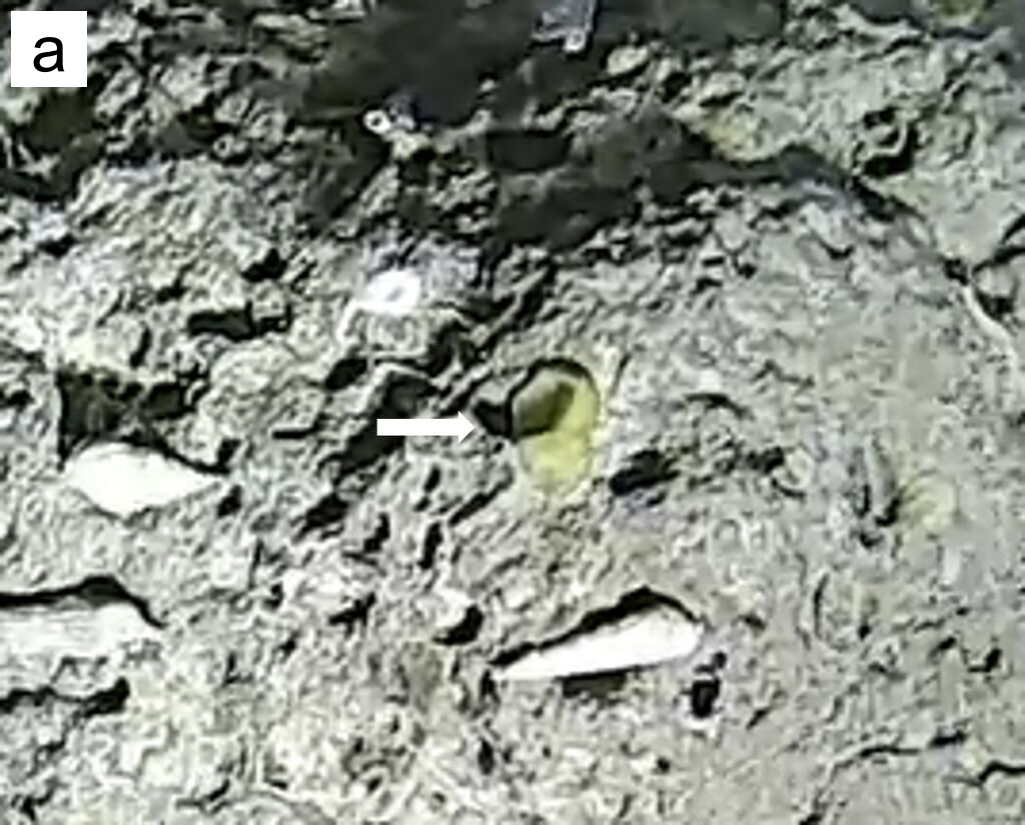
Huvadhu, 490 m;

**Figure 41b.**
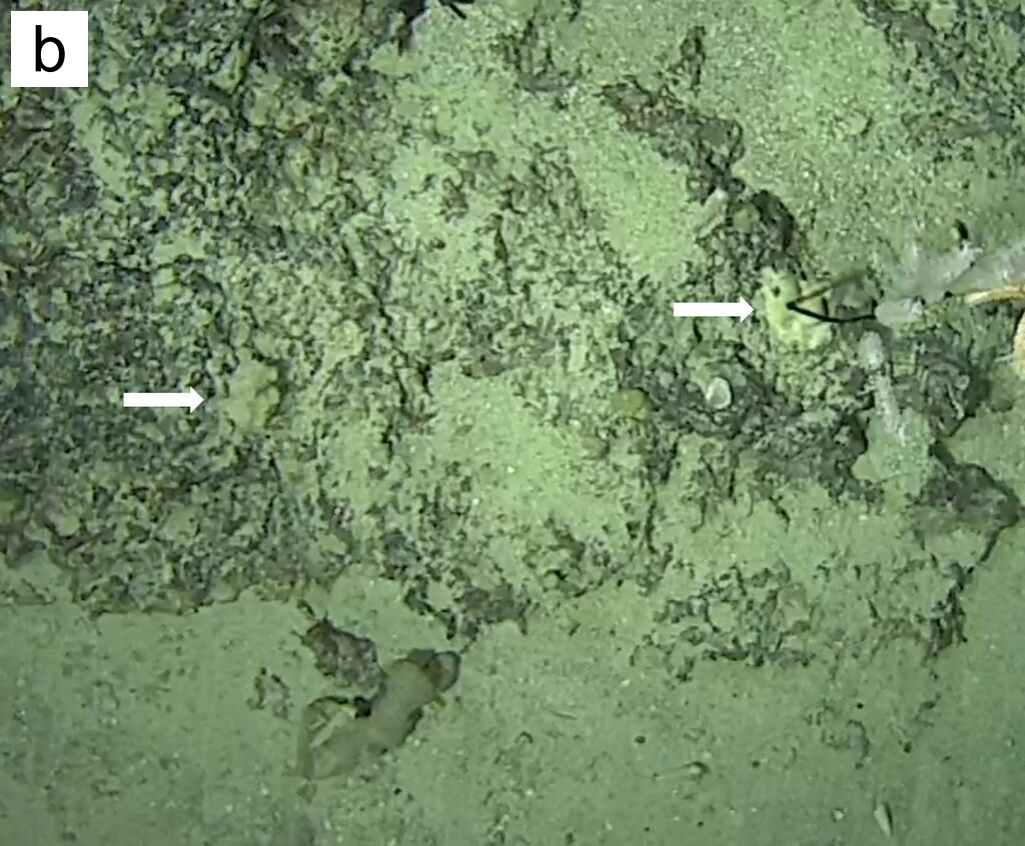
Huvadhu, 490 m.

**Figure 42a.**
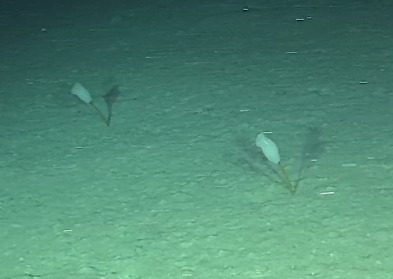
Vaavu, 490 m;

**Figure 42b.**
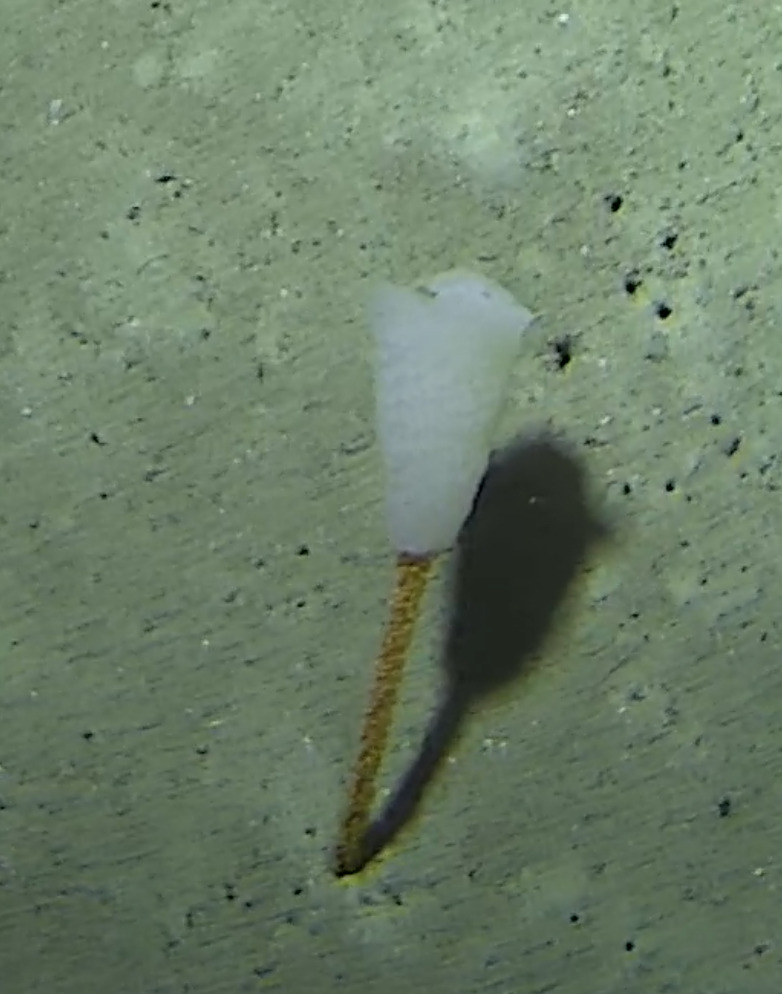
Vaavu, 490 m;

**Figure 42c.**
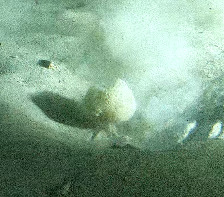
Huvadhu, 497 m, *in situ* photo of collected specimen MAL1_488;

**Figure 42d.**
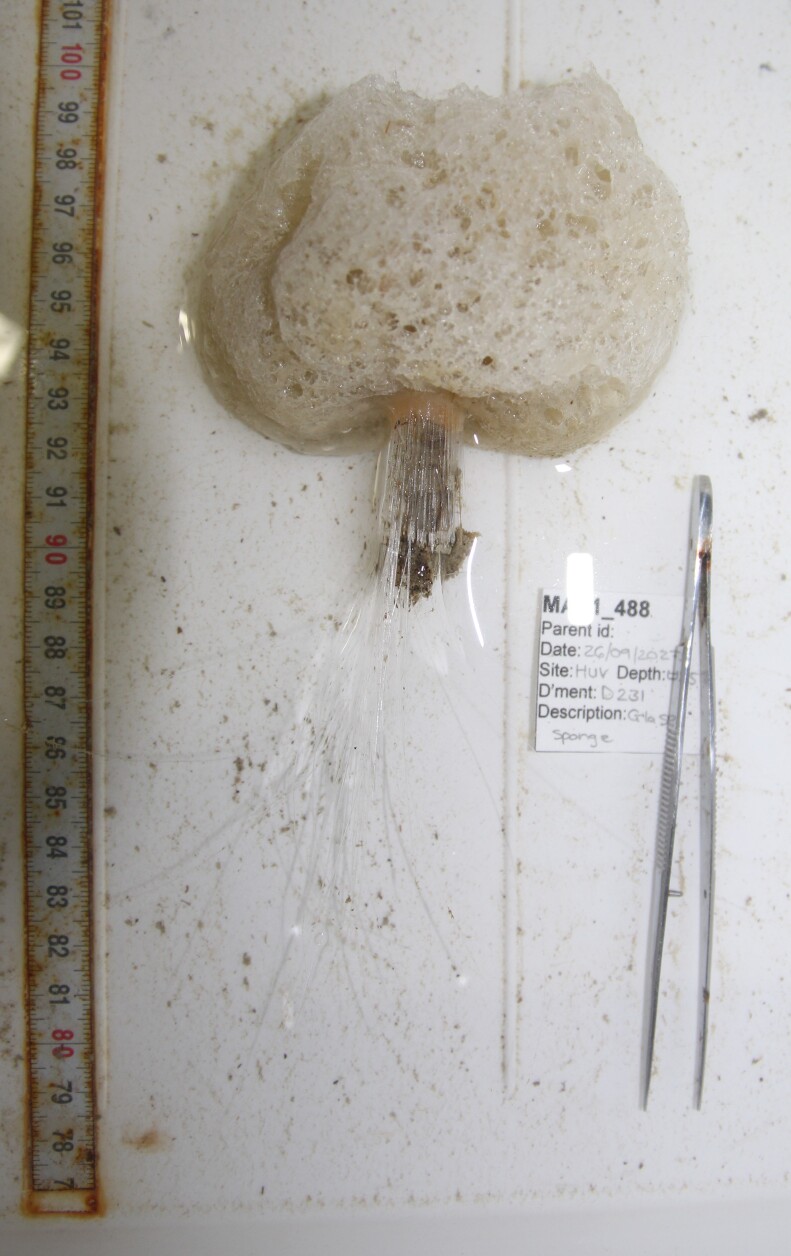
Huvadhu, 497 m, collected specimen MAL1_488.

**Figure 43a.**
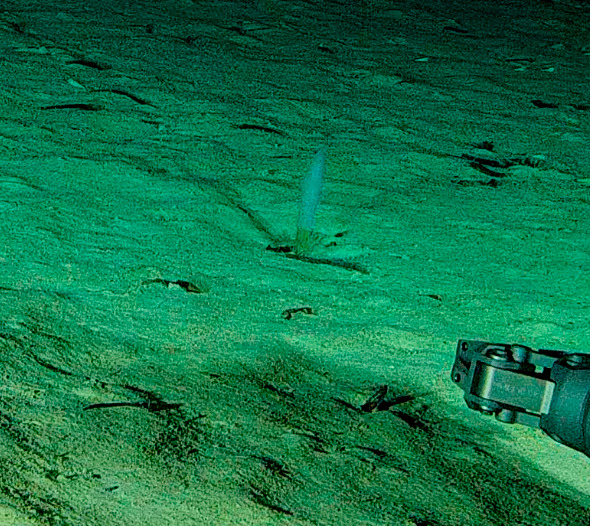
Huvadhu, 497 m, *in situ* photo of collected specimen MAL1_491;

**Figure 43b.**
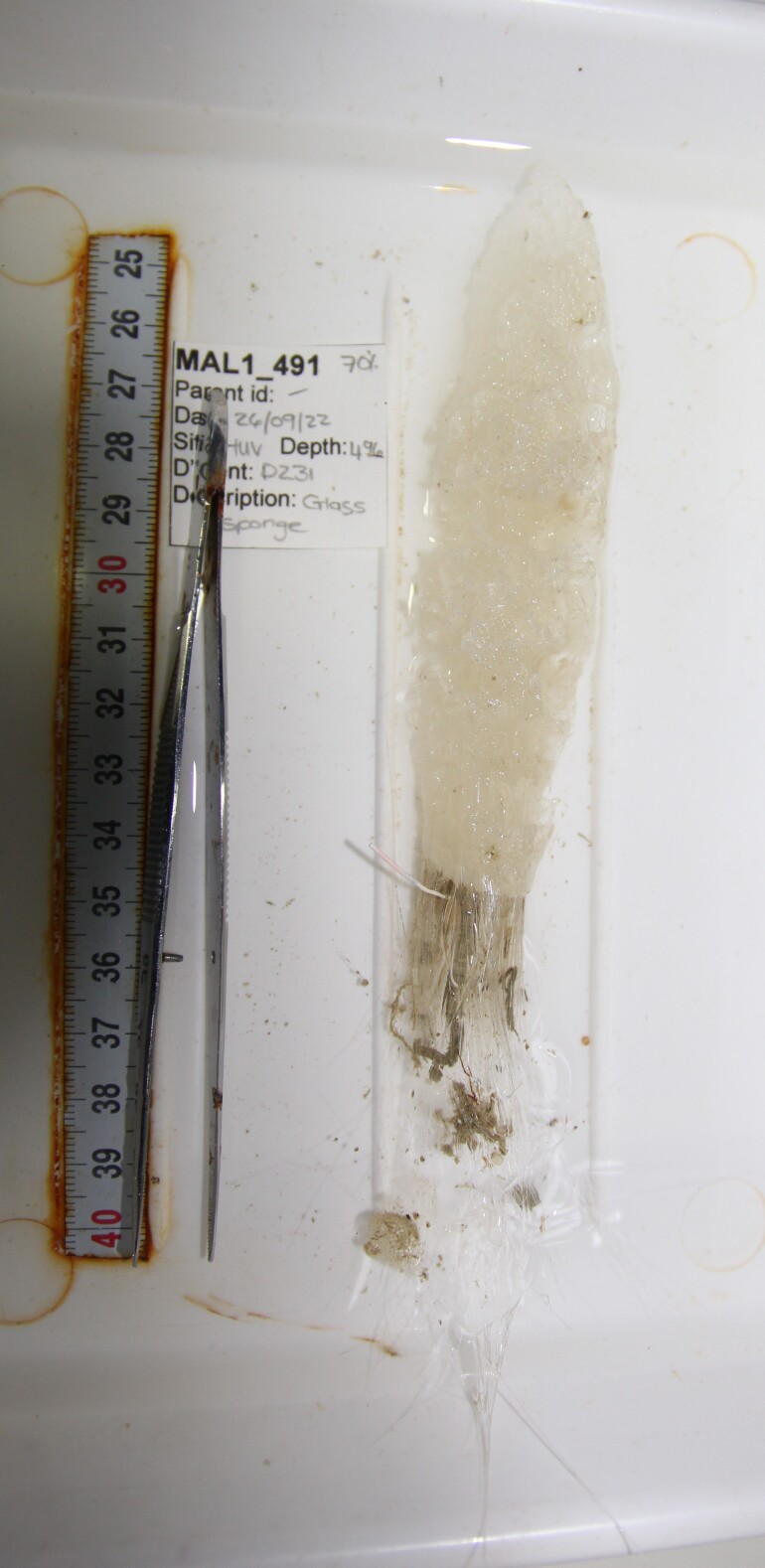
Huvadhu, 497 m, collected specimen MAL1_491;

**Figure 43c.**
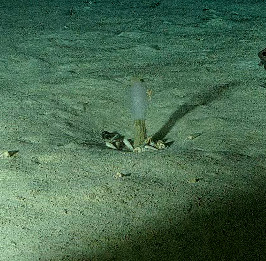
Huvadhu, 500 m, *in situ* photo of collected specimen MAL1_479;

**Figure 43d.**
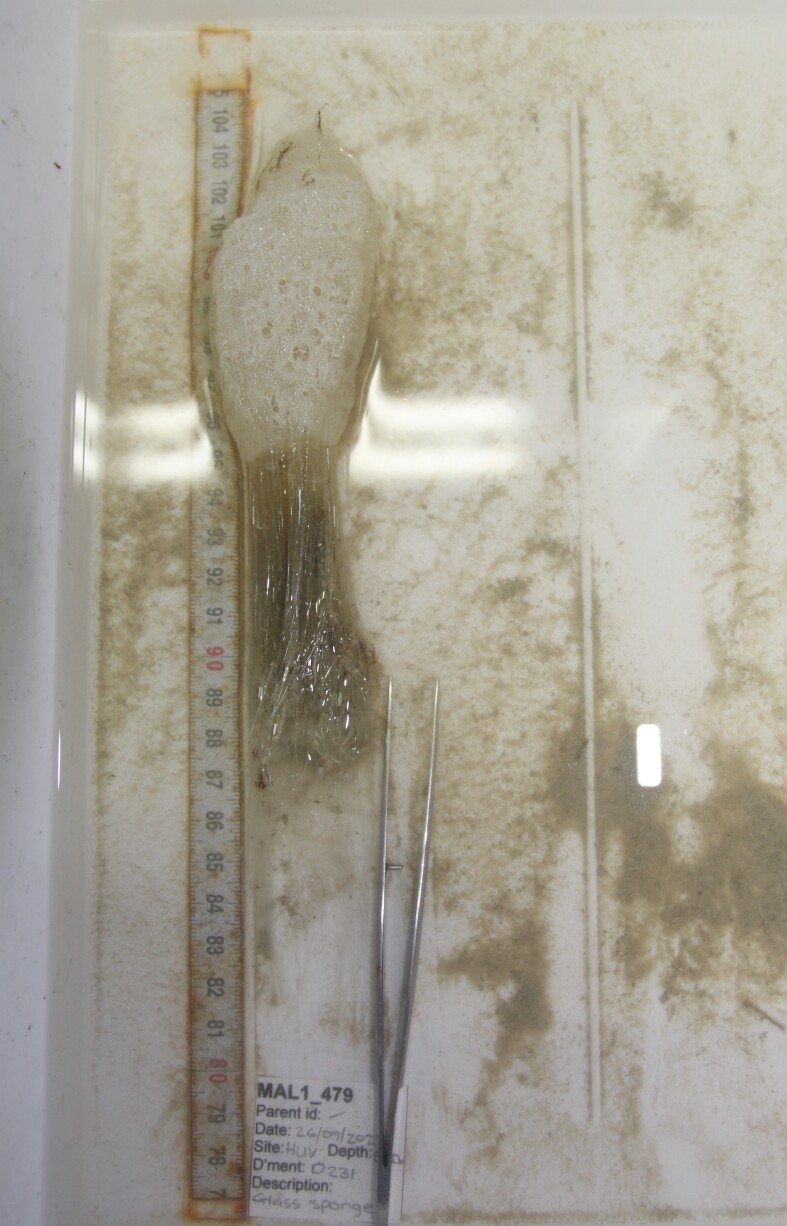
Huvadhu, 500 m, collected specimen MAL1_479.

**Figure 44.**
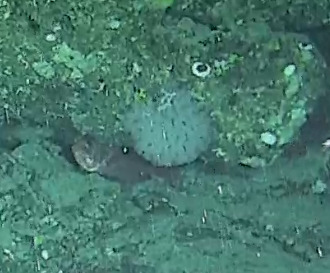
*Farrea* sp. indet. 1, Vaavu, 490 m.

**Figure 45a.**
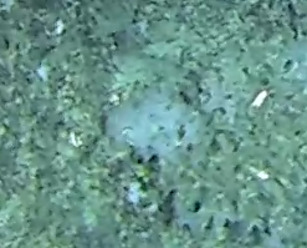
Laamu, 490 m;

**Figure 45b.**
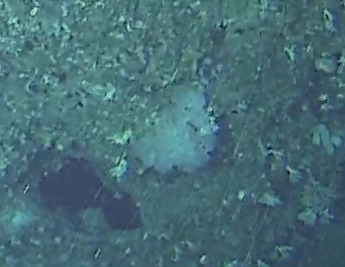
Laamu, 490 m.

**Figure 46a.**
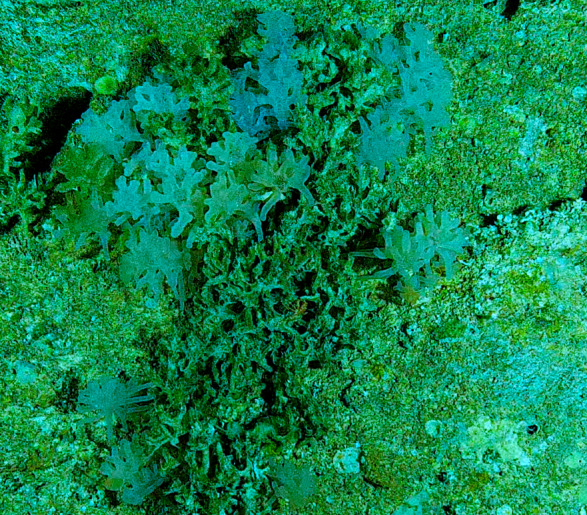
Fuvahmulah, 250 m;

**Figure 46b.**
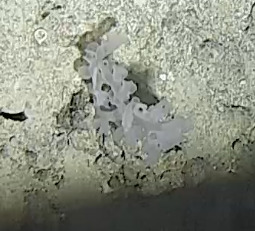
Addu, 490 m.

**Figure 47a.**
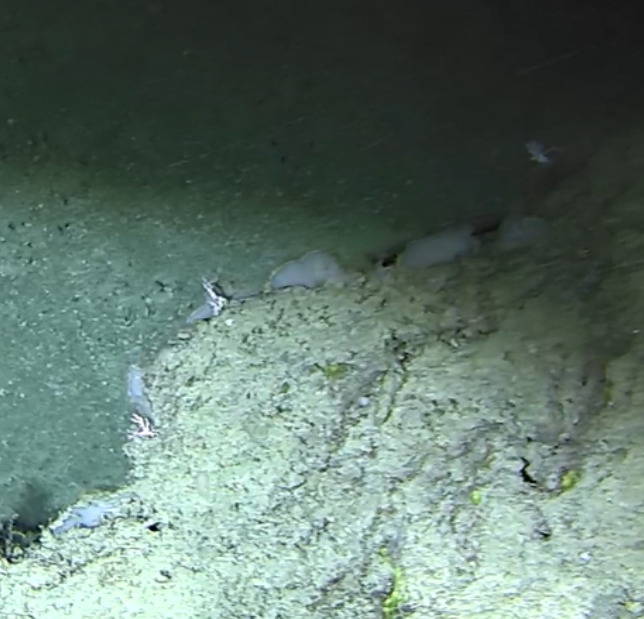
Vaavu, 490 m;

**Figure 47b.**
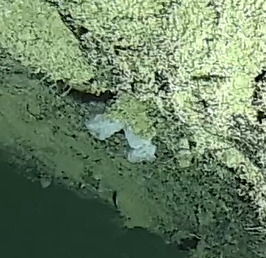
North Male’, 490 m.

**Figure 48a.**
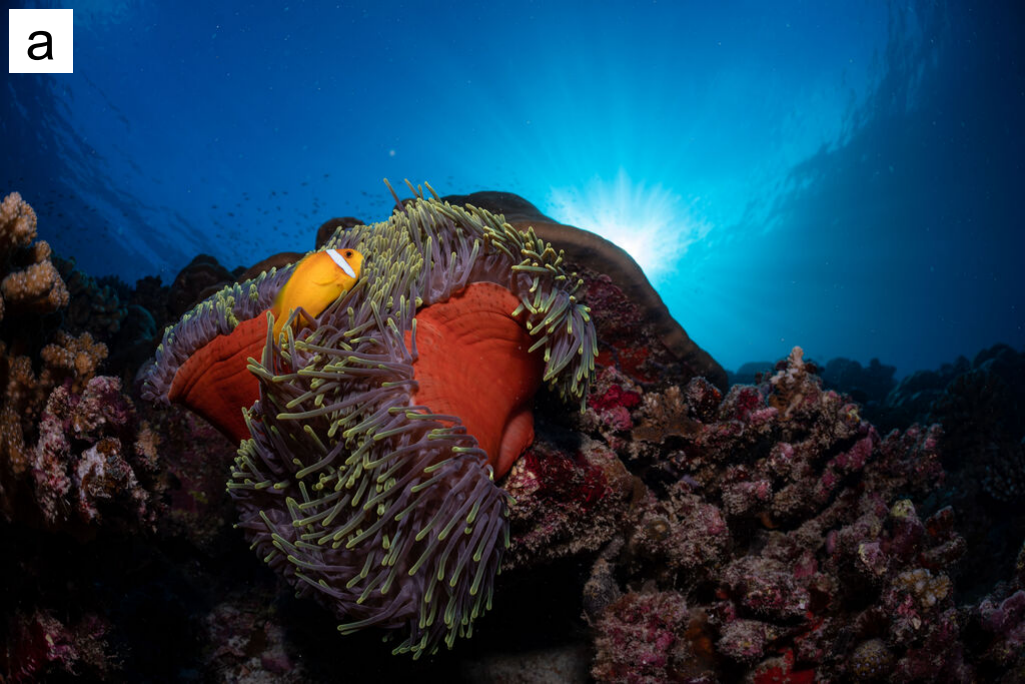
Fuvahmulah, 10-30 m;

**Figure 48b.**
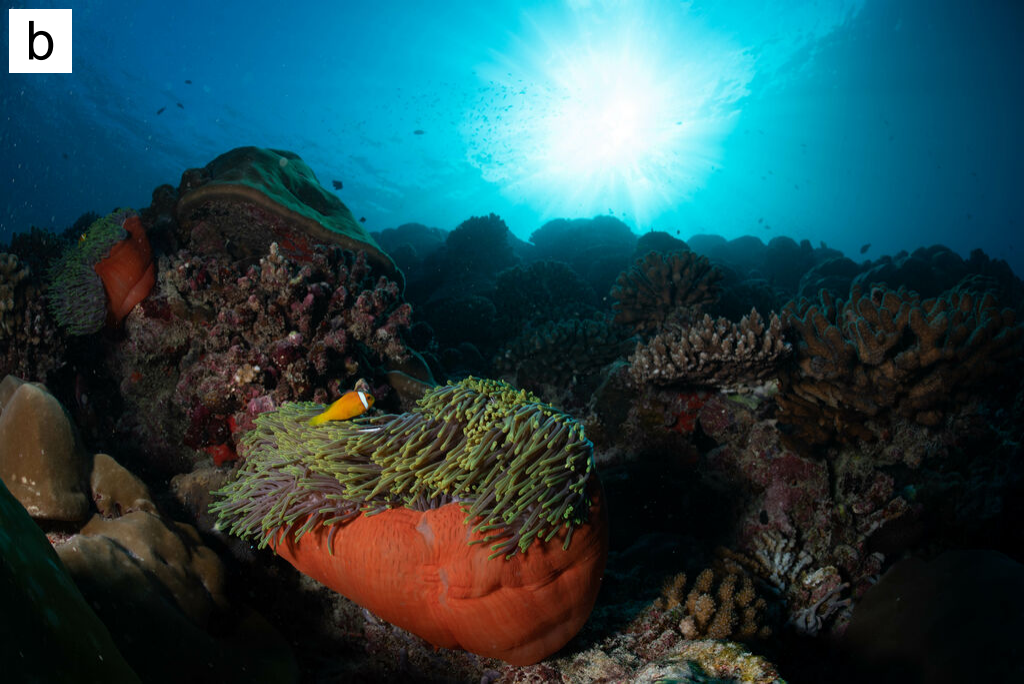
Fuvahmulah, 10-30 m;

**Figure 48c.**
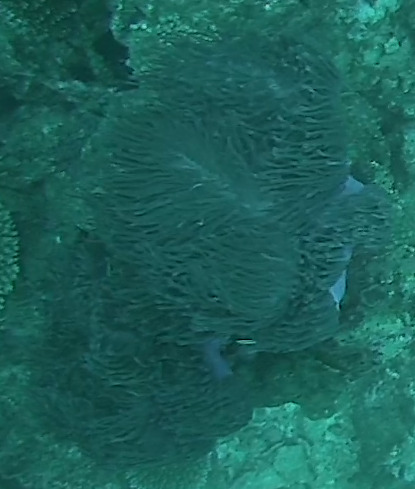
Addu, 30 m.

**Figure 49a.**
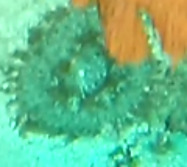
Laamu, 30 m;

**Figure 49b.**
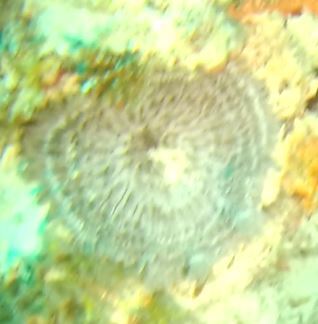
Huvadhu, 30 m;

**Figure 49c.**
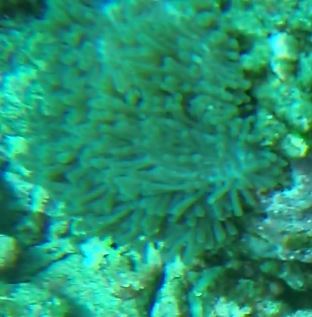
Huvadhu, 10 m.

**Figure 51a.**
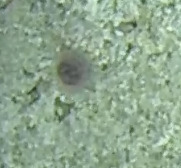
Huvadhu, 250 m;

**Figure 51b.**
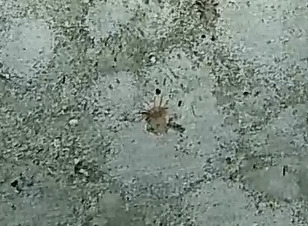
North Male’, 250 m.

**Figure 52a.**
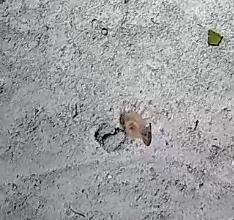
Huvadhu, 490 m;

**Figure 52b.**
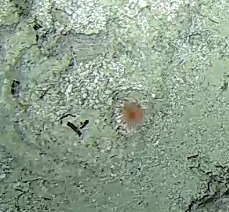
Addu, 250 m.

**Figure 53a.**
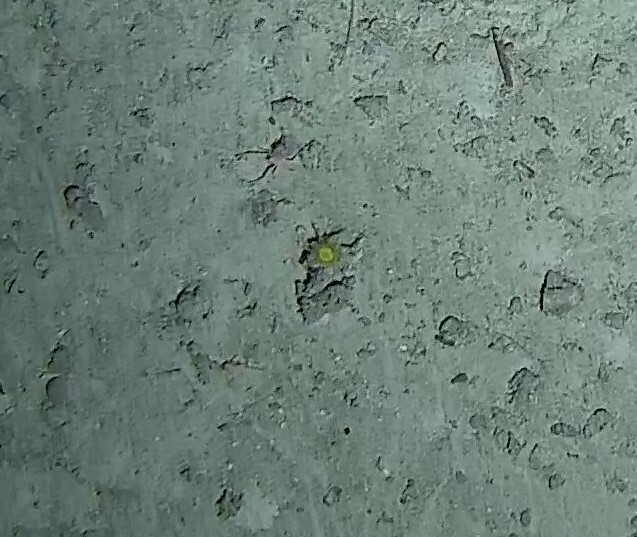
Addu, 490 m;

**Figure 53b.**
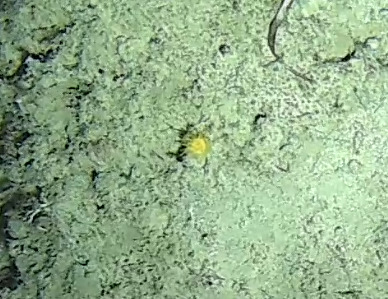
North Male’, 250 m.

**Figure 54a.**
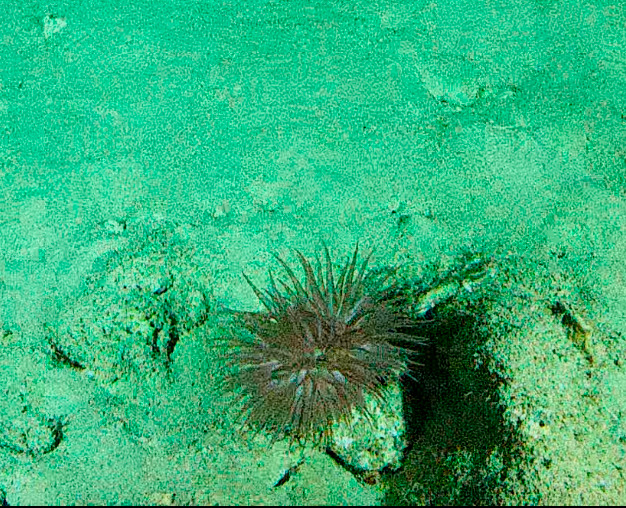
Vaavu, 250 m;

**Figure 54b.**
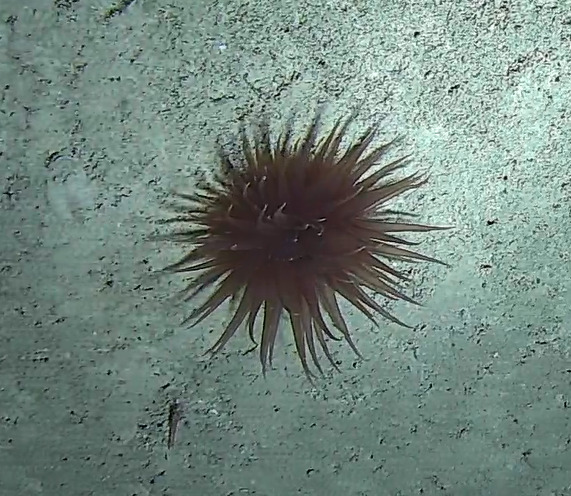
North Male’, 490 m.

**Figure 55.**
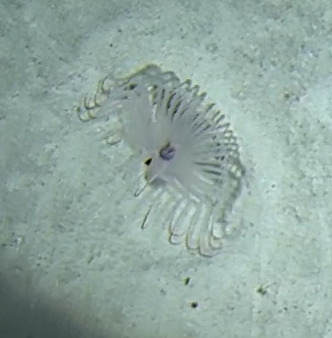
Actiniaria fam. indet. sp. 7, Addu, 490 m.

**Figure 56.**
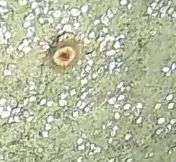
Actiniaria fam. indet. sp. 8, Laamu, 490 m.

**Figure 57a.**
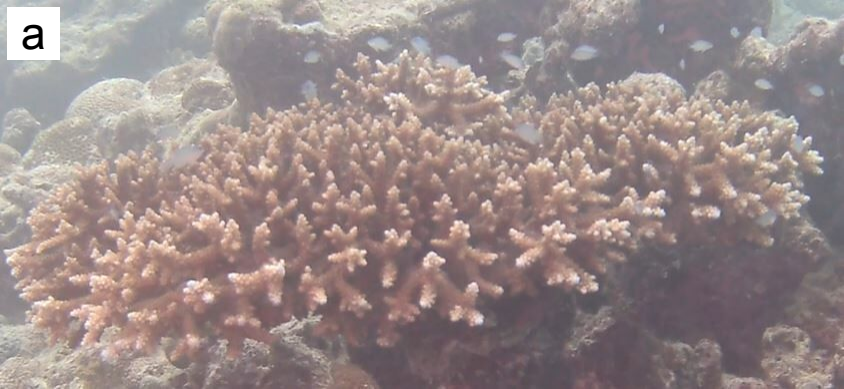
Vaavu, 10 m;

**Figure 57b.**
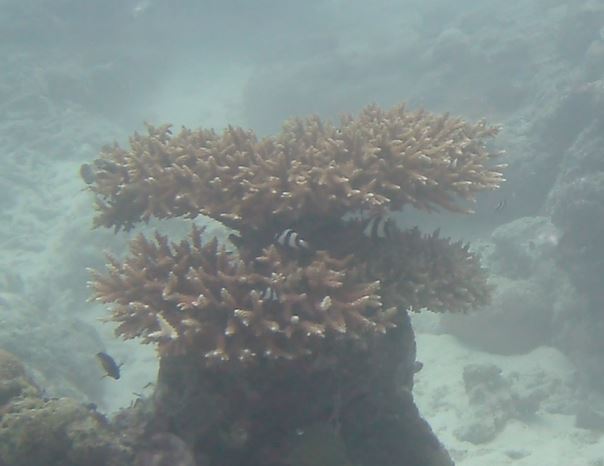
Vaavu, 10 m.

**Figure 58a.**
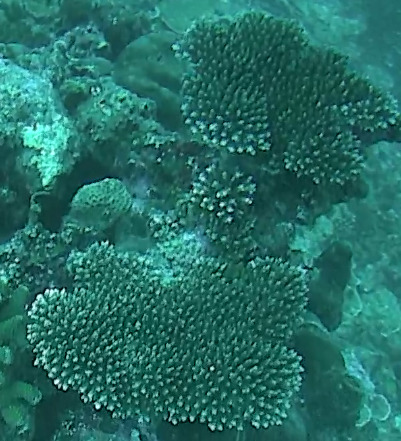
Addu, 30 m;

**Figure 58b.**
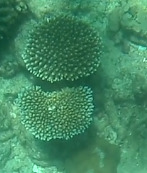
Addu, 2 m.

**Figure 59a.**
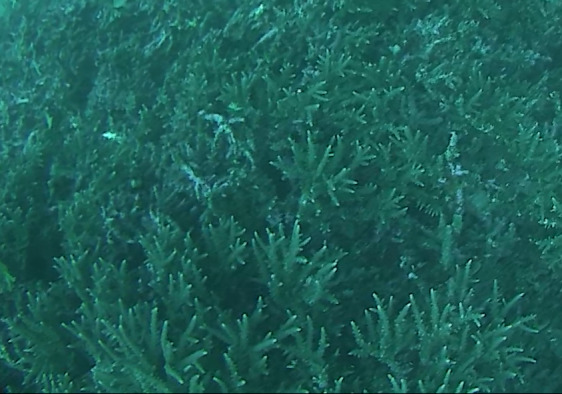
Addu, 30 m;

**Figure 59b.**
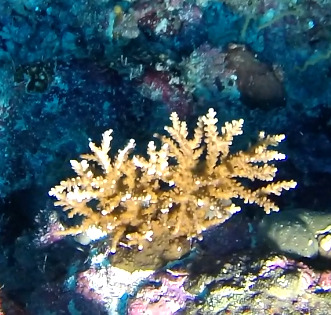
Vaavu, 30 m.

**Figure 60a.**
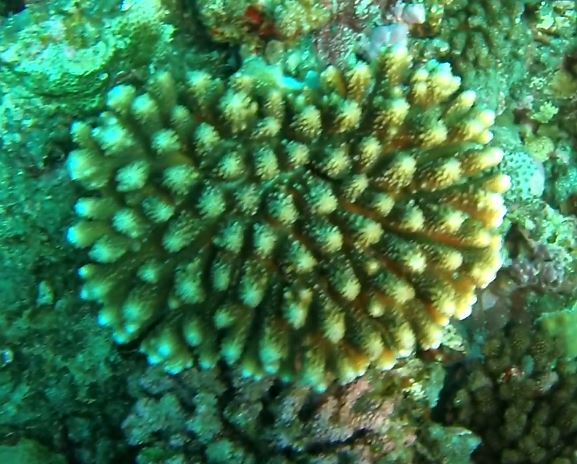
Huvadhu, 10 m;

**Figure 60b.**
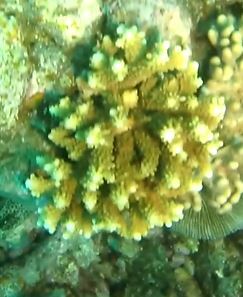
Huvadhu, 10 m.

**Figure 61.**
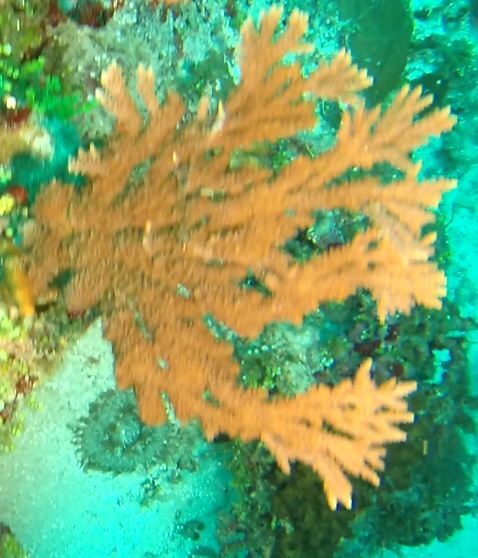
*Acropora* sp. indet. 5, Laamu, 30 m.

**Figure 62.**
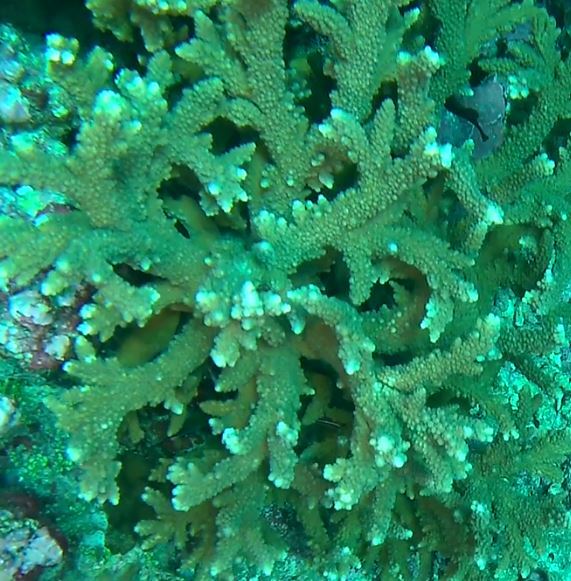
*Acropora* sp. indet. 6, Laamu, 10 m.

**Figure 63a.**
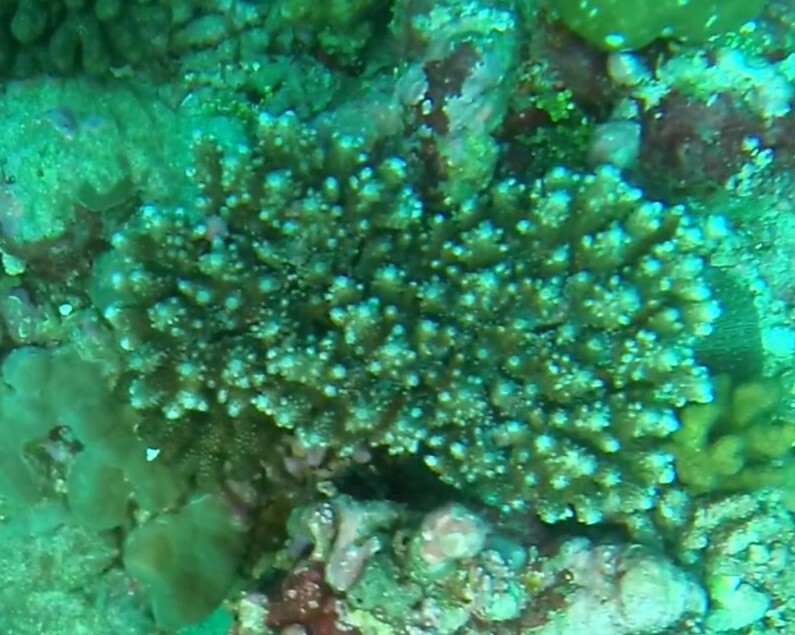
Laamu, 30 m;

**Figure 63b.**
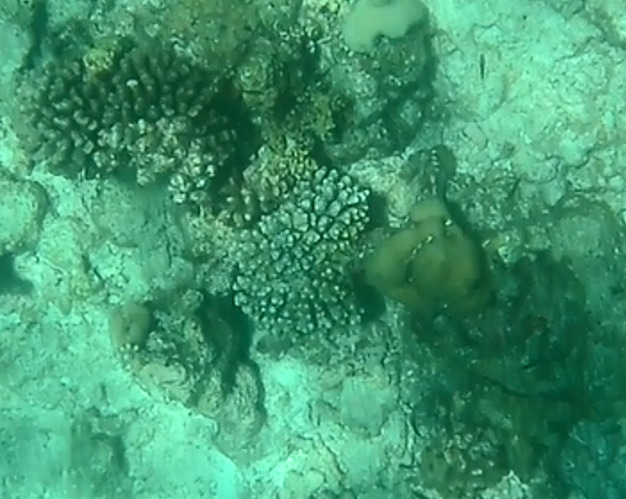
Addu, 2 m.

**Figure 64a.**
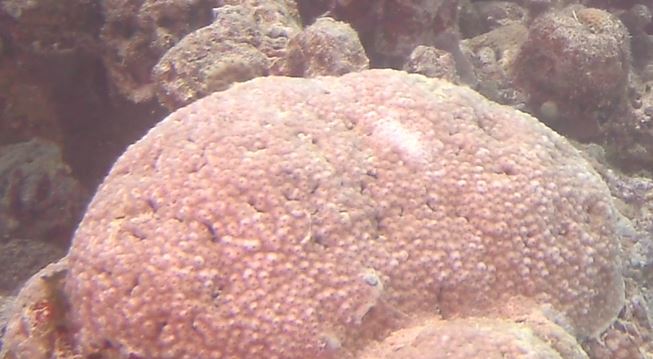
Vaavu, 10 m;

**Figure 64b.**
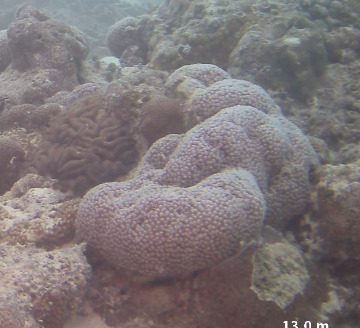
Vaavu, 13 m;

**Figure 64c.**
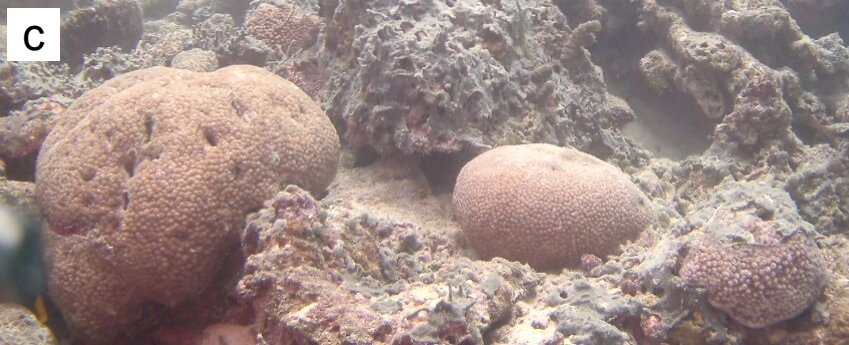
Vaavu, 10 m.

**Figure 65a.**
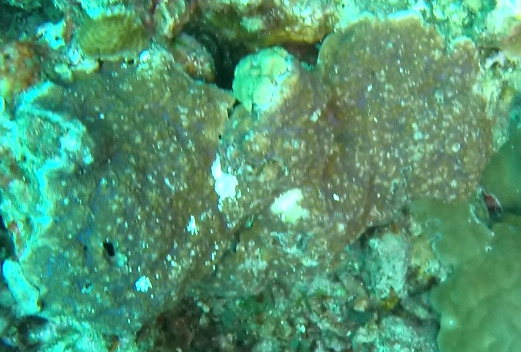
North Male', 10 m;

**Figure 65b.**
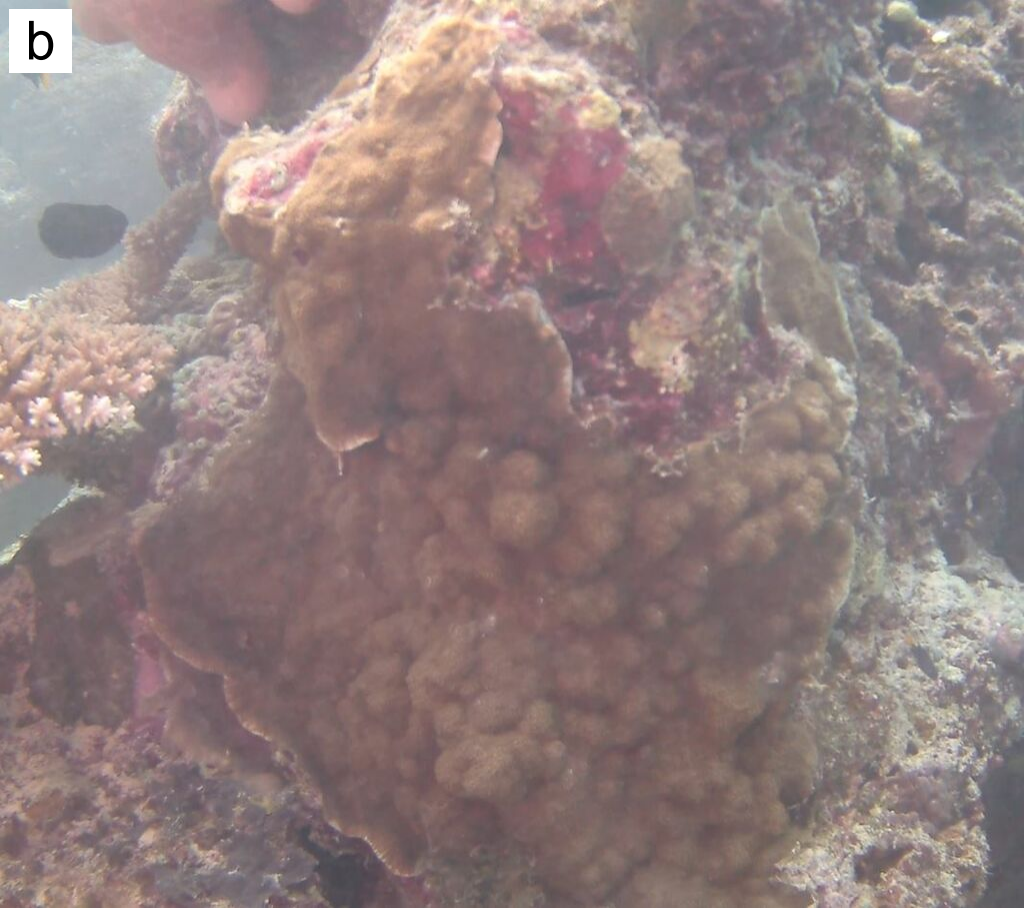
Vaavu, 10 m.

**Figure 66a.**
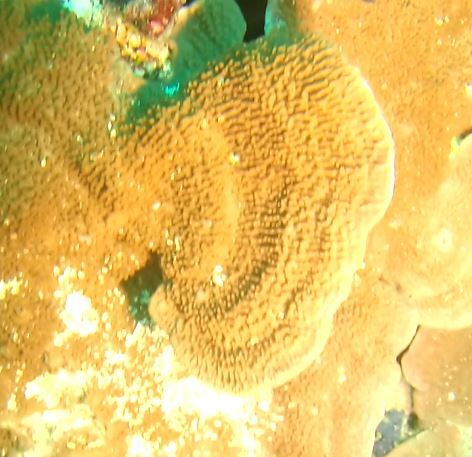
Huvadhu, 30 m;

**Figure 66b.**
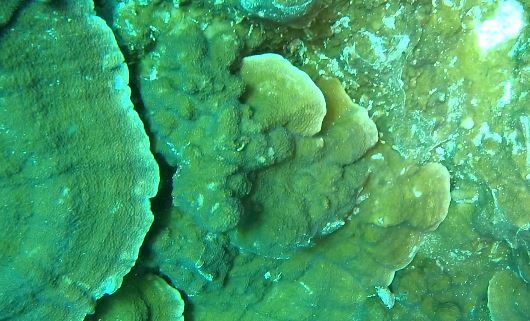
Fuvahmulah, 10 m.

**Figure 68a.**
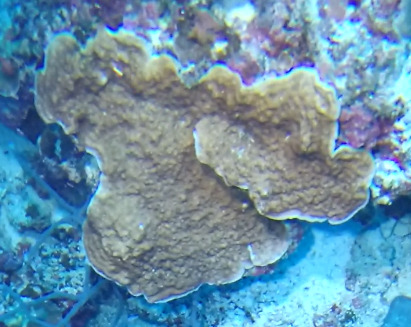
Vaavu, 30 m;

**Figure 68b.**
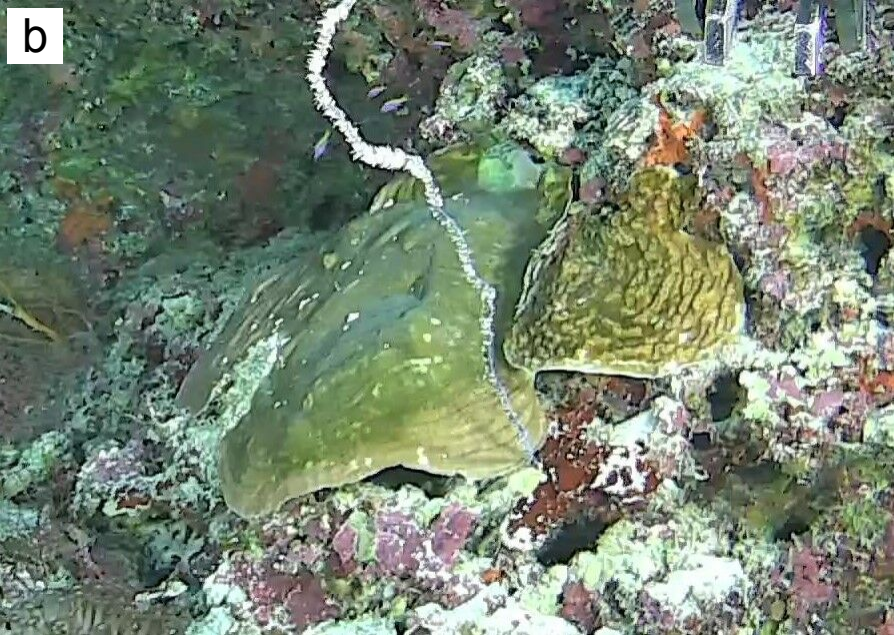
Addu, 60 m.

**Figure 69a.**
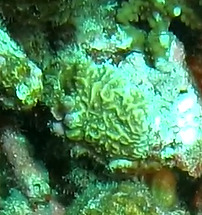
Huvadhu, 10 m;

**Figure 69b.**
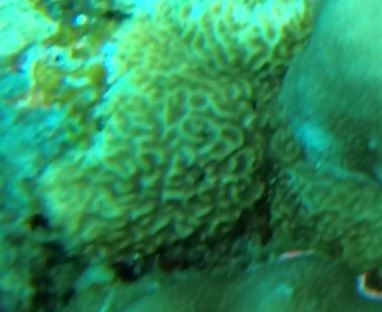
Huvadhu, 10 m.

**Figure 70a.**
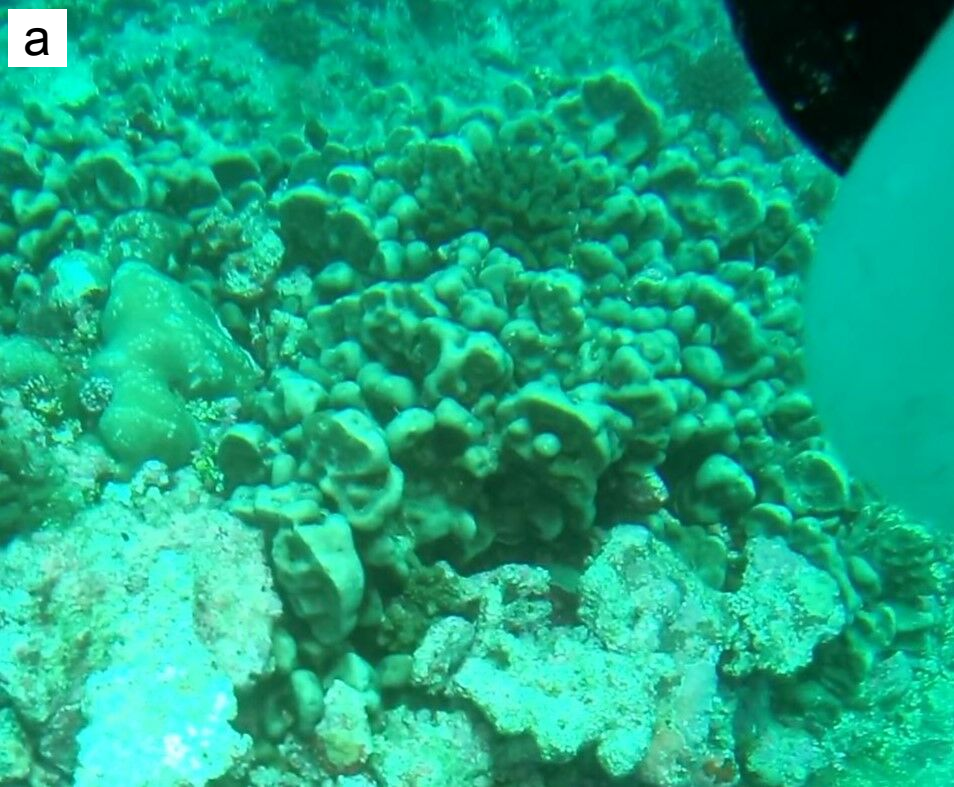
Laamu, 10 m;

**Figure 70b.**
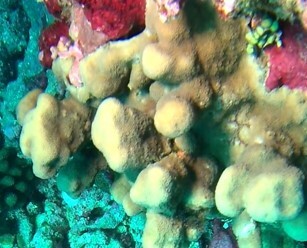
Vaavu, 10 m.

**Figure 71a.**
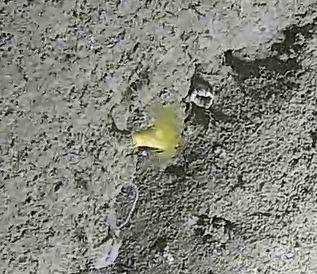
North Male’, 490 m;

**Figure 71b.**
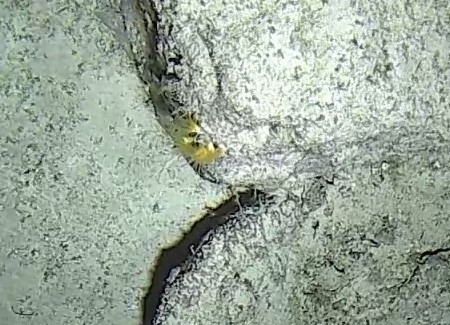
Fuvahmulah, 490 m.

**Figure 72.**
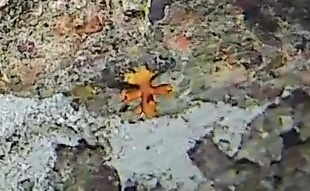
*Tubastraea* sp. indet., North Male’, 60 m.

**Figure 73a.**
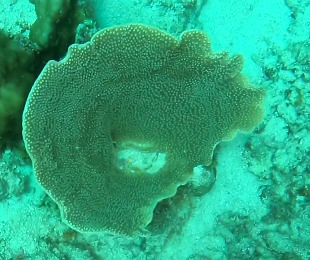
North Male’, 10 m;

**Figure 73b.**
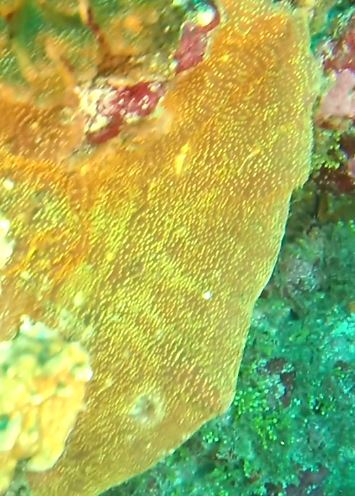
Laamu, 30 m.

**Figure 75.**
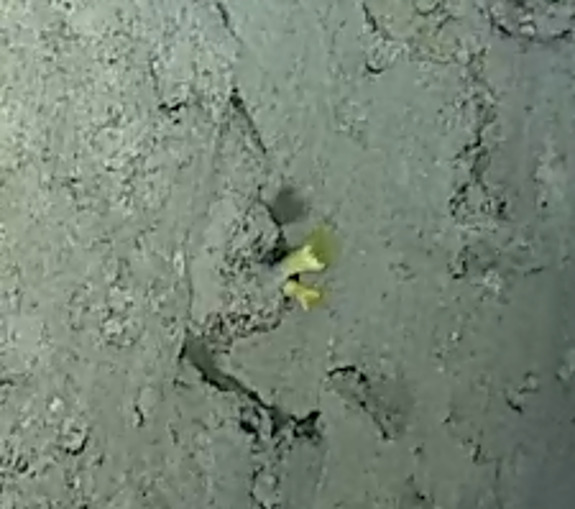
*Balanophyllia* sp. indet., Huvadhu, 490 m.

**Figure 76a.**
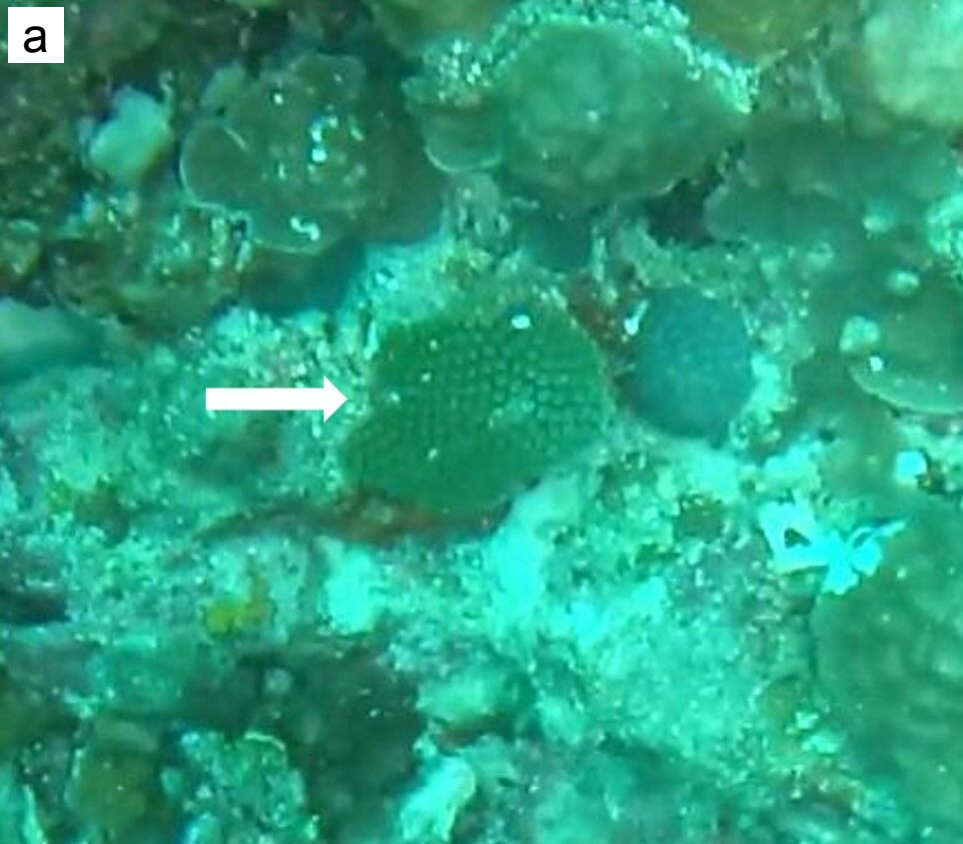
Vaavu, 10 m;

**Figure 76b.**
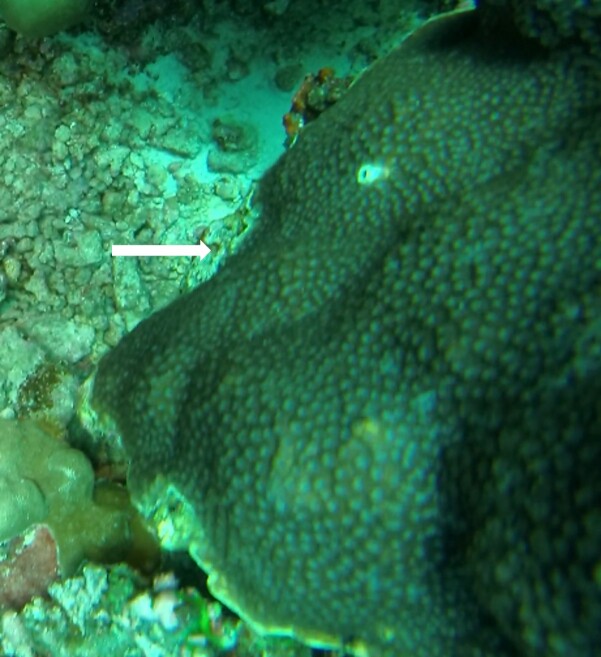
Huvadhu, 10 m.

**Figure 77.**
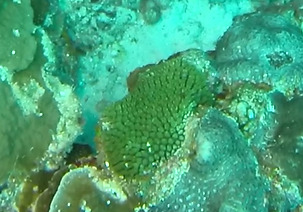
*Galaxea* sp. indet., Vaavu, 10 m.

**Figure 78a.**
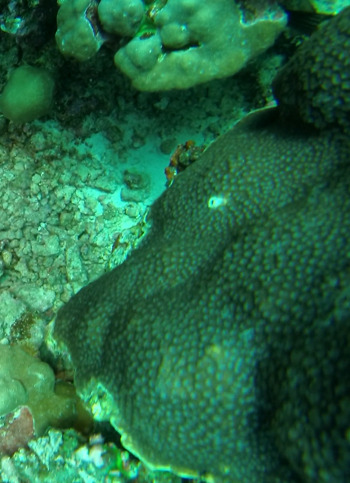
Huvadhu, 10 m;

**Figure 78b.**
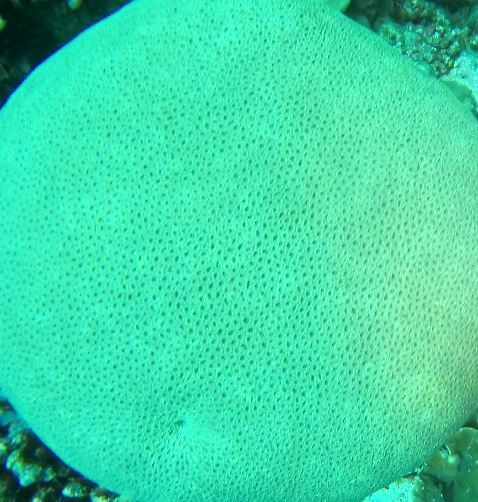
Fuvahmulah, 10 m;

**Figure 78c.**
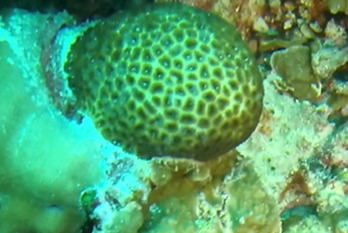
Vaavu, 10 m.

**Figure 80a.**
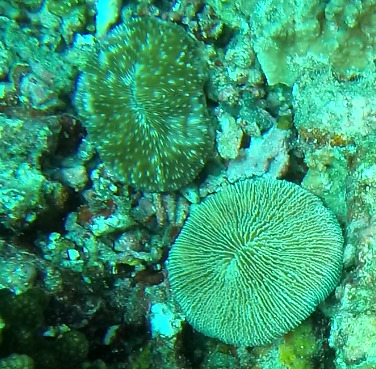
*F. lobactis*, *F. danafungia* North Male’, 30 m;

**Figure 80b.**
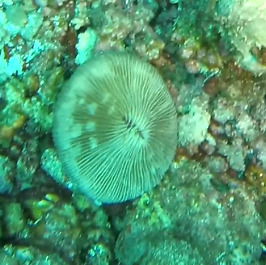
*F. cycloseris*, North Male’, 10 m;

**Figure 80c.**
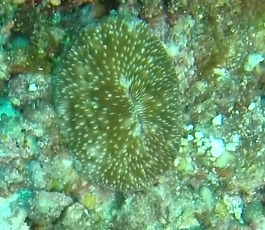
*F. lobactis*, North Male’, 10 m.

**Figure 81a.**
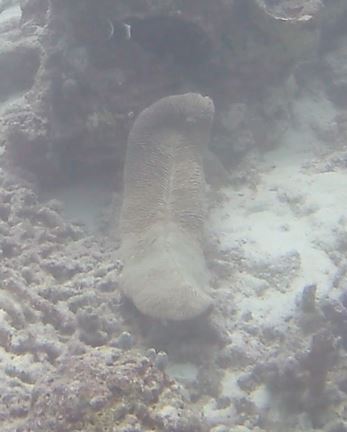
Vaavu, 10 m;

**Figure 81b.**
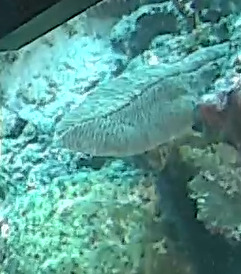
Addu, 28 m.

**Figure 82a.**
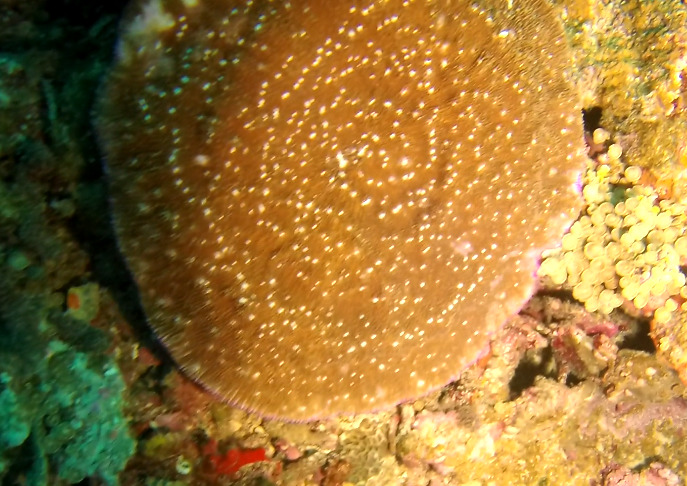
Addu, 30 m;

**Figure 82b.**
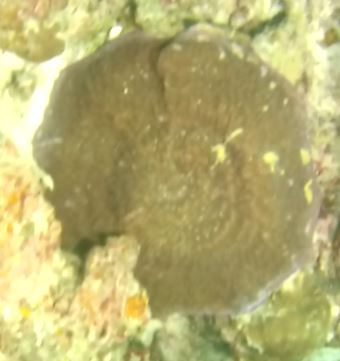
Addu, 30 m.

**Figure 83a.**
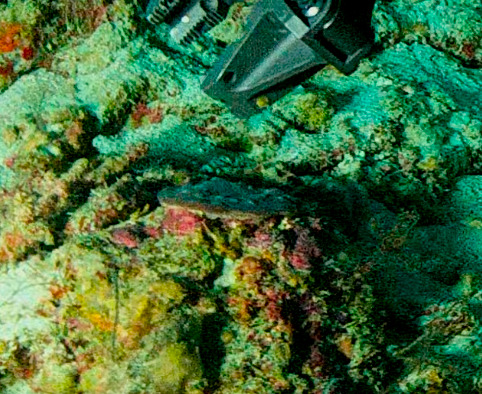
Vaavu, 65 m, *in situ* photo of MAL1_638;

**Figure 83b.**
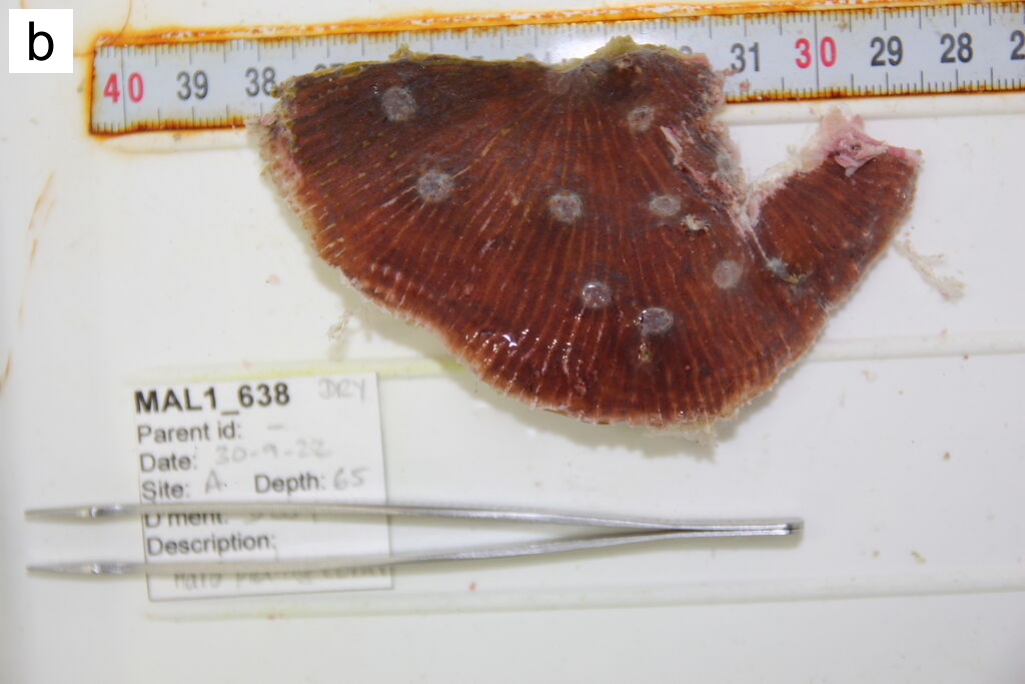
Vaavu, 65 m, collected specimen MAL1_638.

**Figure 84a.**
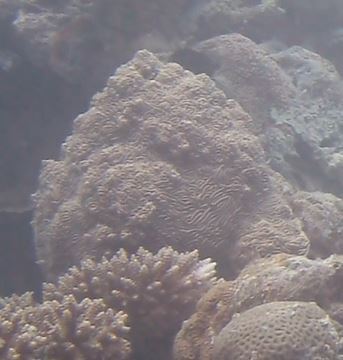
Vaavu, 10 m;

**Figure 84b.**
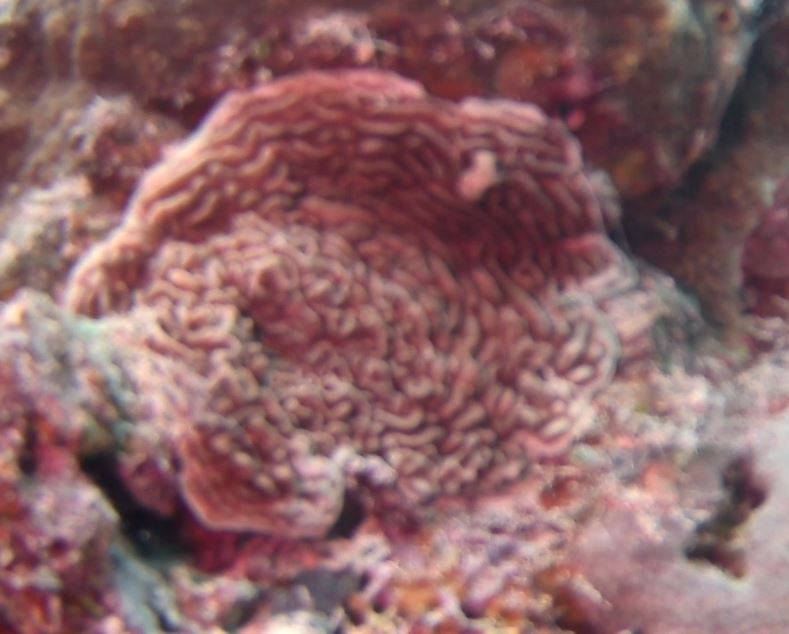
Vaavu, 10 m.

**Figure 85a.**
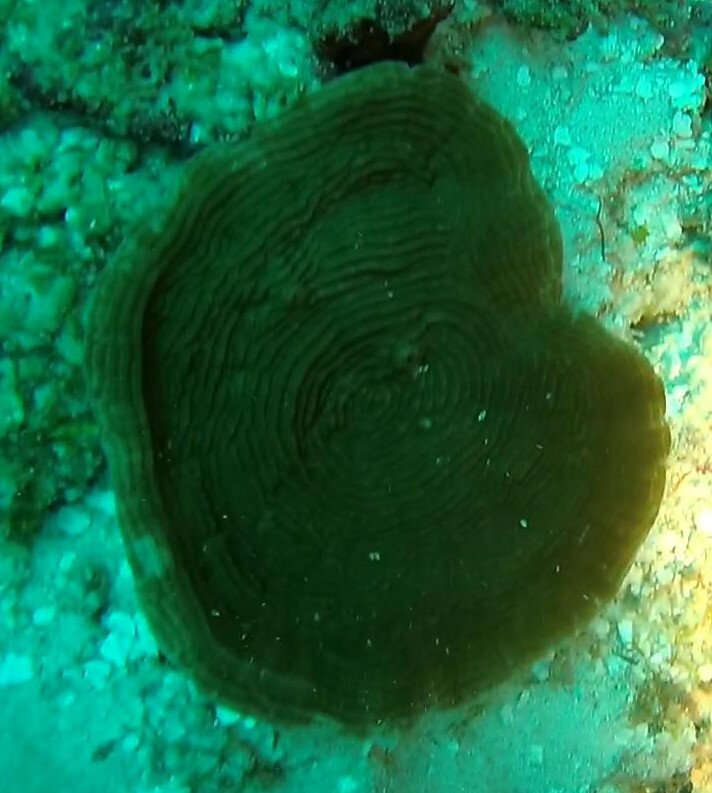
Laamu, 30 m;

**Figure 85b.**
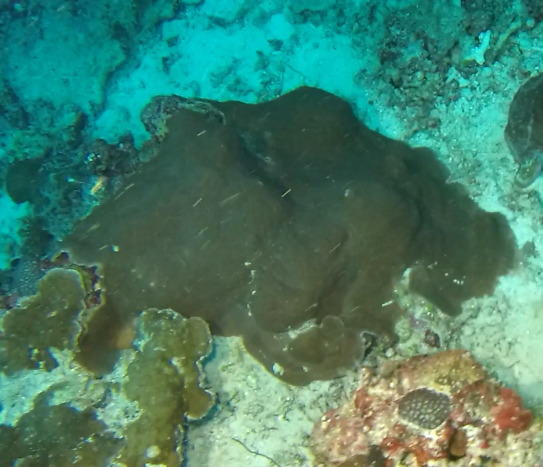
Vaavu, 30 m.

**Figure 86a.**
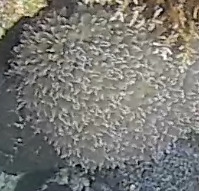
Addu, 30 m;

**Figure 86b.**
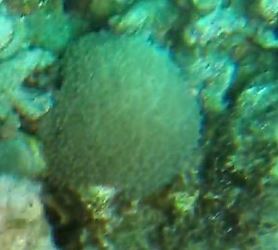
Addu, 10 m.

**Figure 88a.**
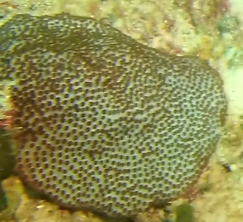
Huvadhu, 30 m;

**Figure 88b.**
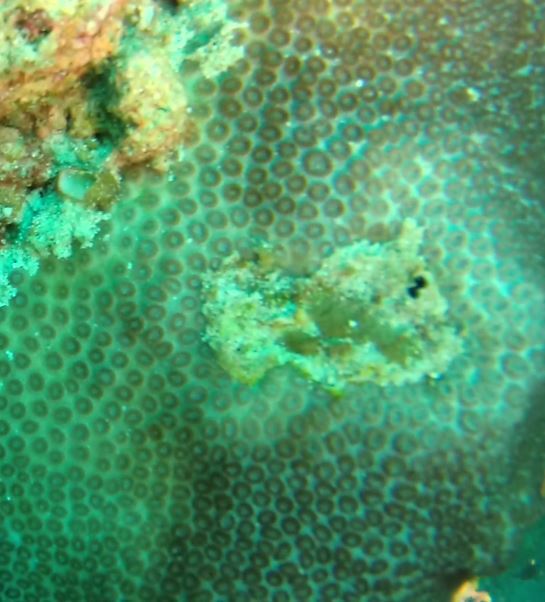
Huvadhu, 30 m.

**Figure 89a.**
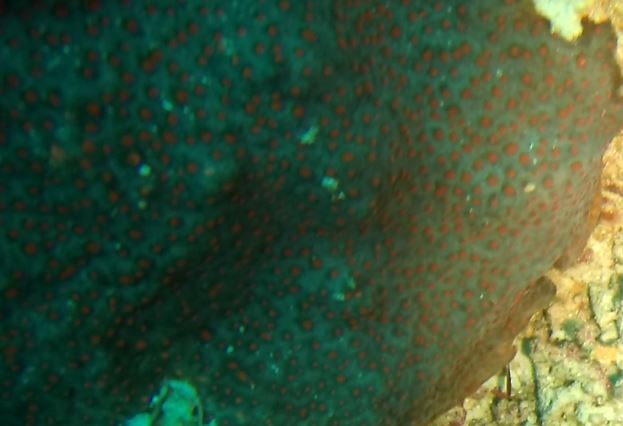
Huvadhu, 30 m;

**Figure 89b.**
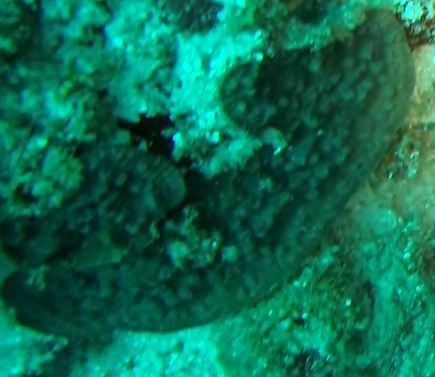
Laamu, 30 m.

**Figure 90a.**
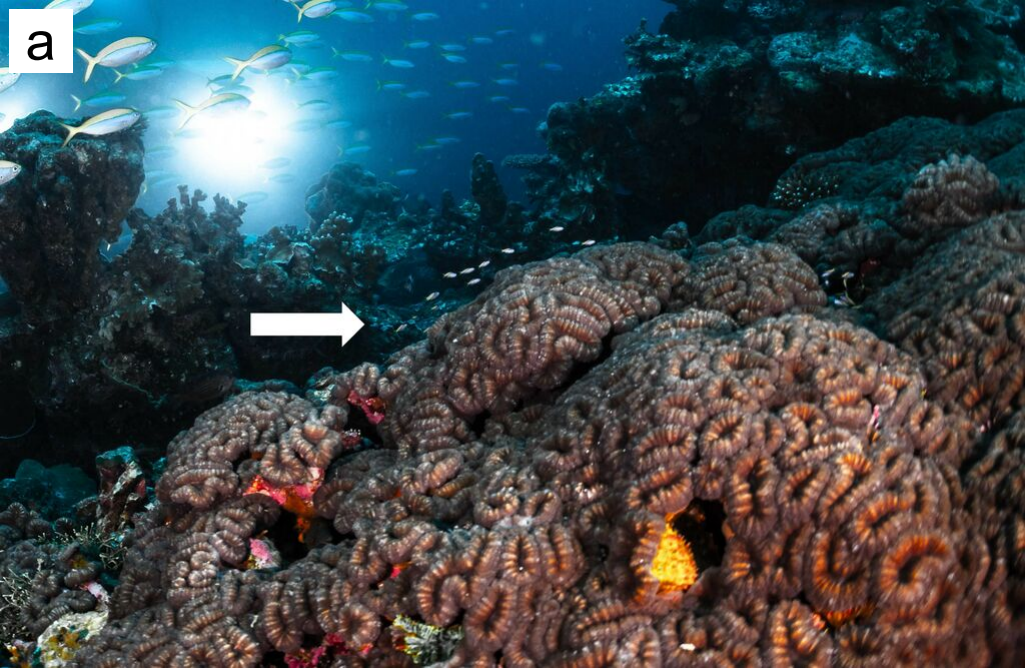
Vaavu, 10-30 m;

**Figure 90b.**
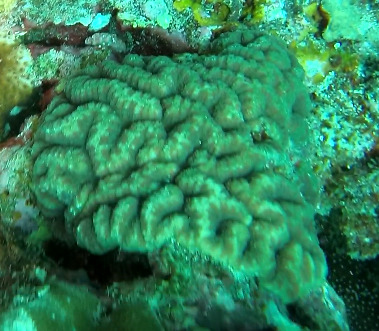
North Male’, 10 m;

**Figure 90c.**
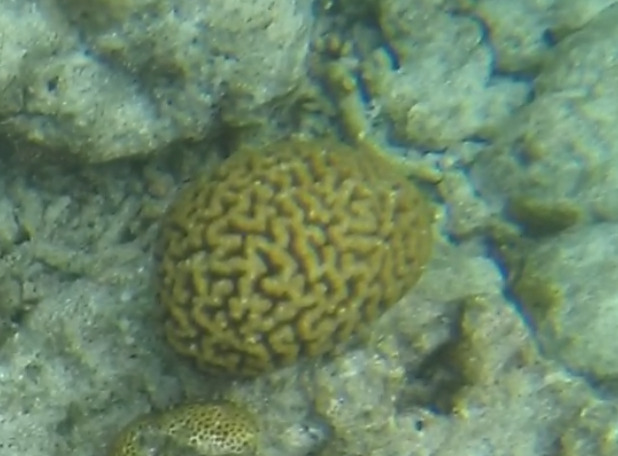
Vaavu, 2 m;

**Figure 90d.**
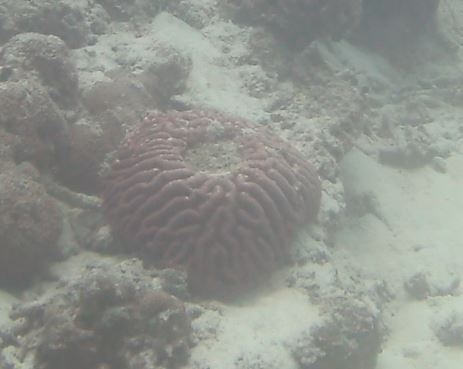
Vaavu, 10 m.

**Figure 91a.**
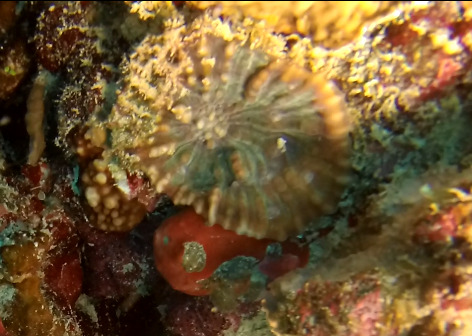
Vaavu, 30 m;

**Figure 91b.**
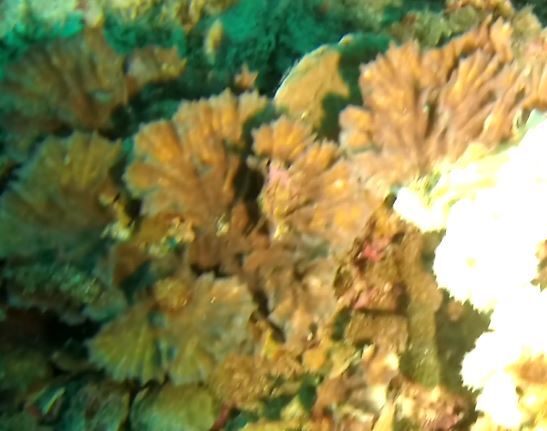
Addu, 30 m.

**Figure 92a.**
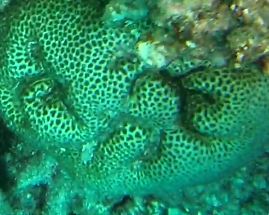
Fuvahmulah, 10 m;

**Figure 92b.**
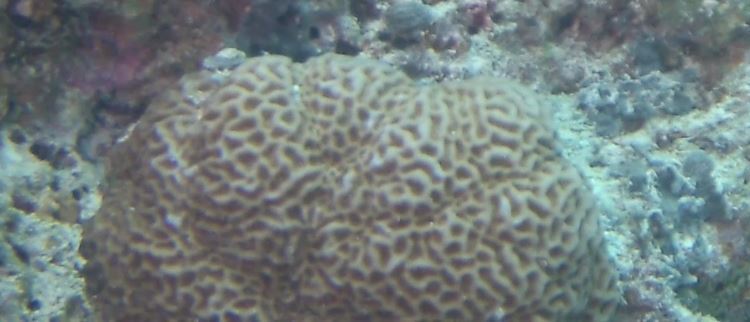
Vaavu, 10 m.

**Figure 93a.**
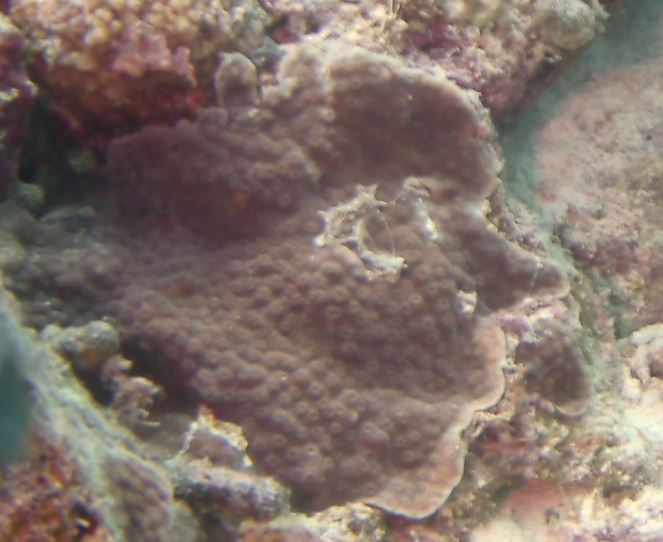
*Echinopora* sp. indet., Vaavu, 10 m;

**Figure 93b.**
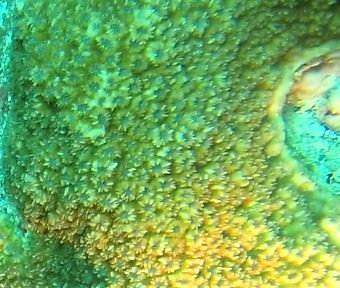
*Echinoporahirsutissima*, Addu, 10 m.

**Figure 94a.**
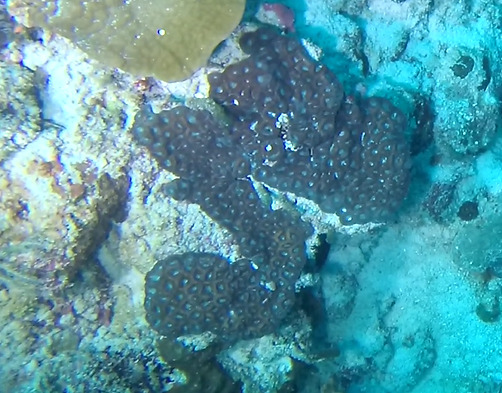
Huvadhu, 30 m;

**Figure 94b.**
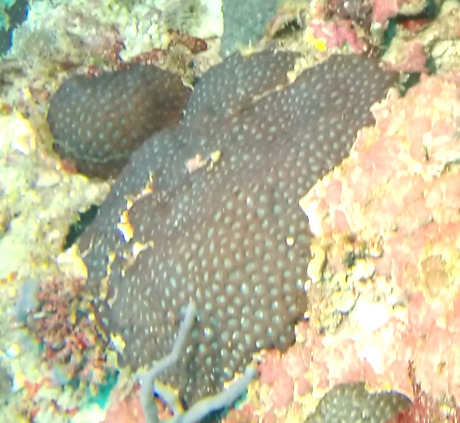
Vaavu, 30 m.

**Figure 95a.**
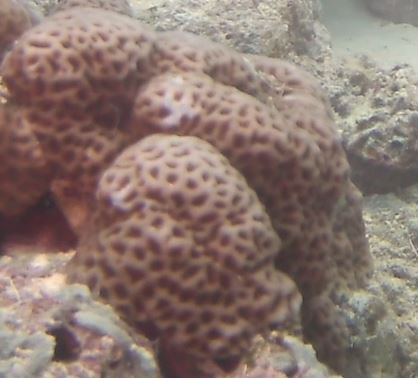


**Figure 95b.**
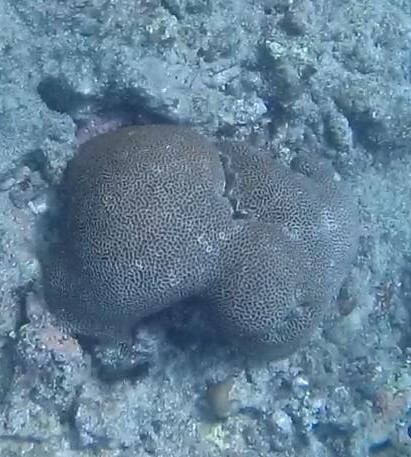
Vaavu, 10 m;

**Figure 95c.**
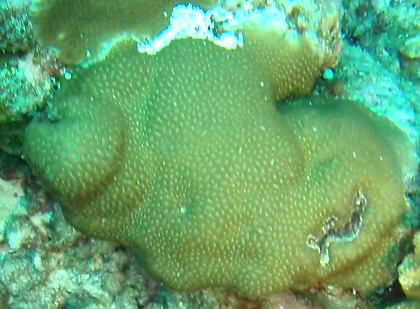
North Male’, 10 m.

**Figure 96.**
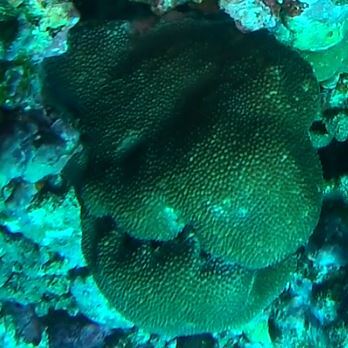
*Hydnophora* sp. indet., Laamu, 10 m.

**Figure 97a.**
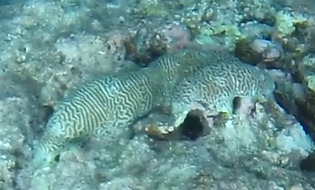
Vaavu, 2 m;

**Figure 97b.**
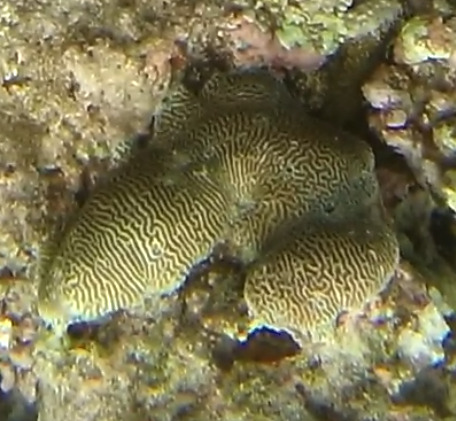
Vaavu, 2 m.

**Figure 98.**
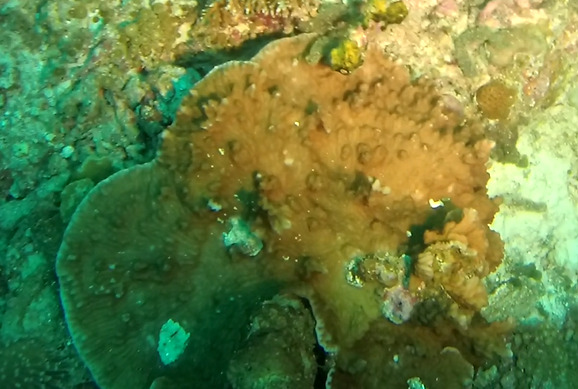
*Merulina* sp. indet., Addu, 30 m.

**Figure 99a.**
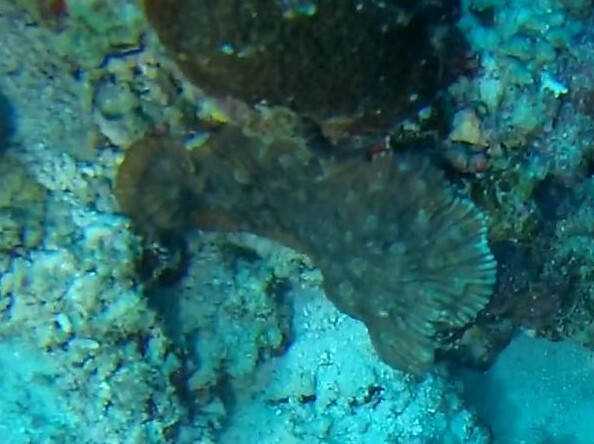
North Male’, 30 m;

**Figure 99b.**
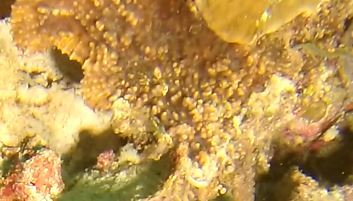
Addu, 30 m.

**Figure 100.**
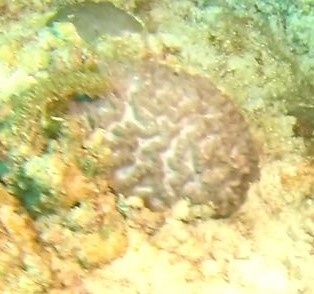
*Oulophyllia* sp. indet., Huvadhu, 30 m.

**Figure 101a.**
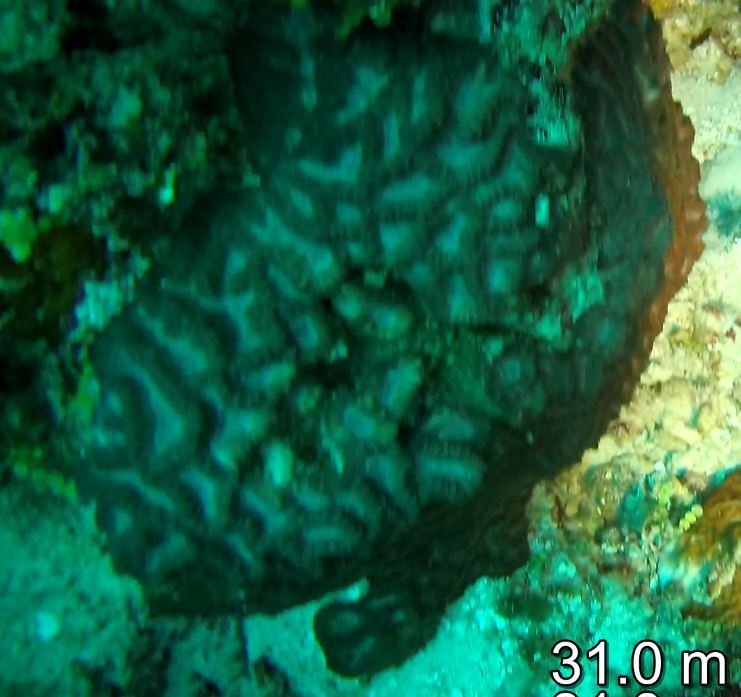
Laamu, 30 m;

**Figure 101b.**
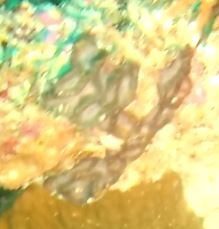
Huvadhu, 3 m.

**Figure 102a.**
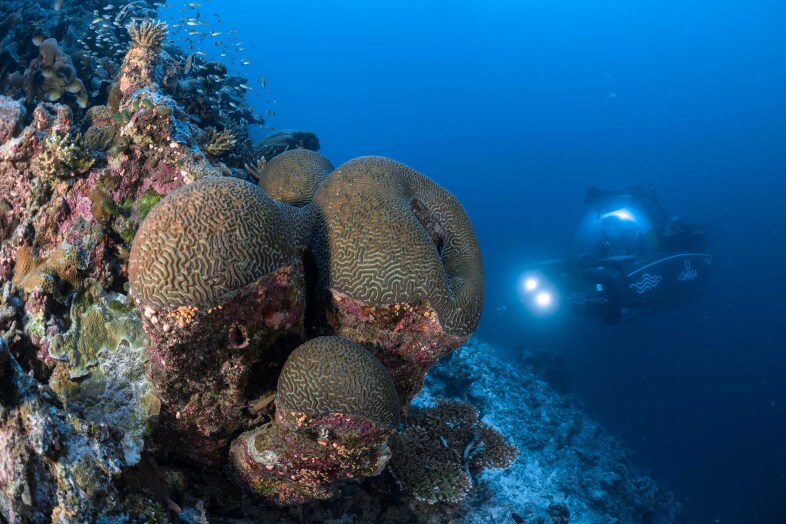
Vaavu, ~10-30 m;

**Figure 102b.**
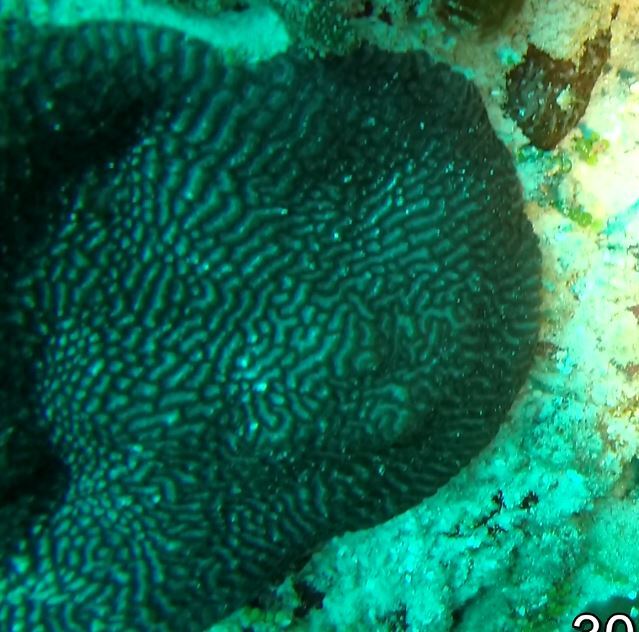
Laamu, 30 m;

**Figure 102c.**
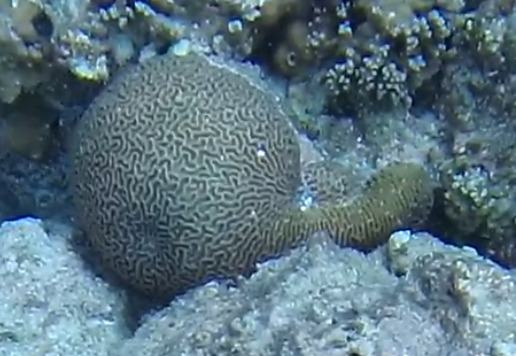
Vaavu, 2 m;

**Figure 102d.**
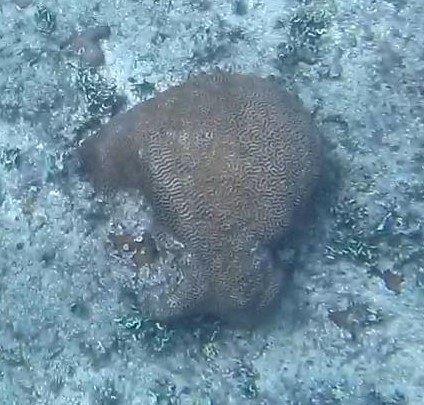
North Male', 5 m.

**Figure 103a.**
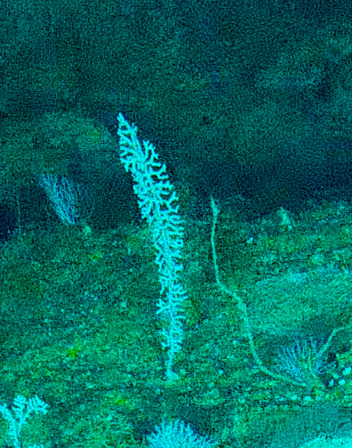
Fuvahmulah, 120 m;

**Figure 103b.**
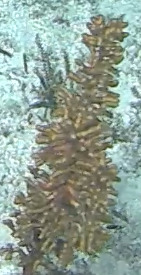
Laamu, 60 m;

**Figure 103c.**
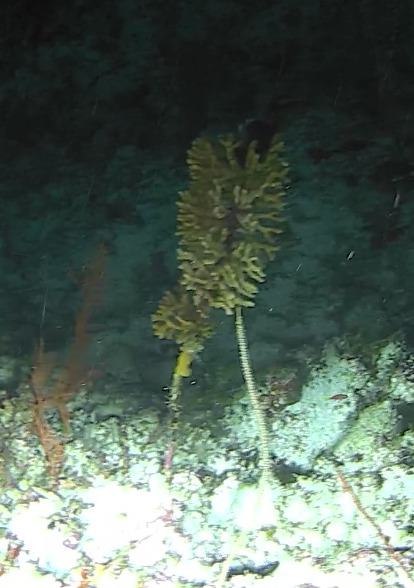
Huvadhu, 120 m.

**Figure 104.**
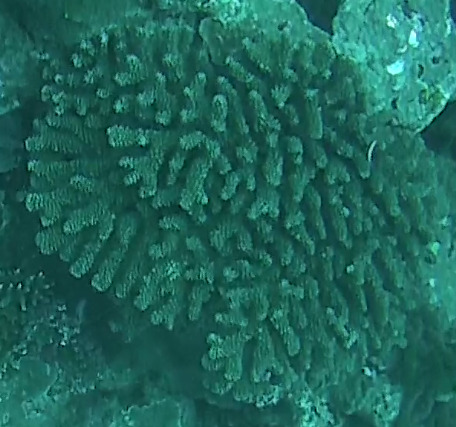
*Pocillopora* sp. indet. 1, Addu, 30 m.

**Figure 105a.**
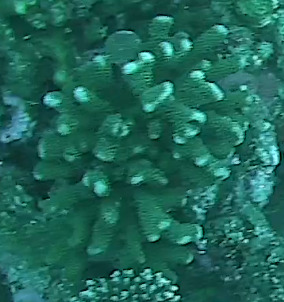
Addu, 30 m;

**Figure 105b.**
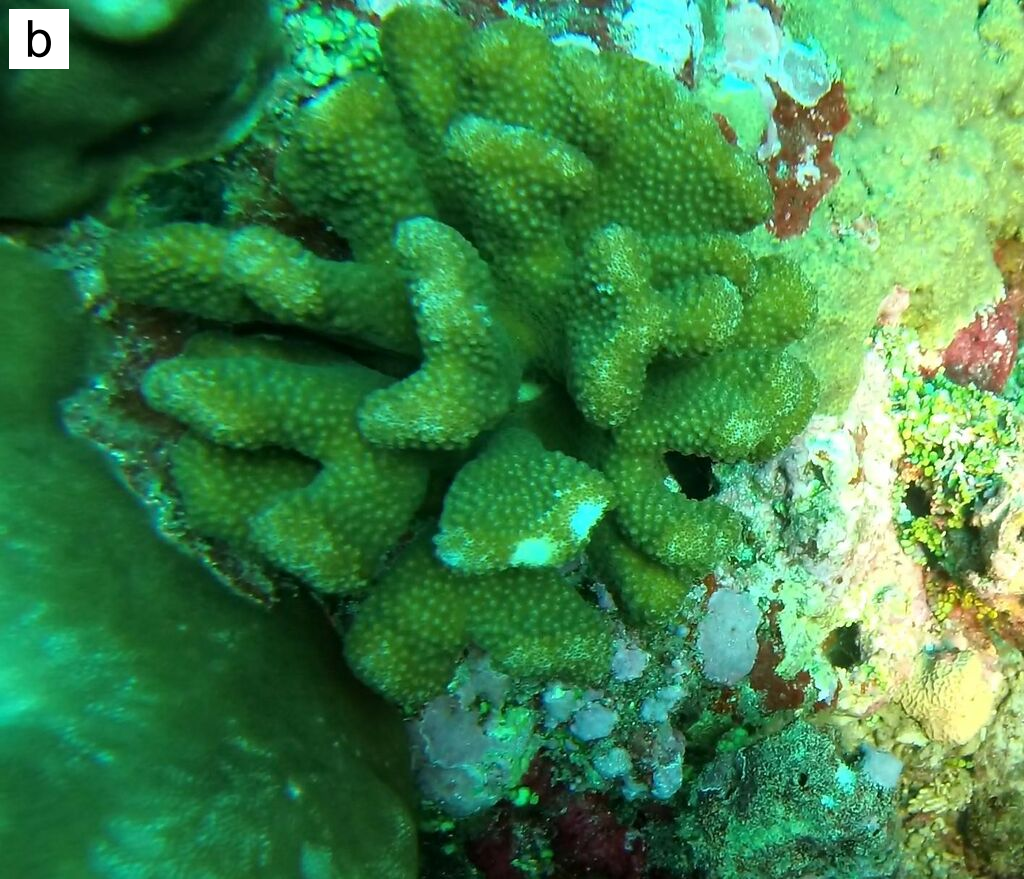
Laamu, 10 m.

**Figure 106a.**
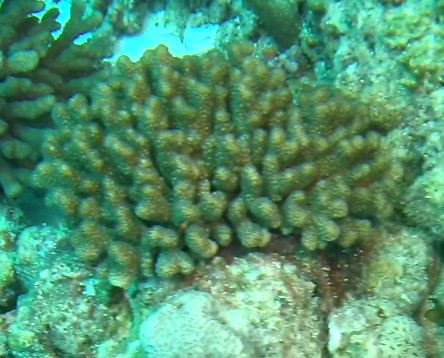
North Male’, 10 m;

**Figure 106b.**
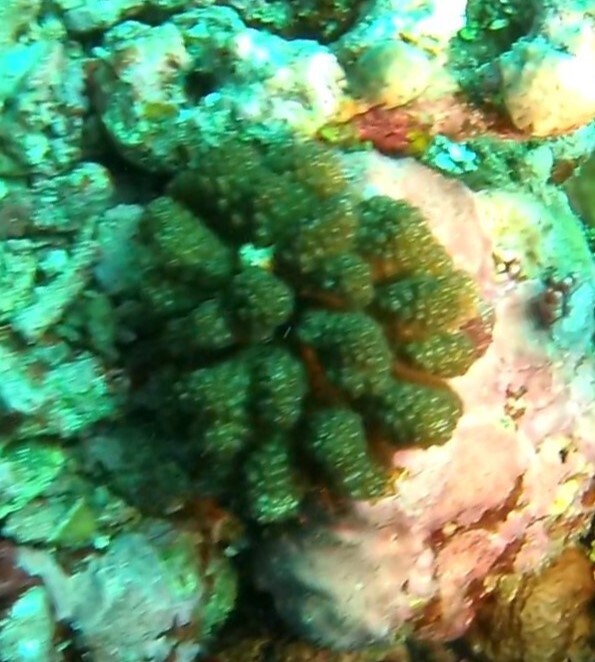
Laamu, 10 m.

**Figure 107a.**
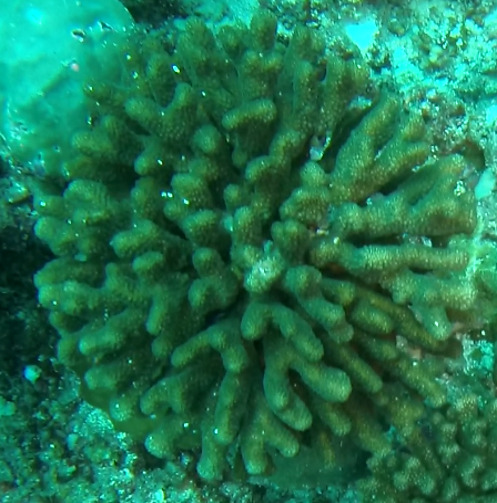
North Male’, 10 m;

**Figure 107b.**
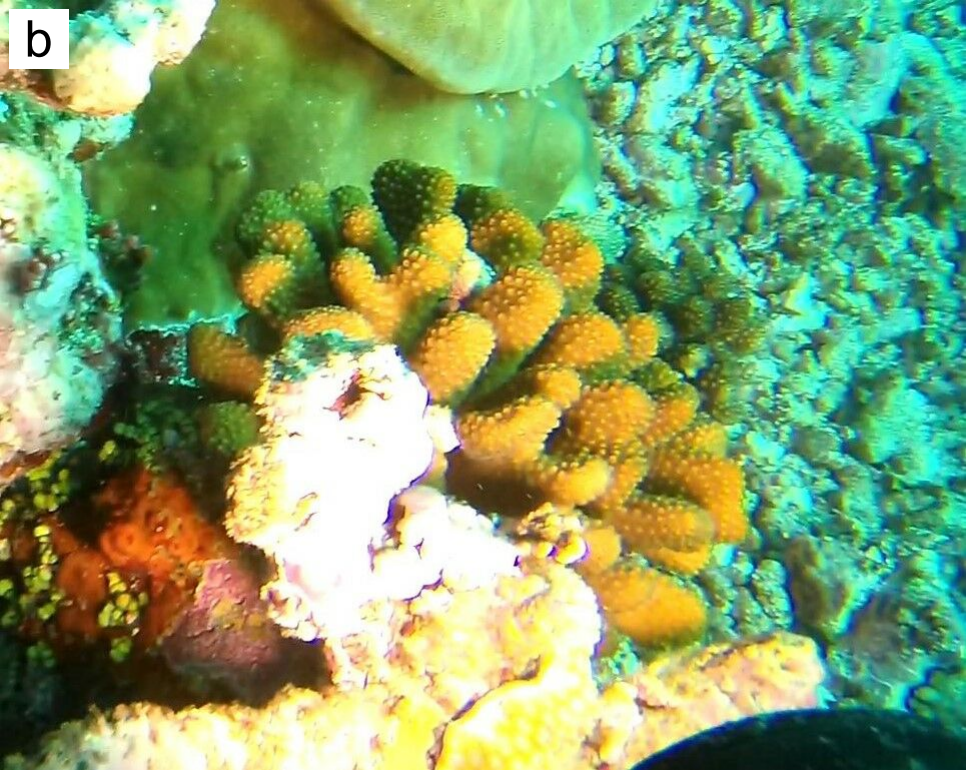
Laamu, 10 m;

**Figure 107c.**
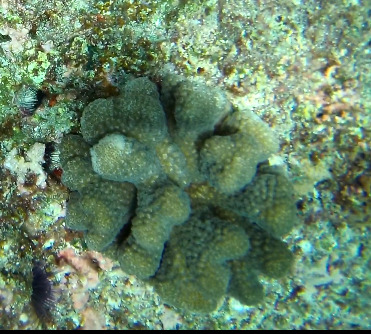
Laamu, 2 m;

**Figure 107d.**
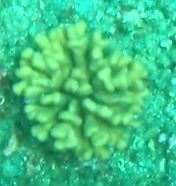
Huvadhu, 10 m.

**Figure 108a.**
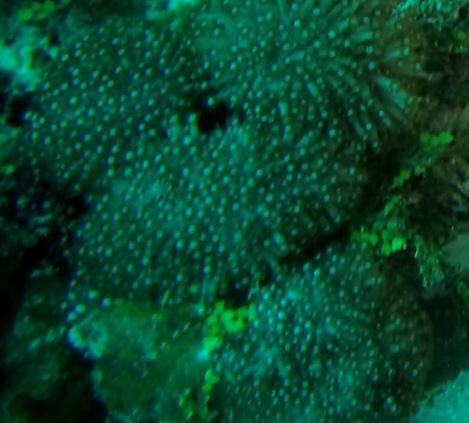
Fuvahmulah, ~ 30-60 m.

**Figure 108b.**
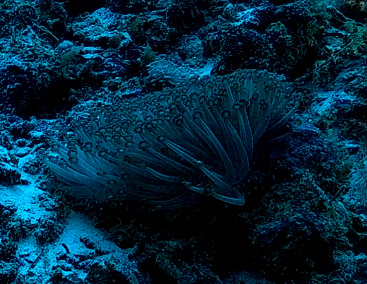


**Figure 109a.**
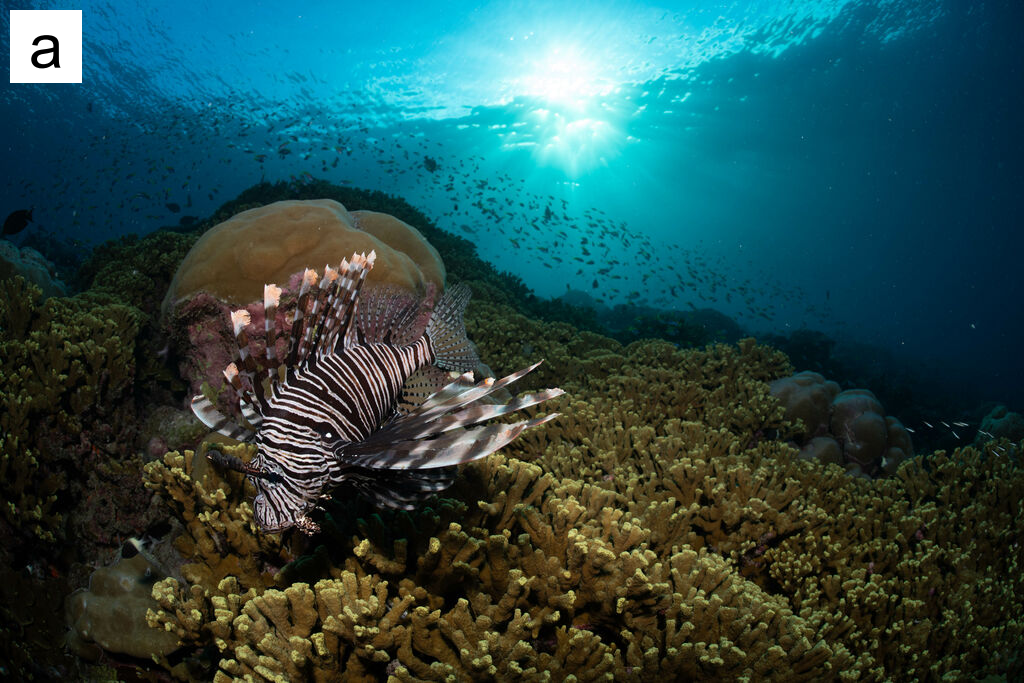
Fuvahmulah, 10-30 m;

**Figure 109b.**
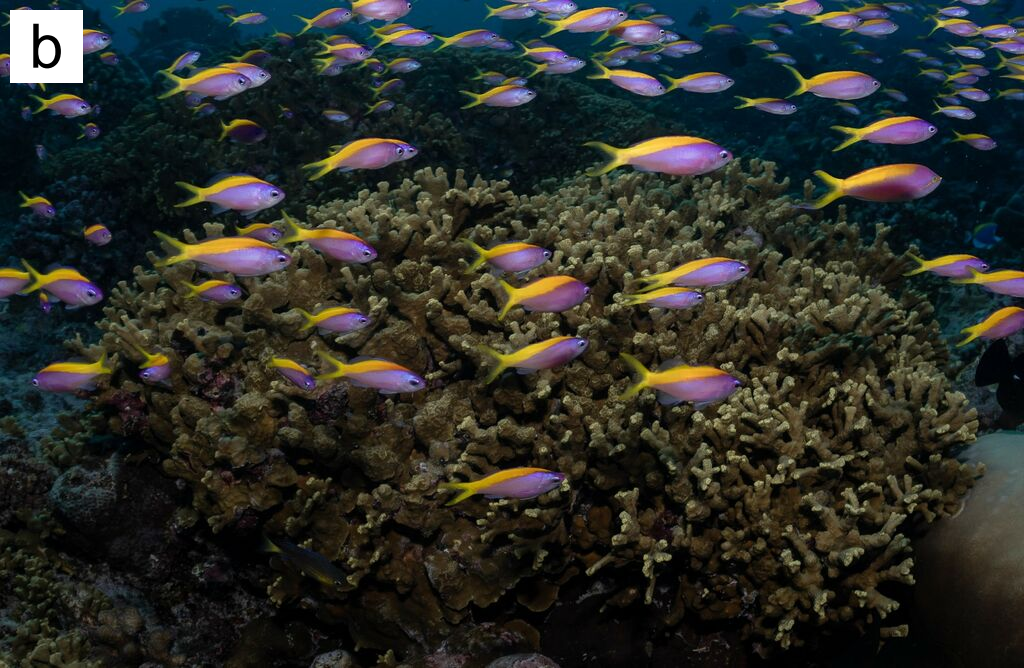
Fuvahmulah, 10-30 m;

**Figure 109c.**
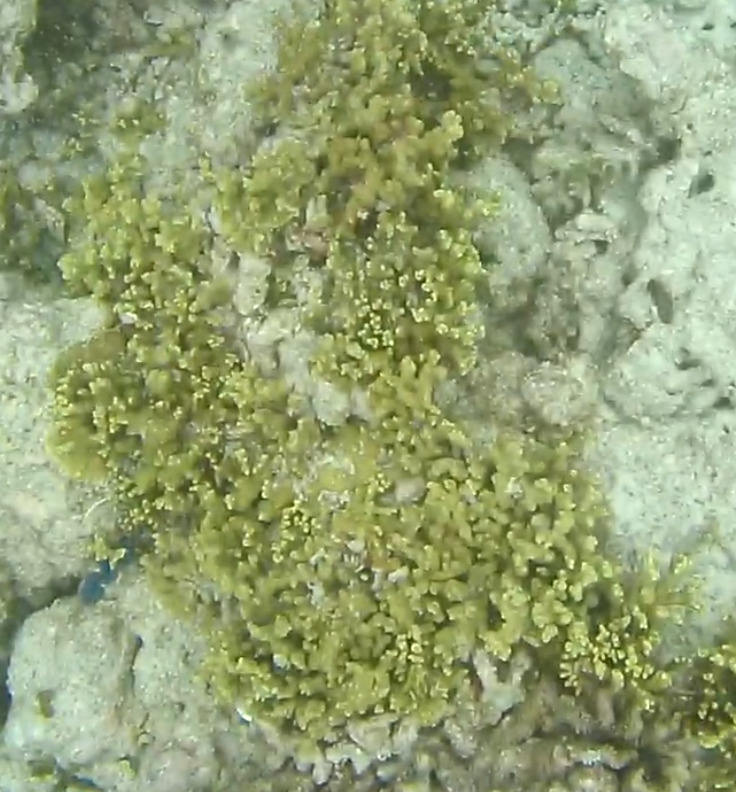
Vaavu, 2 m;

**Figure 109d.**
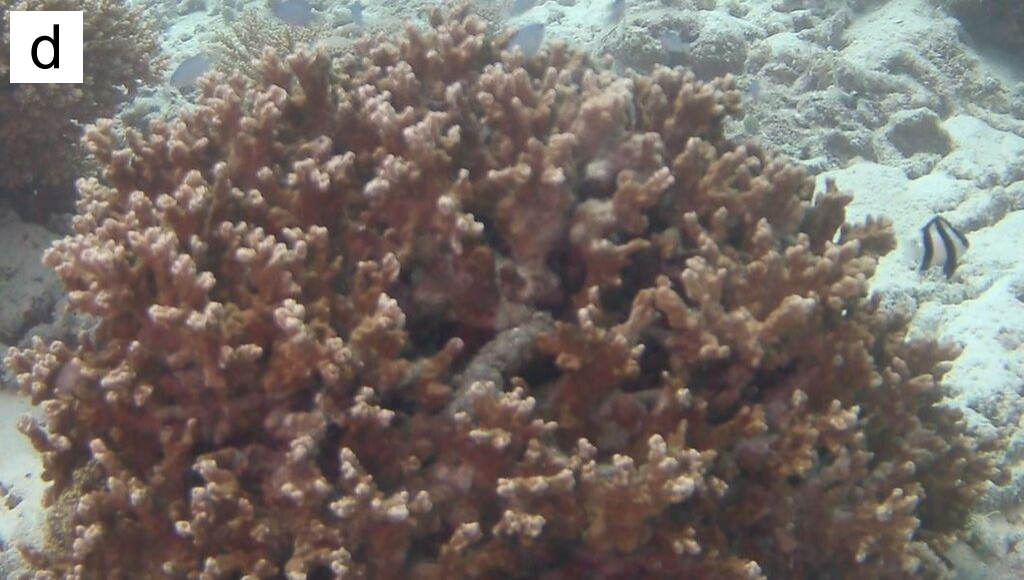
Vaavu, 10 m.

**Figure 110a.**
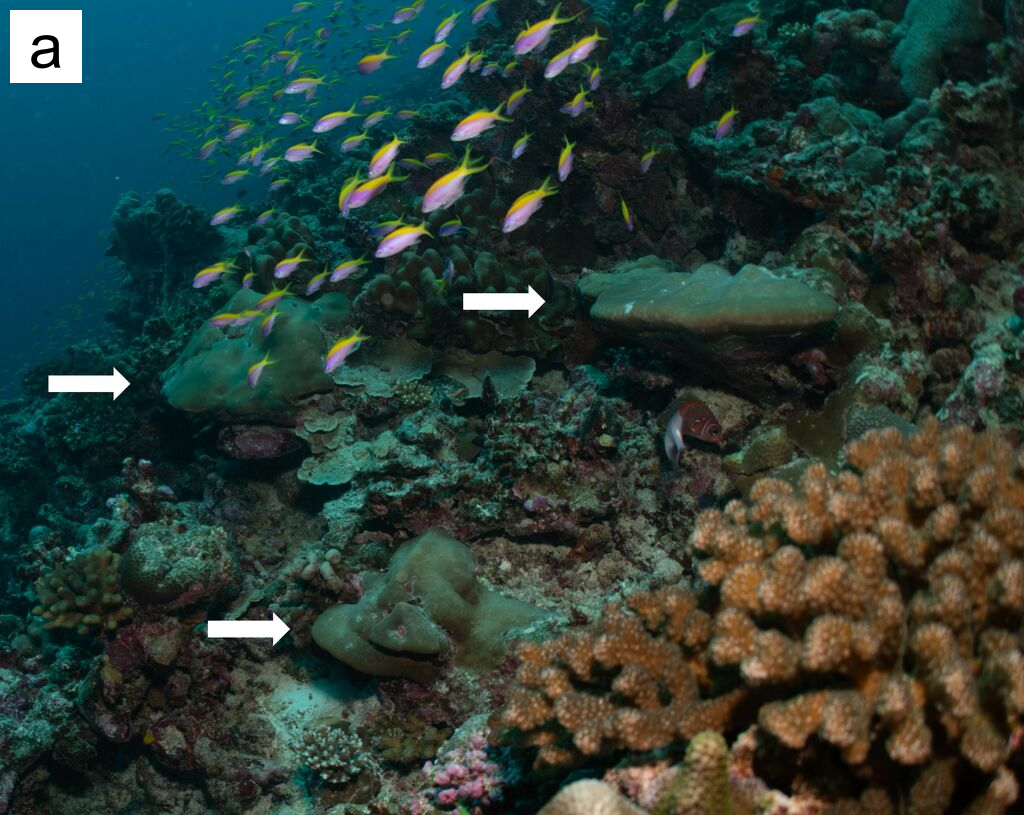
Huvadhu, 10-30 m;

**Figure 110b.**
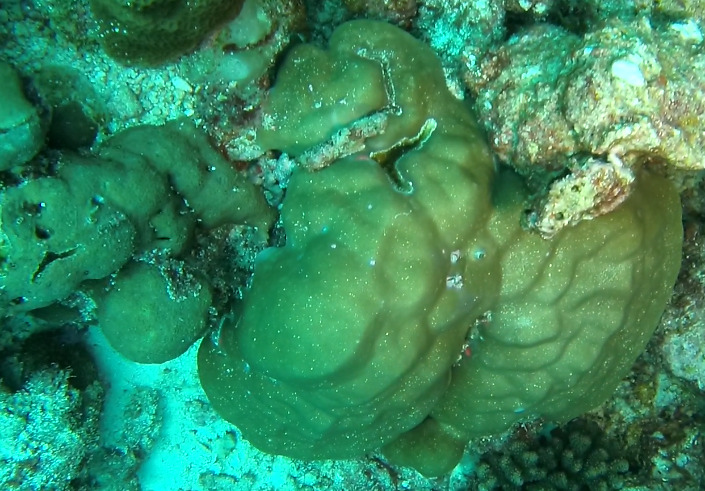
North Male’, 10 m;

**Figure 110c.**
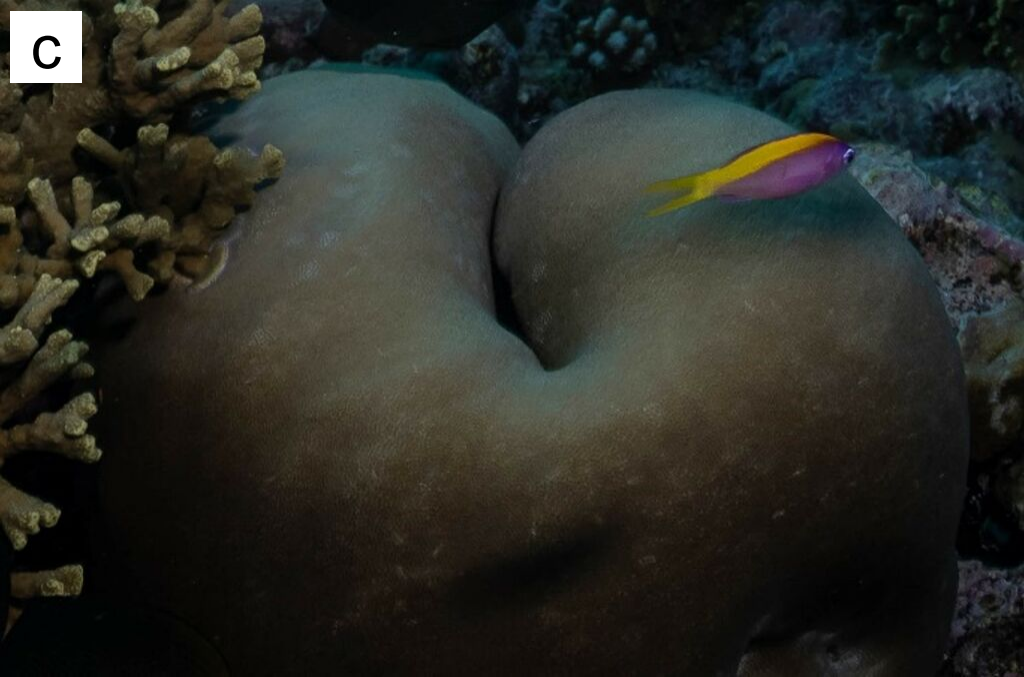
Fuvahmulah, 10-30 m;

**Figure 110d.**
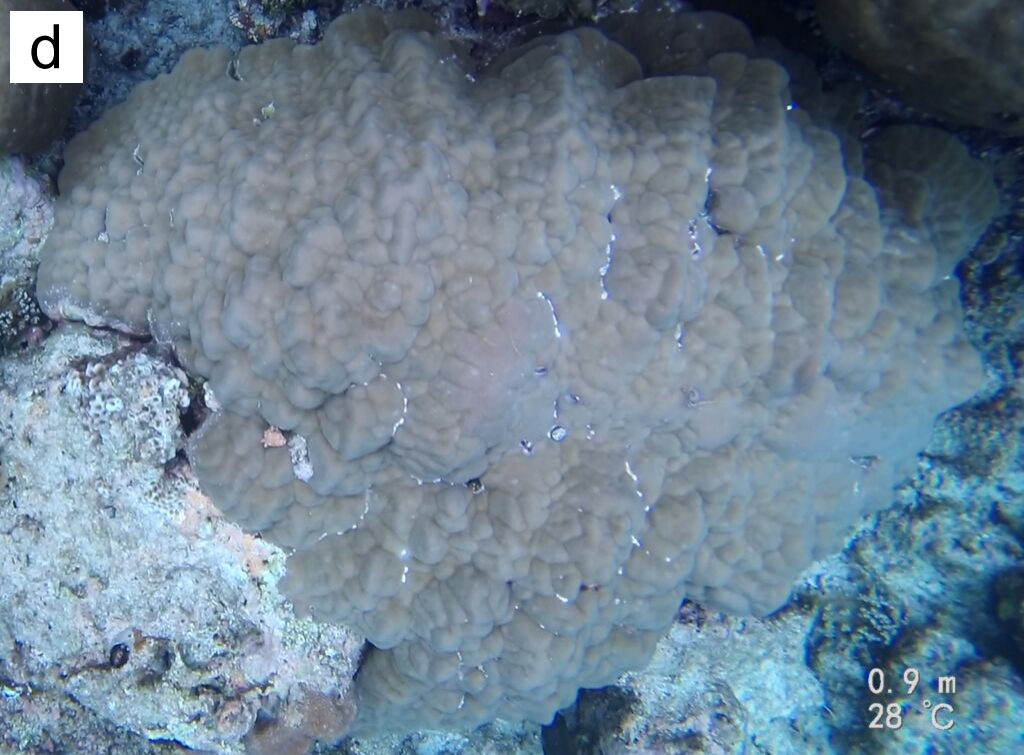
Vaavu, 2 m.

**Figure 111a.**
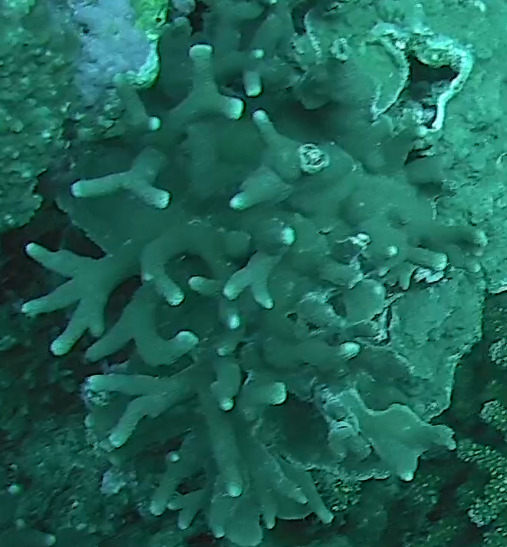
Addu, 30 m;

**Figure 111b.**
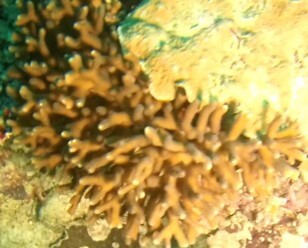
Addu, 30 m.

**Figure 112a.**
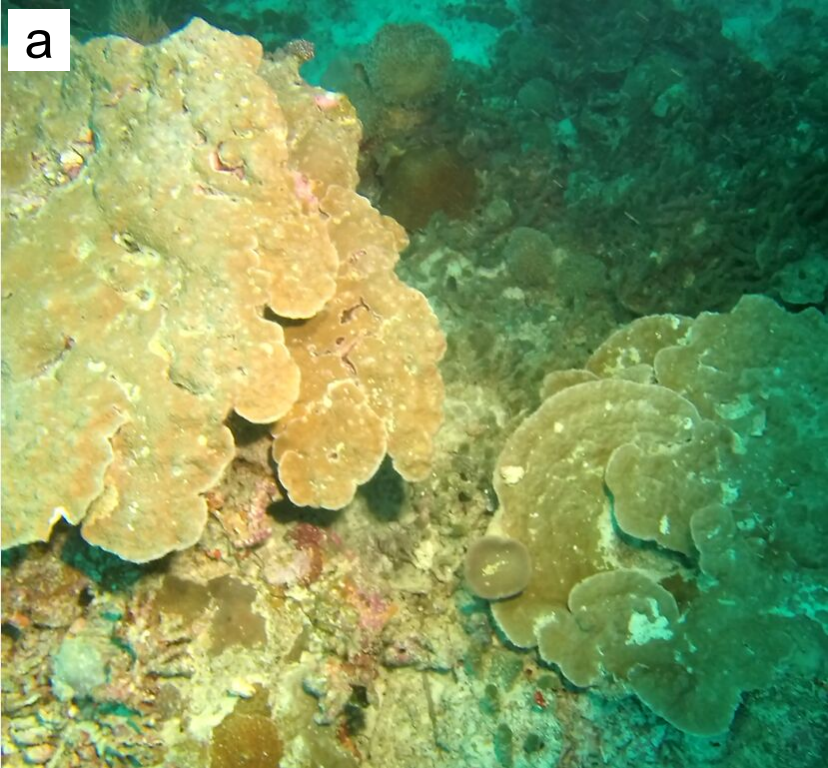
Addu, 30 m;

**Figure 112b.**
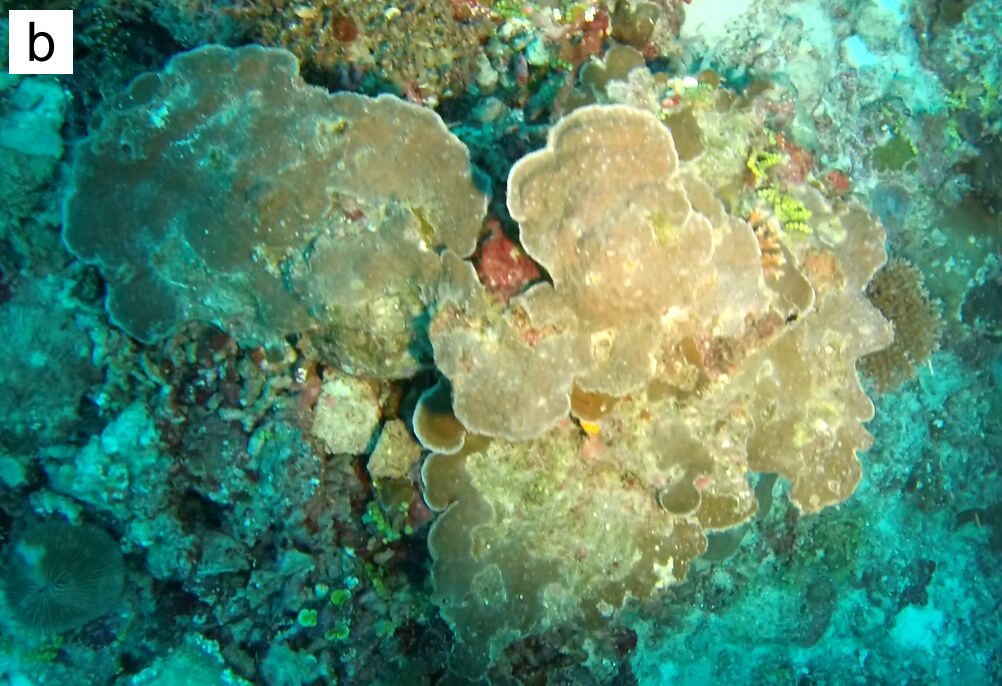
Vaavu, 30 m.

**Figure 113a.**
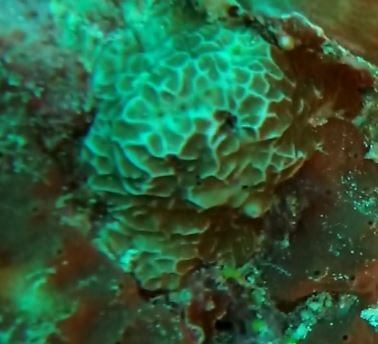
Huvadhu, 30 m;

**Figure 113b.**
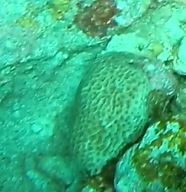
Huvadhu, 10m.

**Figure 114a.**
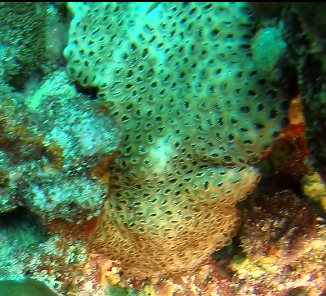
North Male’, 10 m;

**Figure 114b.**
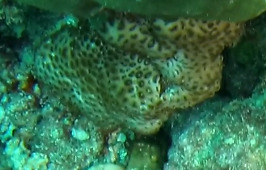
North Male’, 10 m.

**Figure 115.**
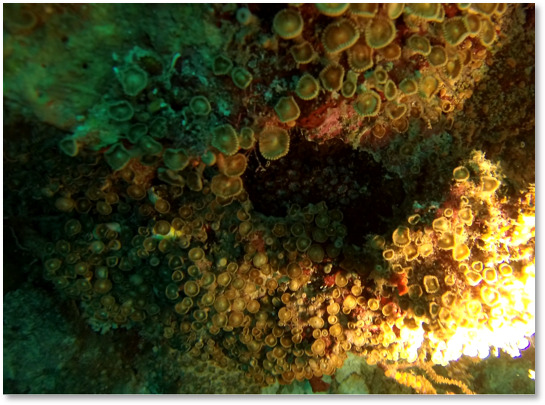
*Palythoa* sp. indet. 2, Addu, 30 m.

**Figure 116.**
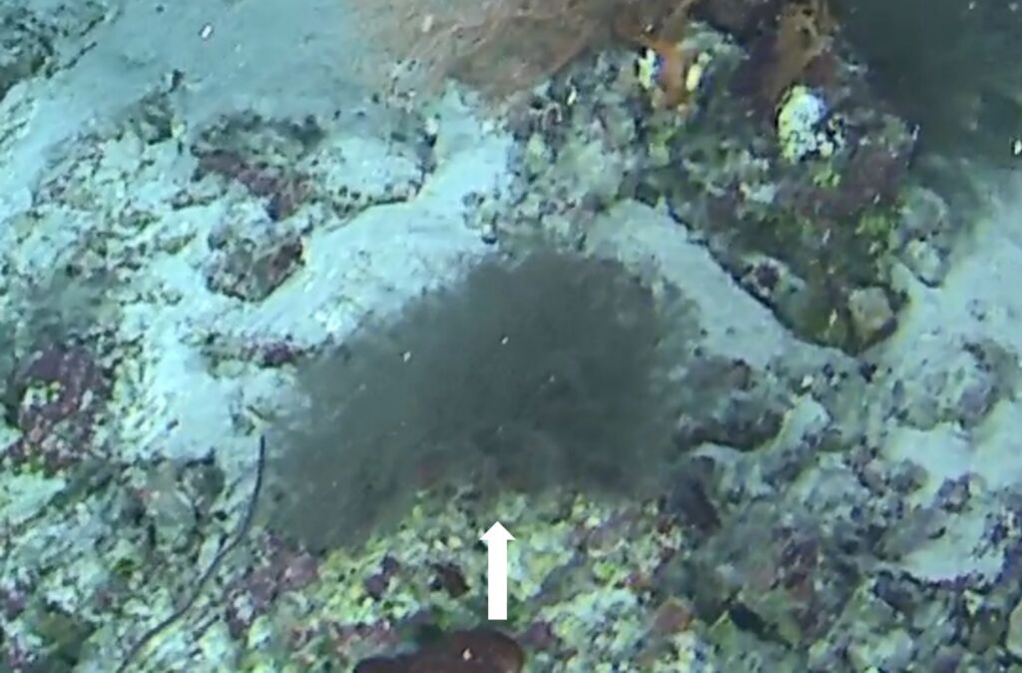
*Arachnopathes* sp. indet., Vaavu, 60 m.

**Figure 117a.**
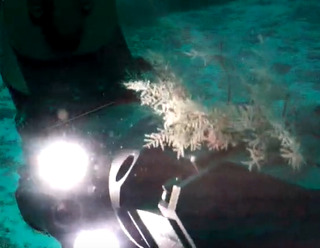
North Male’, 60 m, *in-situ* photo of collected specimen MAL1_747;

**Figure 117b.**
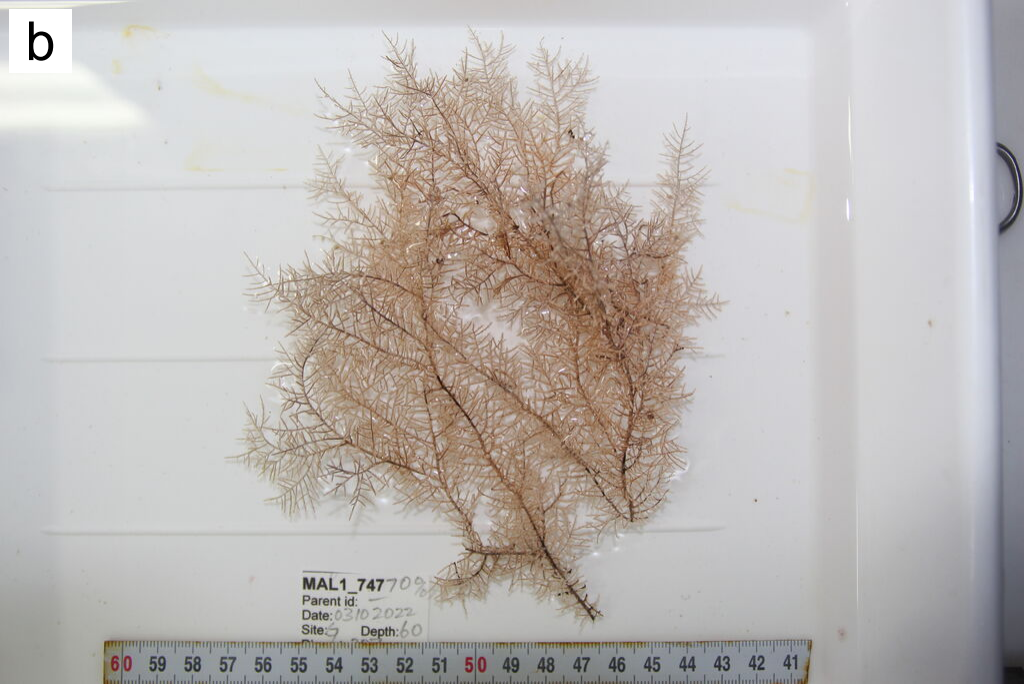
North Male’, 60 m, collected specimen MAL1_747.

**Figure 118.**
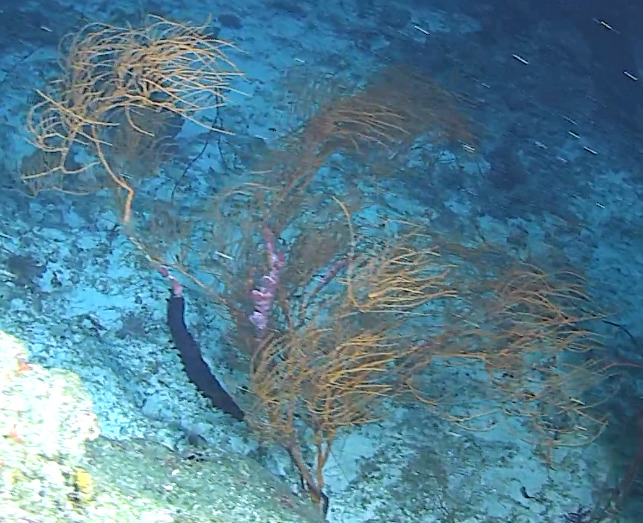
*Antipathes* sp. indet. 2 Huvadhu, 60 m.

**Figure 119a.**
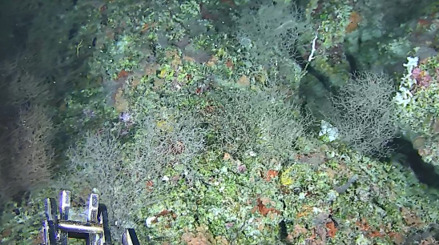
Addu, 60 m;

**Figure 119b.**
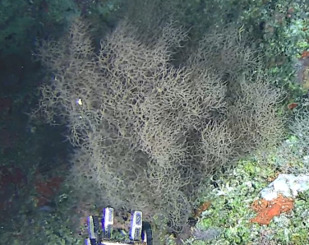
Addu, 60 m.

**Figure 120a.**
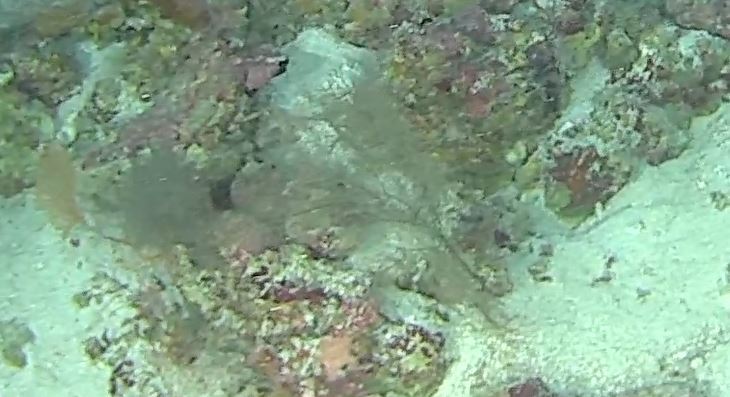
Vaavu, 60 m;

**Figure 120b.**
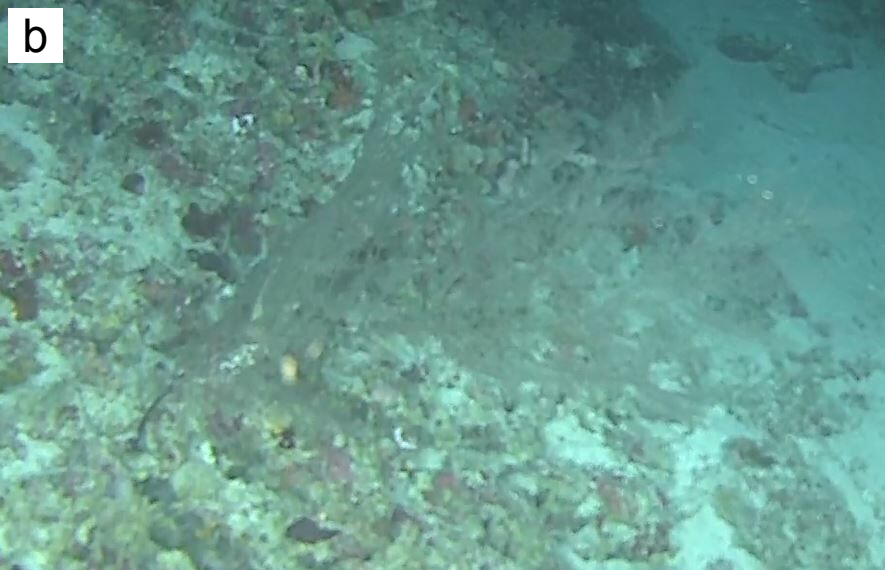
Vaavu, 60 m.

**Figure 121a.**
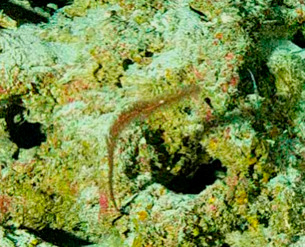
Vaavu, 121 m, in situ photo of collected specimen MAL1_630;

**Figure 121b.**
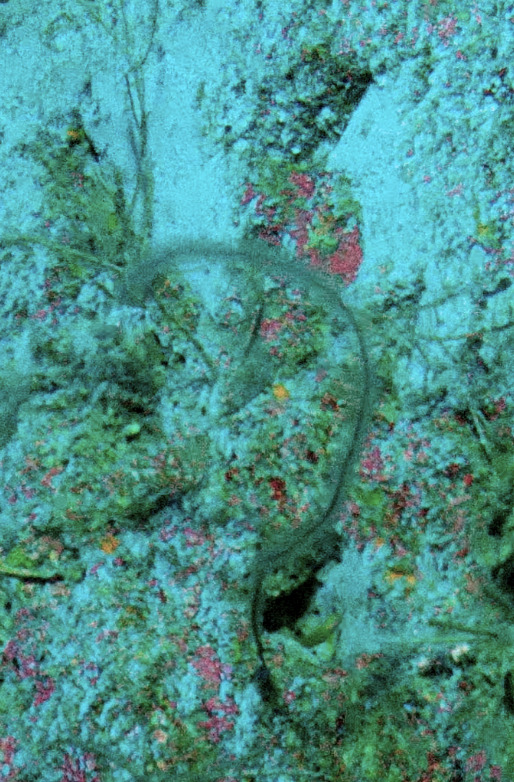
Laamu, 120 m;

**Figure 121c.**
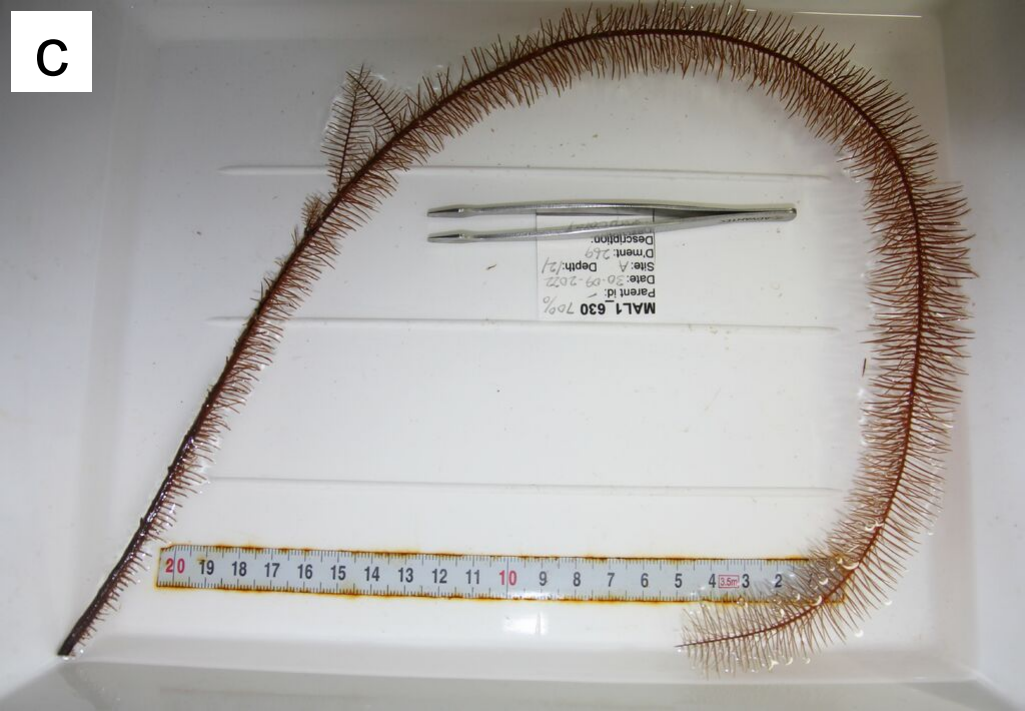
Vaavu, 121 m, Collected specimen MAL1_630.

**Figure 122a.**
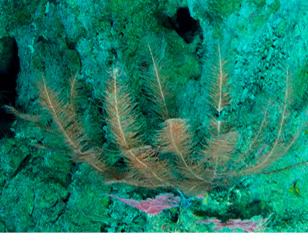
Vaavu, 60 m;

**Figure 122b.**
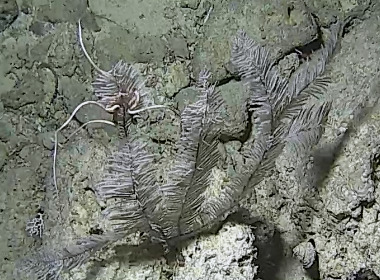
Addu, 250 m;

**Figure 122c.**
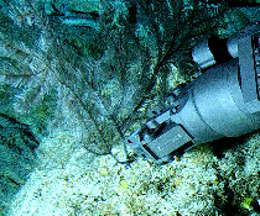
Fuvahmulah, 250 m, *in situ* of collected specimen MAL1_411;

**Figure 122d.**
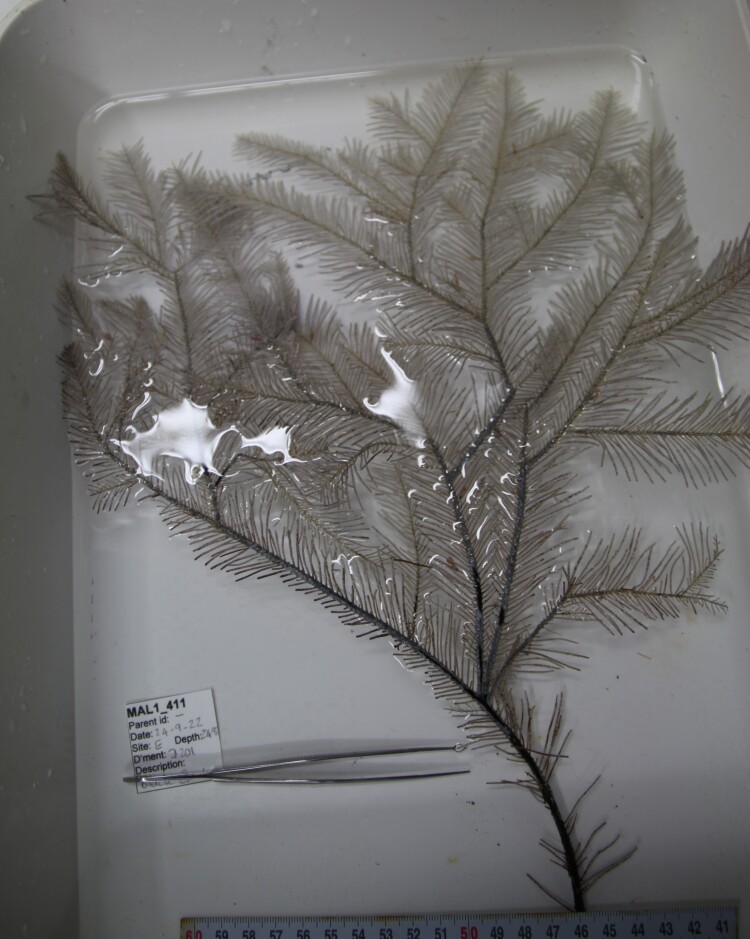
Fuvahmulah, 250 m, Collected specimen MAL1_411 (*Tetrapathes* sp. indet.);

**Figure 122e.**
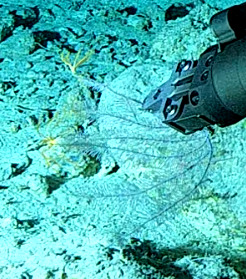
Addu, 262 m, *in situ* of collected specimen MAL1_284;

**Figure 122f.**
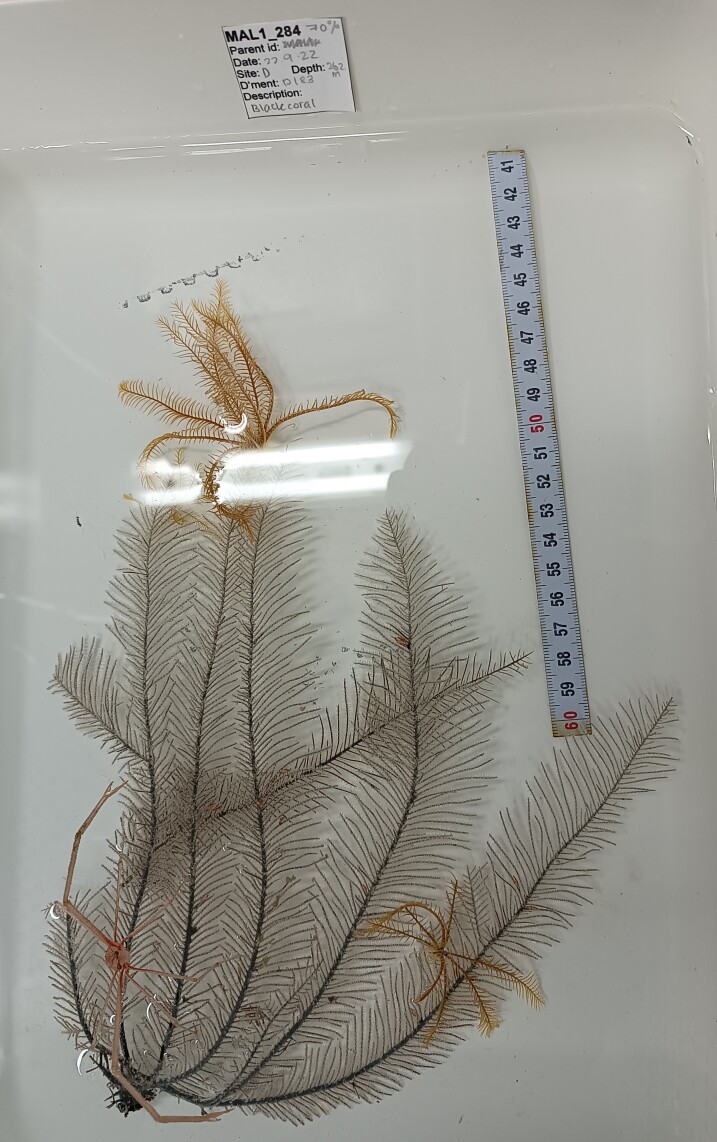
Addu, 262 m, Collected specimen MAL1_284 (*Tetrapathesalata* sp. inc.).

**Figure 123a.**
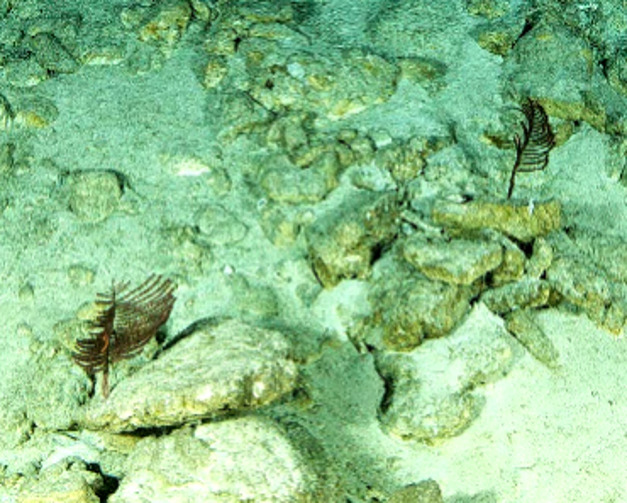
Fuvahmulah, 490 m;

**Figure 123b.**
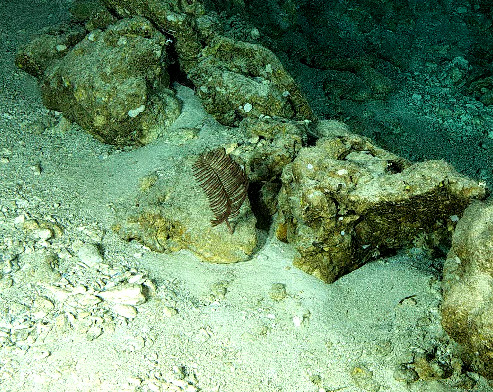
Fuvahmulah, 490 m, *in situ* of collected specimen MAL1_379;

**Figure 123c.**
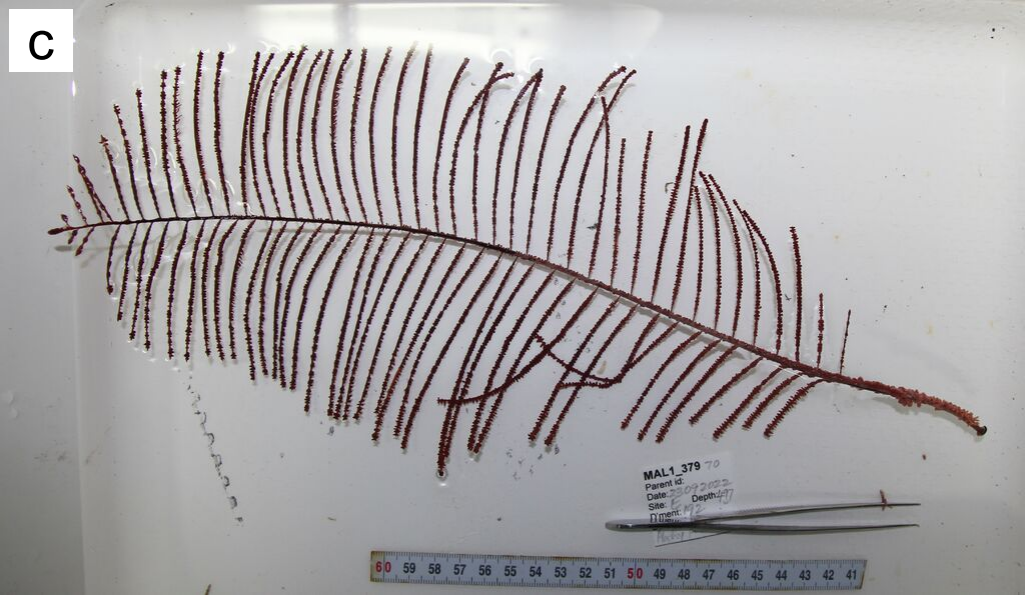
Fuvahmulah, 490 m, collected specimen MAL1_379 (*Bathypathespseudoalternata* sp. inc.).

**Figure 124a.**
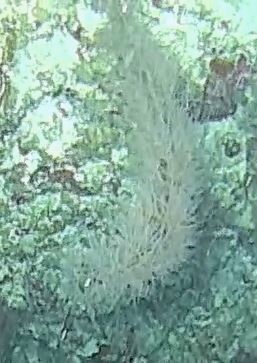
Vaavu, 120 m;

**Figure 124b.**
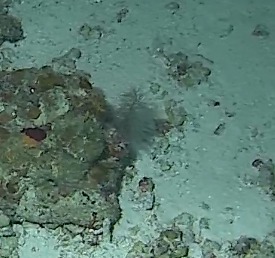
North Male’, 60 m.

**Figure 125.**
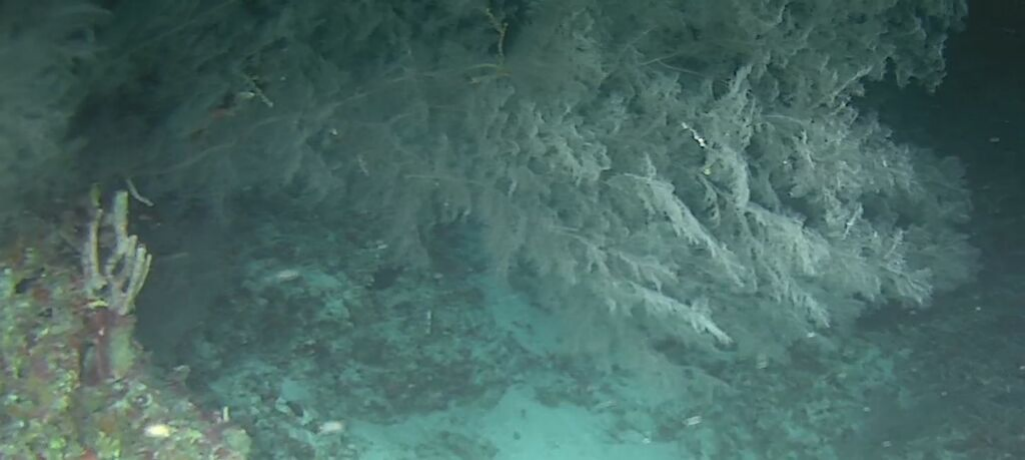
*Myriopathes* sp. indet. 1, Vaavu, 60 m.

**Figure 126a.**
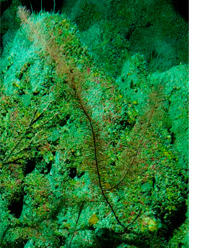
Vaavu, 123 m;

**Figure 126b.**
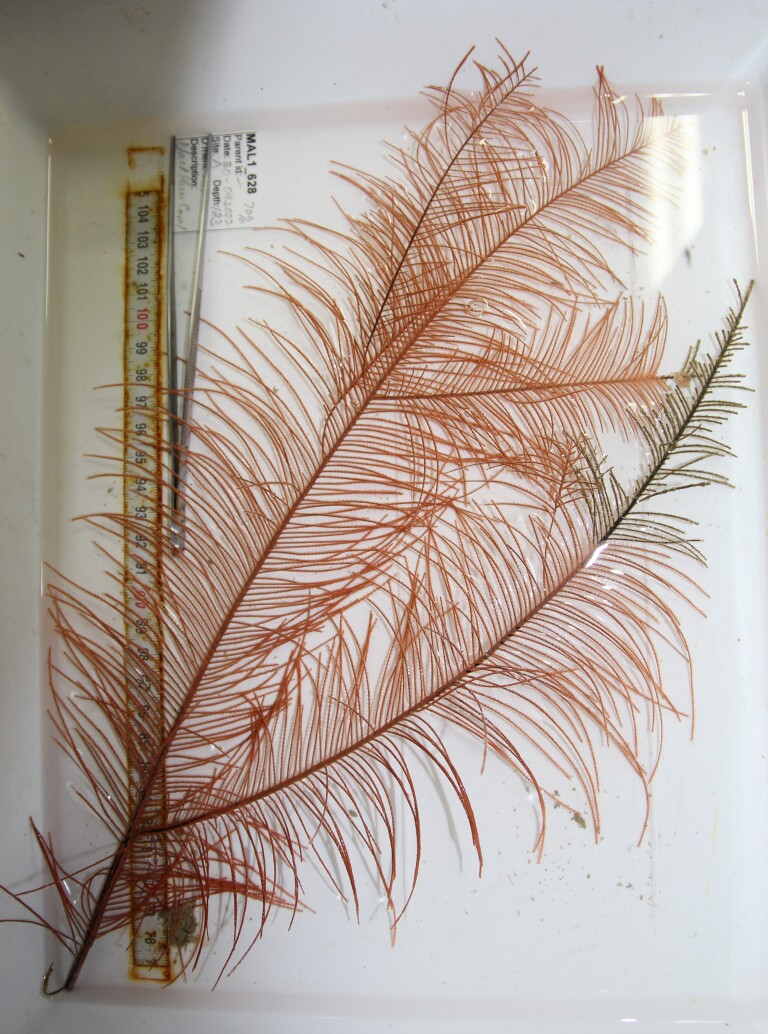
Vaavu, 123 m, collected specimen MAL1_628 (*Pteridopathes* sp. indet.);

**Figure 126c.**
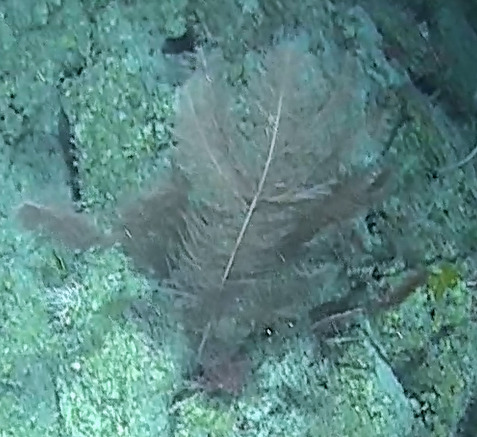
Vaavu, 120 m;

**Figure 126d.**
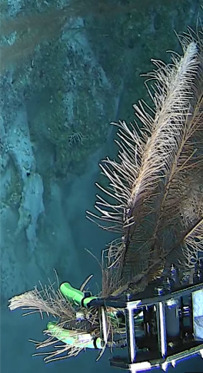
Huvadhu, 120 m, *in situ* photo of collected specimen MAL1_075;

**Figure 126e.**
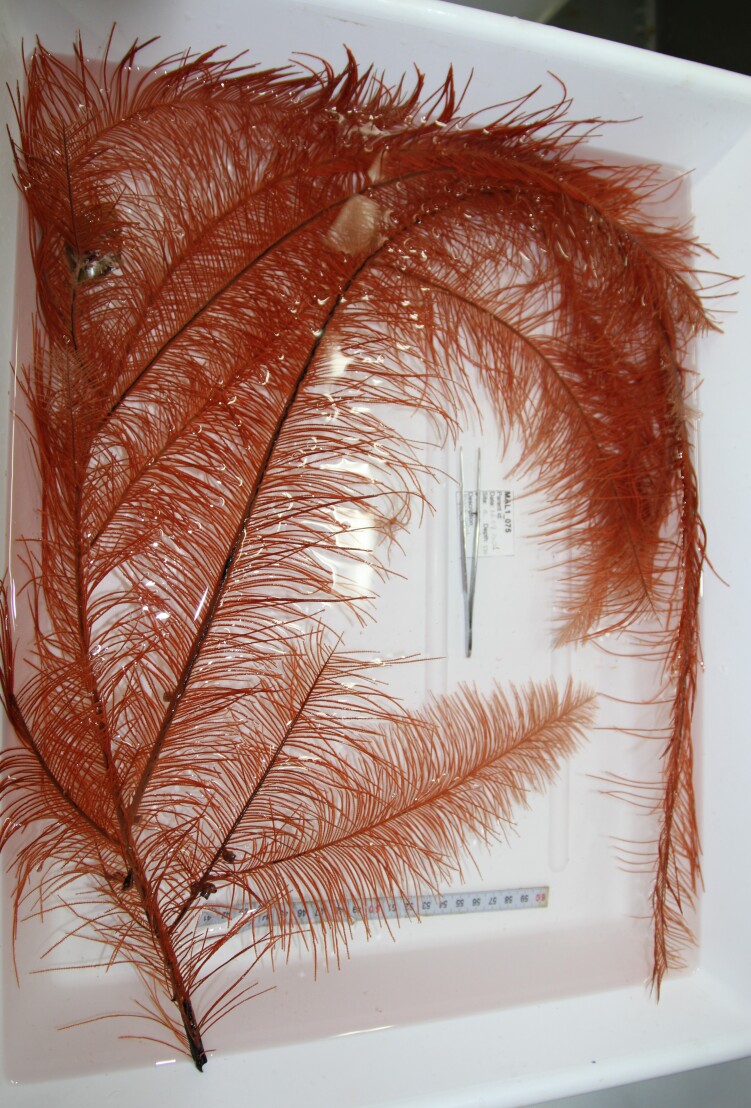
Huvadhu, 120 m, collected specimen MAL1_075.

**Figure 127a.**
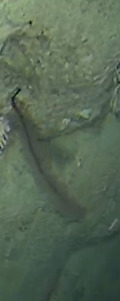
Huvadhu, 490 m;

**Figure 127b.**
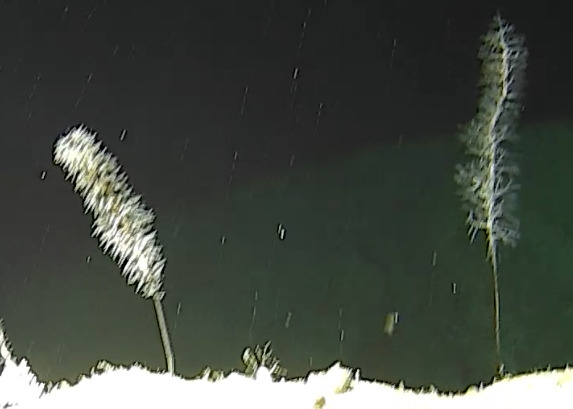
Huvadhu, 120 m.

**Figure 128a.**
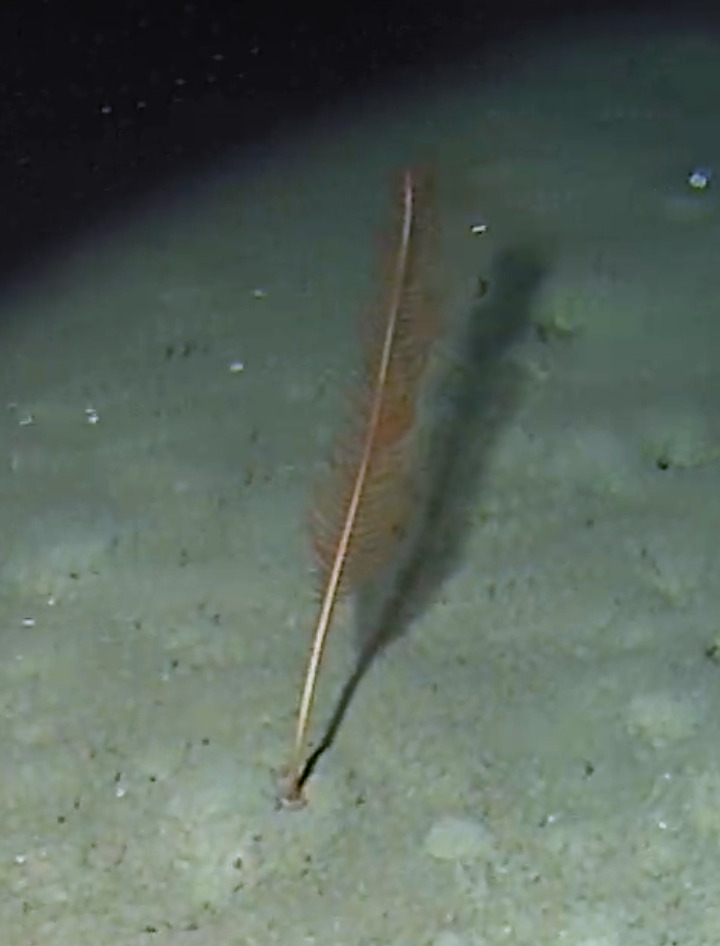
Vaavu, 490 m;

**Figure 128b.**
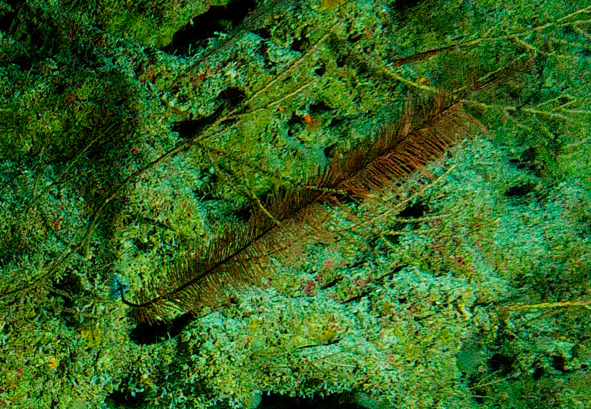
Vaavu, 120 m.

**Figure 129a.**
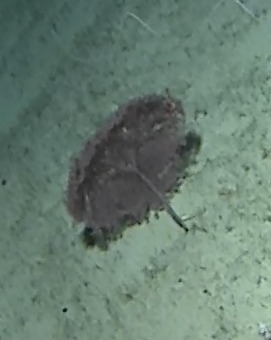
Fuvahmulah, 490 m;

**Figure 129b.**
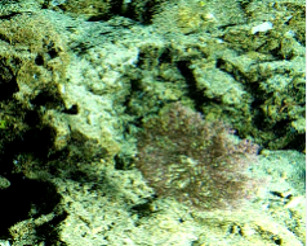
Fuvahmulah, 490 m, *in situ* of collected specimen MAL1_373;

**Figure 129c.**
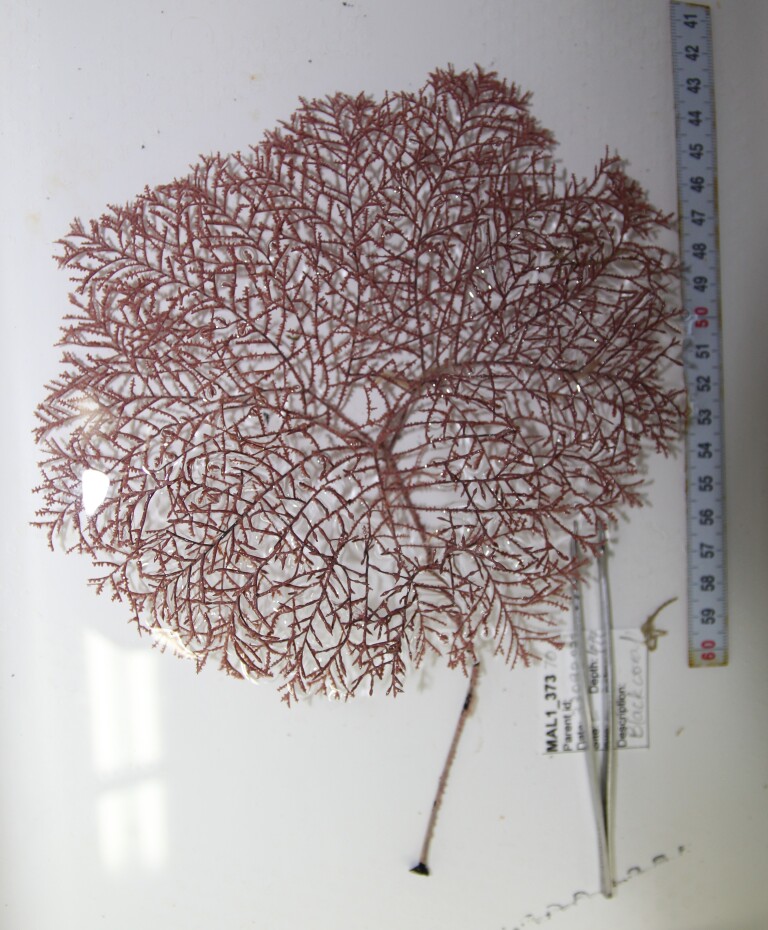
Fuvahmulah, 490 m, collected specimen MAL1_373.

**Figure 130a.**
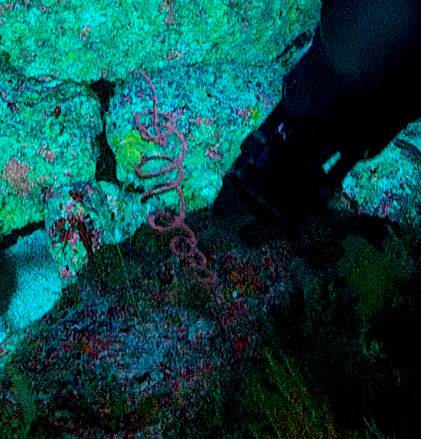
Huvadhu, 120 m, *in situ* photo of collected specimen MAL1_503 (*Stichopathes* sp. indet.);

**Figure 130b.**
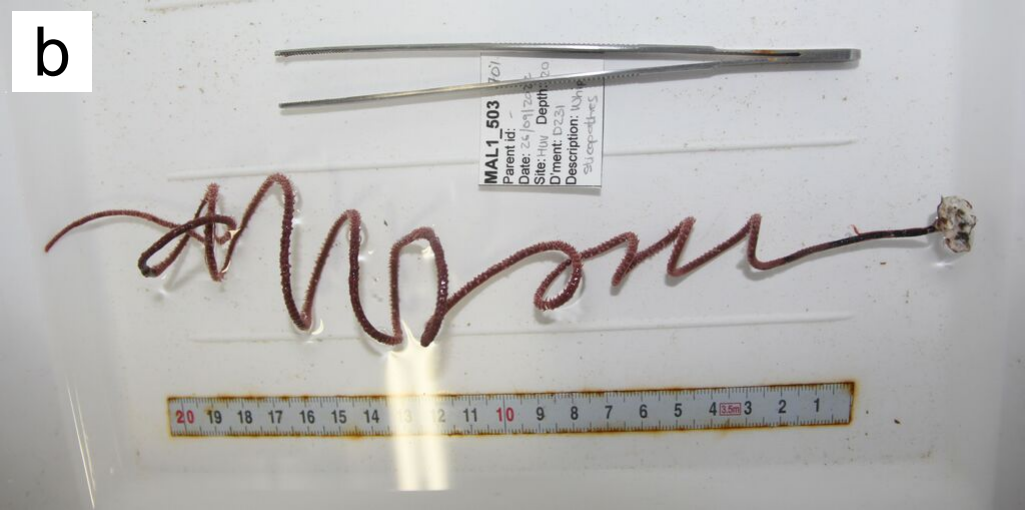
Huvadhu, 120 m collected specimen MAL1_503 (*Stichopathes* sp. indet.);

**Figure 130c.**
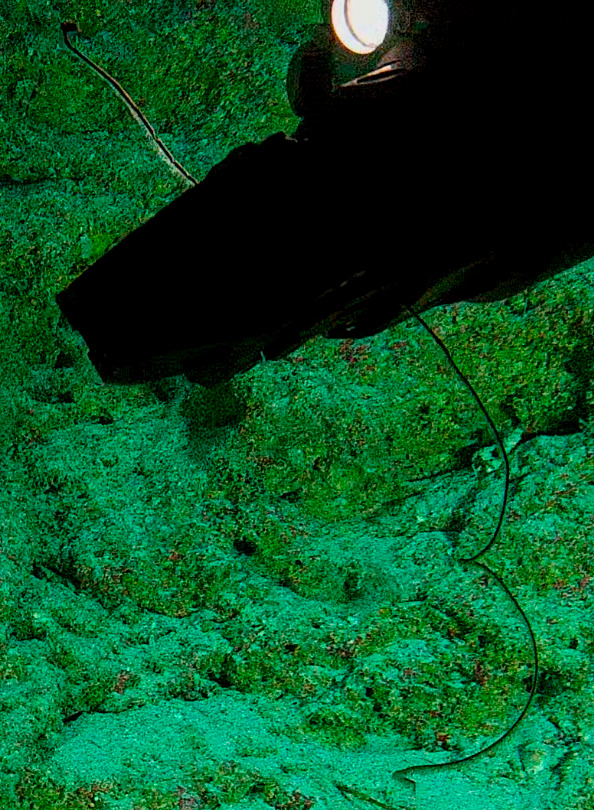
Vaavu, 119 m, *in situ* photo of collected specimen MAL1_643 (*Stichopathes* sp. indet.);

**Figure 130d.**
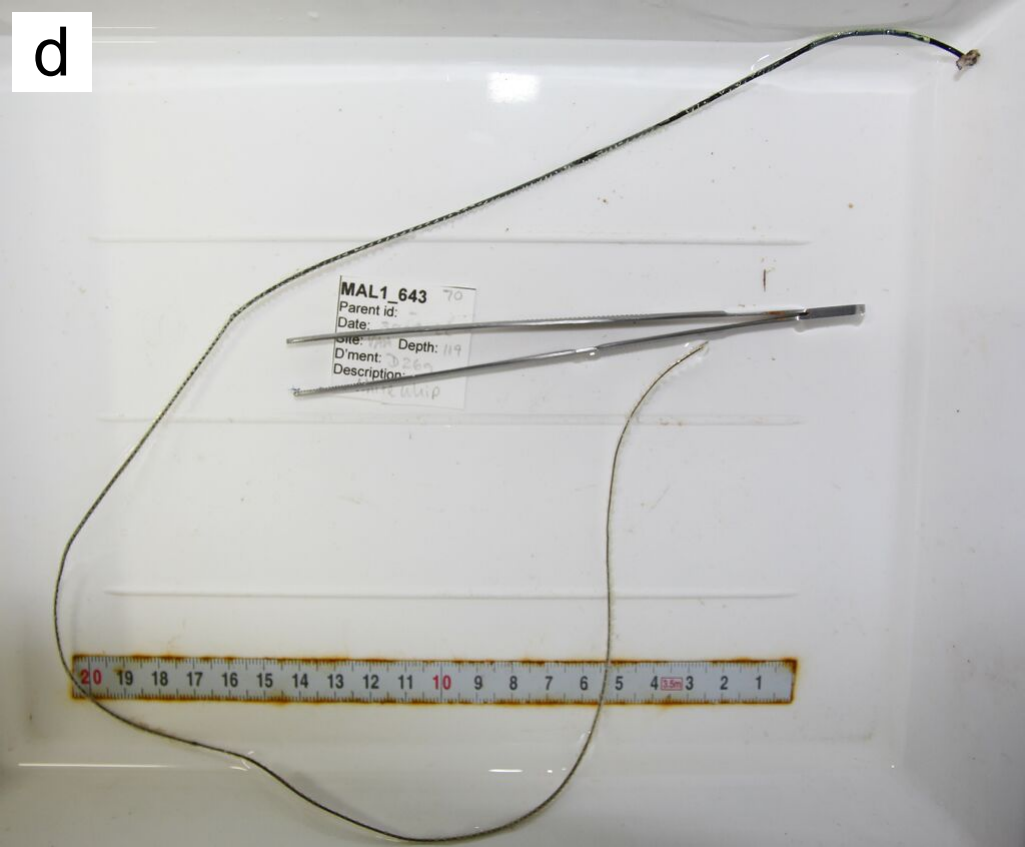
Vaavu, 119 m, collected specimen MAL1_643 (*Stichopathes* sp. indet.);

**Figure 130e.**
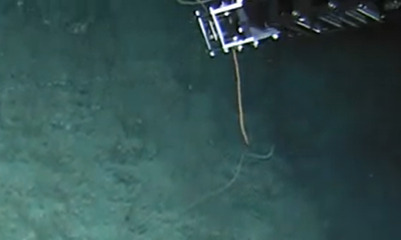
North Male’, 120 m, *in situ* of MAL1_689 (*Cirrhipathes* sp. indet.);

**Figure 130f.**
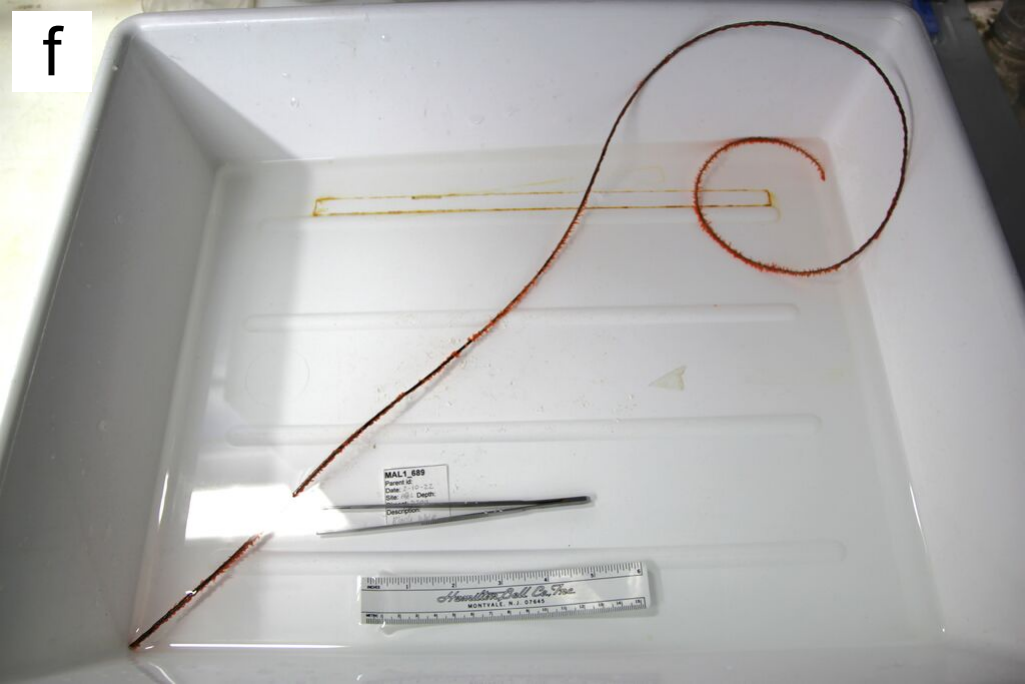
North Male’, 120 m, MAL1_689 (*Cirrhipathes* sp. indet.).

**Figure 131a.**
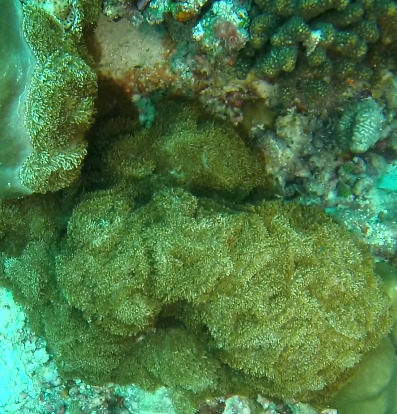
North Male’, 10 m;

**Figure 131b.**
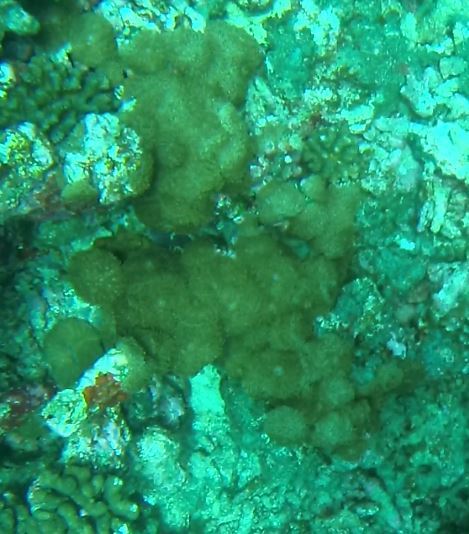
Huvadhu, 10 m.

**Figure 132a.**
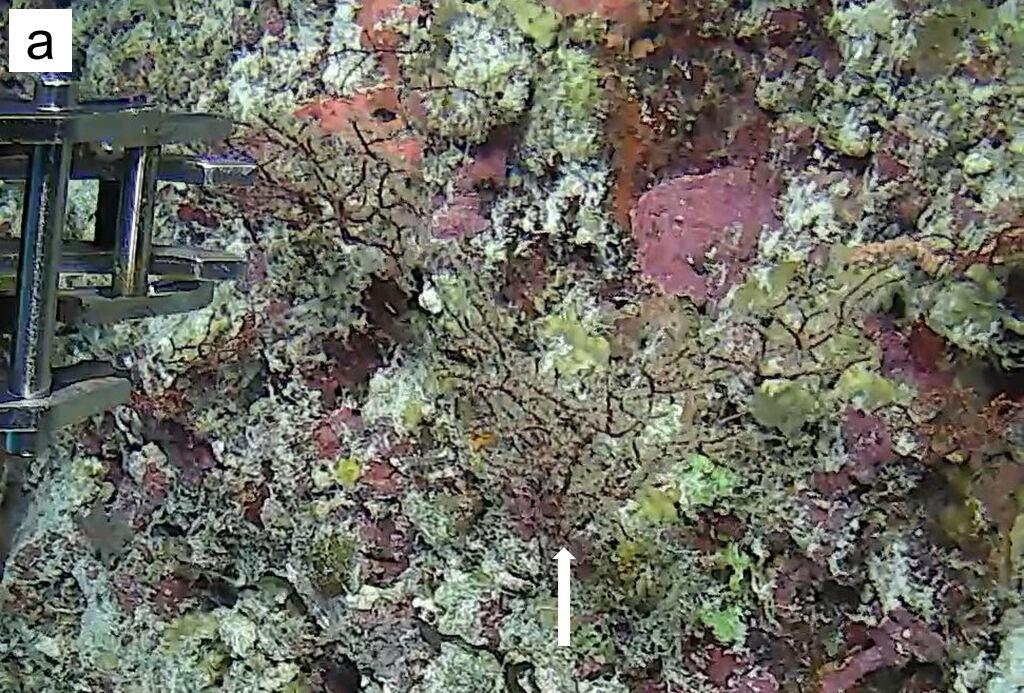
Addu, 60 m;

**Figure 132b.**
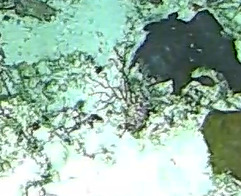
Addu, 60 m.

**Figure 133a.**
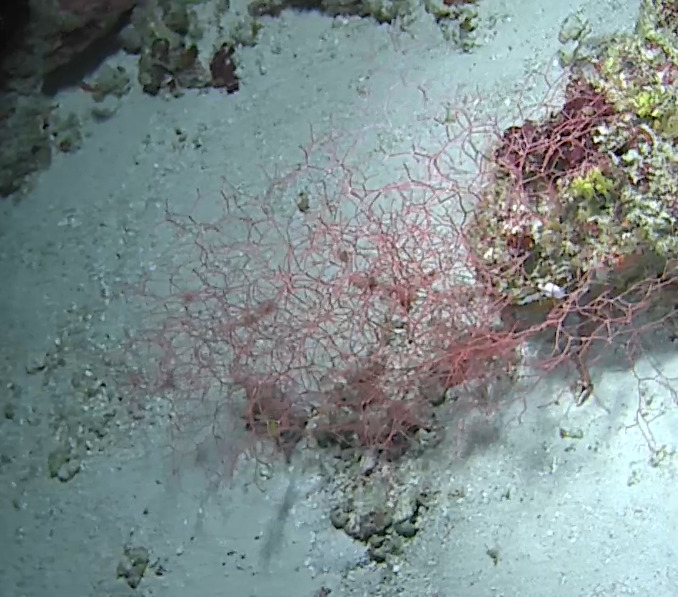
North Male’, 58 m;

**Figure 133b.**
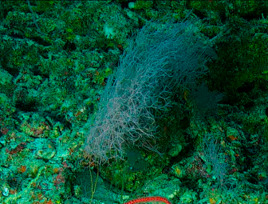
Vaavu, 60 m.

**Figure 134a.**
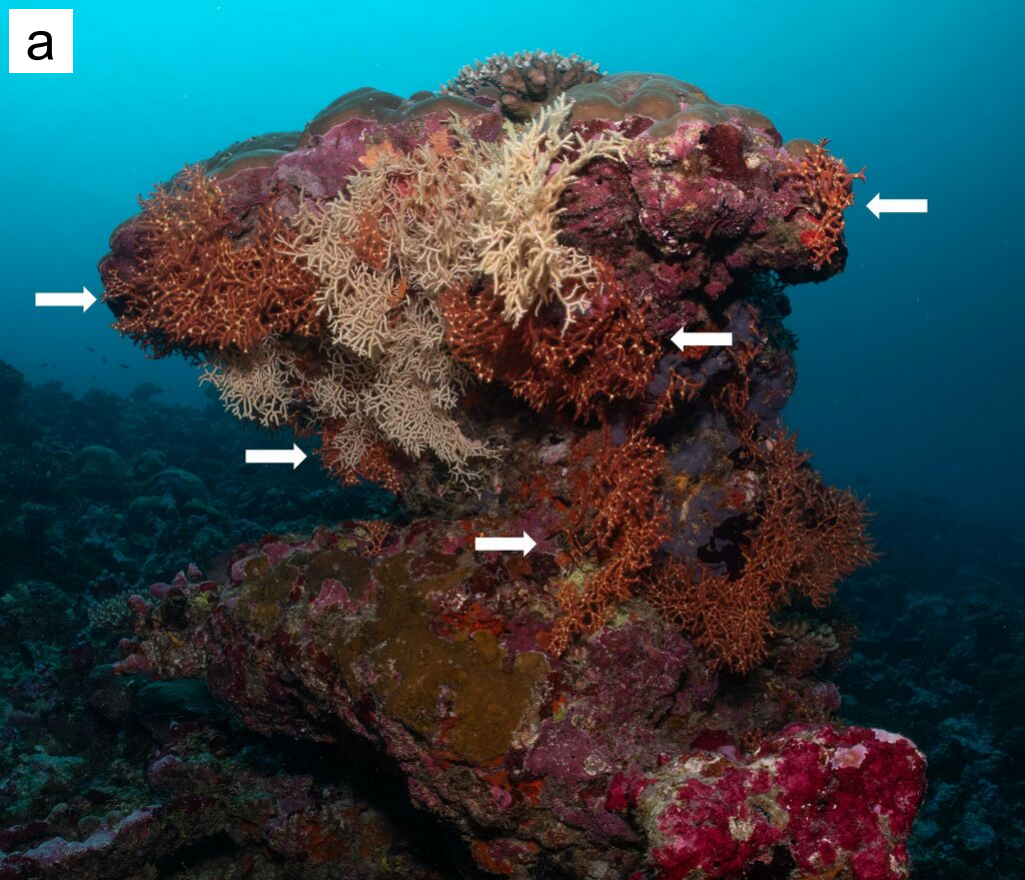
Laamu, 10-30 m;

**Figure 134b.**
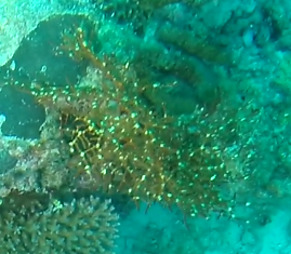
Huvadhu, 30 m.

**Figure 135.**
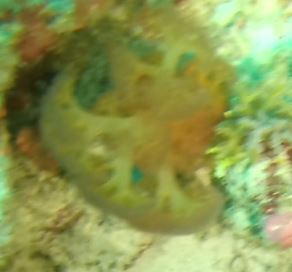
*Dendronephthya* sp. indet. 1, Huvadhu, 30 m.

**Figure 136a.**
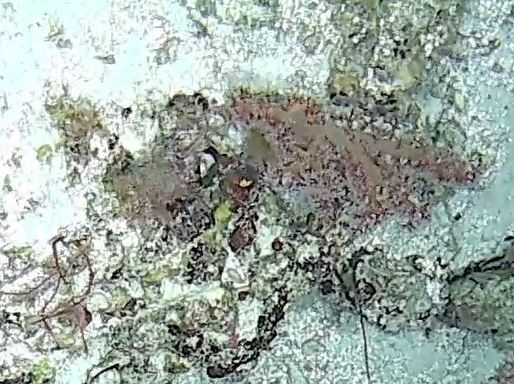
Laamu, 120 m;

**Figure 136b.**
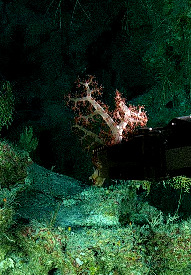
North Male’, 109 m, *in situ* photo of collected specimen MAL1_719;

**Figure 136c.**
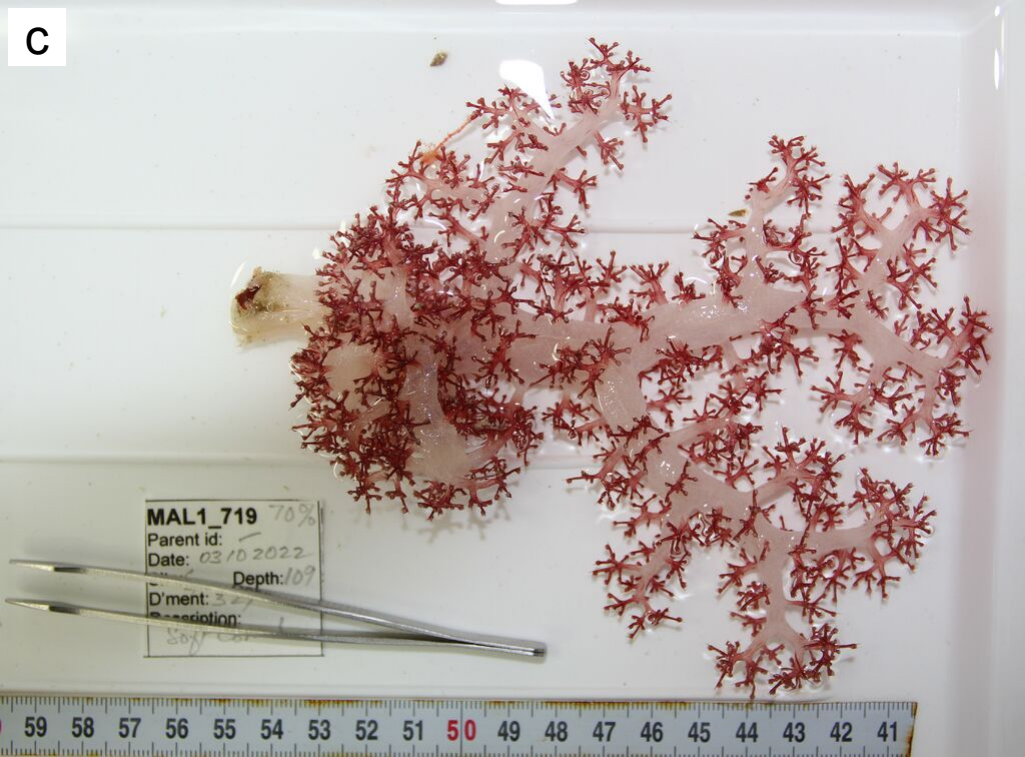
North Male’, 109 m, collected specimen MAL1_719.

**Figure 137a.**
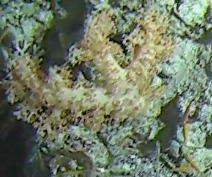
Huvadhu, 120 m;

**Figure 137b.**
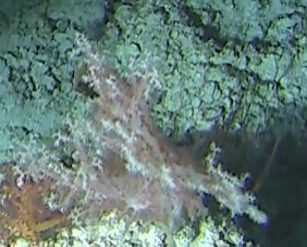
Huvadhu, 120 m.

**Figure 138.**
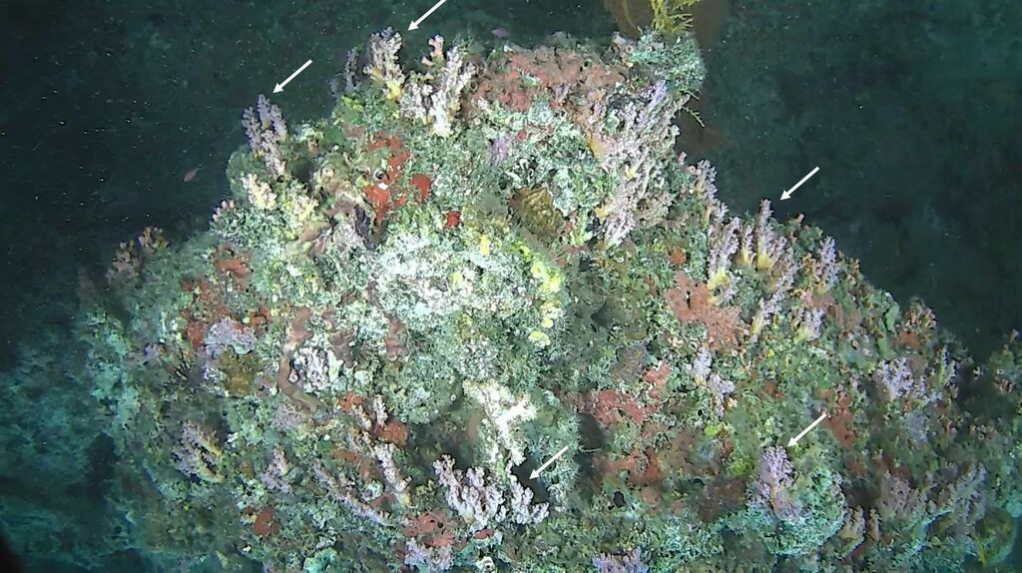
Scleronephythya sp. indet. 2, Huvadhu, 60 m.

**Figure 139a.**
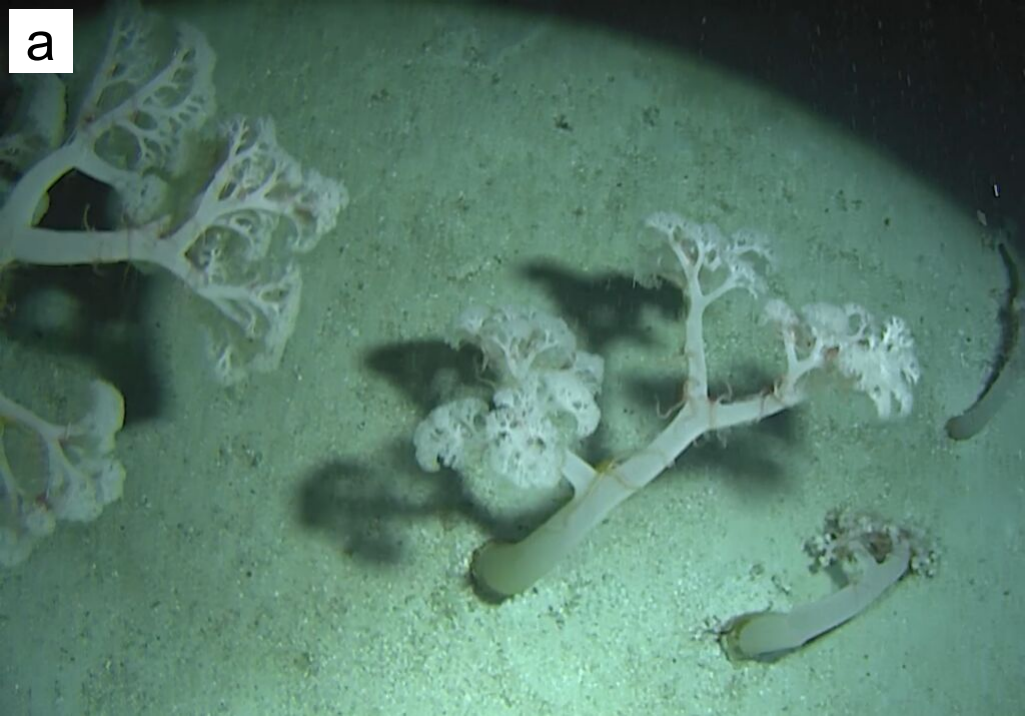
North Male’, 60-120 m;

**Figure 139b.**
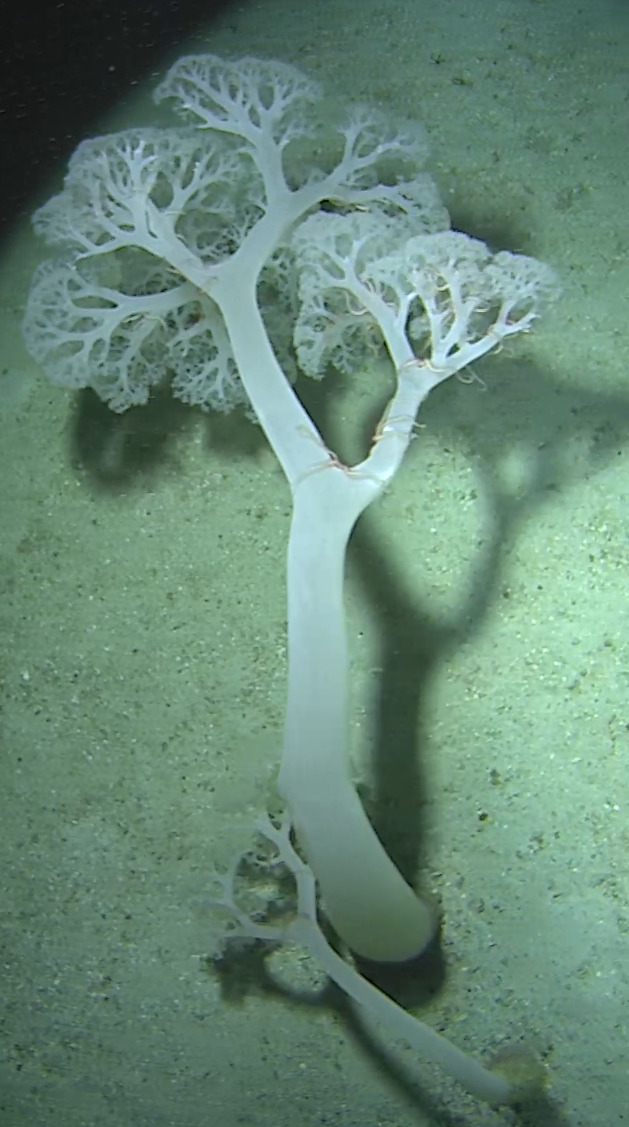
North Male’, 60-120 m.

**Figure 140a.**
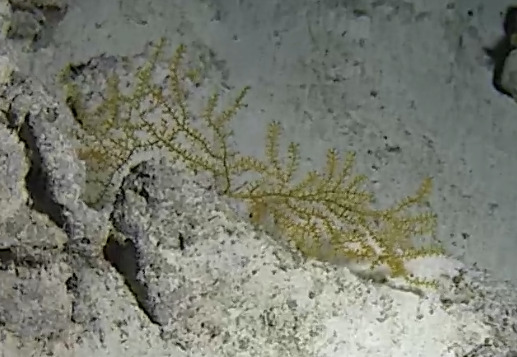
Fuvahmulah, 490 m;

**Figure 140b.**
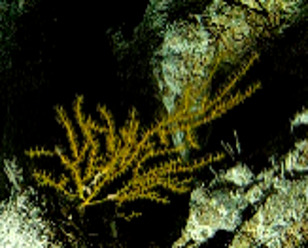
Fuvahmulah, 490 m.

**Figure 141a.**
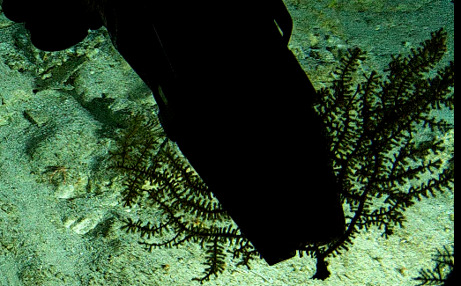
Fuvahmulah, 250 m, *in situ* photo of collected specimen MAL1_426;

**Figure 141b.**
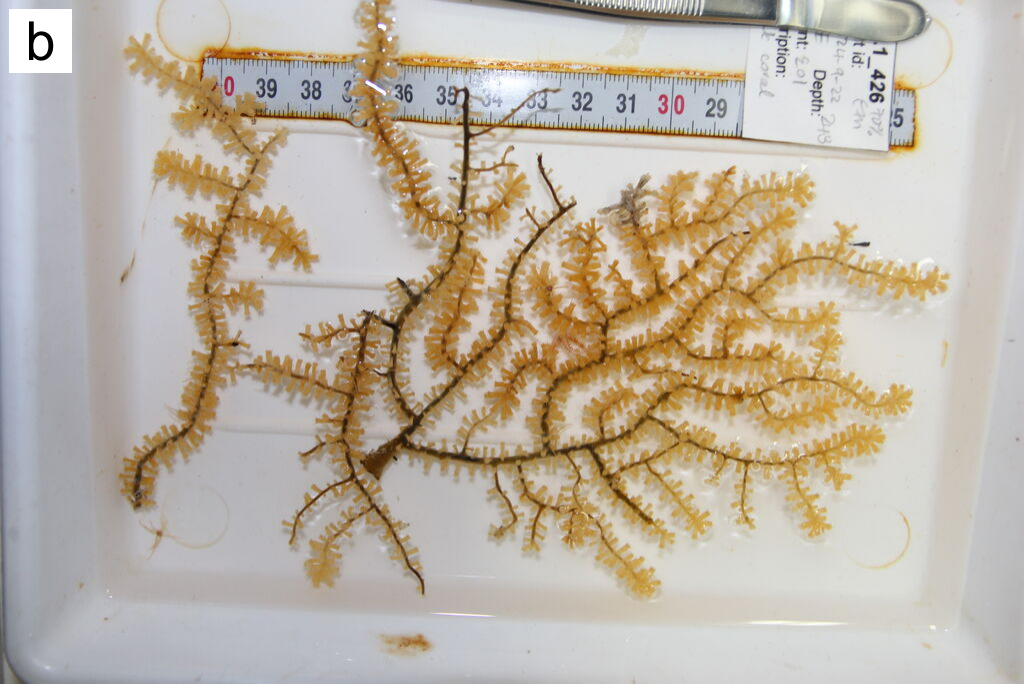
Fuvahmulah, 250 m, collected specimen MAL1_426.

**Figure 142a.**
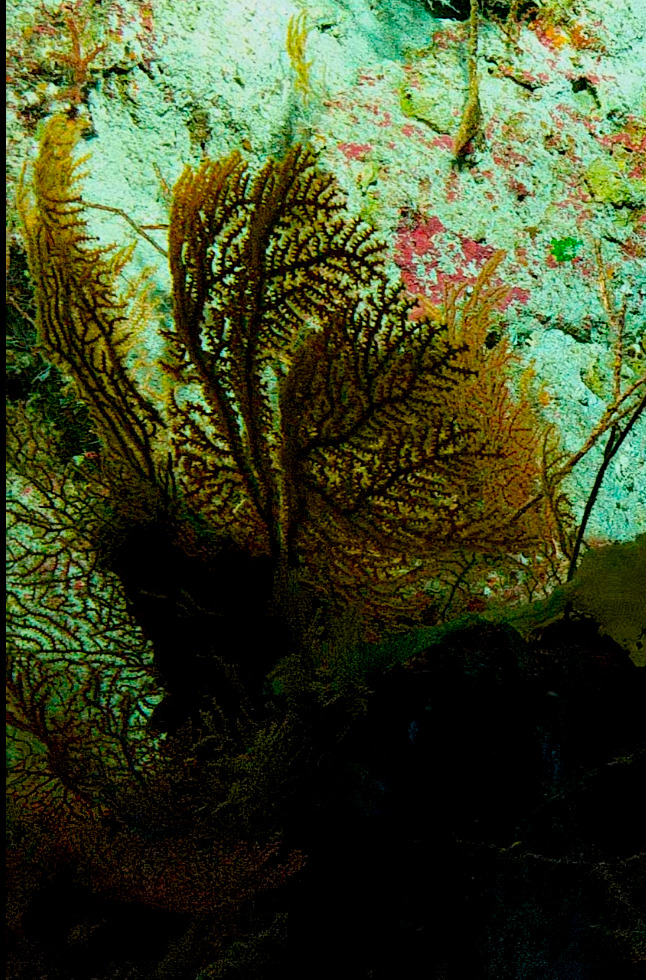
Fuvahmulah, 120 m;

**Figure 142b.**
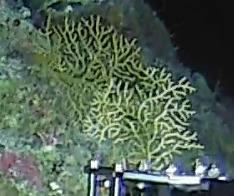
Addu, 250 m;

**Figure 142c.**
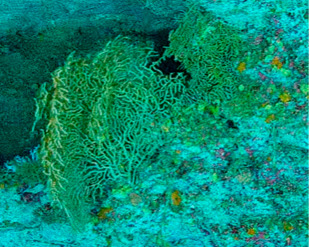
Fiuvahmulah, 120 m.

**Figure 143.**
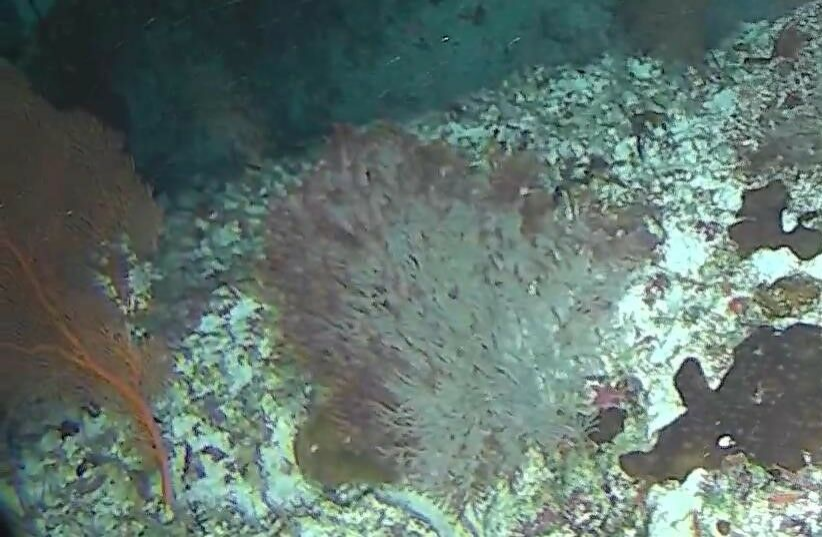
Plexauridae gen. indet. sp. 9, Addu, 60 m.

**Figure 144a.**
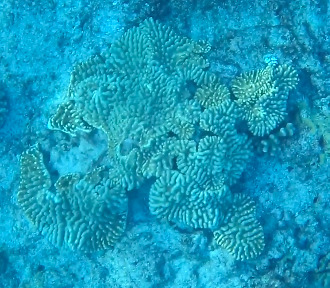
Fuvahmulah, 2 m;

**Figure 144b.**
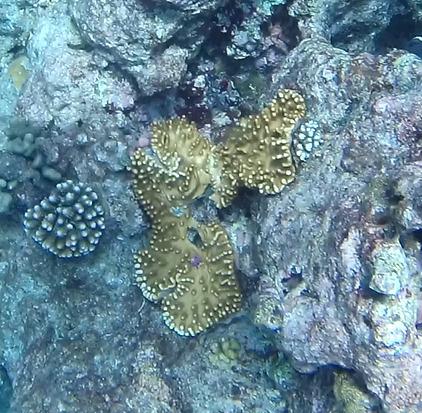
Vaavu, 2 m.

**Figure 145a.**
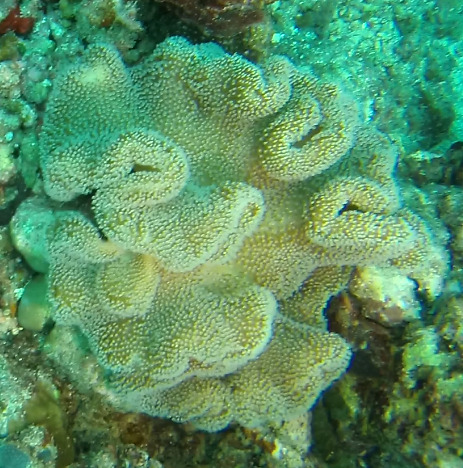
North Male’, 10 m;

**Figure 145b.**
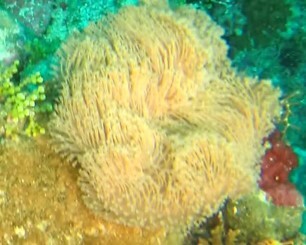
Laamu, 30 m.

**Figure 146a.**
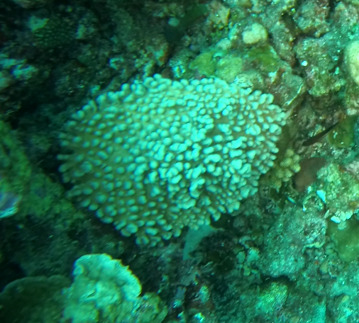
North Male’, 10 m;

**Figure 146b.**
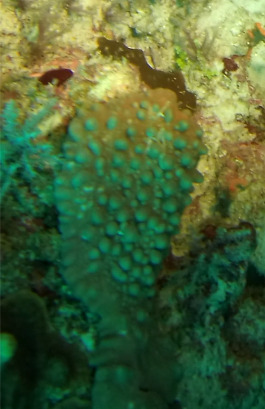
Huvadhu, 30 m.

**Figure 147a.**
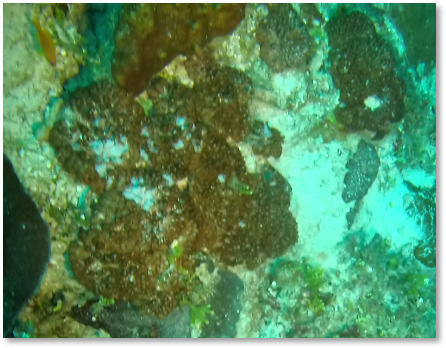
Huvadhu, 10 m;

**Figure 147b.**
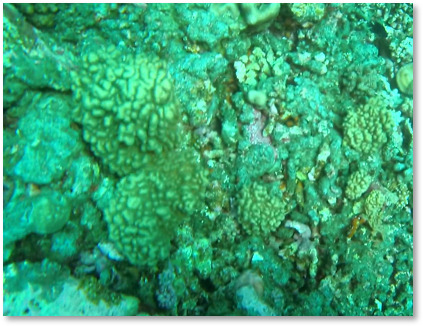


**Figure 147c.**
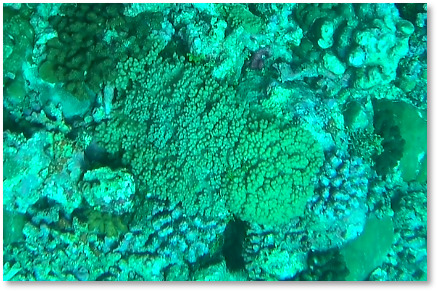
Laamu, 10 m.

**Figure 148.**
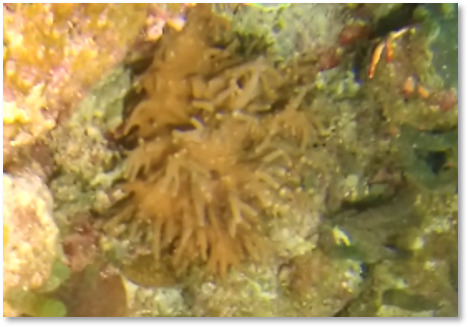
*Sinulariidae* gen. indet. sp. 3, Addu, 30 m.

**Figure 149a.**
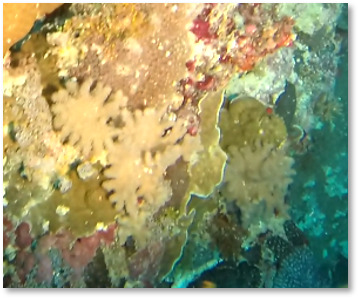
Vaavu, 30 m;

**Figure 149b.**
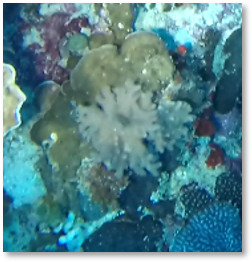
Vaavu, 30 m;

**Figure 149c.**
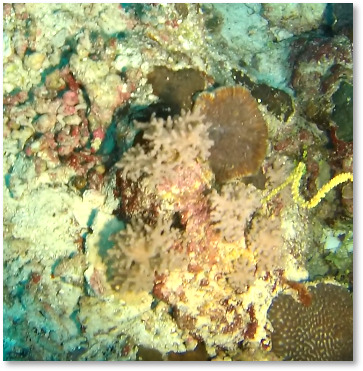
Vaavu, 30 m.

**Figure 150a.**
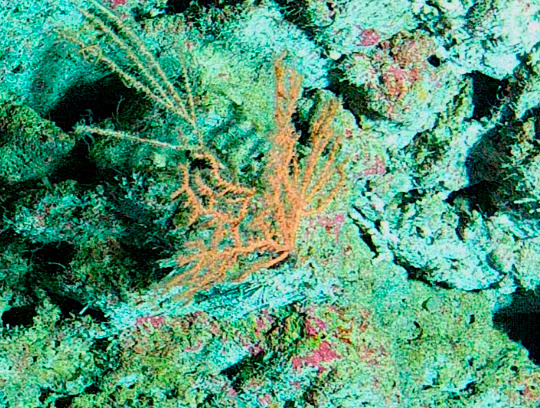
Vaavu, 121 m, *in situ* photo of collected specimen MAL1_632;

**Figure 150b.**
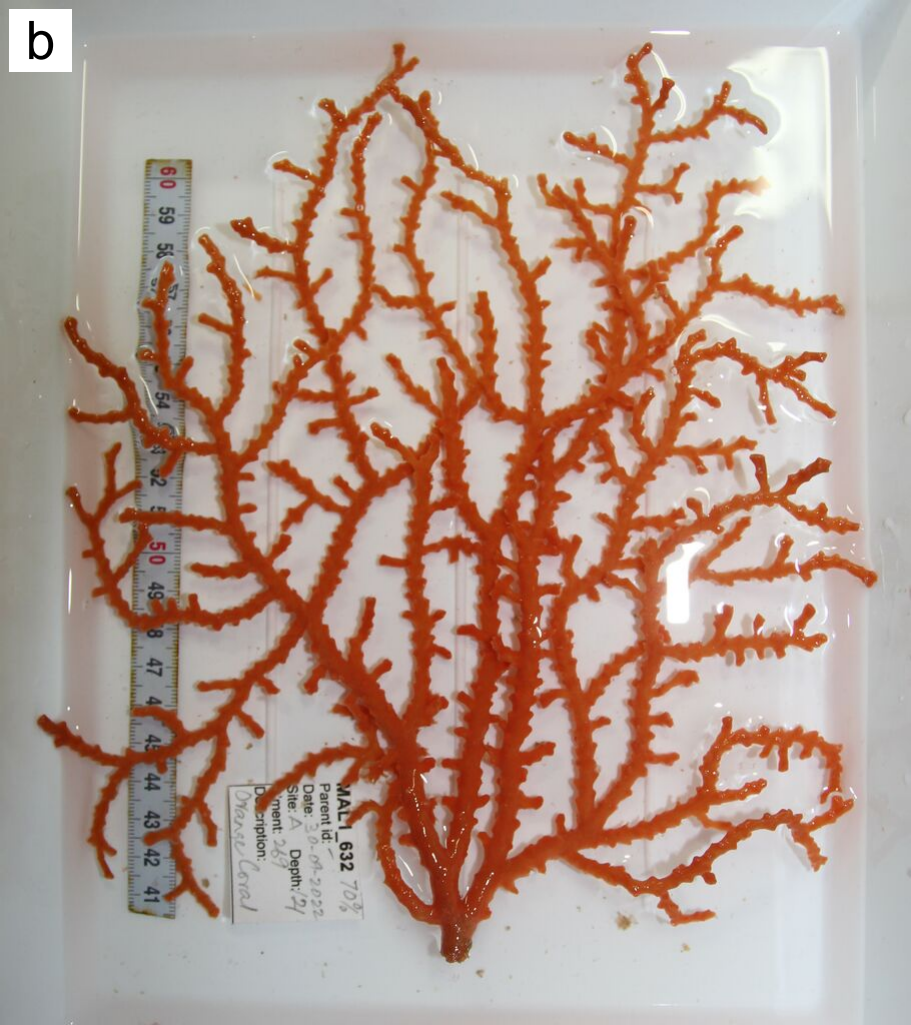
Vaavu, 121 m, collected specimen MAL1_632.

**Figure 151a.**
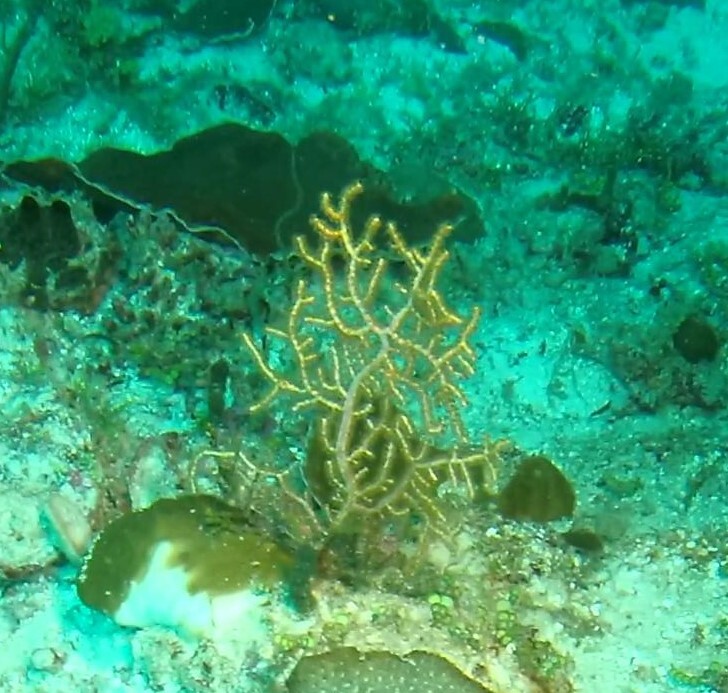
Laamu, 30 m;

**Figure 151b.**
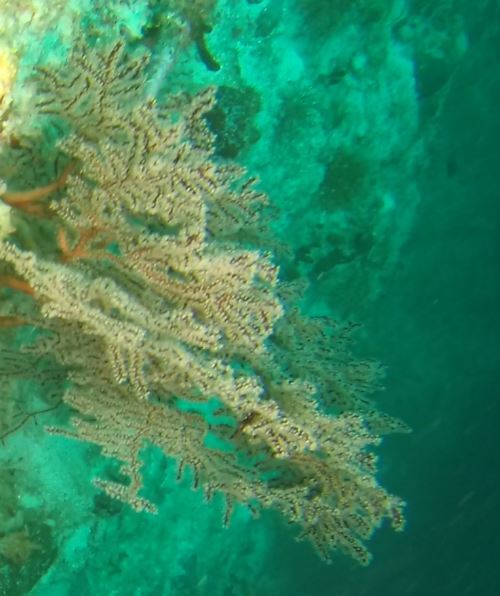
Huvadhu, 30 m;

**Figure 151c.**
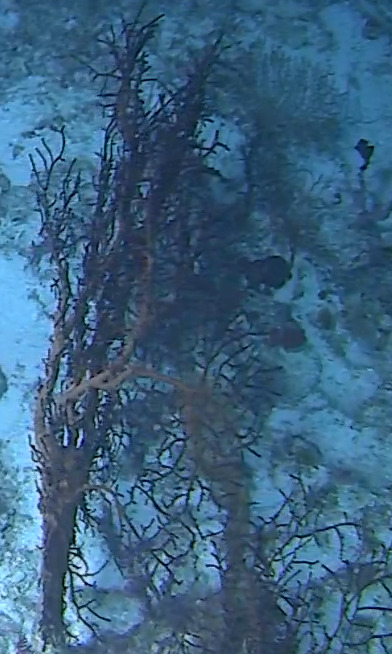
Huvadhu, 60 m.

**Figure 152a.**
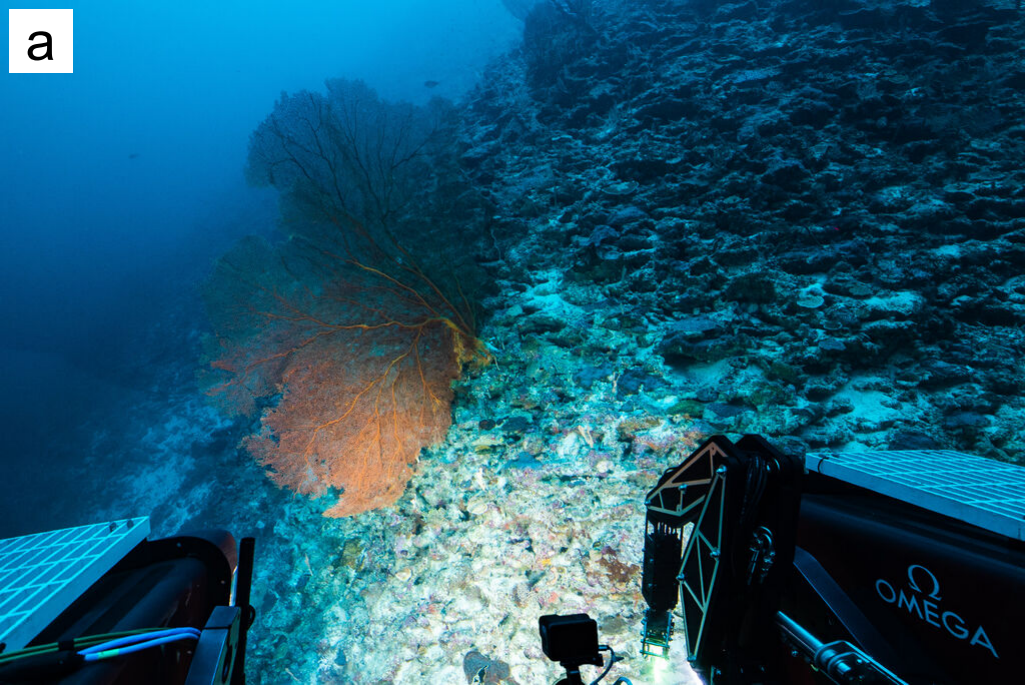
Huvadhu, ~ 60-90 m;

**Figure 152b.**
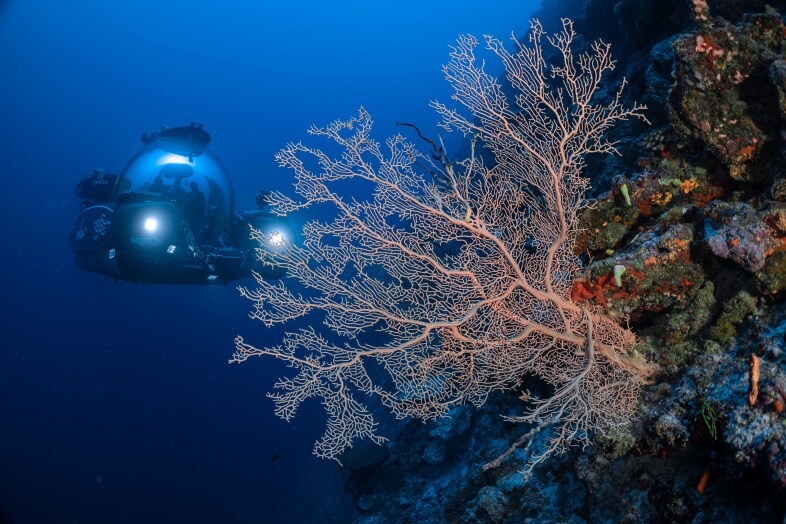
Fuvahmulah, ~ 30 m;

**Figure 152c.**
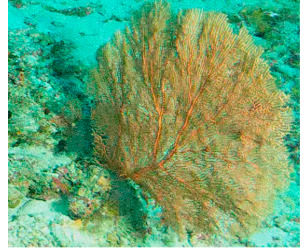
Vaavu, 60 m;

**Figure 152d.**
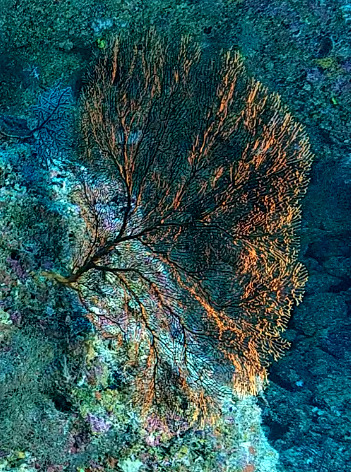
Fuvahmulah, ~ 90-120 m.

**Figure 153a.**
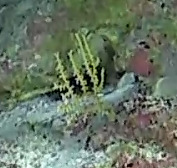
Huvadhu, 120 m, *in situ* photo of collected specimen MAL1_514;

**Figure 153b.**
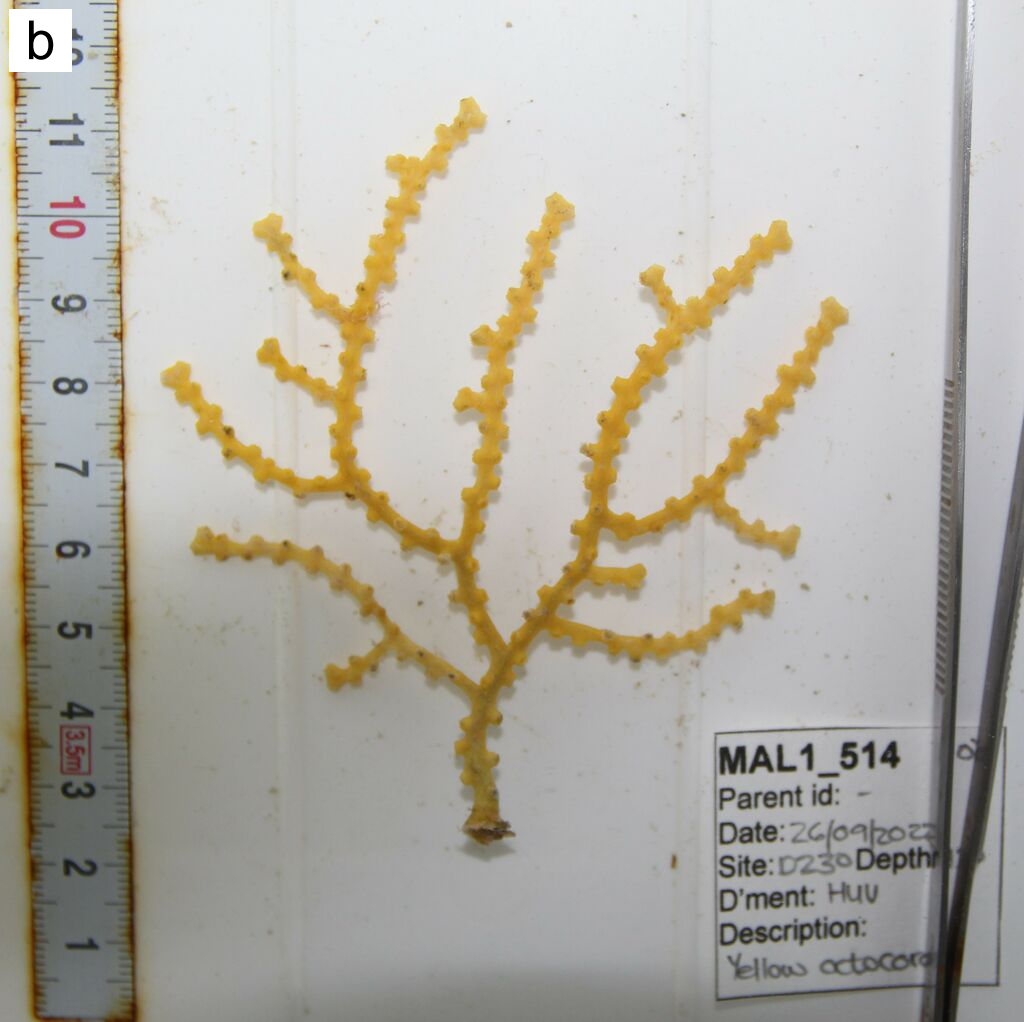
Huvadhu, 120 m, collected specimen MAL1_514;

**Figure 154a.**
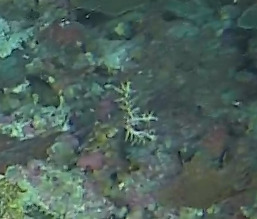
Huvadhu, 60 m;

**Figure 154b.**
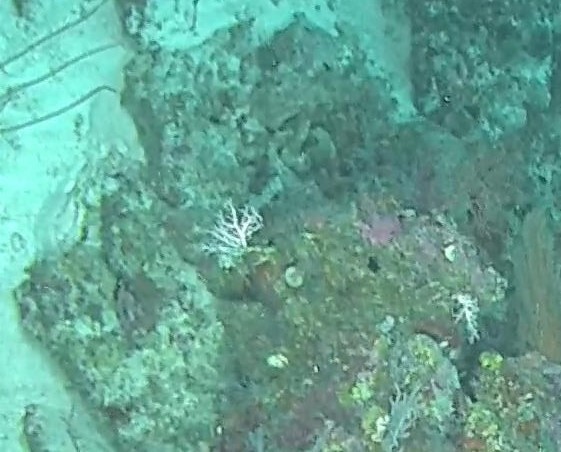
Huvadhu, 60 m.

**Figure 155a.**
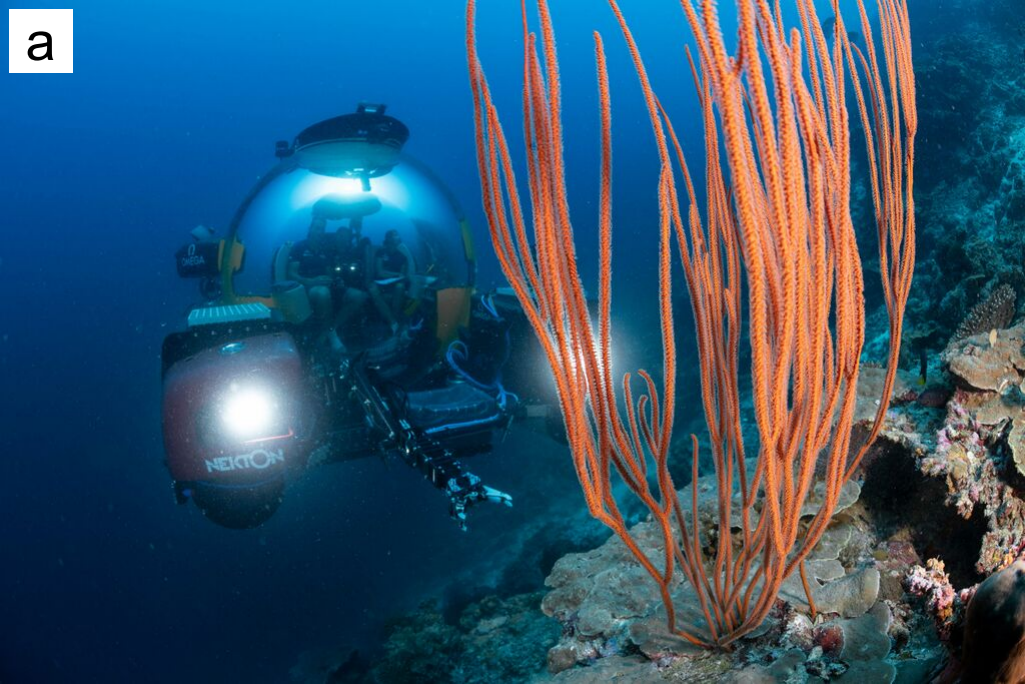
Vaavu, 10-30 m;

**Figure 155b.**
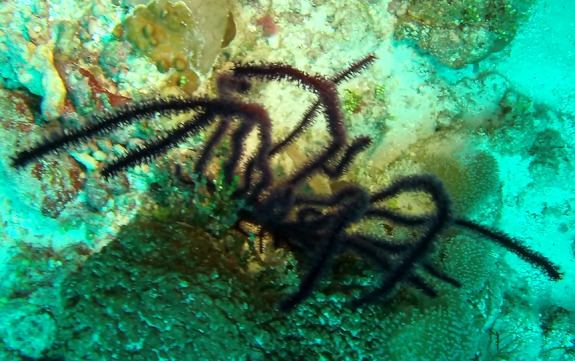
Laamu, 30 m;

**Figure 155c.**
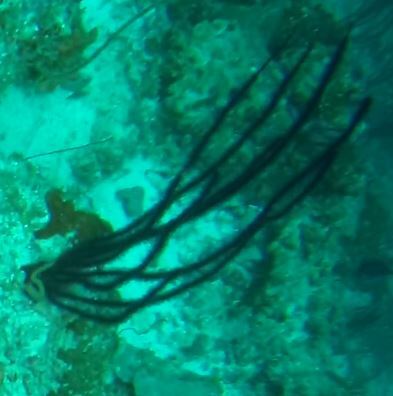
Laamu, 30 m.

**Figure 156a.**
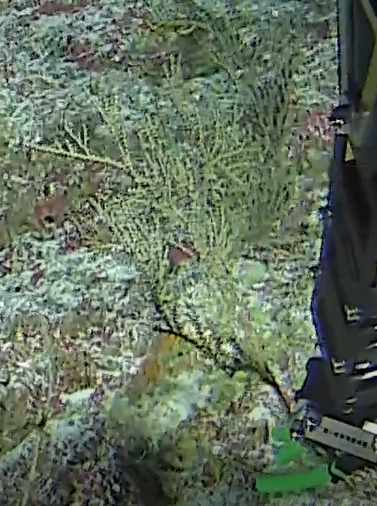
Laamu, 114 m, *in situ* photo of collected specimen MAL1_052;

**Figure 156b.**
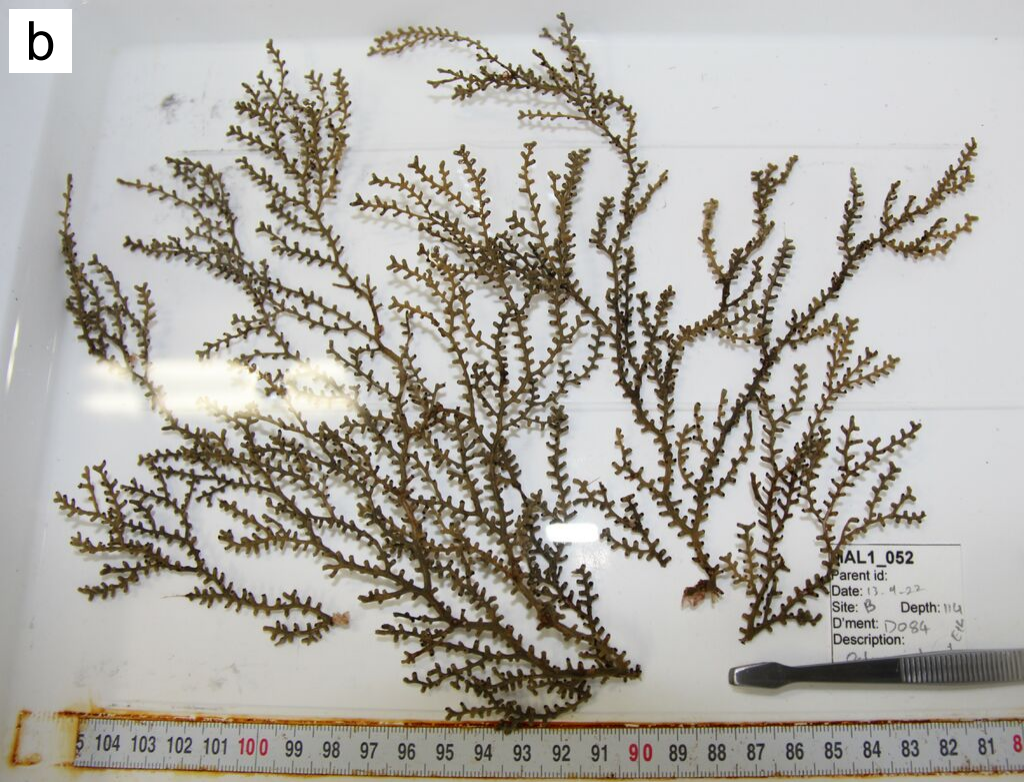
Laamu, 114 m, collected specimen MAL1_052;

**Figure 156c.**
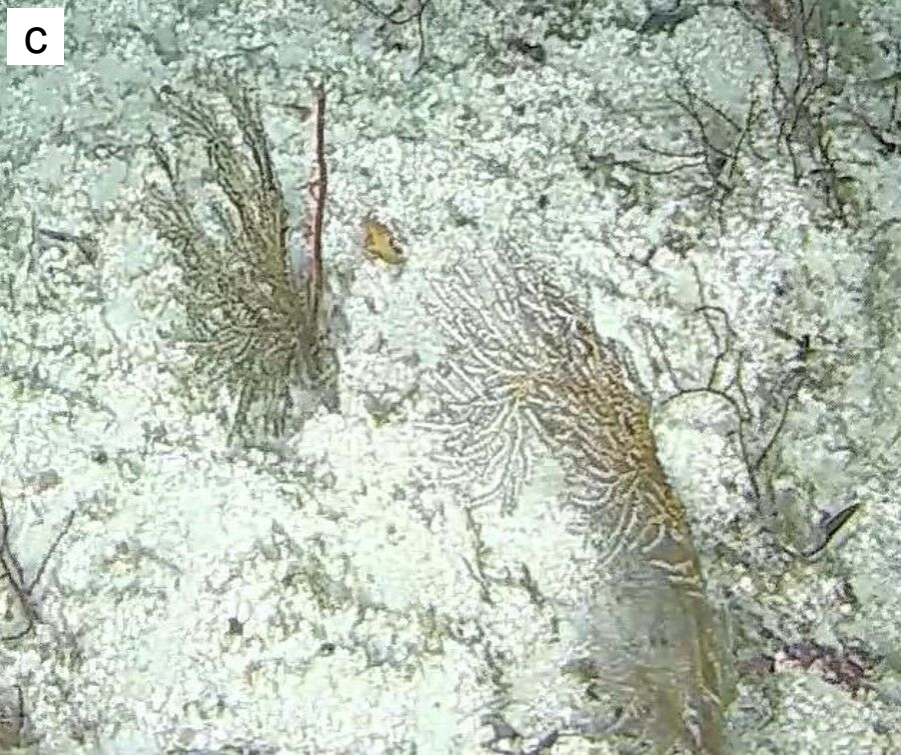
Laamu, 60 m.

**Figure 157a.**
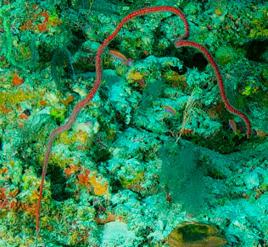
Vaavu, 30 m;

**Figure 157b.**
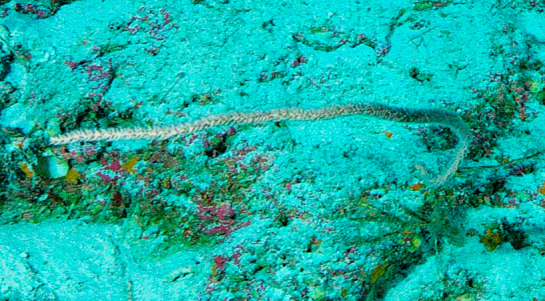
Fuvahmulah, 120 m;

**Figure 157c.**
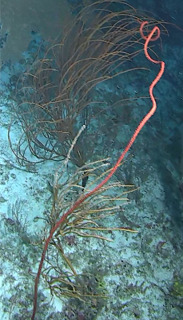
Huvadhu, 60 m.

**Figure 158a.**
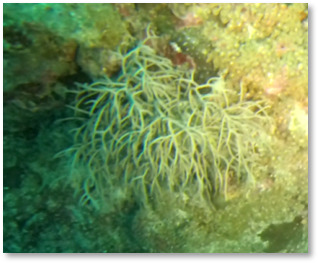
Addu, 30 m;

**Figure 158b.**
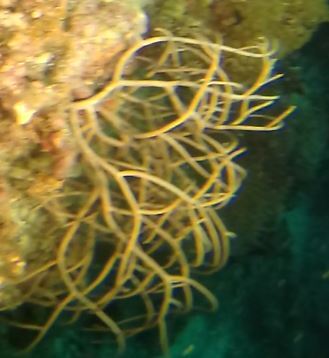
Addu, 30 m;

**Figure 158c.**
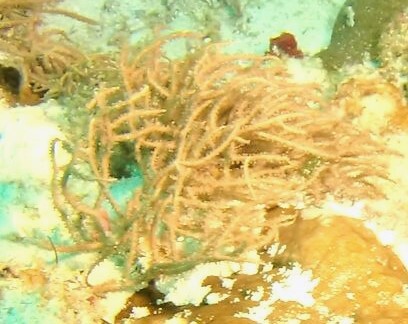
Huvadhu, 30 m.

**Figure 159a.**
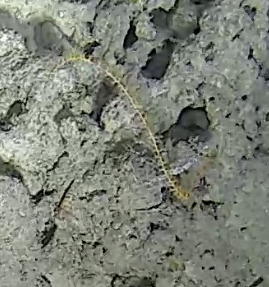
Addu, 243 m, in situ photo of collected specimen MAL_533;

**Figure 159b.**
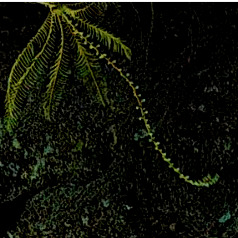
Addu, 255 m, collected specimen MAL_305;

**Figure 159c.**
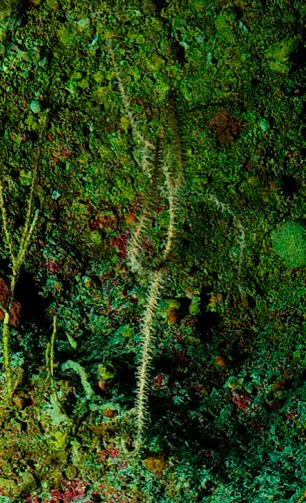
Fuvahmulah, 120 m.

**Figure 160a.**
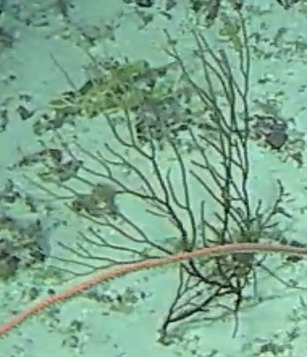
Laamu, 60 m;

**Figure 160b.**
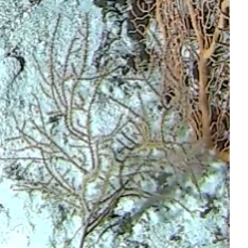
Huvadhu, 120 m.

**Figure 161.**
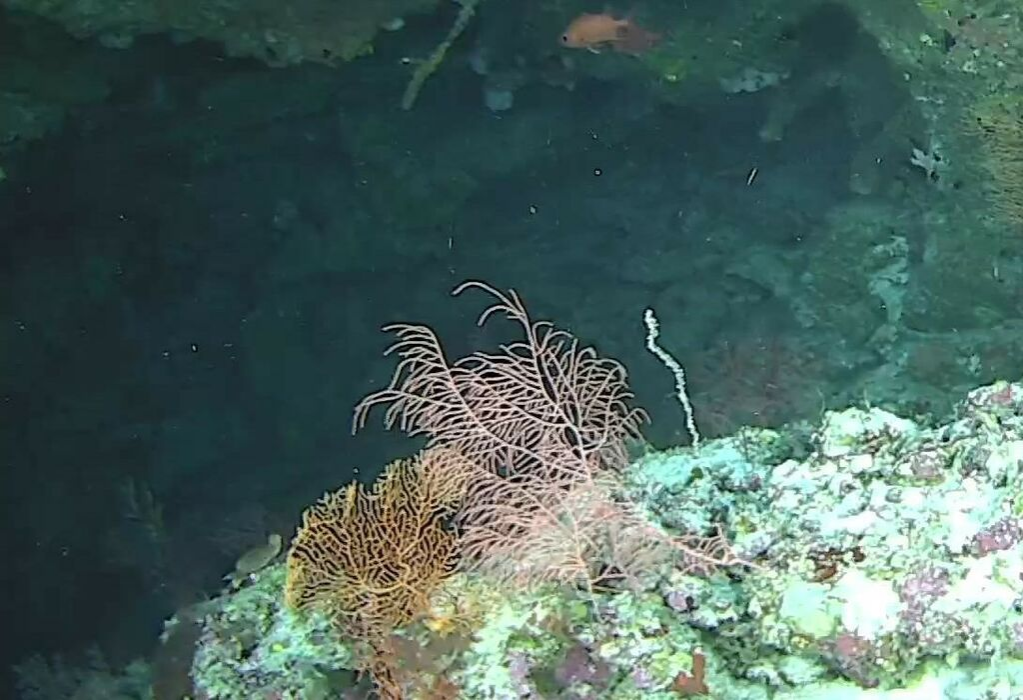
Ellisellidae gen. indet. sp. 8, Addu, 60 m.

**Figure 162a.**
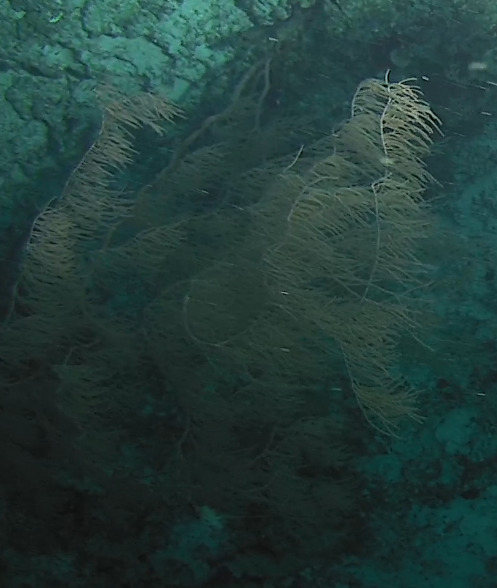
Huvadhu, 120 m;

**Figure 162b.**
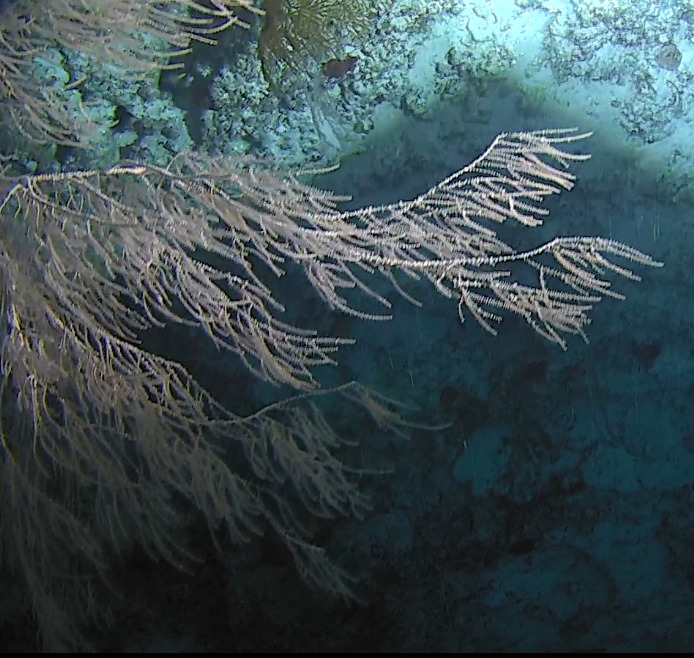
HUvadhu, 120 m.

**Figure 163.**
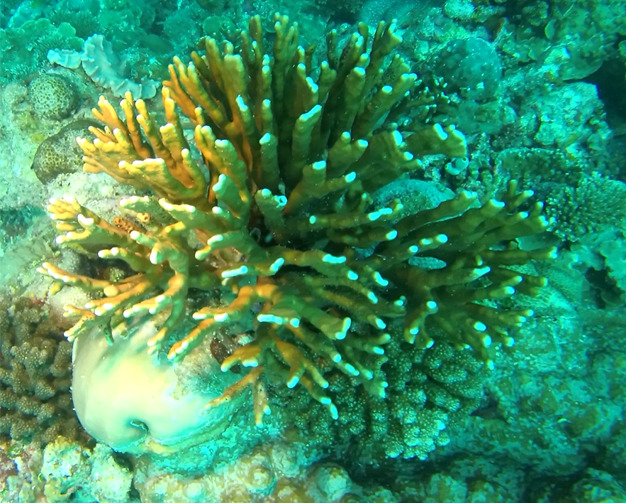
*Heliopora* sp. indet., Vaavu, 10 m.

**Figure 164a.**
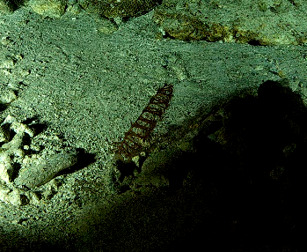
Fuvahmulah, 490 m, *in situ* photo of collected specimen MAL1_367;

**Figure 164b.**
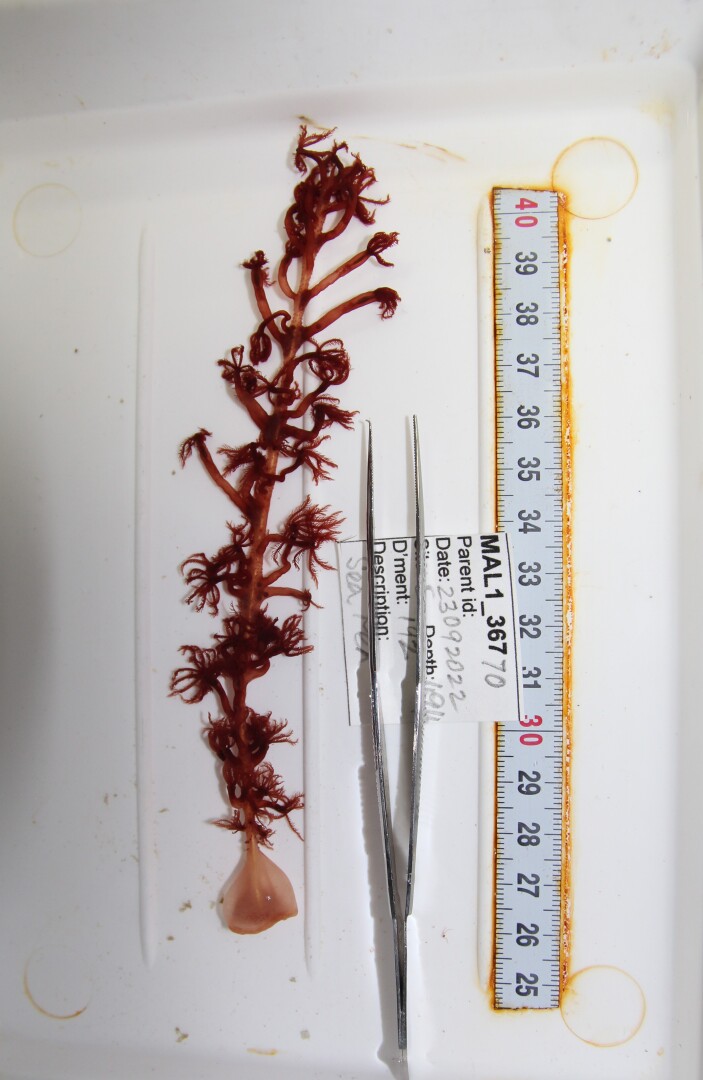
Fuvahmulah, 490 m, collected specimen MAL1_367;

**Figure 164c.**
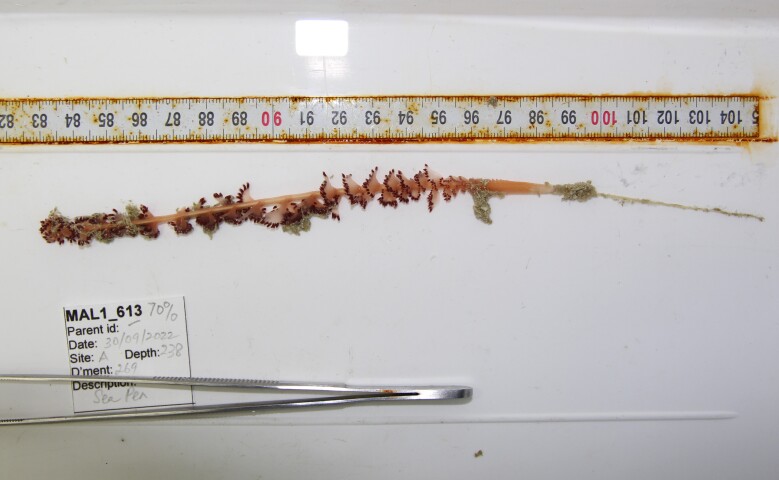
Vaavu, 238 m, collected specimen MAL1_613;

**Figure 164d.**
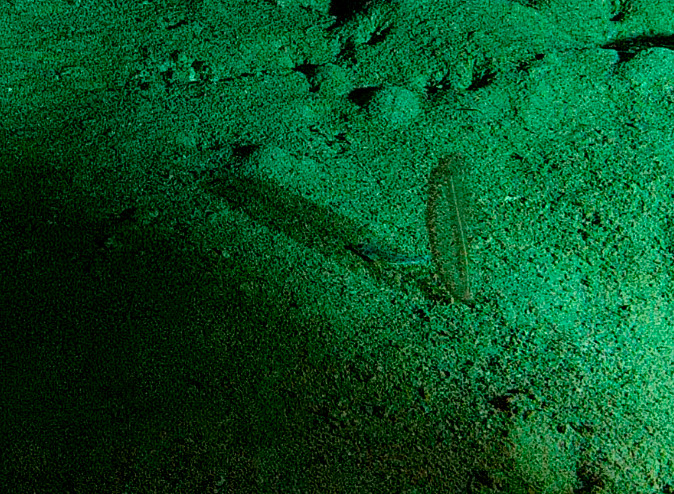
Vaavu, 238 m, *in situ* photo of collected specimen MAL1_613.

**Figure 165a.**
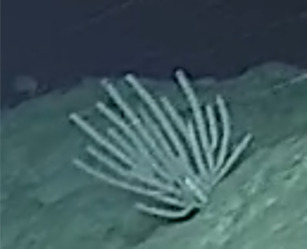
Laamu, 490 m;

**Figure 165b.**
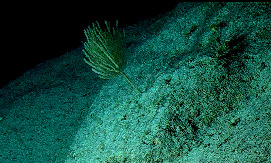
Fuvahmulah, 490 m, *in situ* photo of collected specimen MAL1_402;

**Figure 165c.**
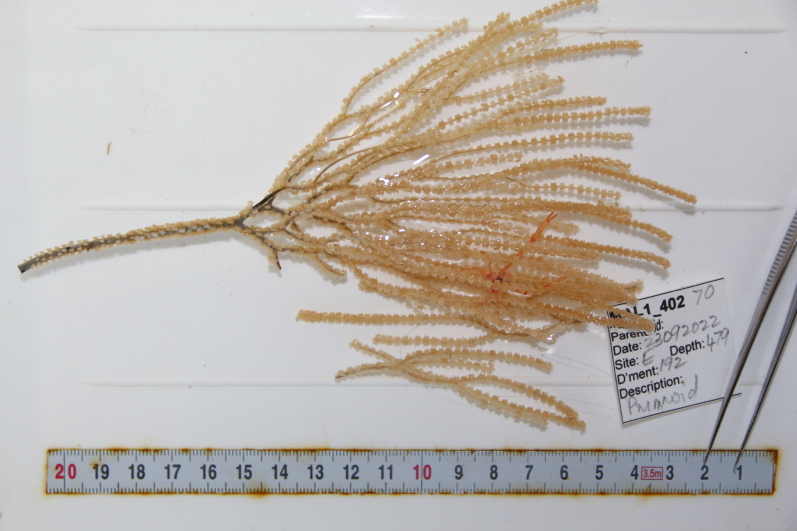
Fuvahmulah, 490 m, collected specimen MAL1_402.

**Figure 166a.**
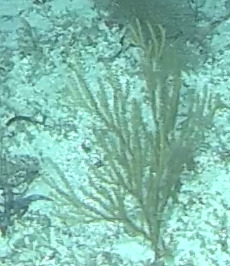
Laamu, 60 m;

**Figure 166b.**
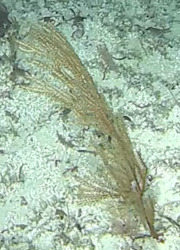
Laamu, 60 m.

**Figure 167a.**
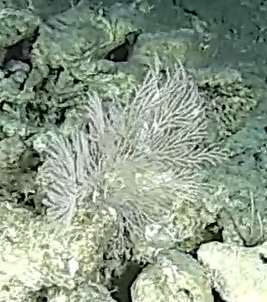
Huvadhu, 250 m, *in situ* photo of collected specimen MAL1_082;

**Figure 167b.**
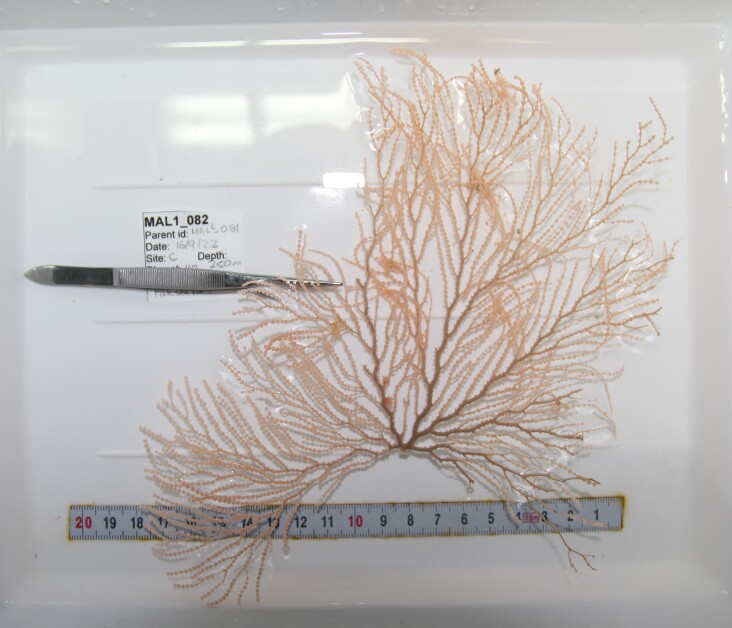
Huvadhu, 250 m, collected specimen MAL1_082.

**Figure 168a.**
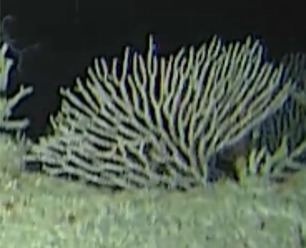
Huvadhu, 490 m;

**Figure 168b.**
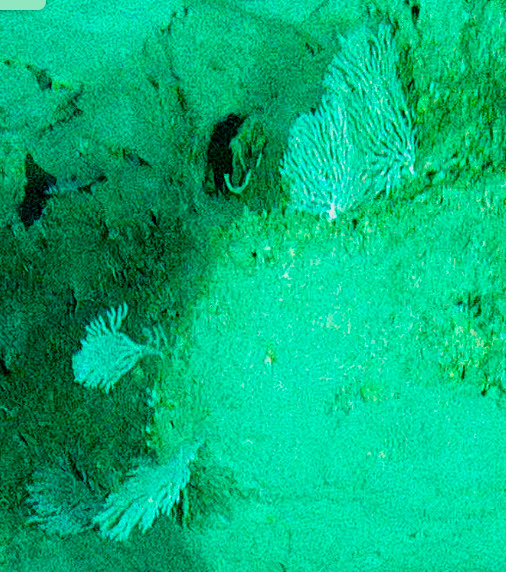
Huvadhu, 490 m.

**Figure 169a.**
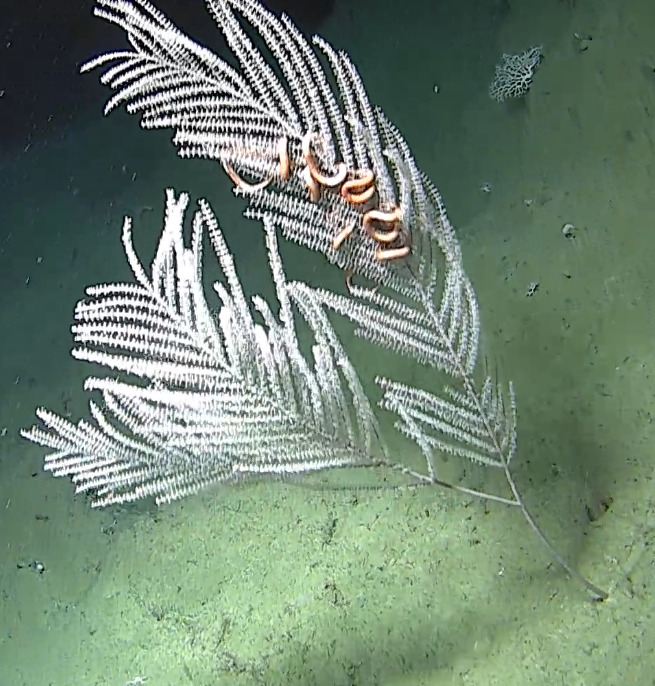
North Male’, 490 m;

**Figure 169b.**
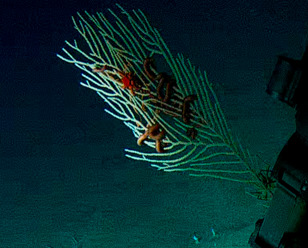
North Male’, 490 m;

**Figure 169c.**
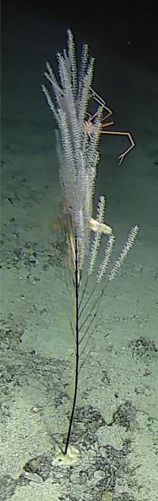
Huvadhu, 490 m.

**Figure 170a.**
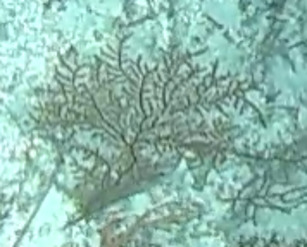
Laamu, 60 m;

**Figure 170b.**
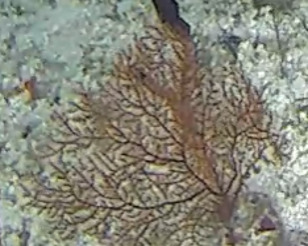
Laamu, 60 m.

**Figure 171a.**
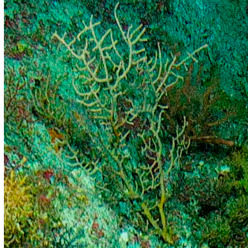
Fuvahmulah, 120 m;

**Figure 171b.**
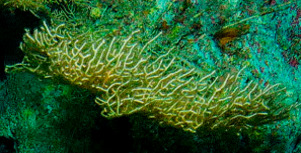
Fuvahmulah, 120 m.

**Figure 172a.**
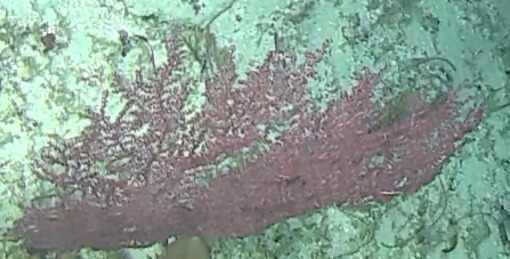
Laamu, 60 m;

**Figure 172b.**
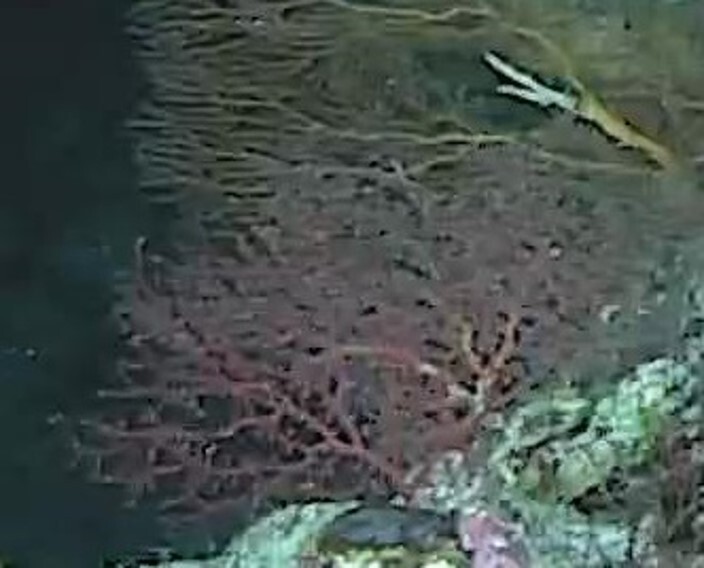
Addu, 60 m.

**Figure 173.**
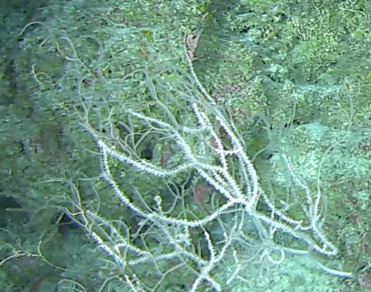
Octocorallia ord. indet. sp. 6, Vaavu, 120 m.

**Figure 174a.**
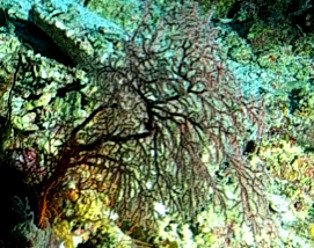
Fuvahmulah, ~ 120 m;

**Figure 174b.**
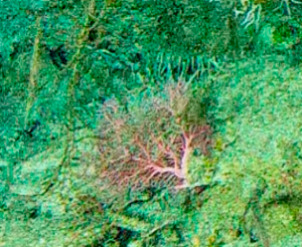
Vaavu, 120 m.

**Figure 175.**
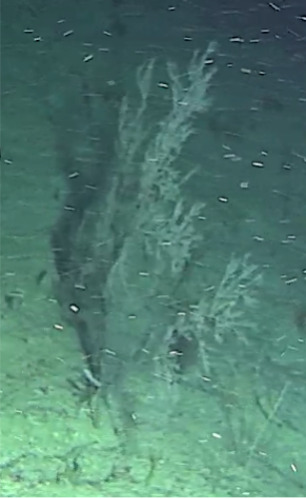
Octocorallia ord. indet. sp. 8, North Male’, 120 m.

**Figure 176a.**
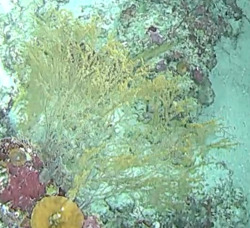
Vaavu, 60 m;

**Figure 176b.**
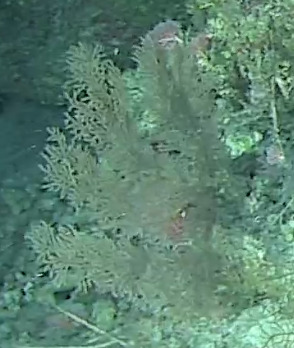
Vaavu, 120 m.

**Figure 177a.**
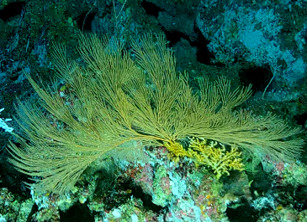
Fuvahmulah, ~120 m;

**Figure 177b.**
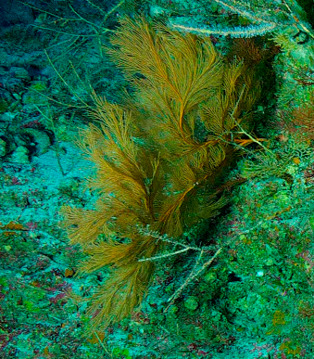
Fuvahmulah, 120 m.

**Figure 178a.**
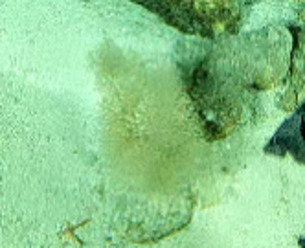
Fuvahmulah, 490 m, *in situ* photo of collected specimen MAL1_376;

**Figure 178b.**
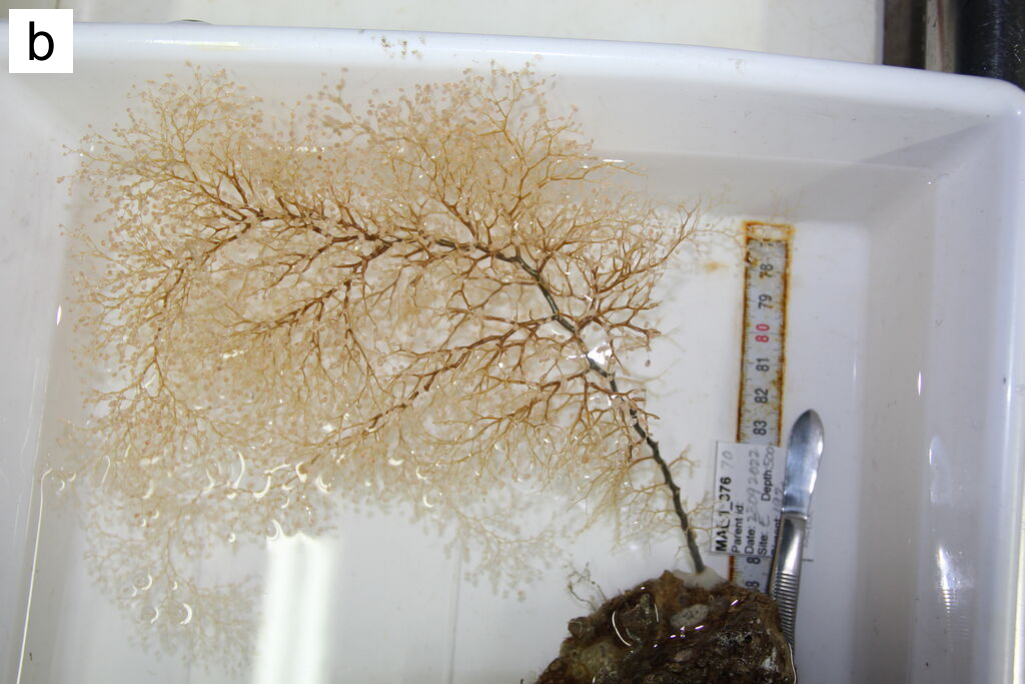
Fuvahmulah, 490 m, collected specimen MAL1_376.

**Figure 179a.**
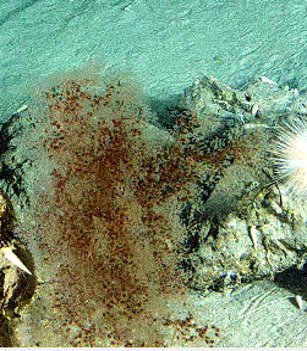
Huvadhu, 120 m, *in situ* photo of collected specimen MAL1_506;

**Figure 179b.**
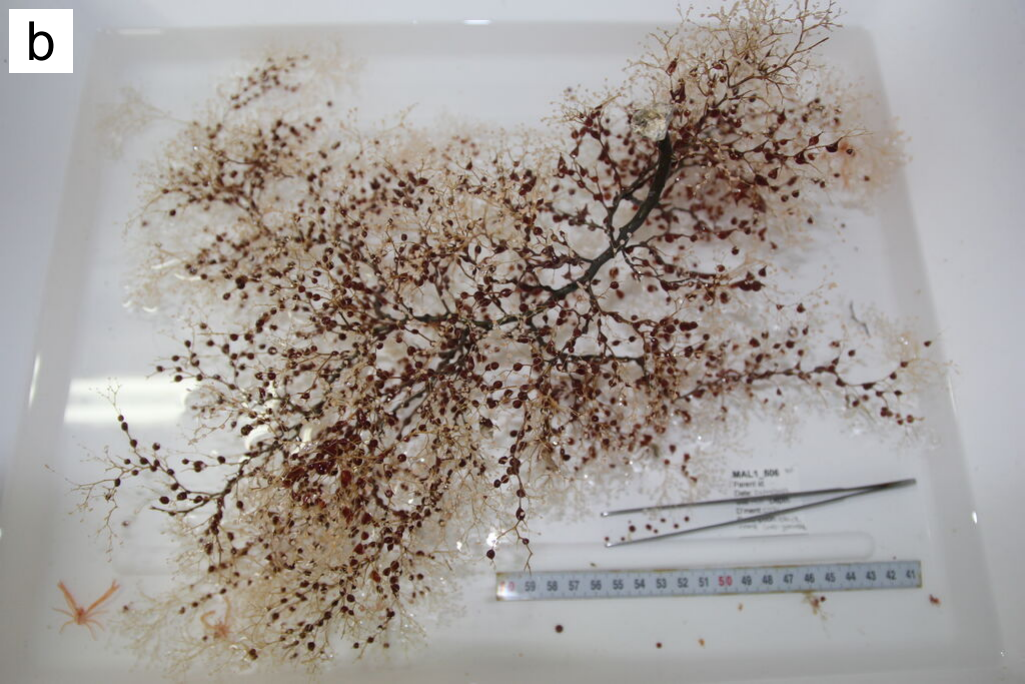
Huvadhu, 120 m, collected specimen MAL1_506.

**Figure 180a.**
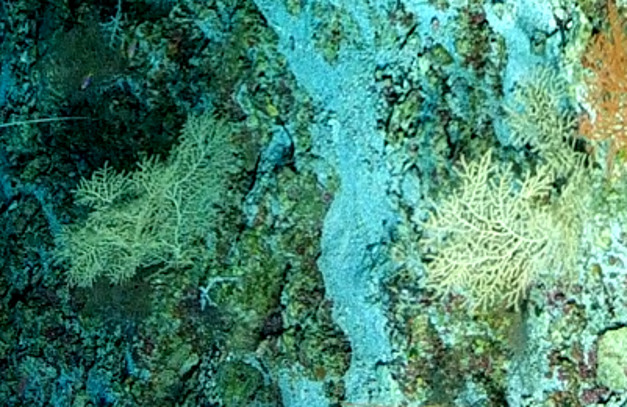
Fuvahmulah, ~ 120 m;

**Figure 180b.**
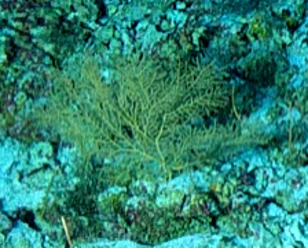
Fuvahmulah, ~ 120 m.

**Figure 181.**
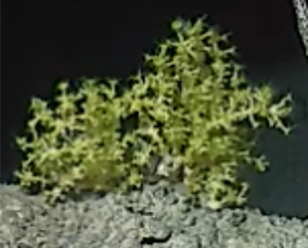
Octocorallia ord. indet. sp. 14, Addu, 250 m.

**Figure 182.**
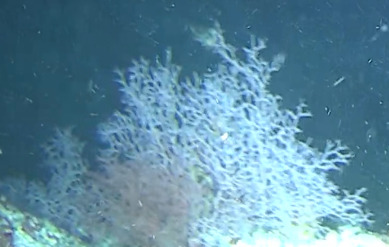
Octocorallia ord. indet. sp. 15, Laamu, 60 m.

**Figure 183.**
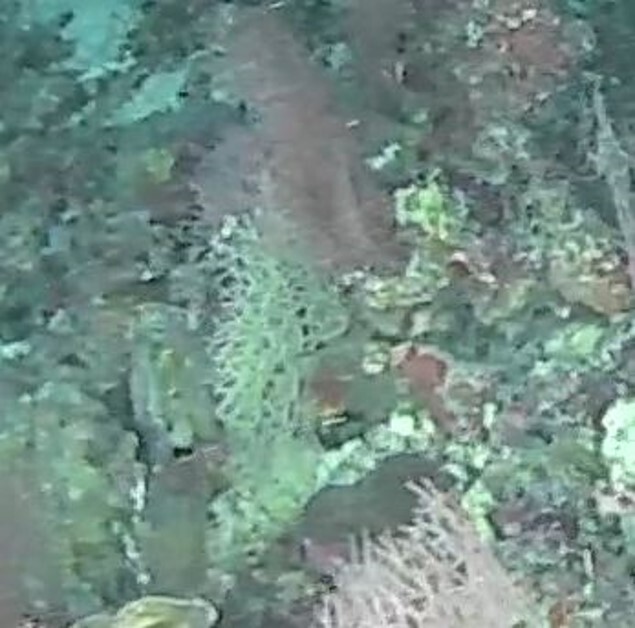
Octocorallia ord. indet. sp. 18, Vaavu, 60 m.

**Figure 184.**
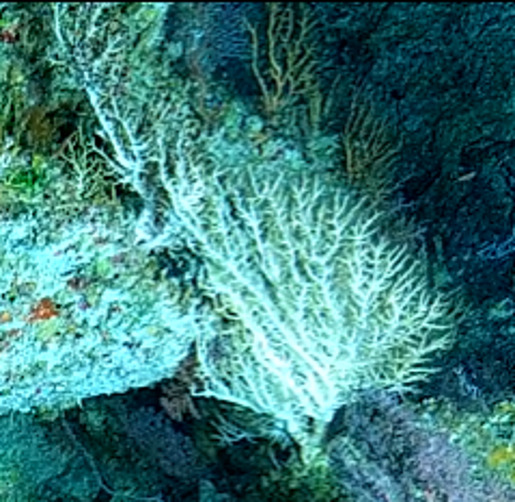
Octocorallia ord. indet. sp. 19, Fuvahmulah, 490 m.

**Figure 185.**
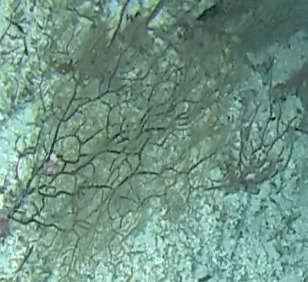
Octocorallia ord. indet. sp. 20, Laamu, 60 m.

**Figure 186.**
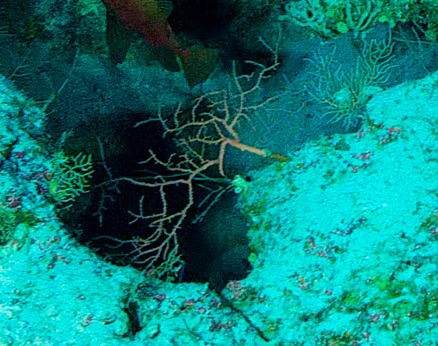
Octocorallia ord. indet. sp. 21, Fuvahmulah, 120 m.

**Figure 187a.**
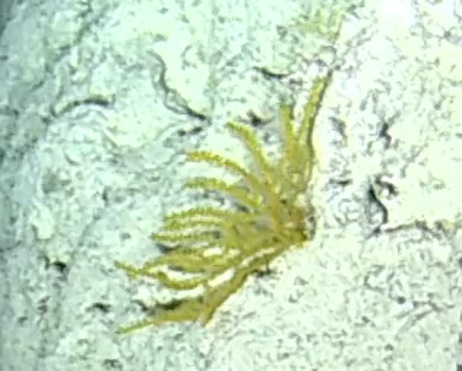
Fuvahmulah, 490 m;

**Figure 187b.**
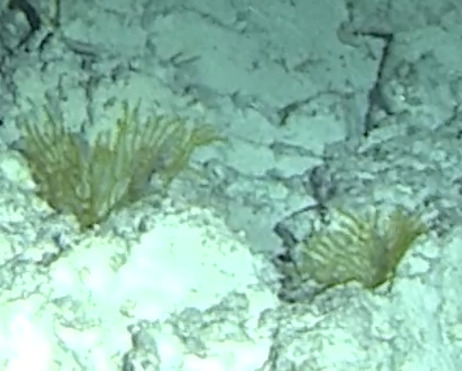
Fuvahmulah, 490 m.

**Figure 188a.**
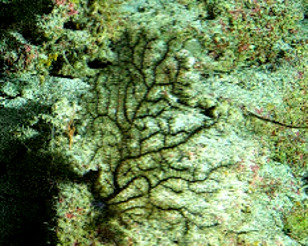
Vaavu, 124 m, *in situ* photo of collected specimen MAL1_645;

**Figure 188b.**
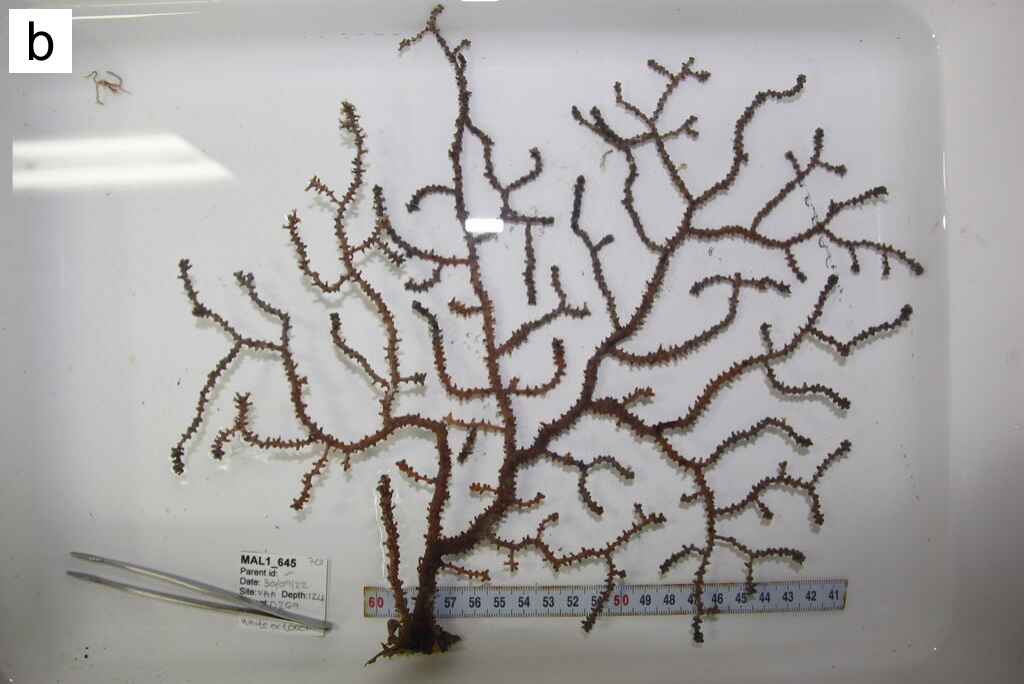
Vaavu, 124 m, collected specimen MAL1_645.

**Figure 189a.**
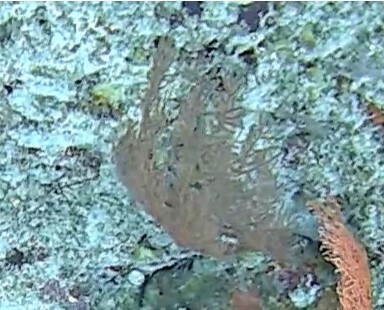
Laamu, 60 m;

**Figure 189b.**
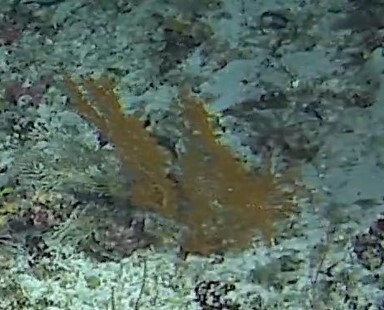
Laamu, 60 m.

**Figure 190.**
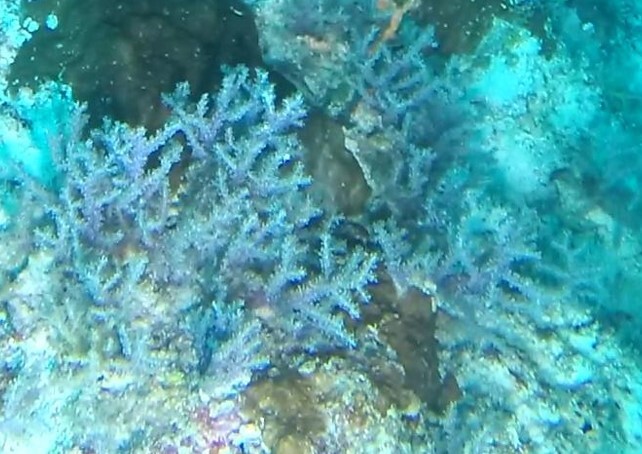
Octocorallia ord. indet. sp. 27, Huvadhu, 30 m.

**Figure 191.**
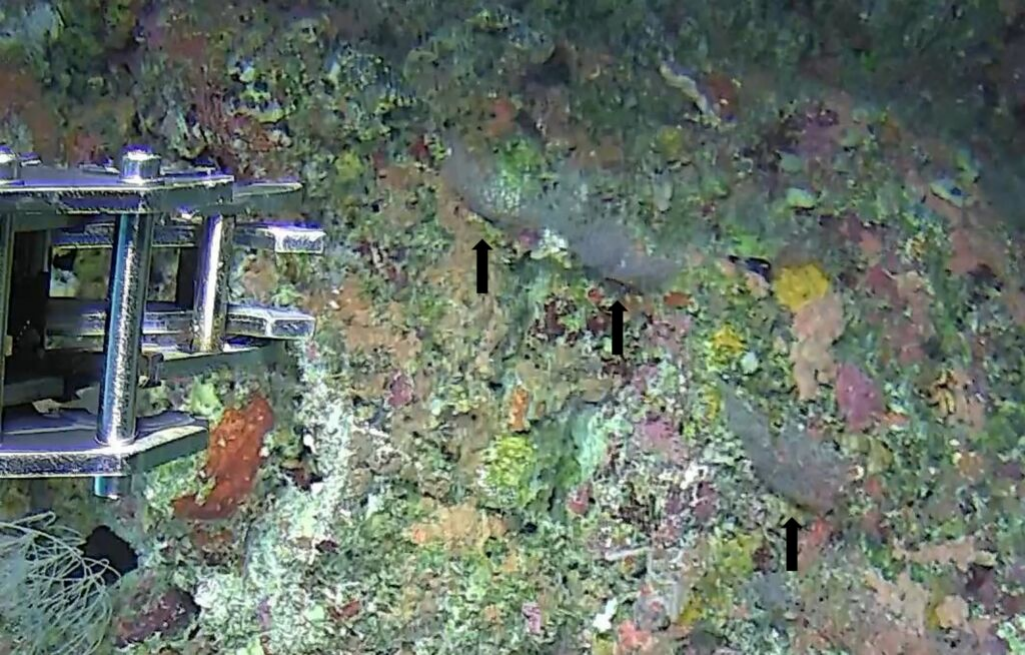
Octocorallia ord. indet. sp. 29, Addu, 60 m.

**Figure 192a.**
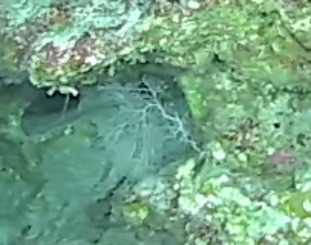
Addu, 120 m;

**Figure 192b.**
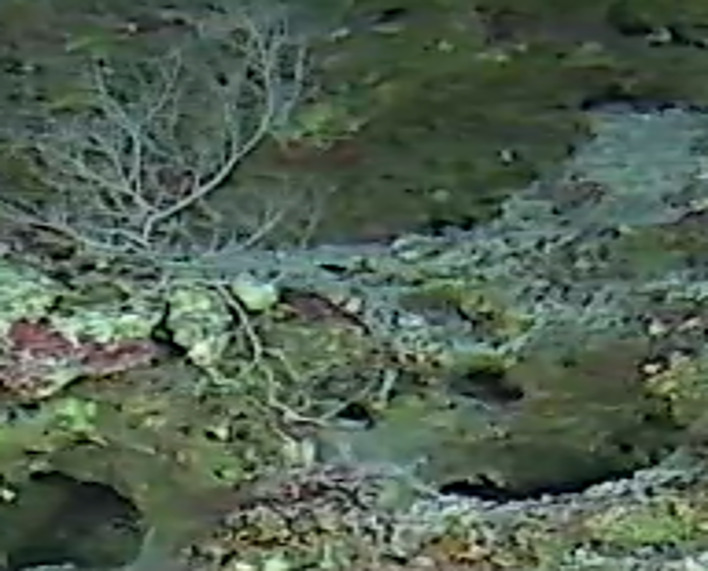
Addu, 120 m.

**Figure 193.**
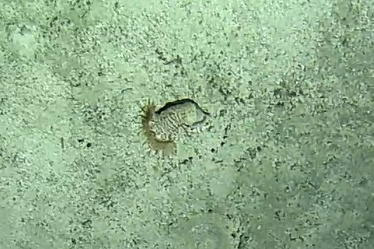
Ceriantharia stet., North Male’, 250 m.

**Figure 194a.**
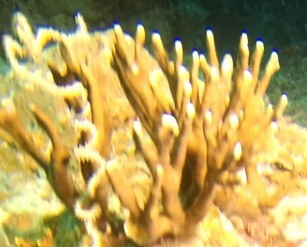
Addu, 30 m;

**Figure 194b.**
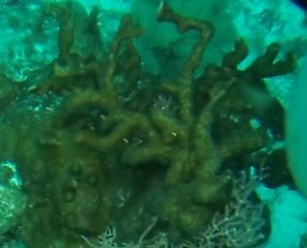
Laamu, 30 m.

**Figure 195a.**
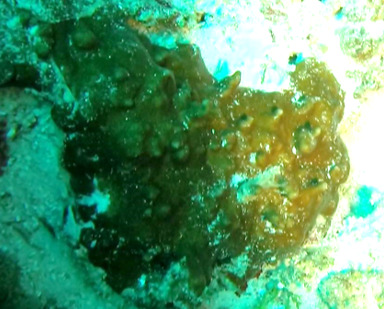
Laamu, 30 m;

**Figure 195b.**
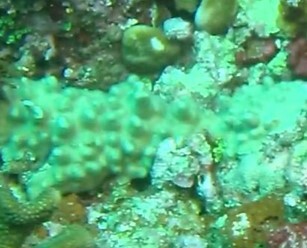
Laamu, 10 m.

**Figure 196a.**
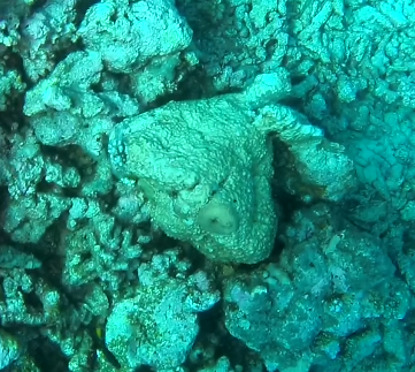
Addu, 10 m;

**Figure 196b.**
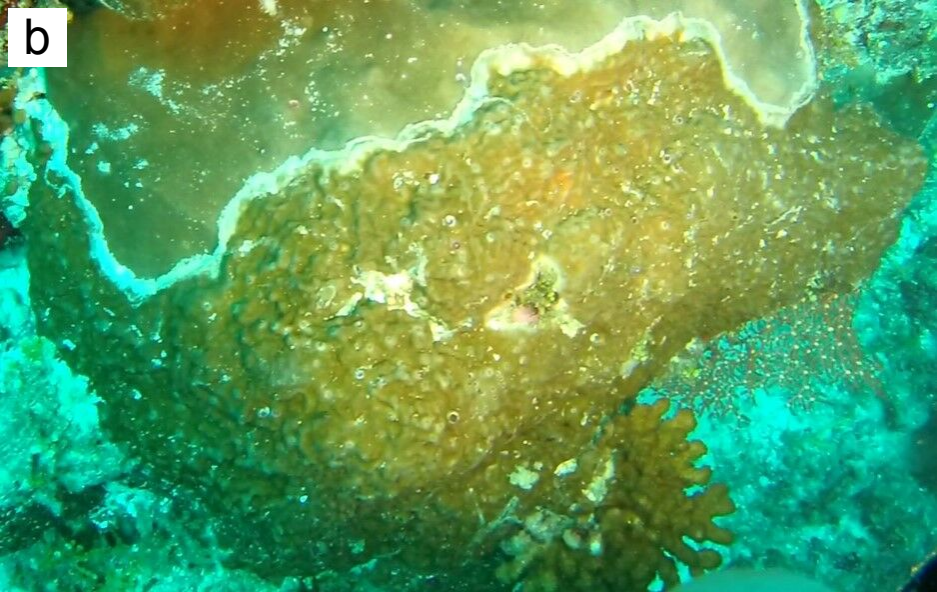
Laamu, 30 m.

**Figure 197a.**
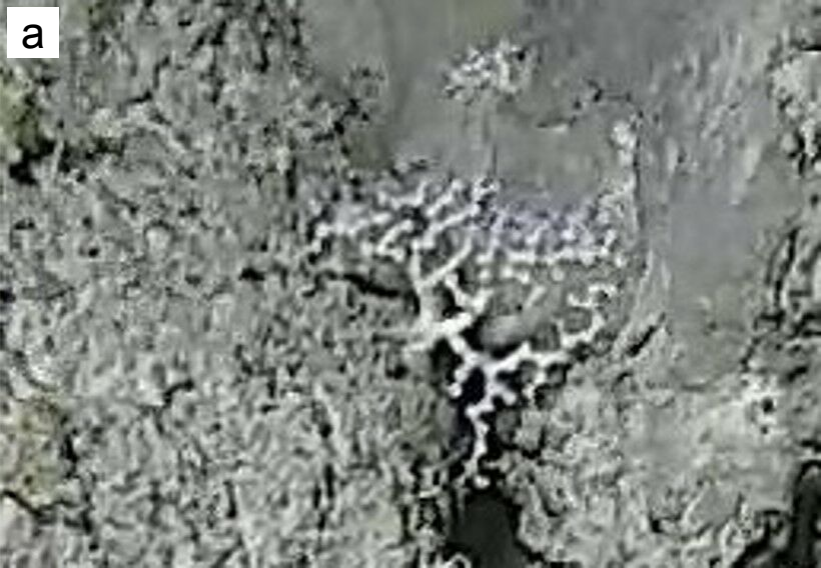
Addu, 250 m;

**Figure 197b.**
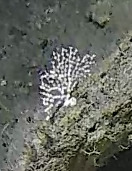
Huvadhu, 250 m.

**Figure 198a.**
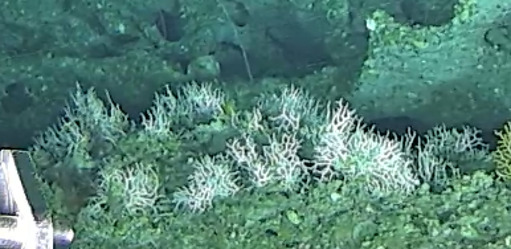
Addu, 120 m;

**Figure 198b.**
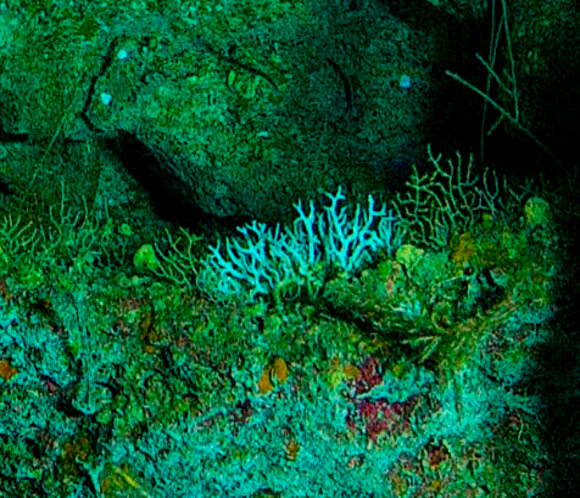
Fuvahmulah, 120 m.

**Figure 199.**
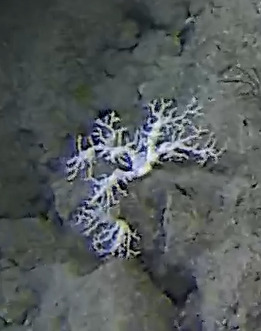
Stylasteridae gen. indet. sp. 5, Huvadhu, 250 m.

**Figure 200.**
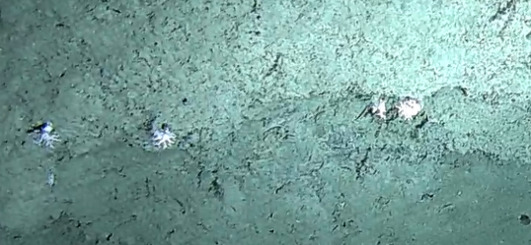
Stylasteridae gen. indet. sp. 6, North Male’, 490 m.

**Figure 201a.**
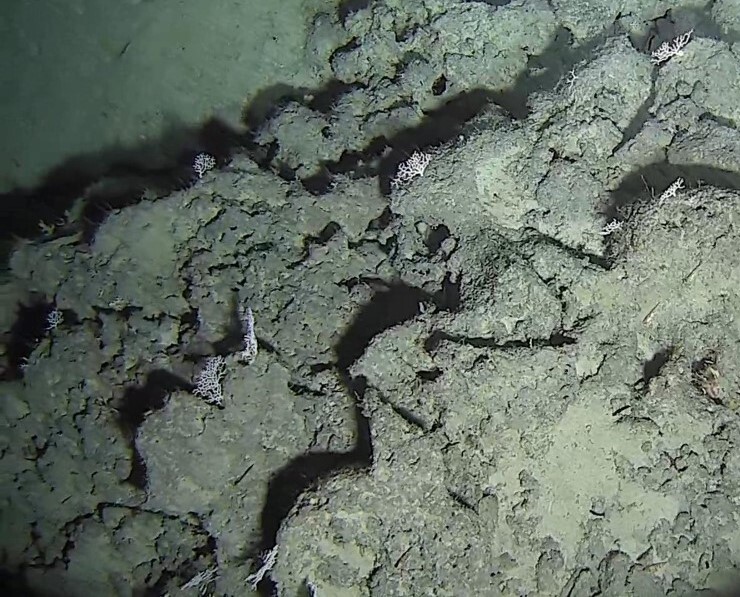
Addu, 250 m;

**Figure 201b.**
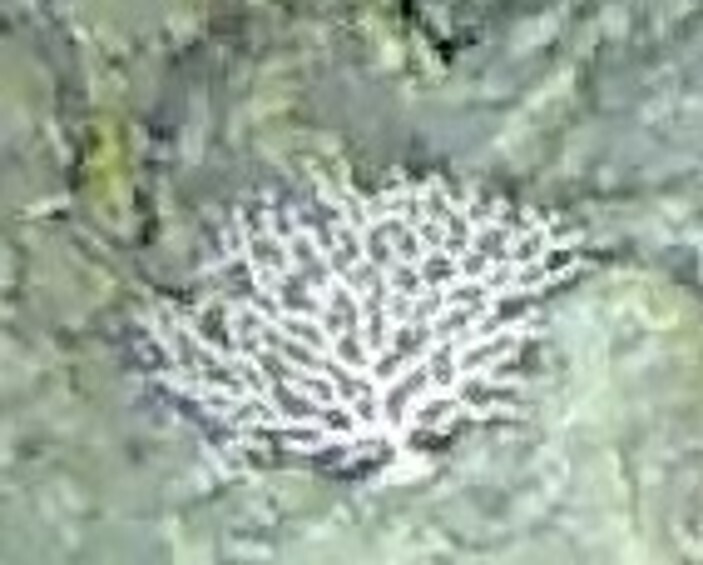
Addu, 250 m.

**Figure 202a.**
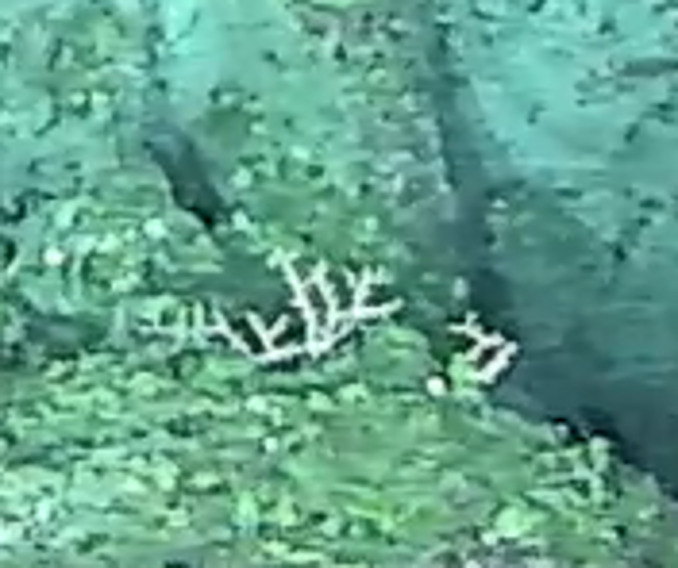
Addu, 120 m;

**Figure 202b.**
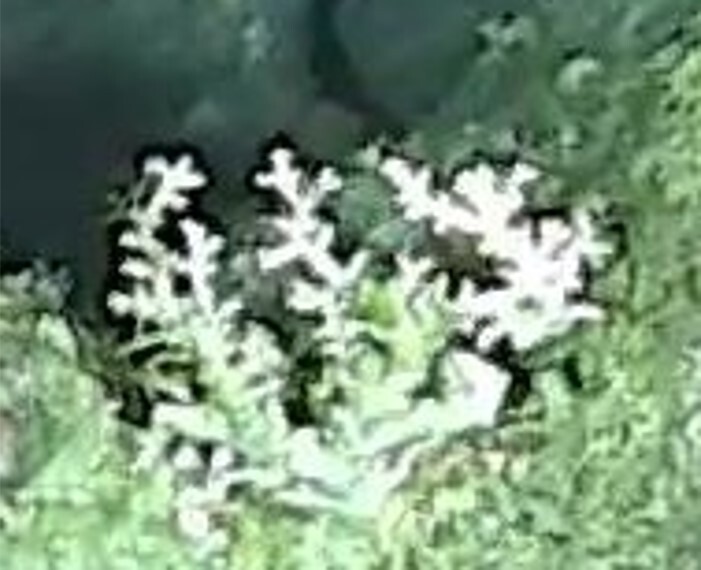
Addu, 120 m.

**Figure 203.**
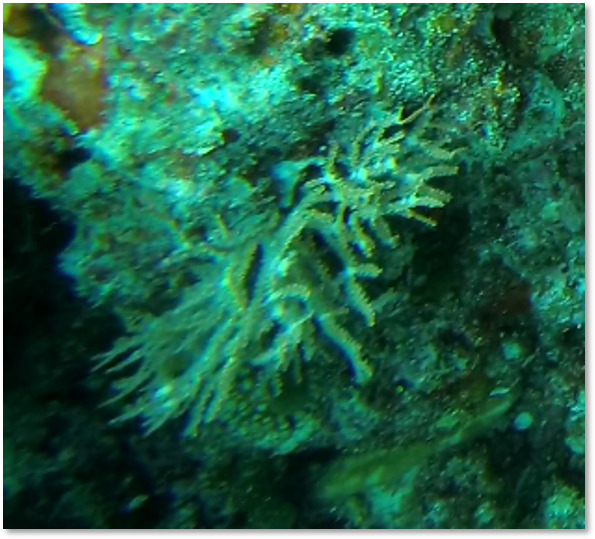
Hydrozoa ord. indet. sp. 1, North Male’, 10 m.

**Figure 204.**
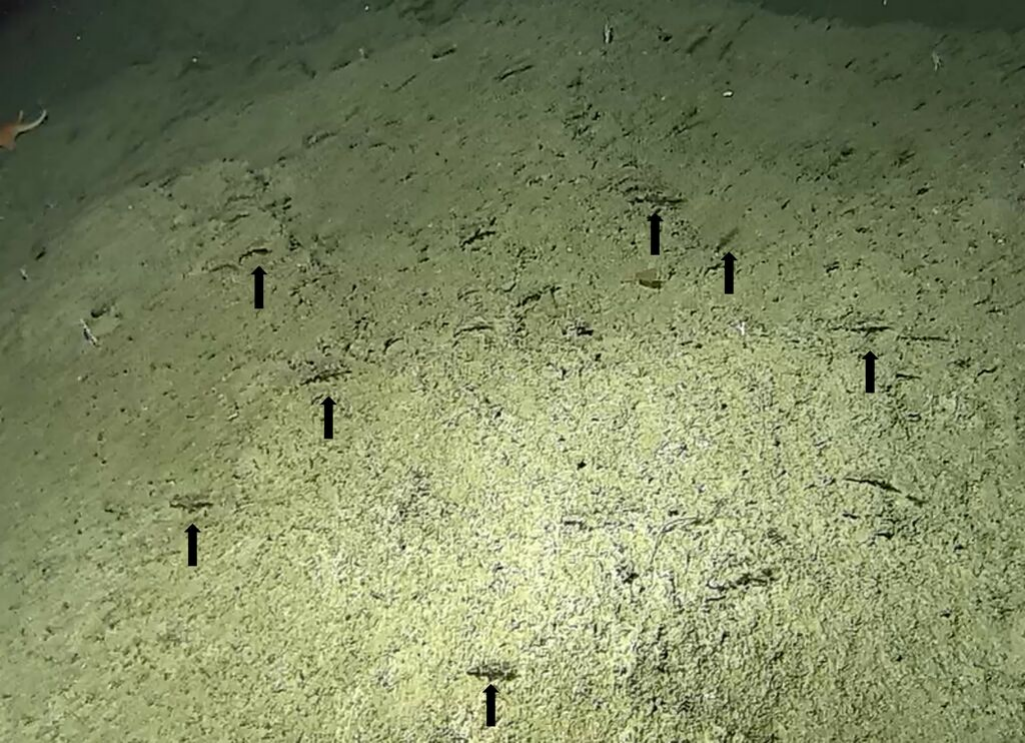
Hydrozoa ord. indet. sp. 4, North Male’, 490 m.

**Figure 205a.**
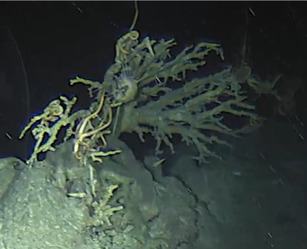
Addu, 490 m;

**Figure 205b.**
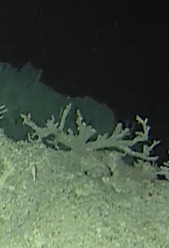
Huvadhu, 490 m.

**Figure 206a.**
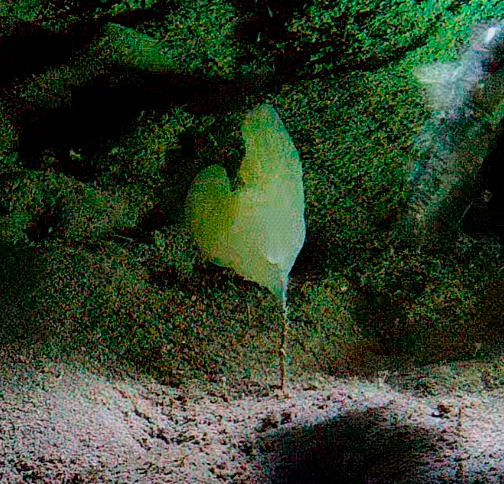
Vaavu, 238 m, *in situ* of collected specimen MAL1_616;

**Figure 206b.**
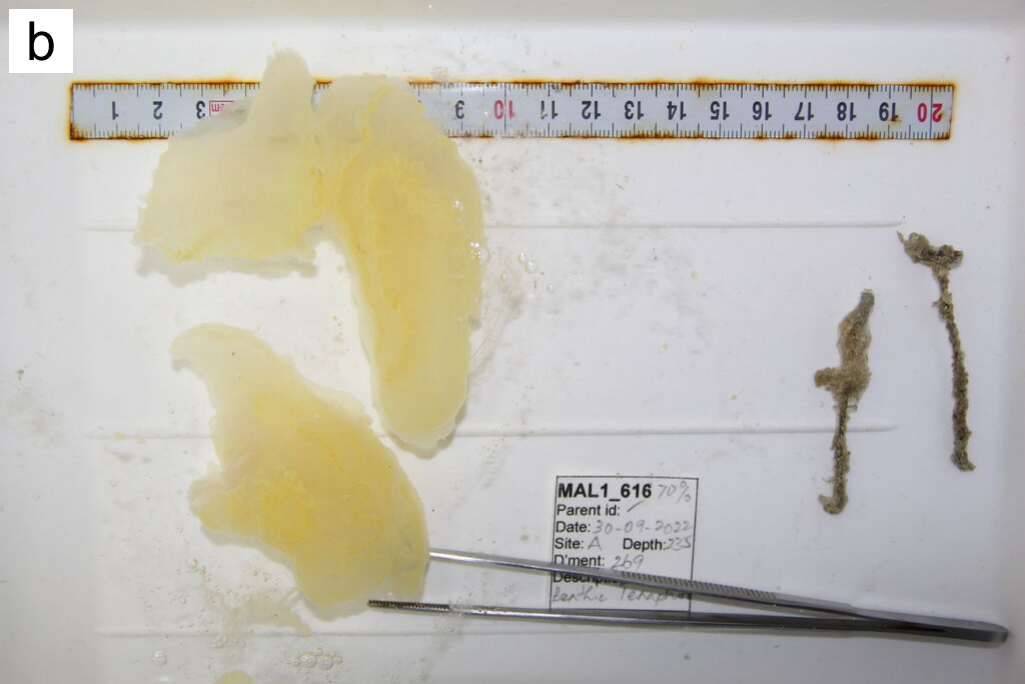
Vaavu, 238 m, collected specimen MAL1_616.

**Figure 207.**
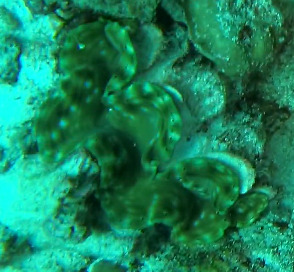
*Tridacna* sp. indet., North Male’, 10 m.

**Figure 208a.**
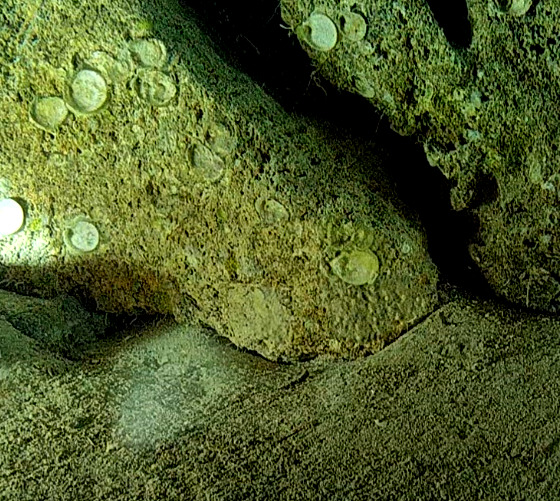
Fuvahmulah, 490 m;

**Figure 208b.**
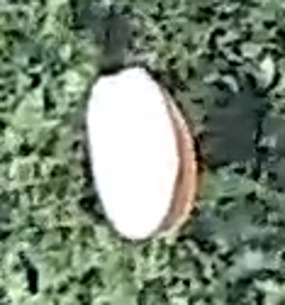
Laamu, 490 m.

**Figure 209.**
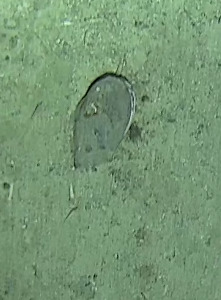
Bivalvia ord. indet. sp. 2, Huvadhu, 490 m.

**Figure 210.**
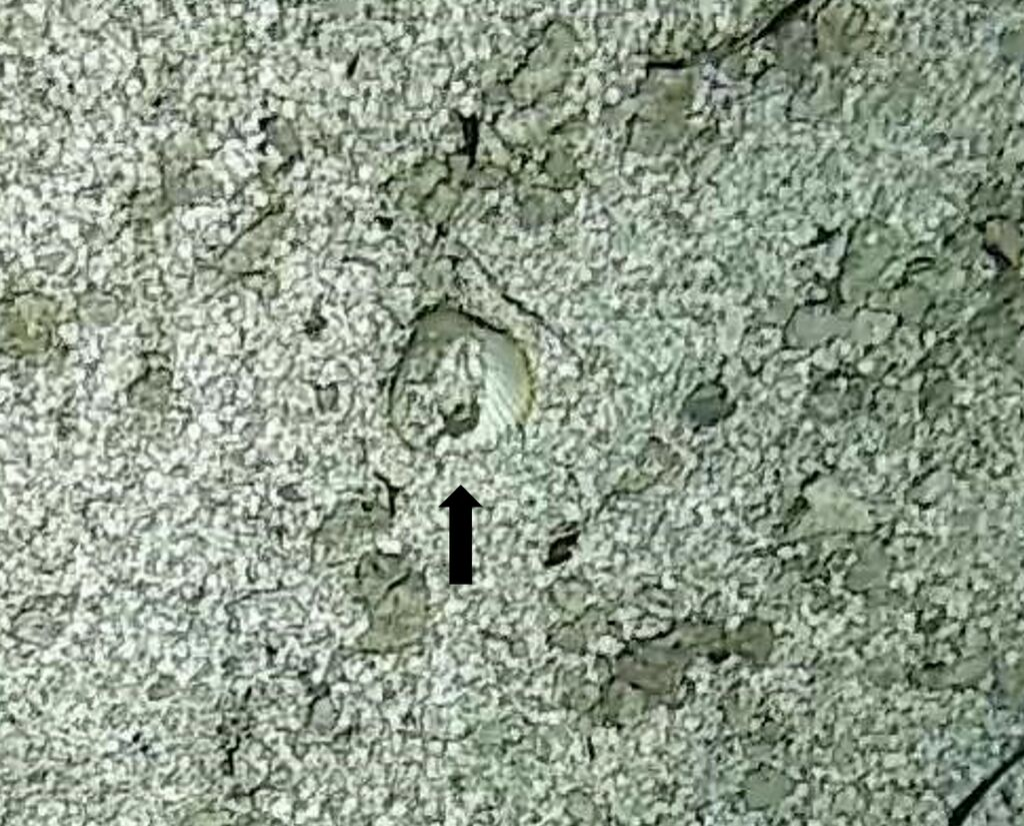
Bivalvia ord. indet. sp. 3, Laamu, 250 m.

**Figure 211a.**
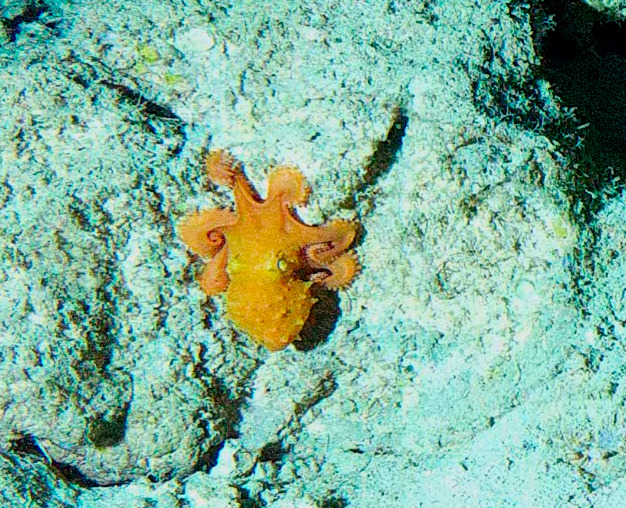
Vaavu, 250 m;

**Figure 211b.**
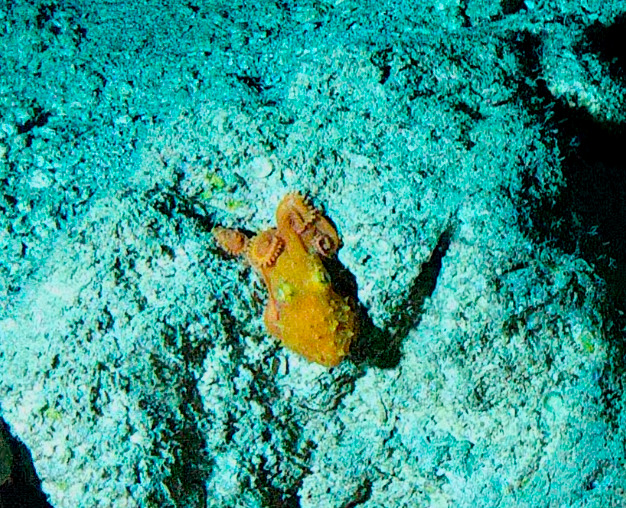
Vaavu, 250 m.

**Figure 212.**
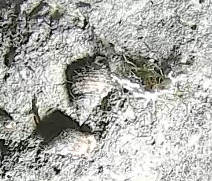
Strombidae gen. indet. sp. 1, Addu, 250 m.

**Figure 213.**
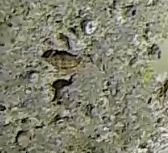
Strombidae gen. indet. sp. 2, Huvadhu, ~ 120-250 m.

**Figure 214a.**
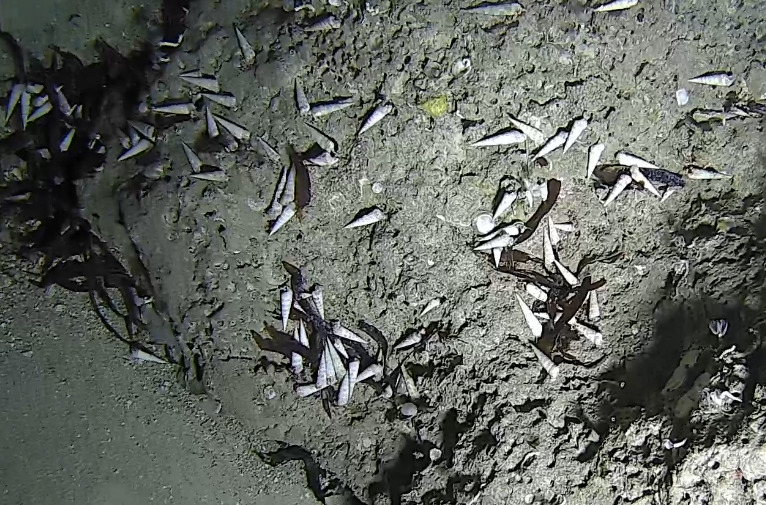
Huvadhu, 490 m;

**Figure 214b.**
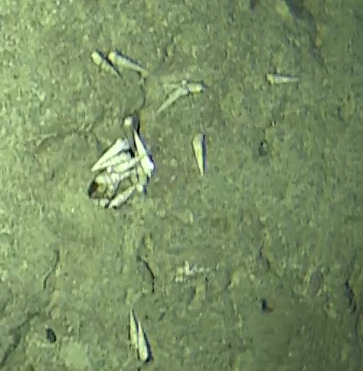
Laamu, 490 m;

**Figure 214c.**
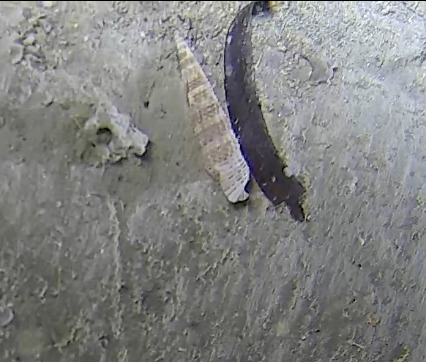
Huvadhu, 490 m.

**Figure 215.**
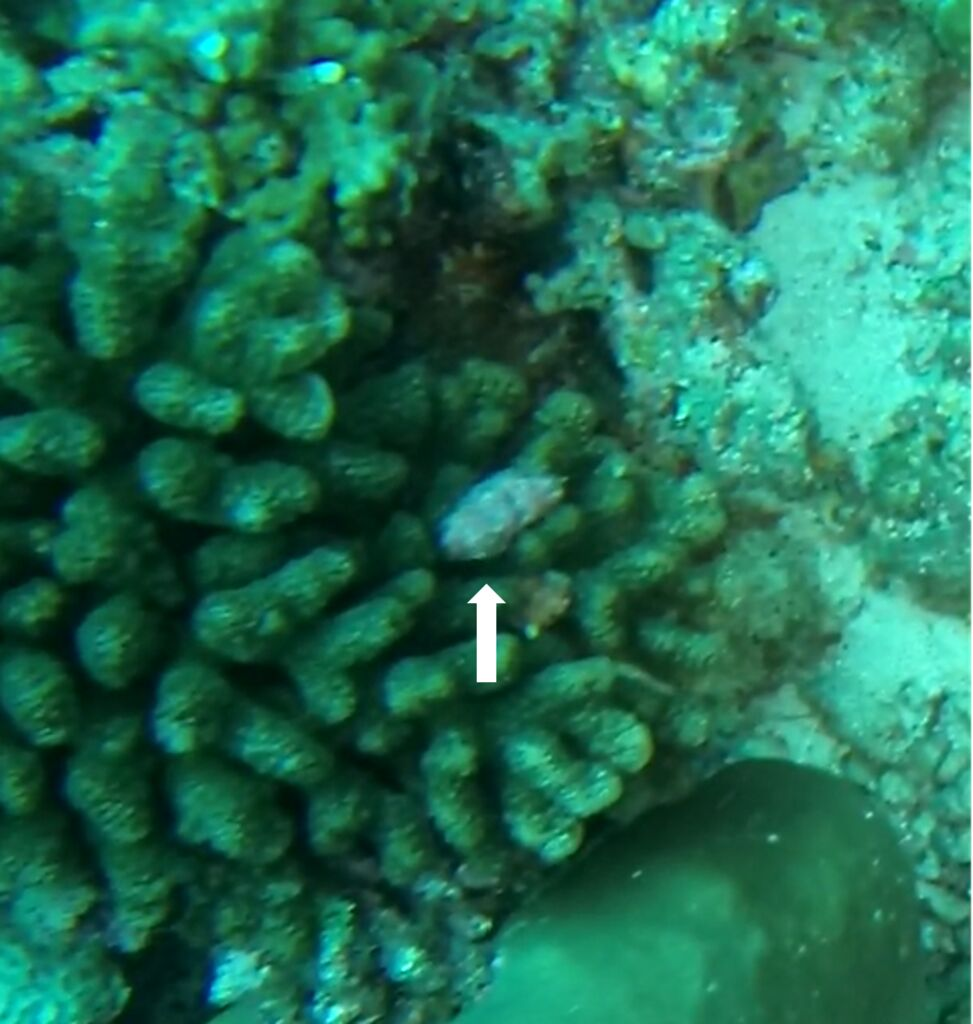
*Drupella* sp. indet., North Male’, 10 m.

**Figure 216.**
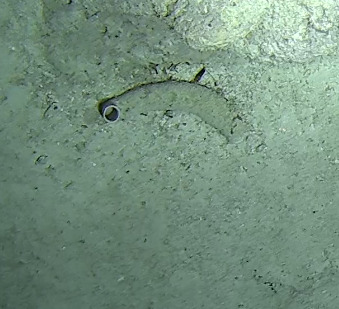
Polychaeta ord. indet. sp. 1, Addu, 250 m.

**Figure 217a.**
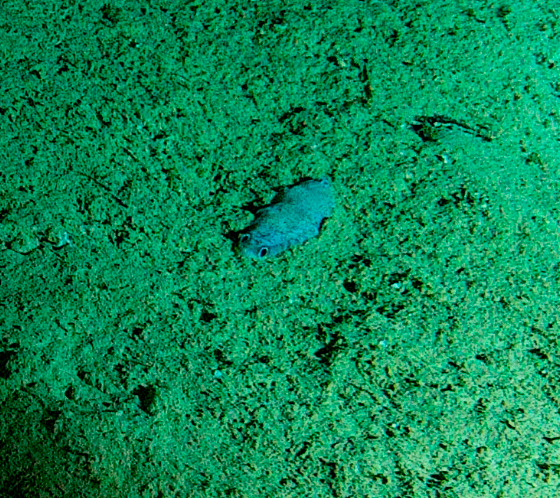
North Male’, 490 m;

**Figure 217b.**
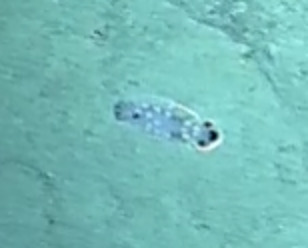
Huvadhu, 490 m.

**Figure 218.**
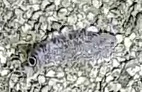
Polychaeta ord. indet. sp. 3, Laamu, 490 m.

**Figure 219.**
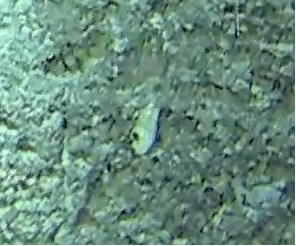
Polychaeta ord. indet. sp. 4, Laamu, 490 m.

**Figure 220.**
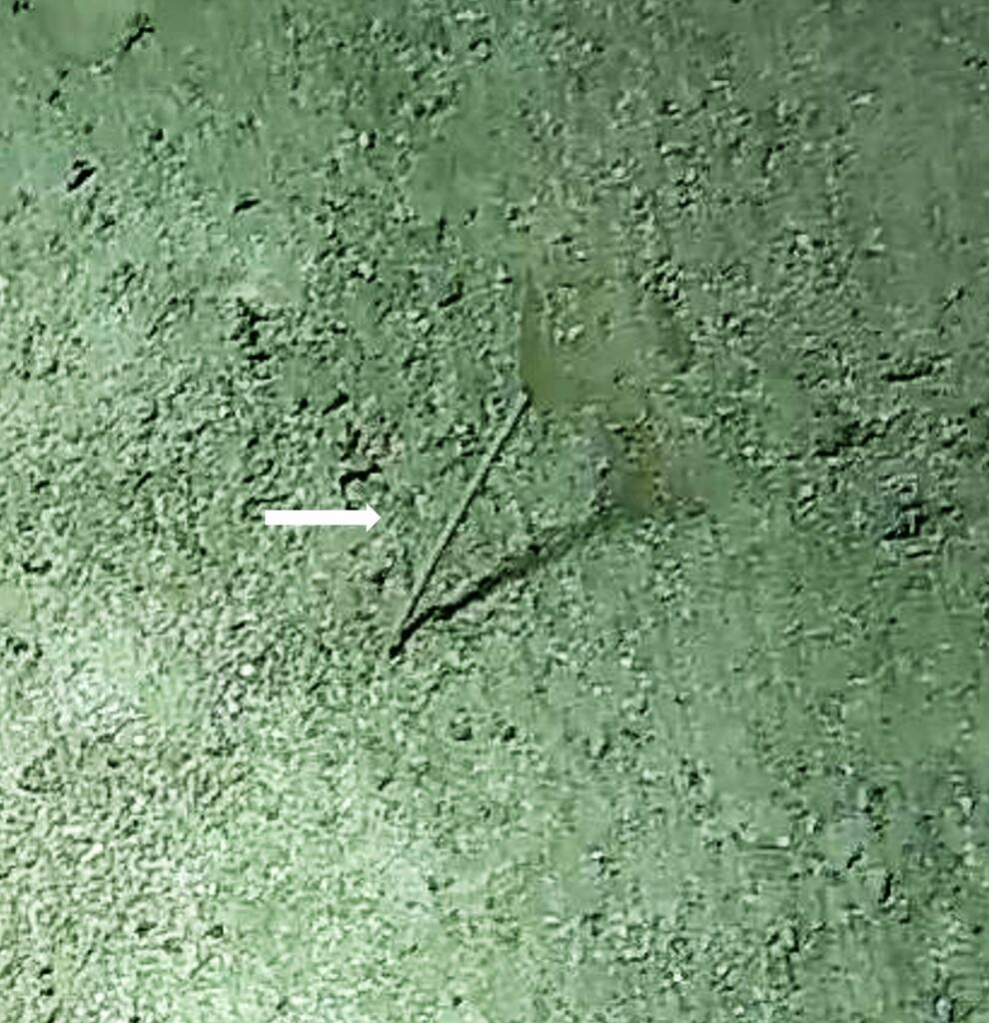
Polychaeta ord. indet. sp. 5, North Male', 250 m.

**Figure 221a.**
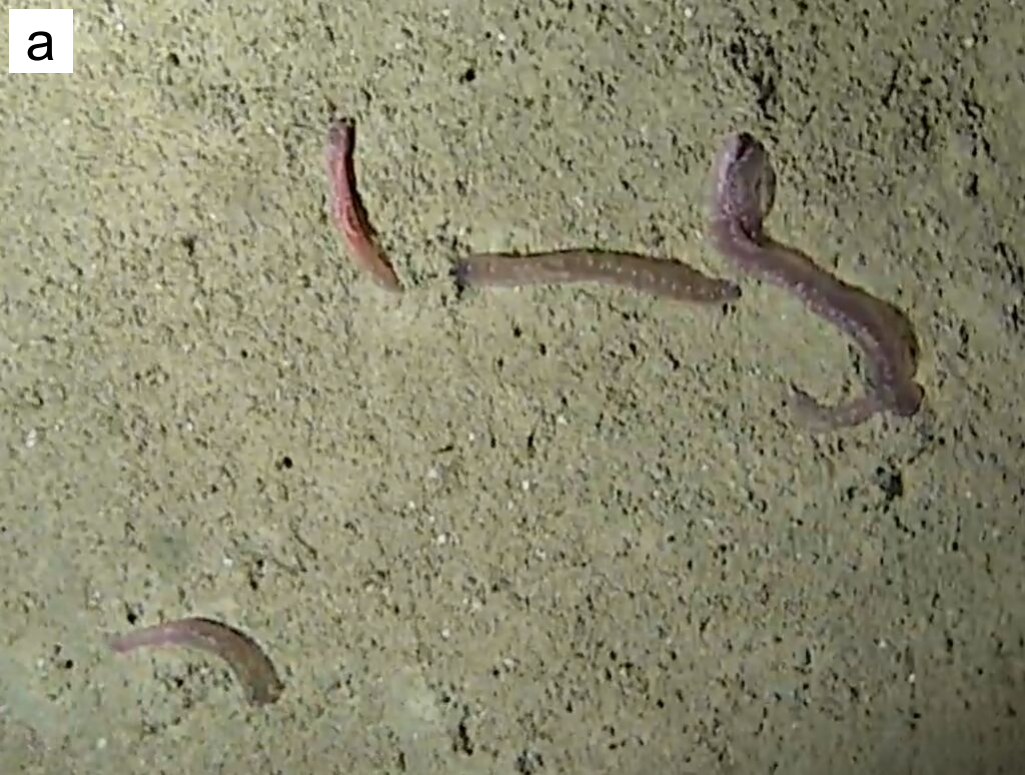
Vaavu, 490 m;

**Figure 221b.**
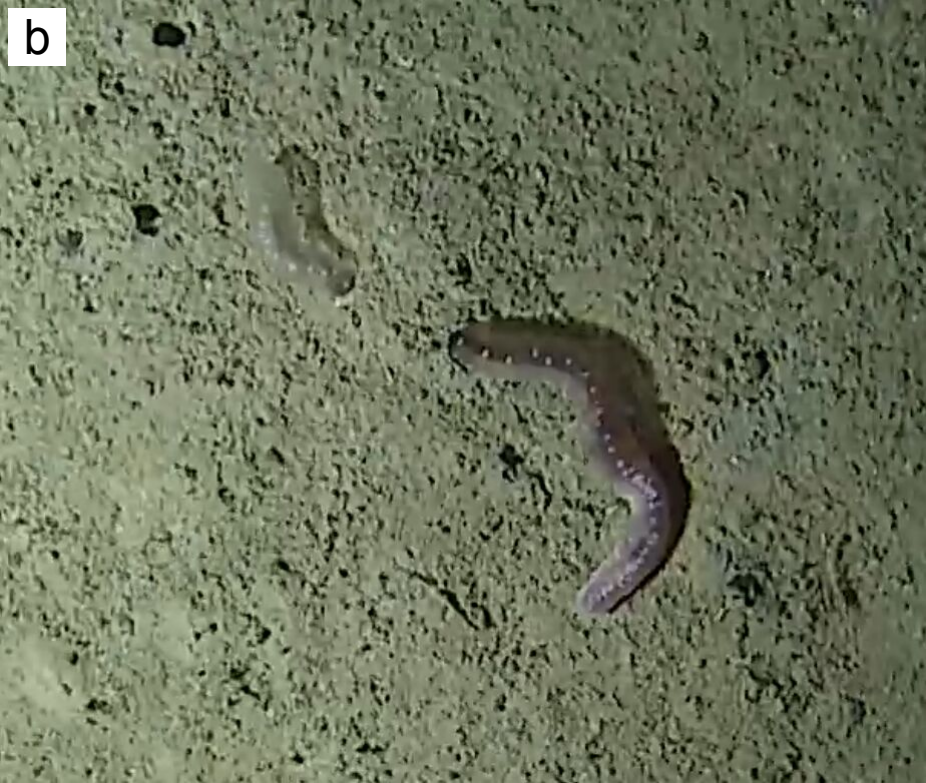
Vaavu, 490 m.

**Figure 222.**
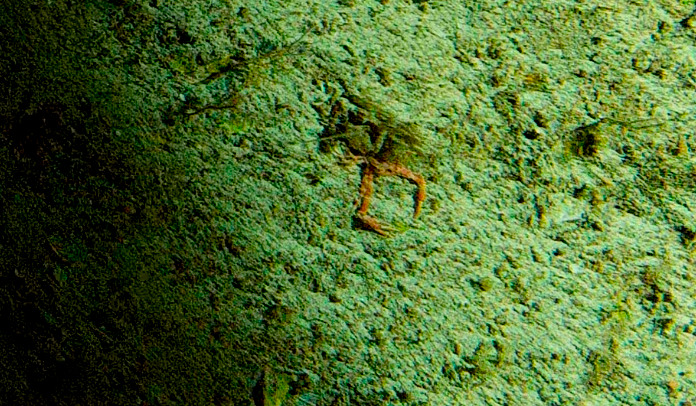
Galatheoidea gen. indet. sp., Fuvahmulah, 250 m.

**Figure 223.**
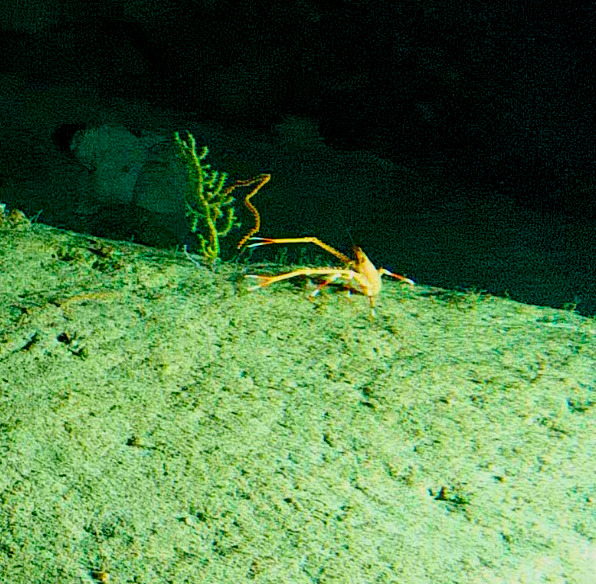
Chirostyloidea gen. indet. sp. 1, Laamu, 250 m.

**Figure 224.**
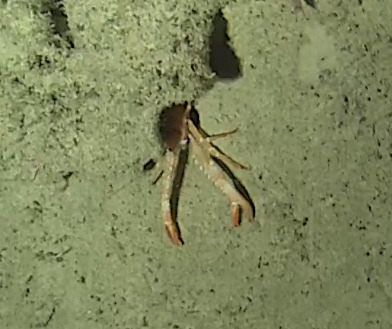
Chirostyloidea gen. indet. sp. 2, Vaavu, 250 m.

**Figure 225.**
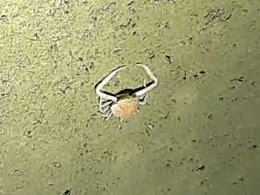
Leucosiidae gen. indet. sp., North Male’, 490 m.

**Figure 226a.**
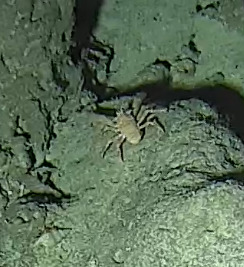
Addu, 490 m;

**Figure 226b.**
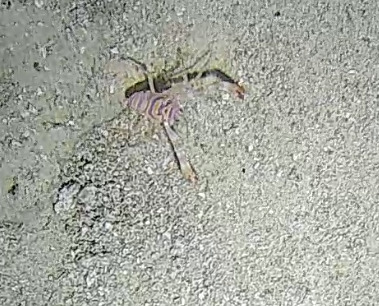
Addu, 250 m.

**Figure 227.**
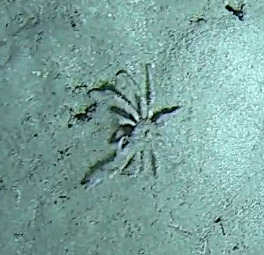
Mithracidae gen. indet. sp., North Male’, 490 m.

**Figure 228a.**
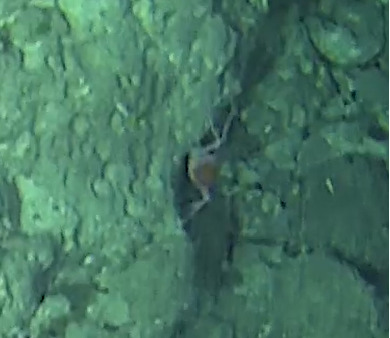
Laamu, 490 m;

**Figure 228b.**
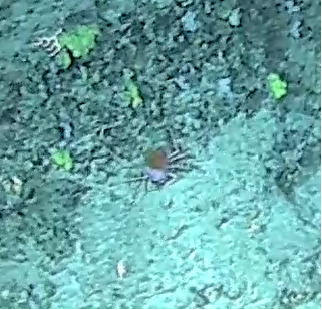
Laamu, 490 m.

**Figure 229a.**
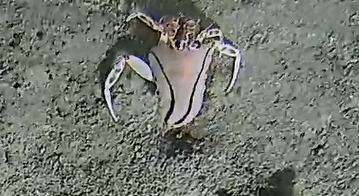
North Male’, 250 m;

**Figure 229b.**
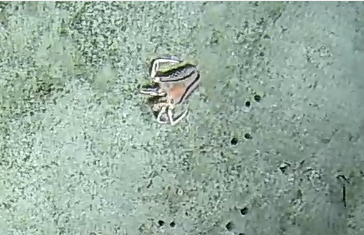
North Male’, 250 m.

**Figure 230a.**
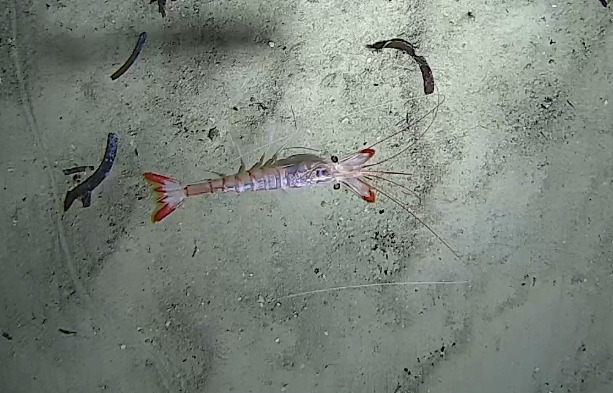
Adult, Huvadhu, 490 m;

**Figure 230b.**
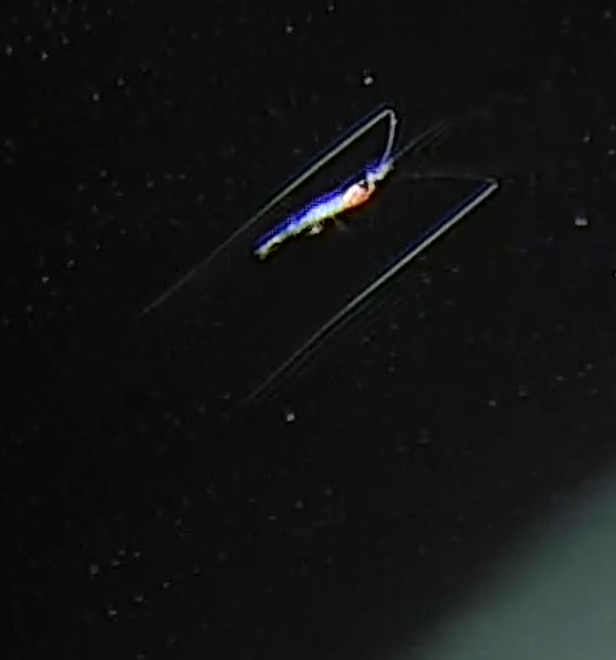
Juvenile, Vaavu, 490 m.

**Figure 231.**
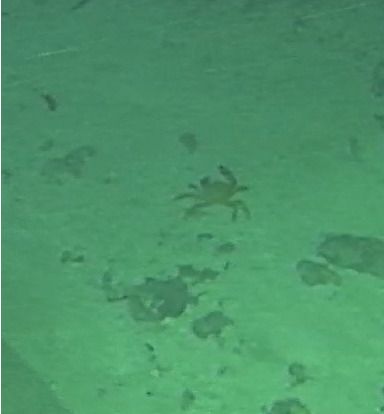
Xanthidae gen. indet. sp., Huvadhu, 490 m.

**Figure 232.**
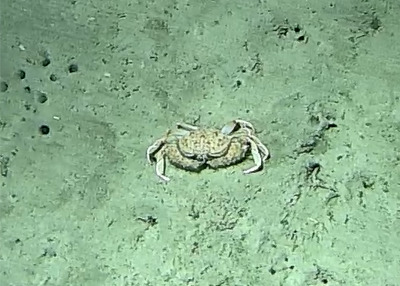
Calappidae gen. indet. sp., North Male’, 250 m.

**Figure 233.**
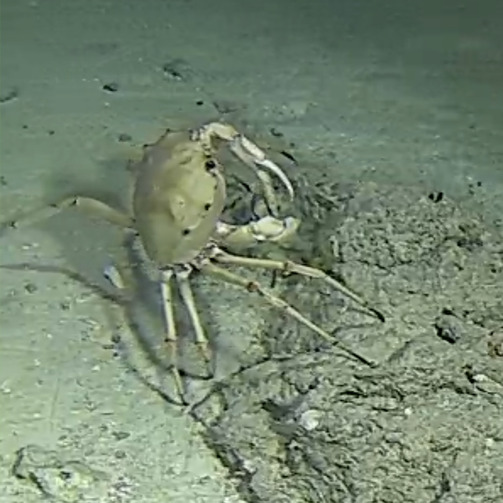
Geryonidae gen. indet. sp., Huvadhu, 490 m.

**Figure 234.**
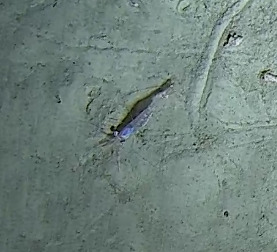
Caridea fam. indet. sp., Huvadhu, 490 m.

**Figure 235.**
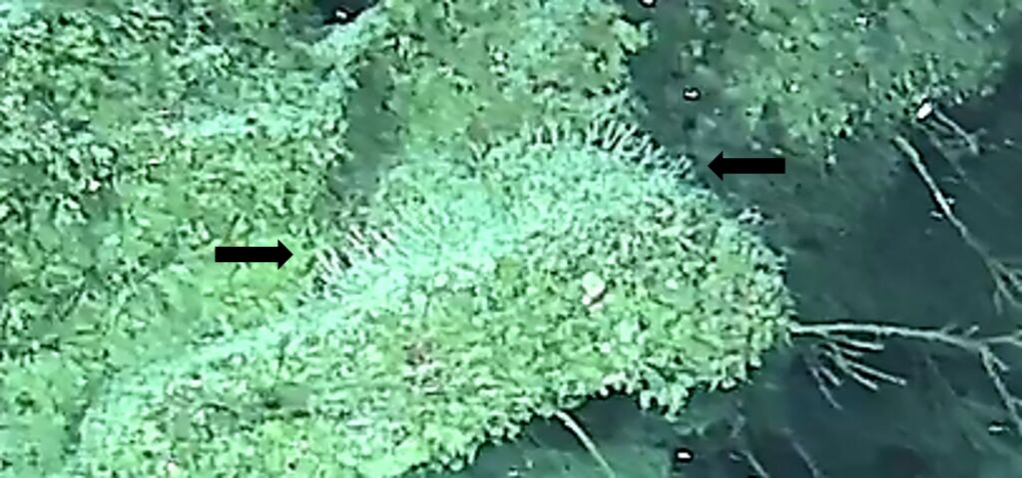
Bryozoa clas. indet. sp. 1, Vaavu, 120 m.

**Figure 236.**
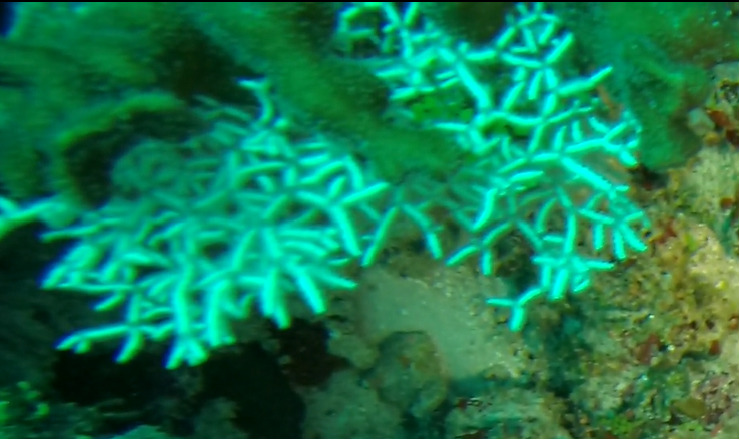
*Cellaria* sp. indet., Laamu, 30 m.

**Figure 237a.**
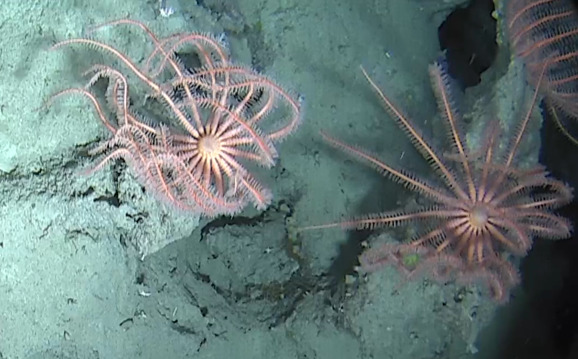
Vaavu, 250 m;

**Figure 237b.**
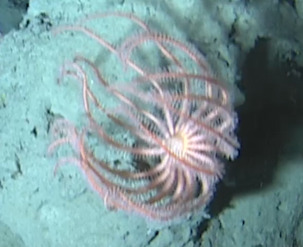
Vaavu, 250 m.

**Figure 238a.**
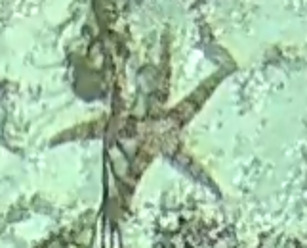
Laamu, 60 m;

**Figure 238b.**
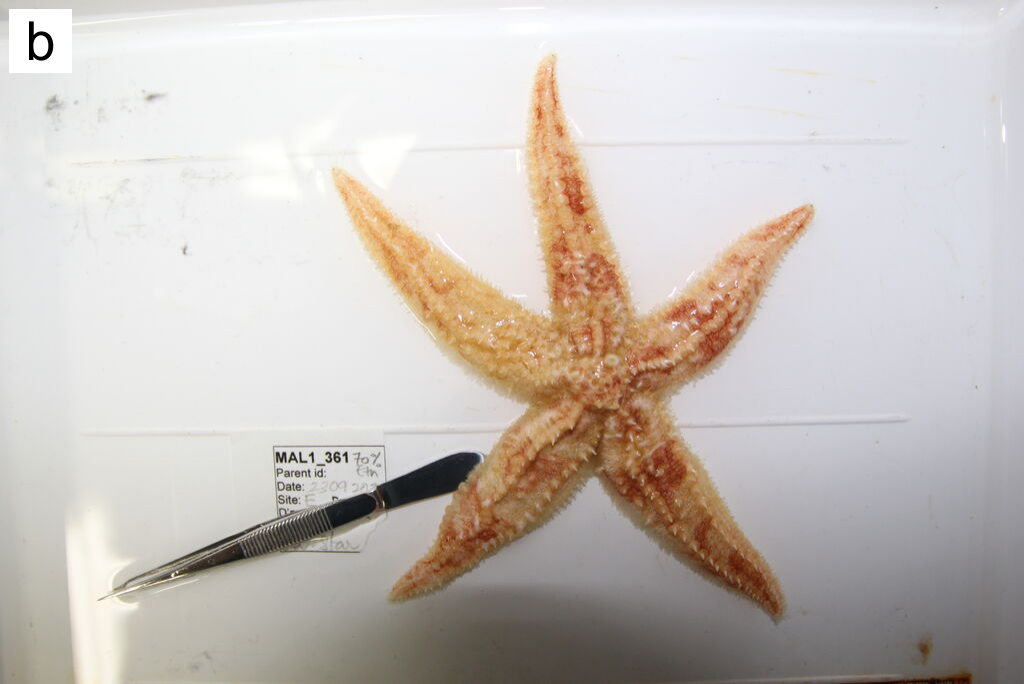
Fuvahmulah, 488 m, collected specimen MAL1_361.

**Figure 239a.**
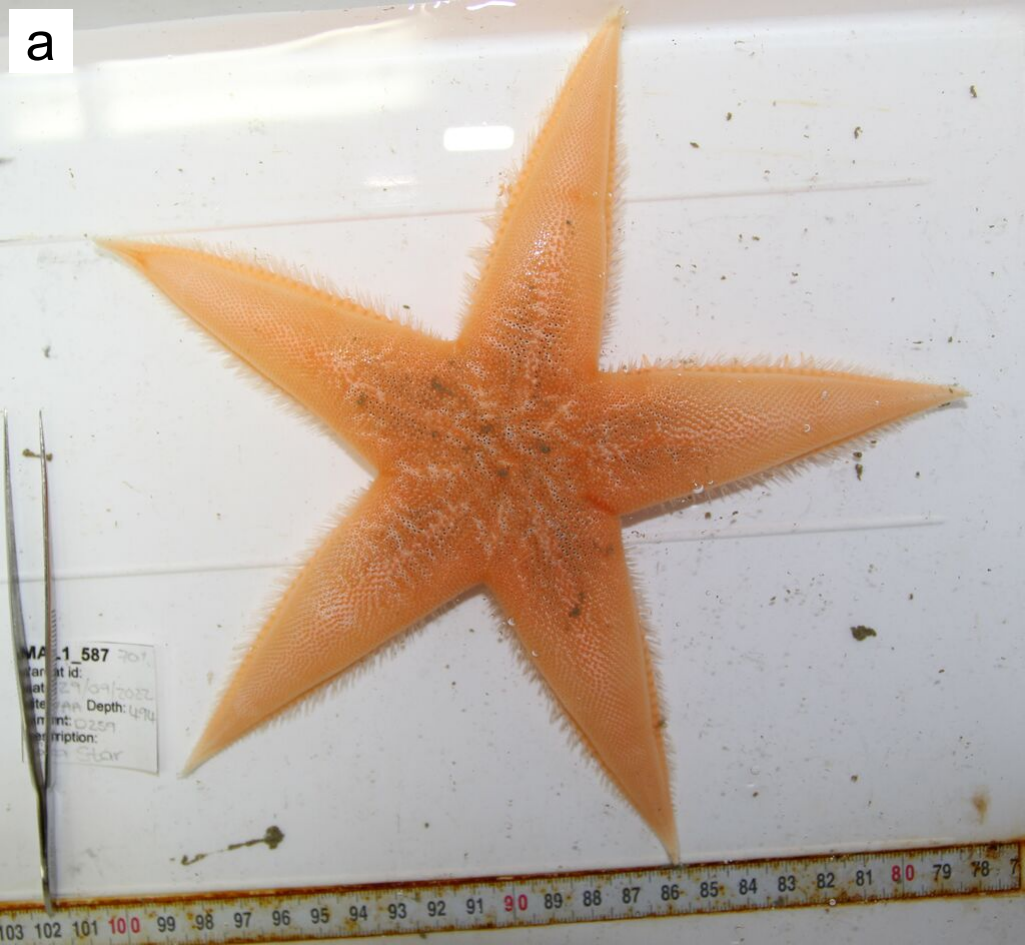
Vaavu, 494 m, collected specimen MAL1_587;

**Figure 239b.**
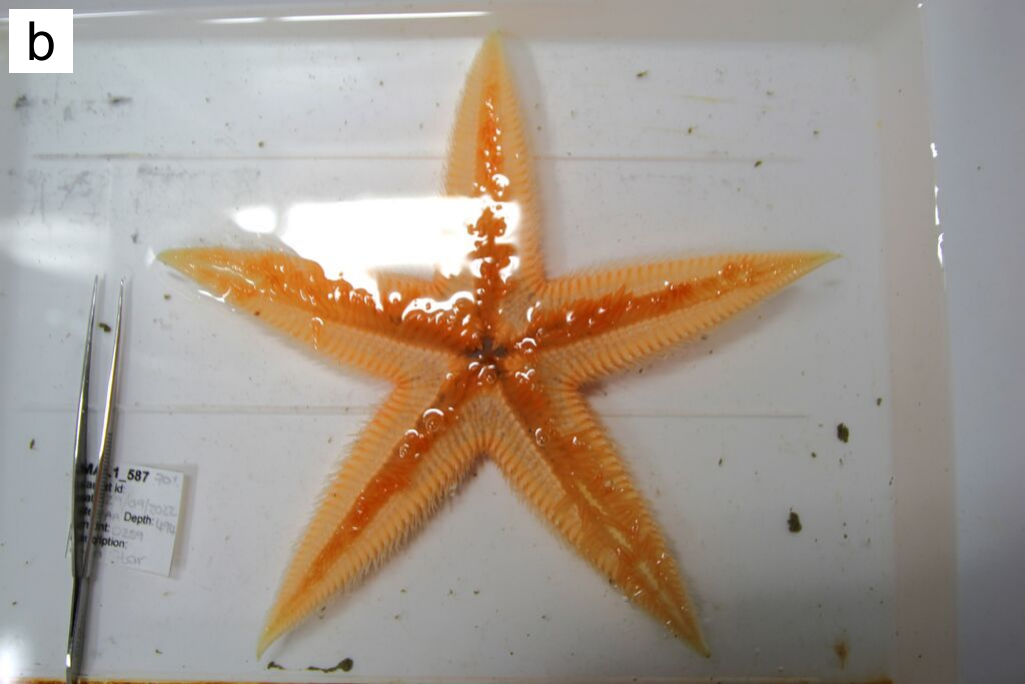
Vaavu, 494 m, collected specimen MAL1_587;

**Figure 239c.**
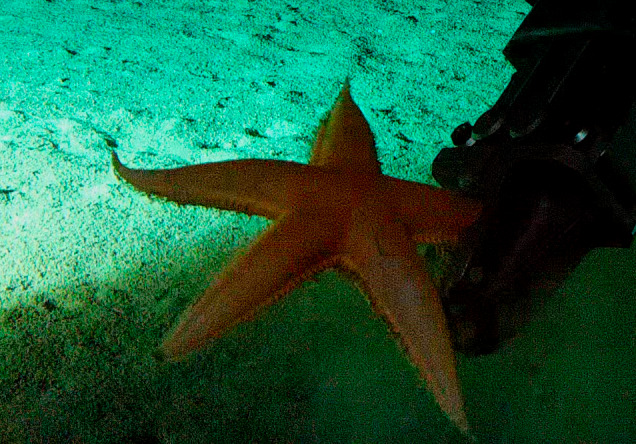
Vaavu, 494 m, in situ photo of collected specimen MAL1_587.

**Figure 240.**
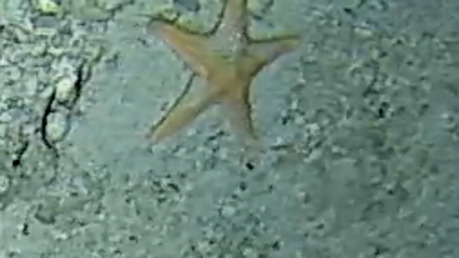
Astropectinidae gen. indet. sp. 1, Addu, 250 m.

**Figure 241a.**
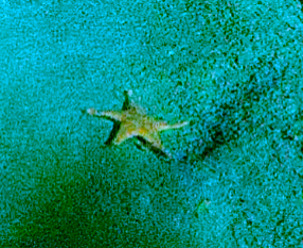
Vaavu, 248 m, *in situ* of collected specimen MAL1_619;

**Figure 241b.**
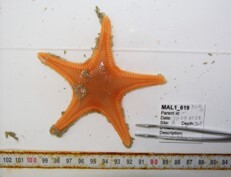
Vaavu, 248 m, collected specimen MAL1_619.

**Figure 243.**
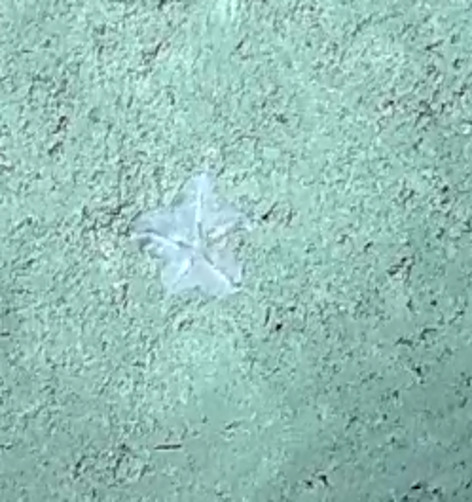
Anseropoda sp. indet., North Male’, 490 m.

**Figure 244.**
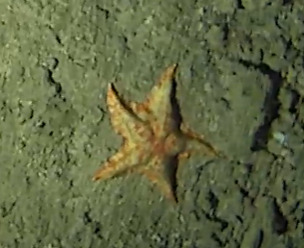
*Paranepanthia* sp. indet., Vaavu, 250 m.

**Figure 245a.**
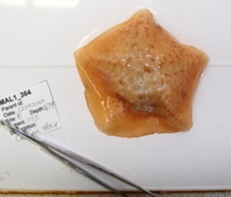
Fuvahmulah, 490 m, collected specimen MAL1_364;

**Figure 245b.**
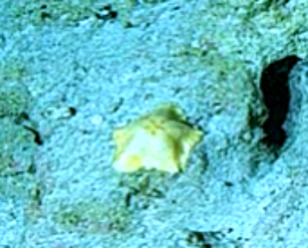
Fuvahmulah, 490 m.

**Figure 246.**
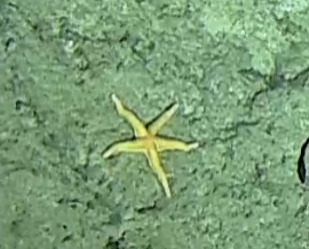
Asterinidae gen. indet. sp., Laamu, 250 m.

**Figure 247a.**
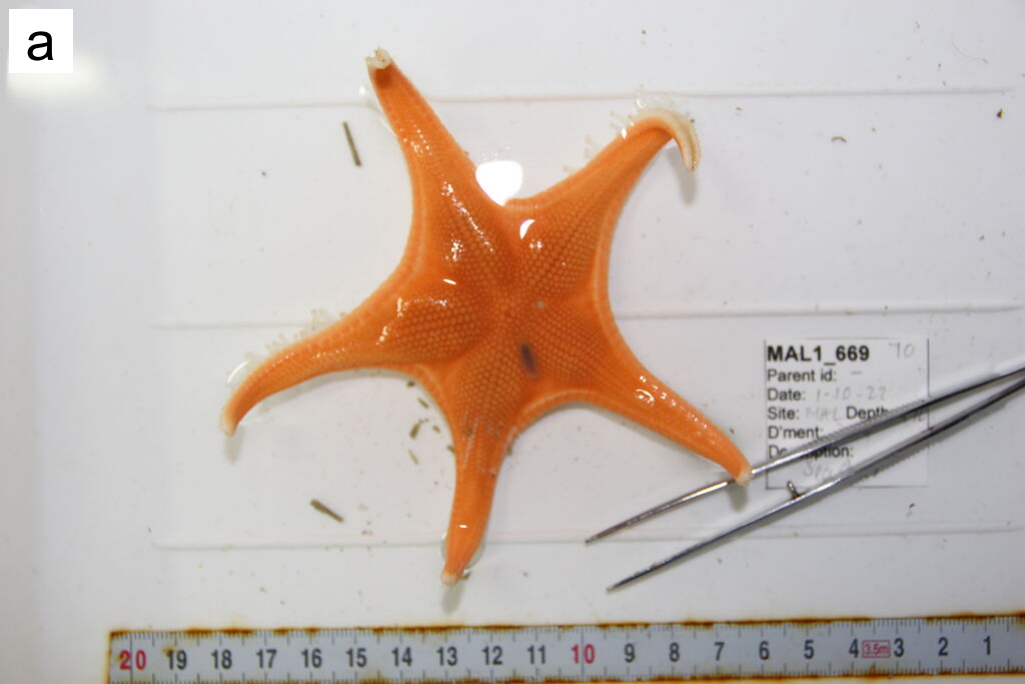
North Male', 490 m, collected specimen MAL1_669;

**Figure 247b.**
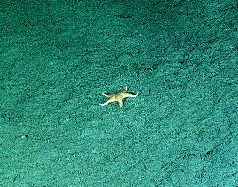
North Male', 490 m, in situ photo of collected specimen MAL1_669;

**Figure 247c.**
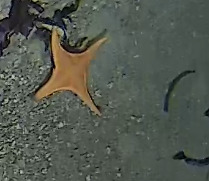
Huvadhu, 490 m;

**Figure 247d.**
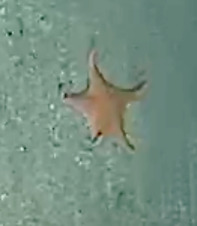
Laamu, 490 m;

**Figure 247e.**
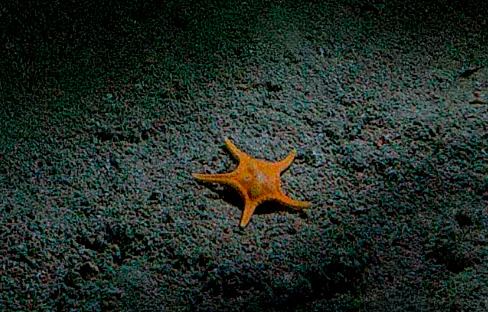
Vaavu, 242 m in situ photo of collected specimen of MAL1_621;

**Figure 247f.**
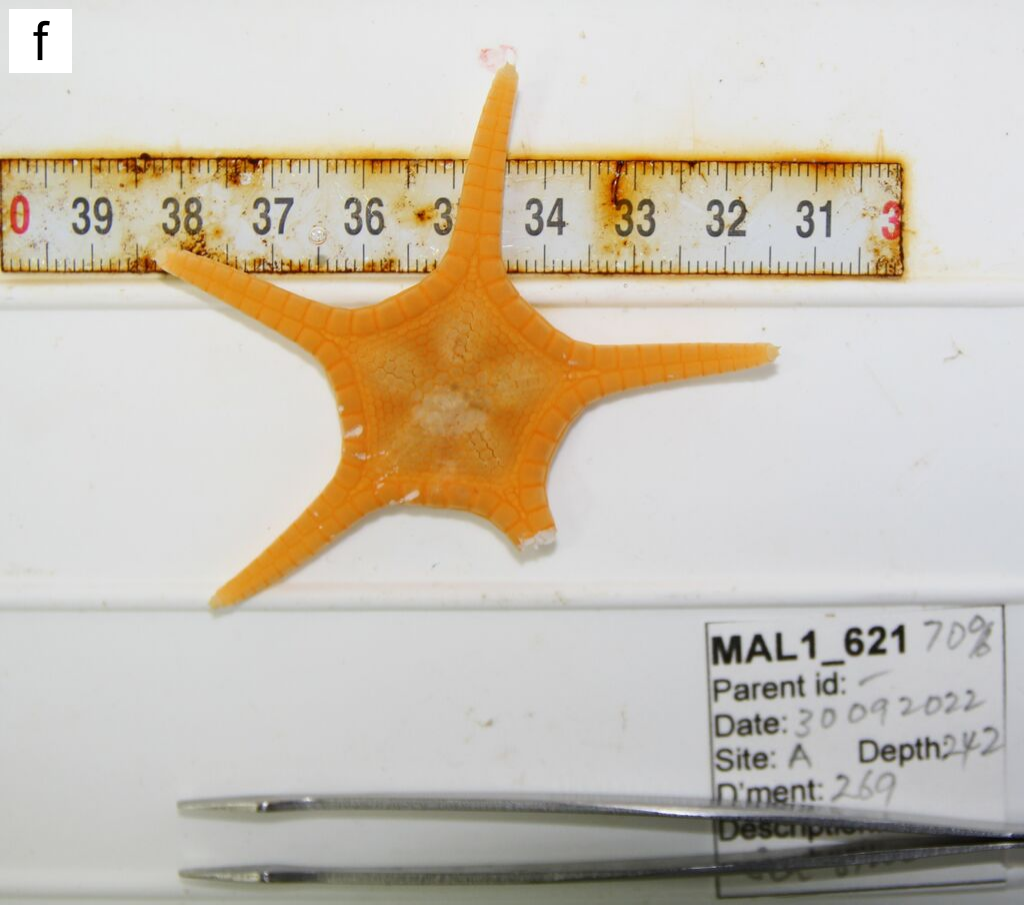
Vaavu, 242m collected specimen MAL1_621.

**Figure 248a.**
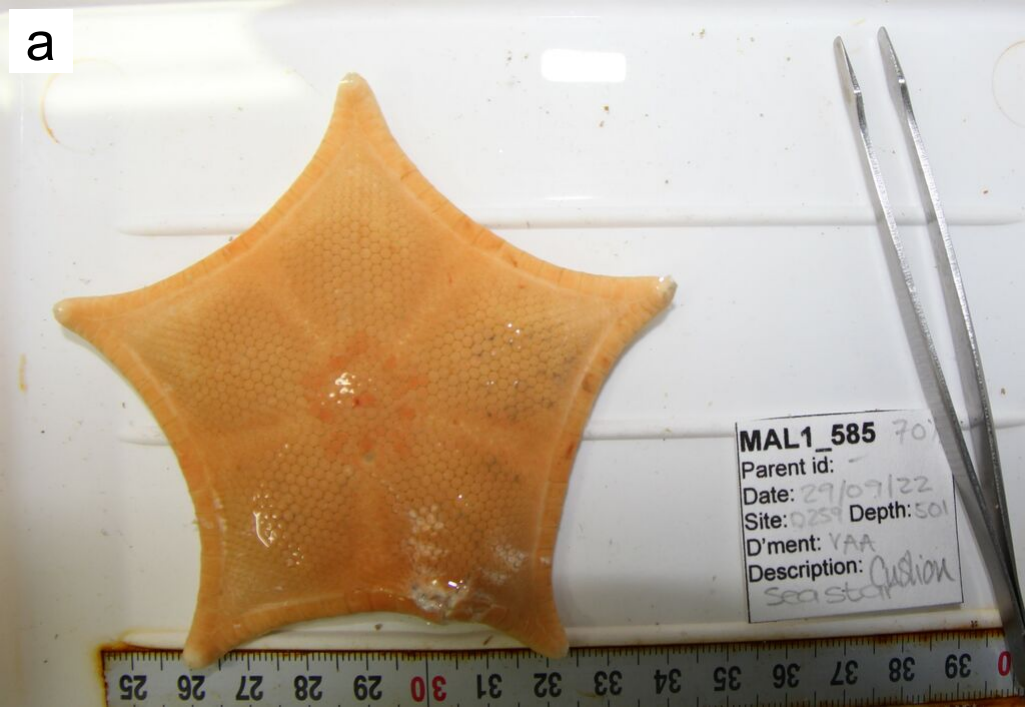
Vaavu, 501 m, collected specimen MAL1_585;

**Figure 248b.**
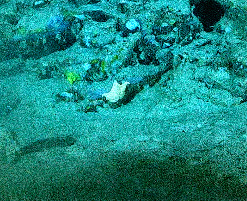
Vaavu, 501 m, in situ photo of collected specimen MAL1_585;

**Figure 248c.**
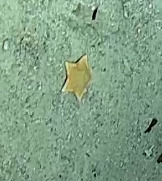
Huvadhu, 250 m;

**Figure 248d.**
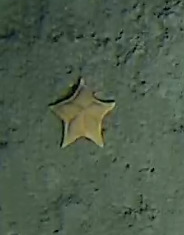
Vaavu, 250 m.

**Figure 249.**
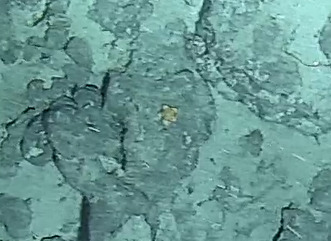
*Sphaeriodiscus* sp. indet., Addu, 250 m.

**Figure 250.**
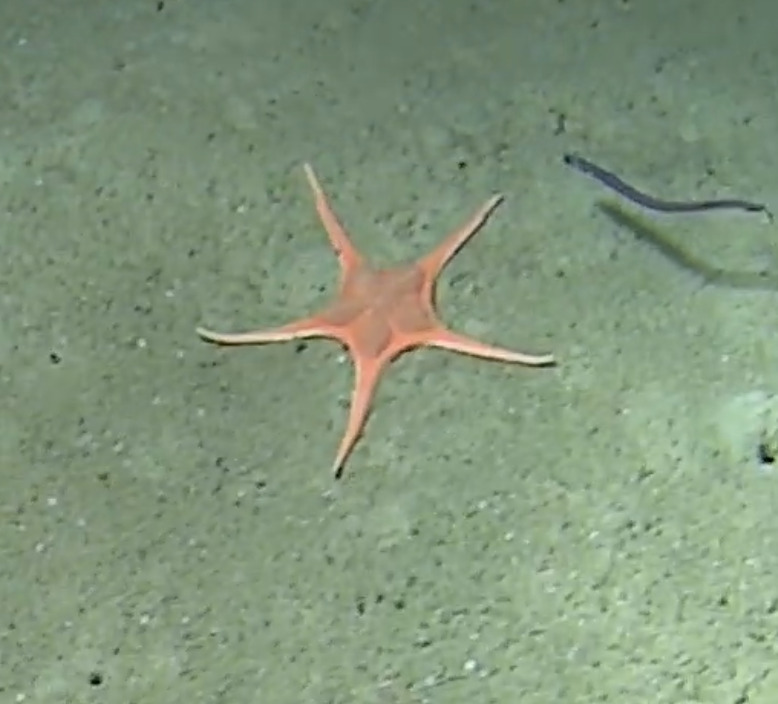
*Nymphaster* sp. indet., Vaavu, 490 m.

**Figure 251a.**
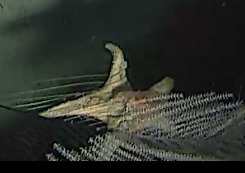
Huvadhu, 490 m;

**Figure 251b.**
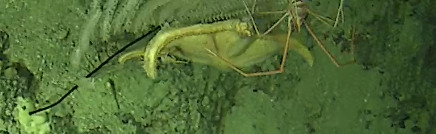
Huvadhu, 490 m.

**Figure 252a.**
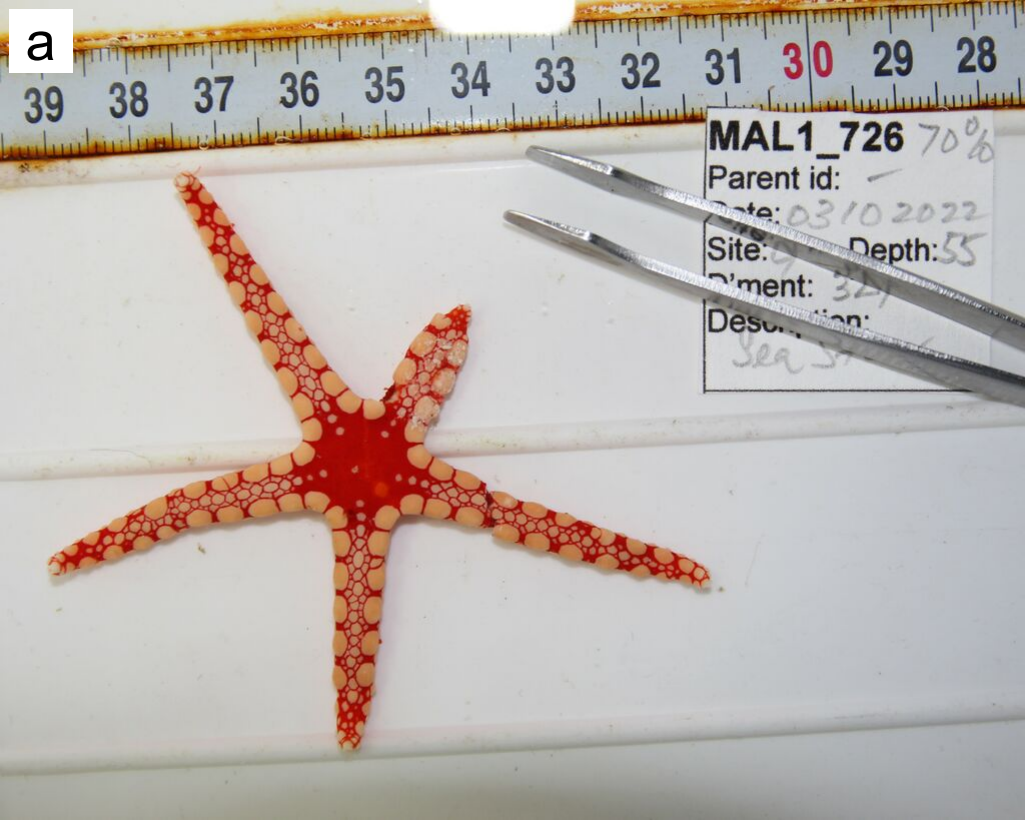
North Male’, 55 m, collected specimen MAL1_726;

**Figure 252b.**
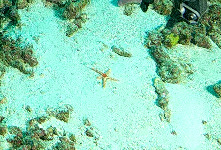
North Male’, 55 m, *in situ* photo of collected specimen MAL1_726.

**Figure 253a.**
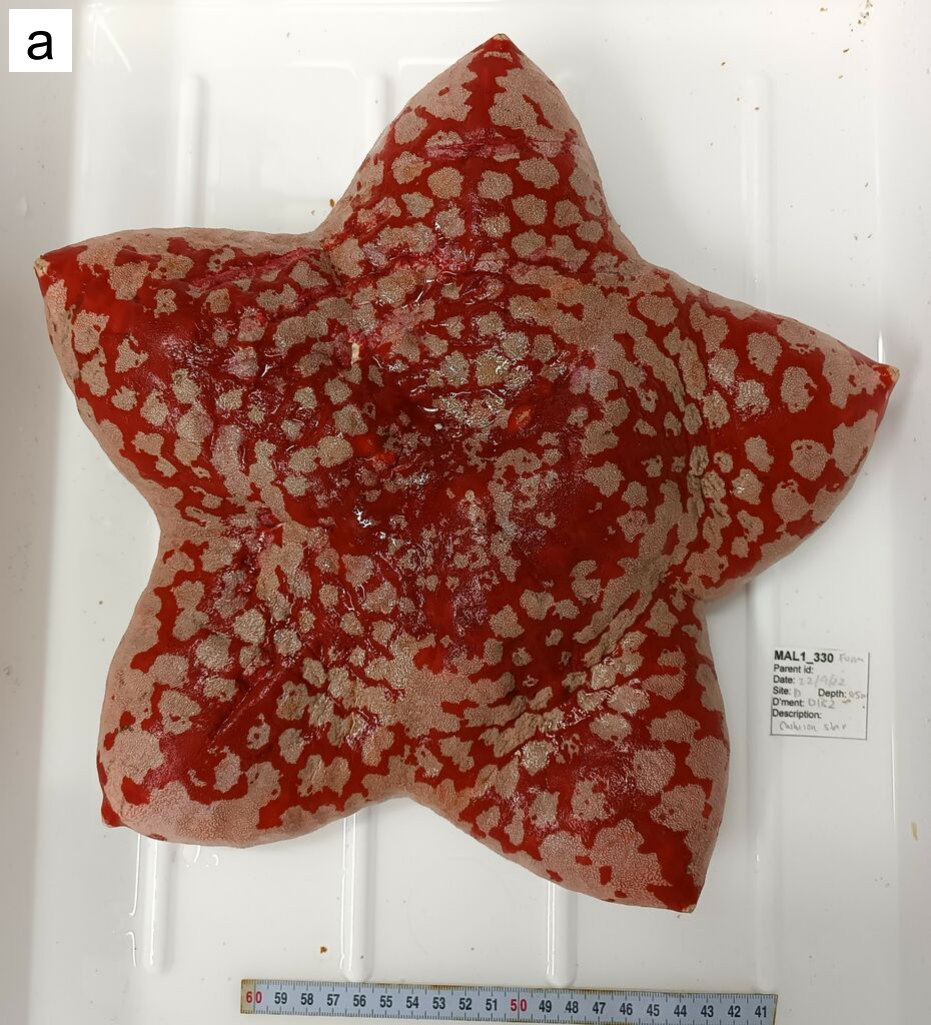
Addu, 85 m, collected specimen MAL1_334;

**Figure 253b.**
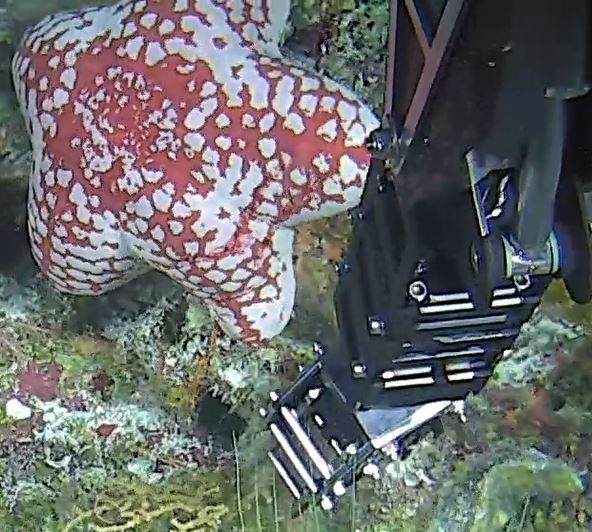
Addu, 85 m, *in situ* photo of collected specimen MAL1_334.

**Figure 254a.**
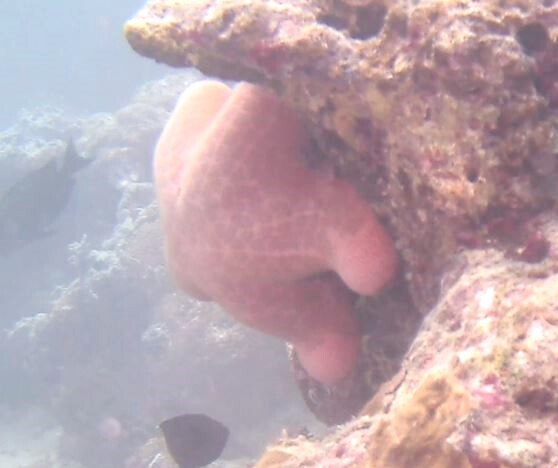
Vaavu, 10 m;

**Figure 254b.**
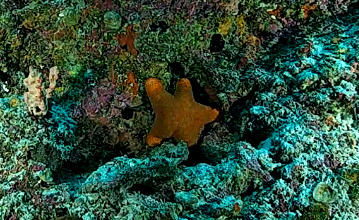
Fuvahmulah, ~ 40-60 m.

**Figure 256a.**
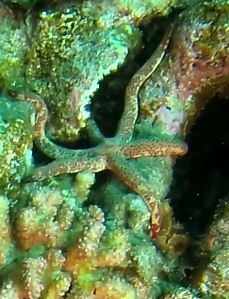
Huvadhu, 10 m;

**Figure 256b.**
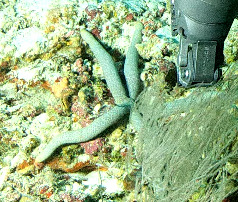
Vaavu, 62m, *in situ* photo of collected specimen MAL1_651;

**Figure 256c.**
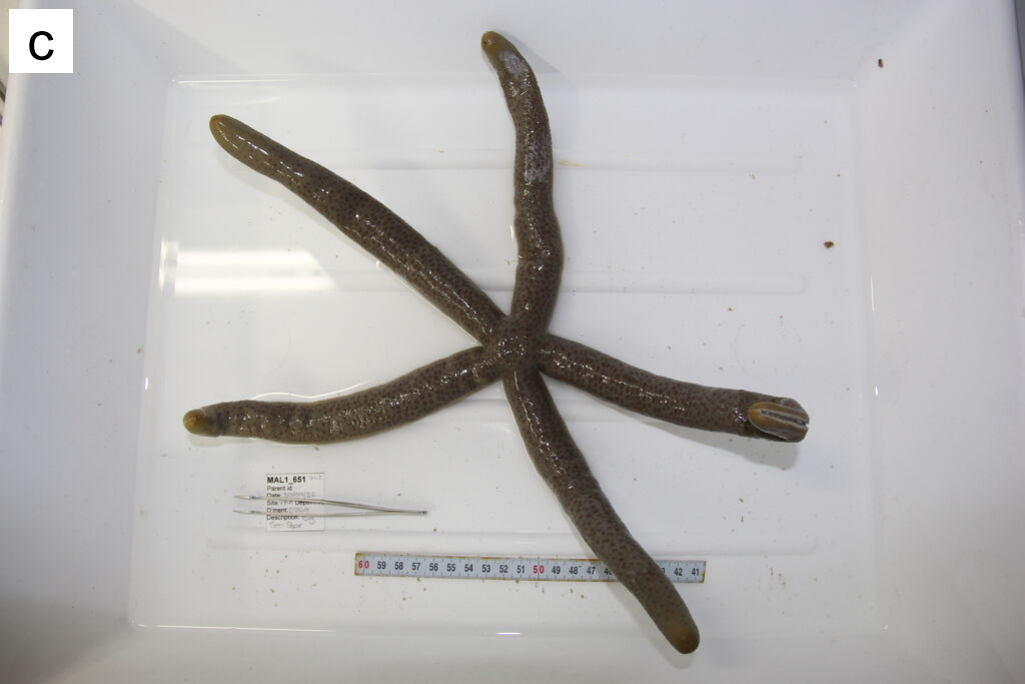
Vaavu, 62m, collected specimen MAL1_651;

**Figure 256d.**
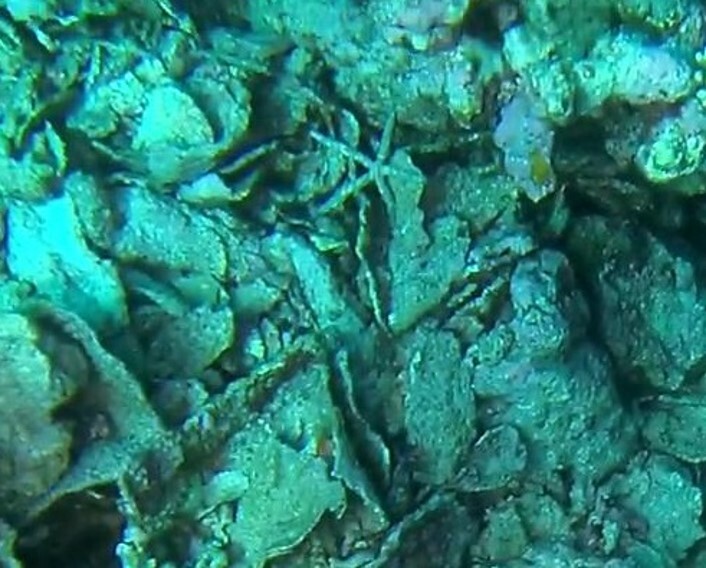
Addu, 10 m, likely *Linckiamultiflora*;

**Figure 256e.**
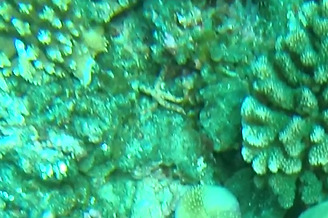
Huvadhu, 10 m, likely *L.multiflora*.

**Figure 257.**
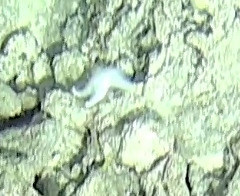
Asteroidea ord. indet. sp. 5, Addu, 490 m.

**Figure 258a.**
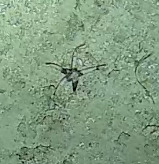
Addu, 250 m.

**Figure 259a.**
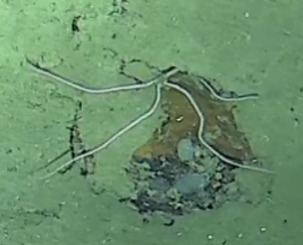
North Male’, 490 m;

**Figure 259b.**
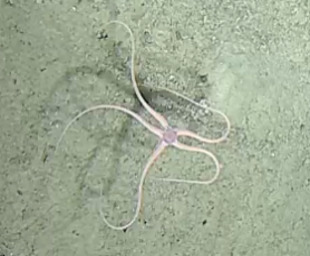
Huvadhu, 490 m;

**Figure 259c.**
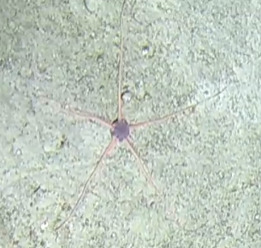
Addu, 490 m.

**Figure 260a.**
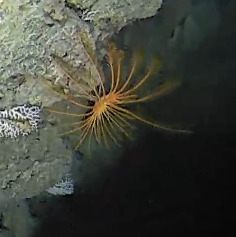
Addu, 250 m;

**Figure 260b.**
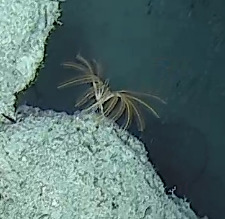
Huvadhu, 250 m.

**Figure 261.**
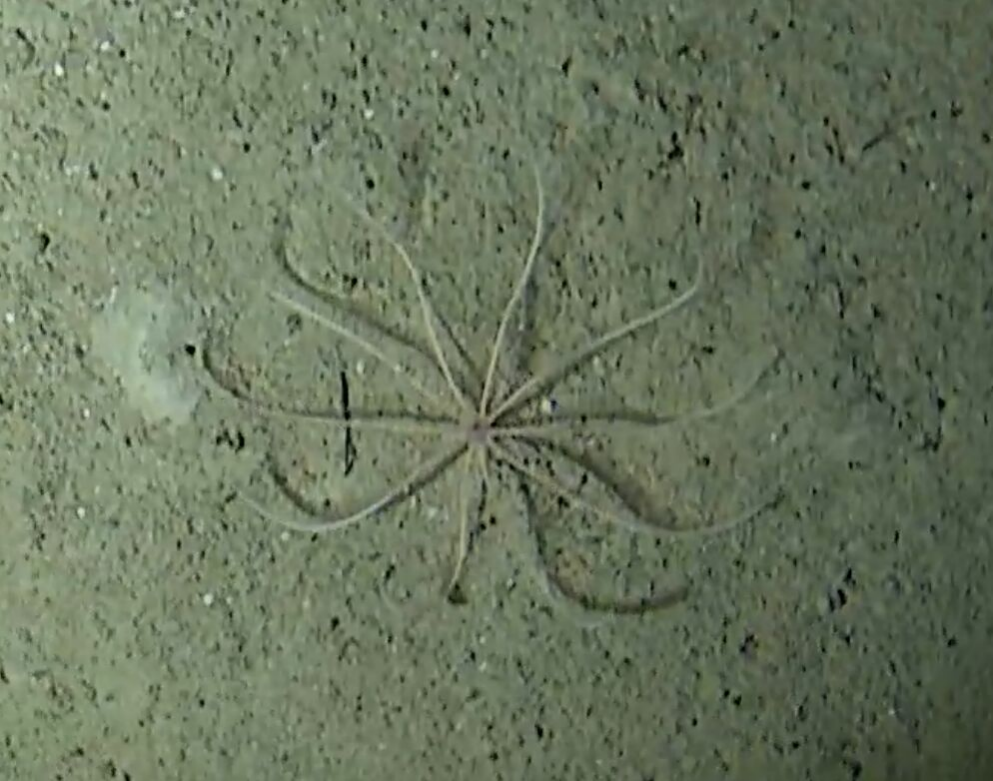
Crinoidea ord. indet. sp. 2, Vaavu, 490 m.

**Figure 262a.**
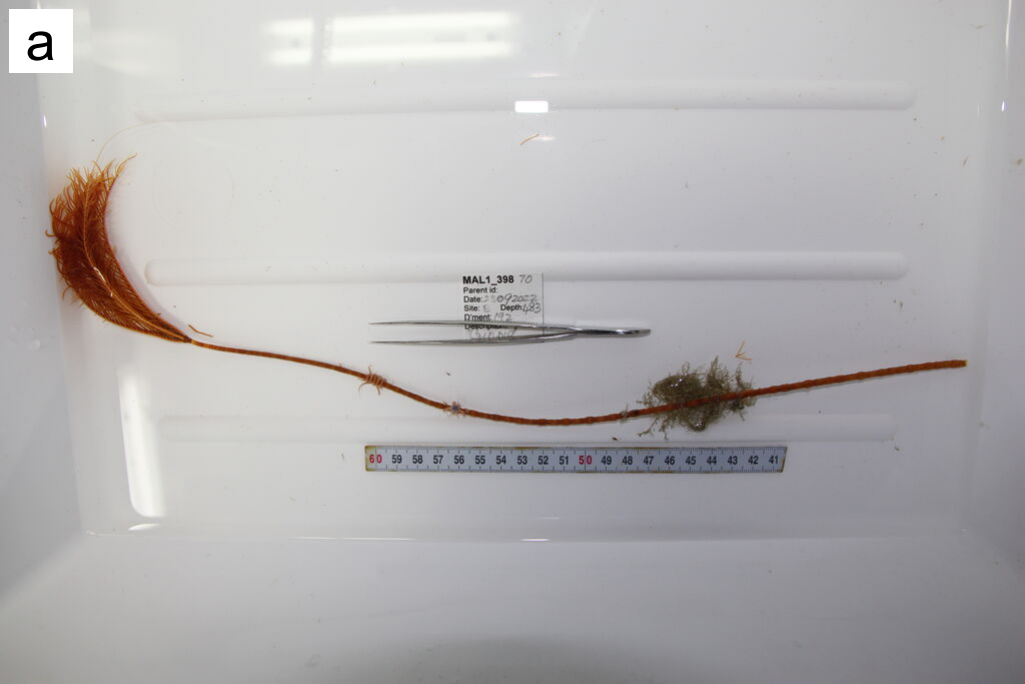
Fuvahmulah, ~ 490 m, collected specimen MAL1_398;

**Figure 262b.**
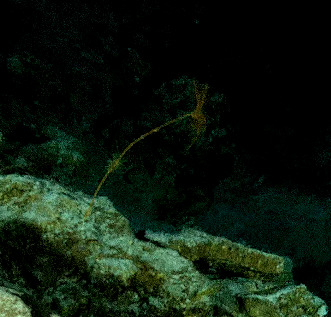
Fuvahmulah, ~ 490 m, *in situ* photo of collected specimen MAL1_398;

**Figure 262c.**
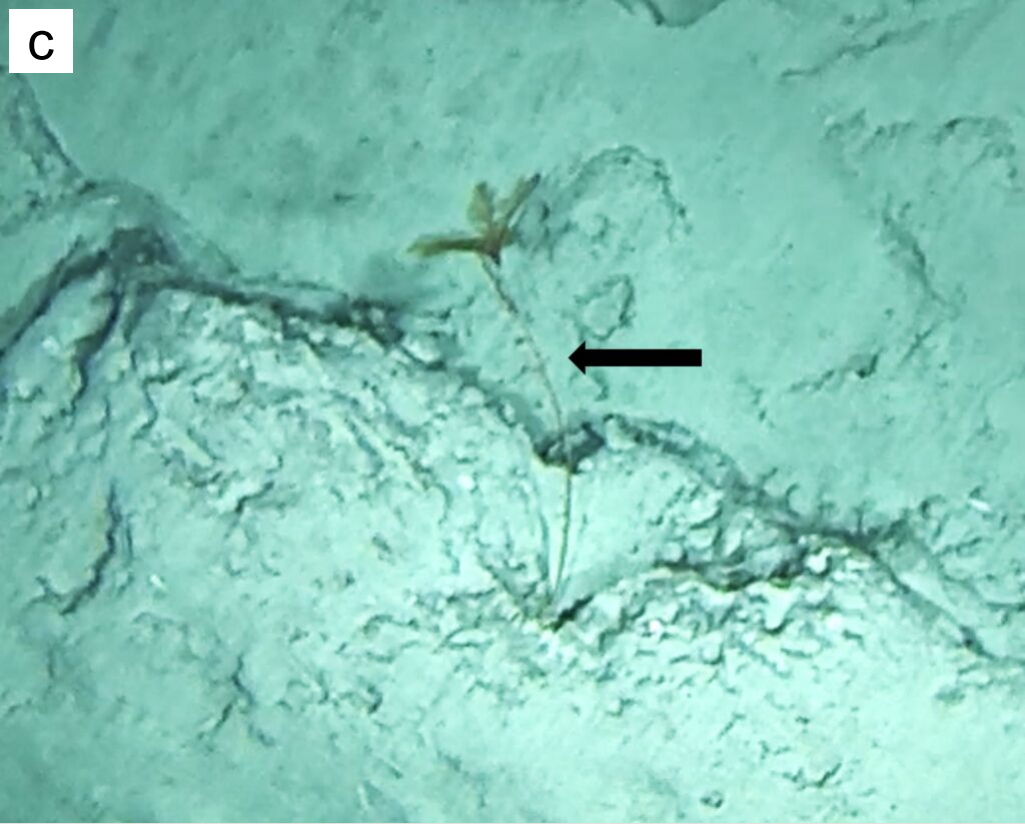
Huvadhu, ~ 490 m.

**Figure 263a.**
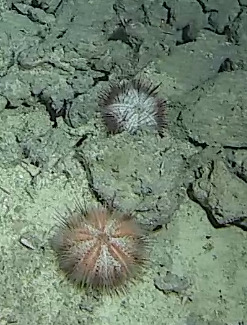
Huvadhu, 250 m;

**Figure 263b.**
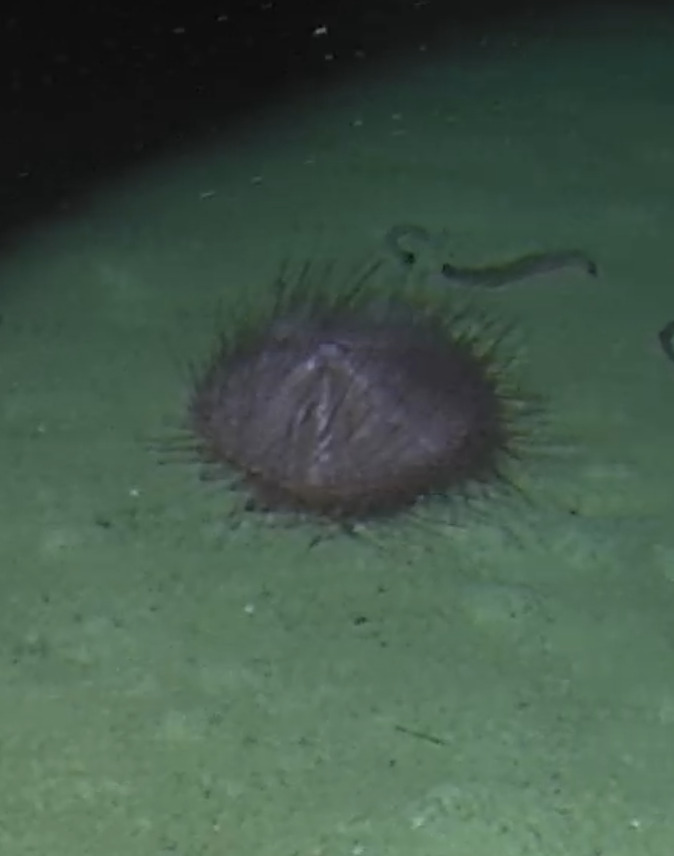
Vaavu, 490 m;

**Figure 263c.**
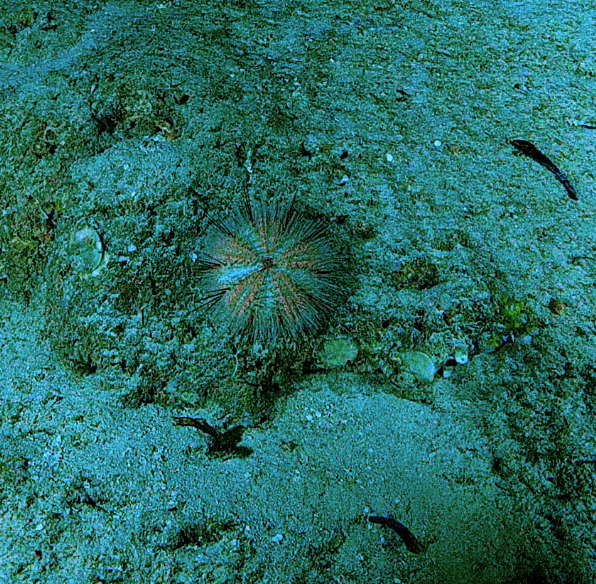
Laamu, 246 m, *in situ* photo of collected specimen MAL1_541;

**Figure 263d.**
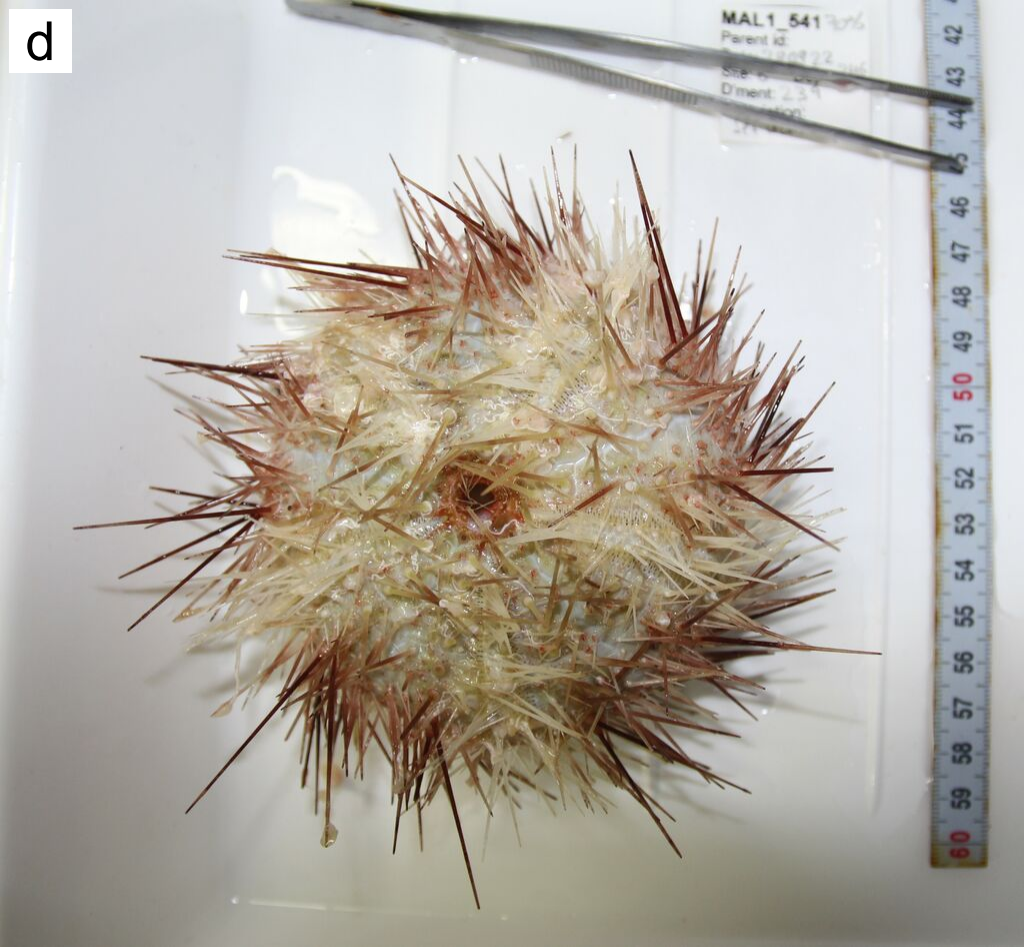
Laamu, 246 m, collected specimen MAL1_541.

**Figure 264a.**
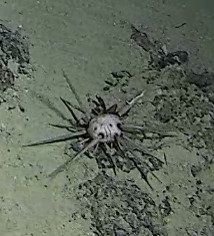
Huvadhu, 490 m;

**Figure 264b.**
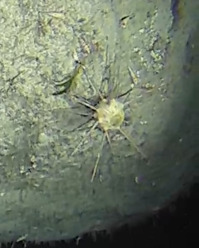
Laamu, 250 m.

**Figure 265a.**
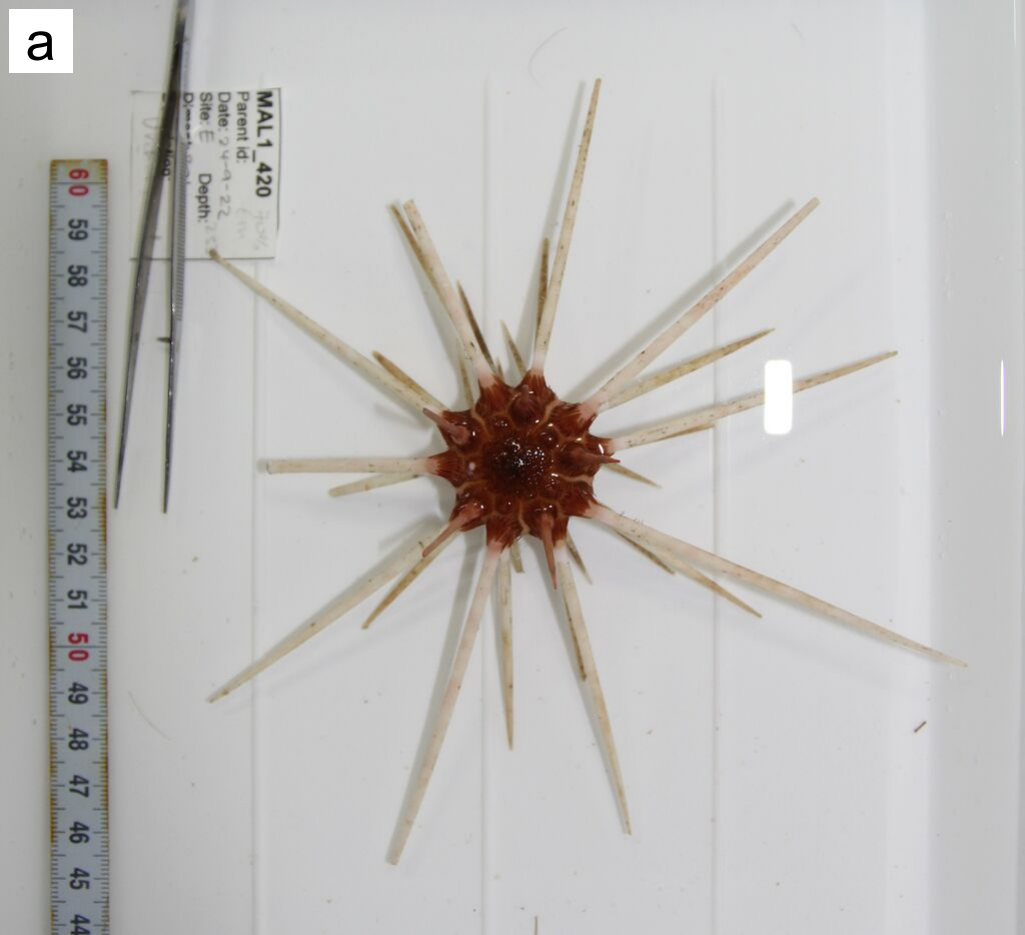
Fuvahmulah, 250 m, collected specimen MAL1_420;

**Figure 265b.**
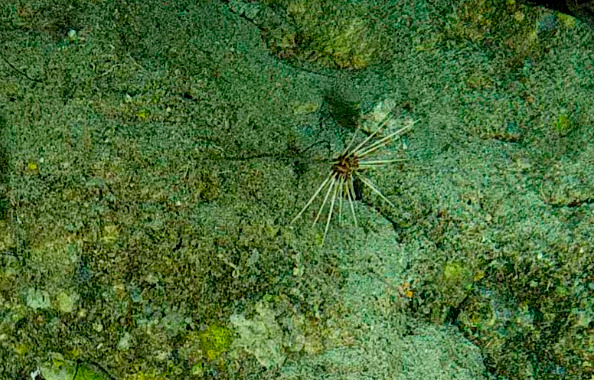
Fuvahmulah, 250 m, *in situ* photo of collected specimen MAL1_420;

**Figure 265c.**
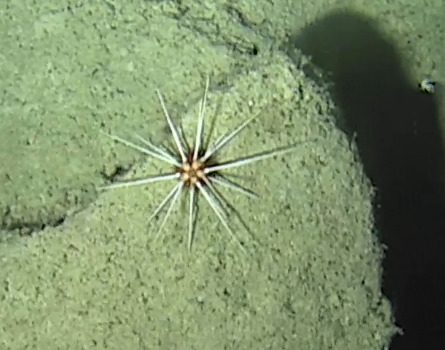
Vaavu, 250 m.

**Figure 266a.**
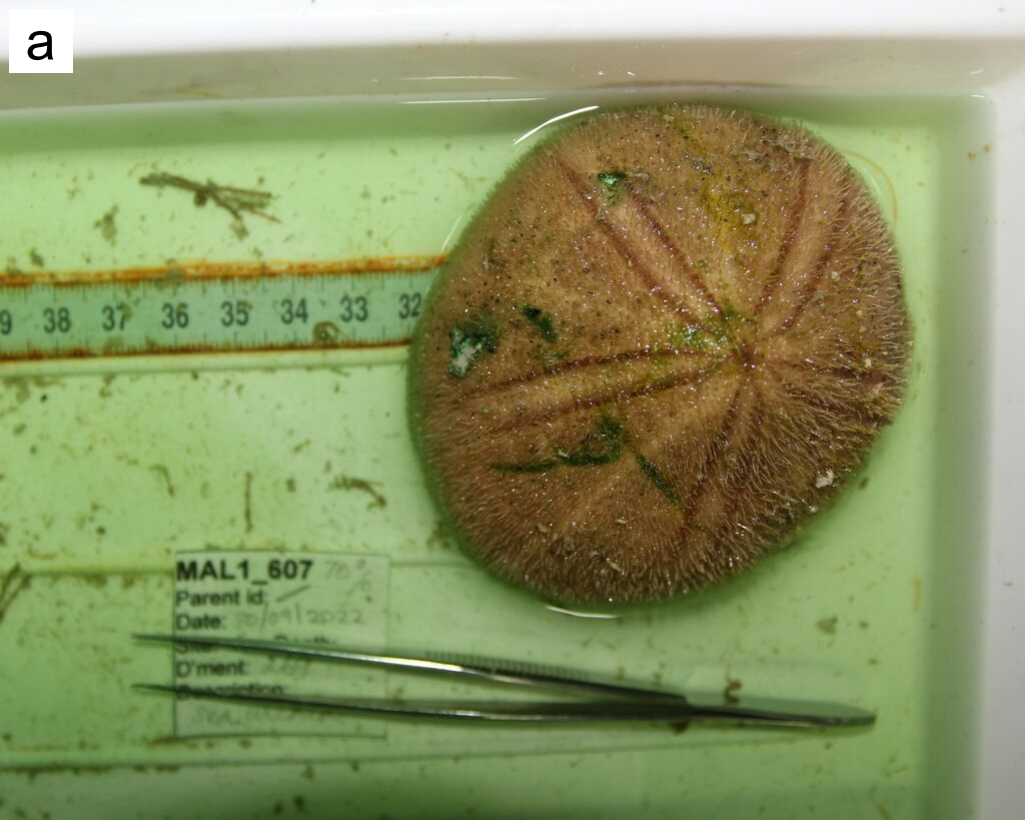
Vaavu, 246 m, collected specimen MAL1_607;

**Figure 266b.**
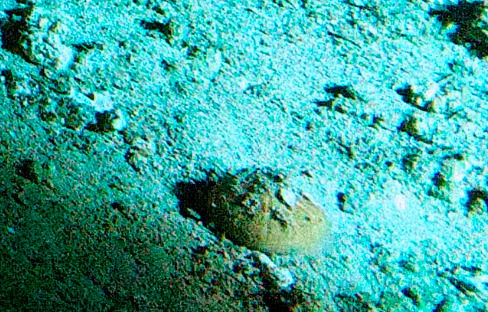
Vaavu, 246 m, *in situ* photo of collected specimen MAL1_607;

**Figure 266c.**
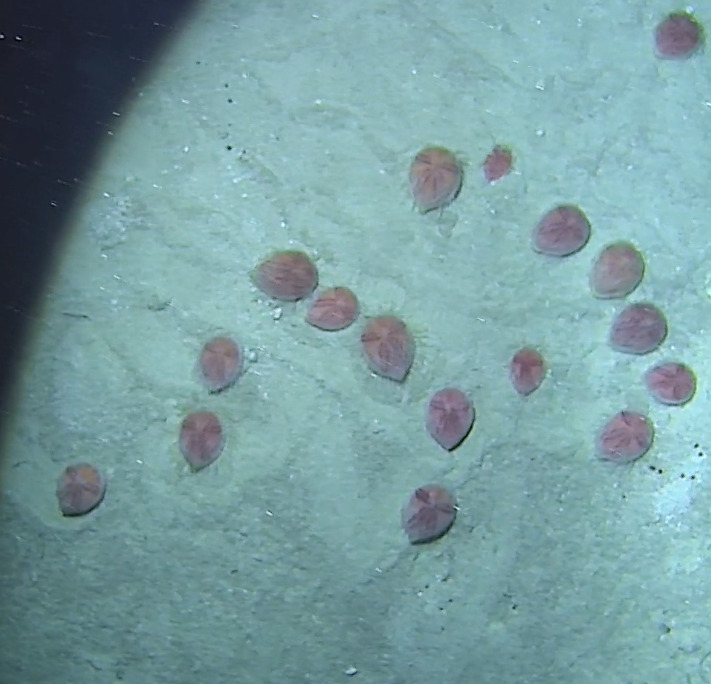
Vaavu, 250 m.

**Figure 268a.**
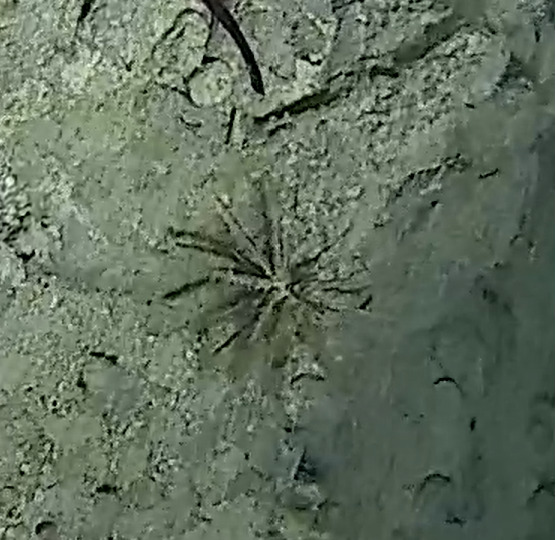
Vaavu, 120 m.

**Figure 269.**
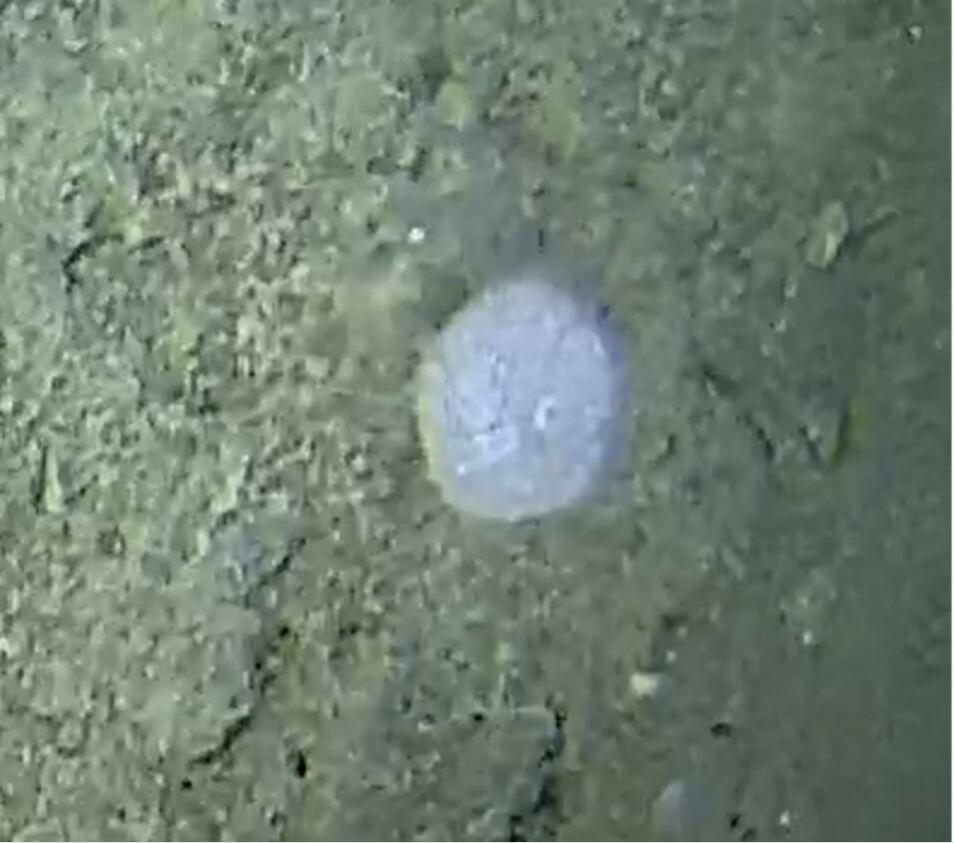
Echinoidea ord. indet. sp. 2, Laamu, 490 m.

**Figure 270a.**
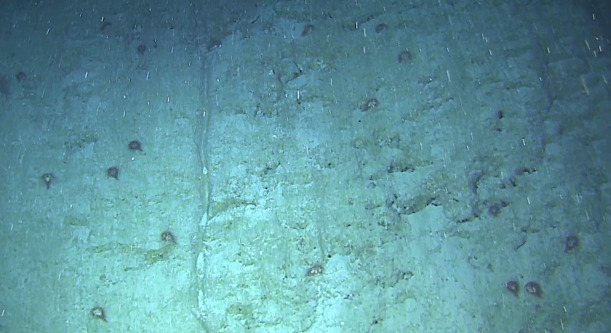
North Male’, 120 m;

**Figure 270b.**
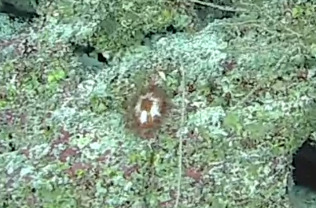
North Male’, 120 m.

**Figure 271a.**
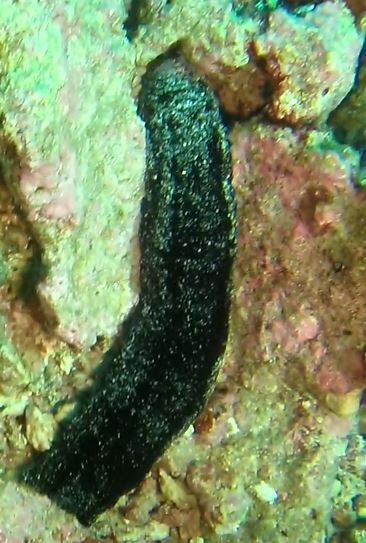
Laamu, 10 m;

**Figure 271b.**
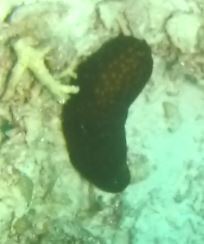
Addu, 30 m.

**Figure 274.**
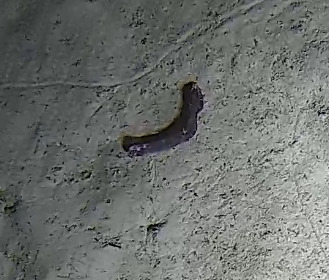
Holothuroidea ord. indet. sp. 3, Huvadhu, 490 m.

**Figure 275a.**
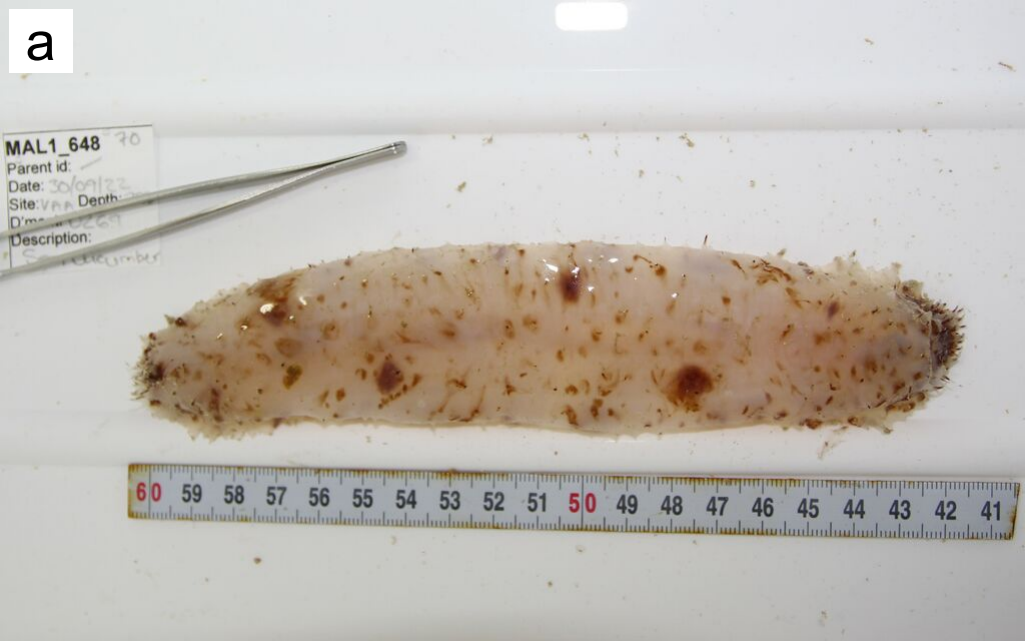
Vaavu, 233 m, collected specimen MAL1_648;

**Figure 275b.**
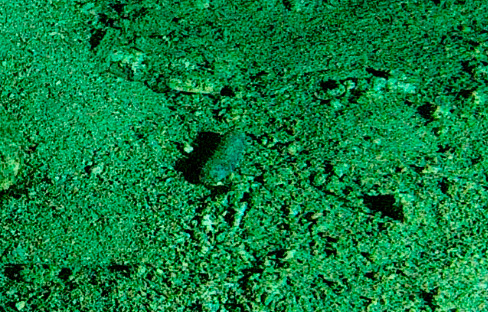
Vaavu, 233 m, *in situ* photo of collected specimen MAL1_648;

**Figure 275c.**
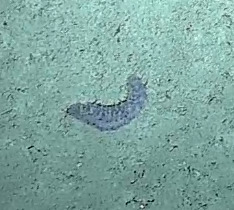
North Male’, 250 m.

**Figure 276a.**
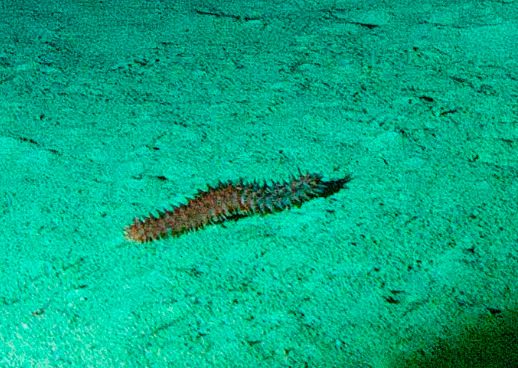
Vaavu, 496 m, *in situ* photo of collected specimen MAL1_589;

**Figure 276b.**
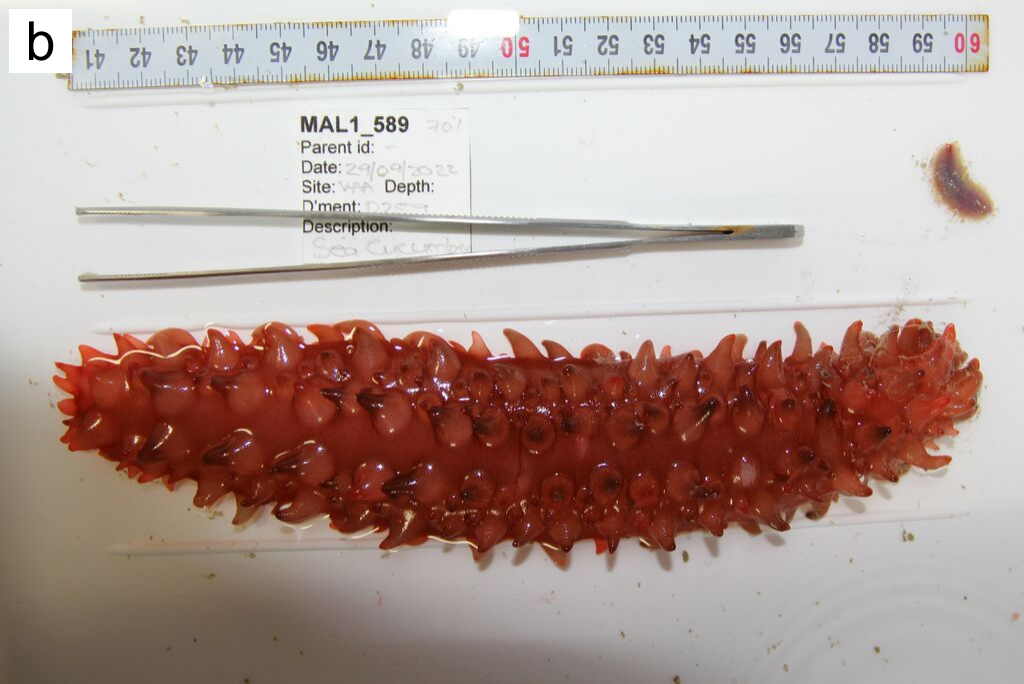
Vaavu, 496 m, collected specimen MAL1_589.

**Figure 277a.**
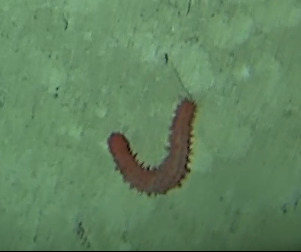
North Male’, 250 m;

**Figure 277b.**
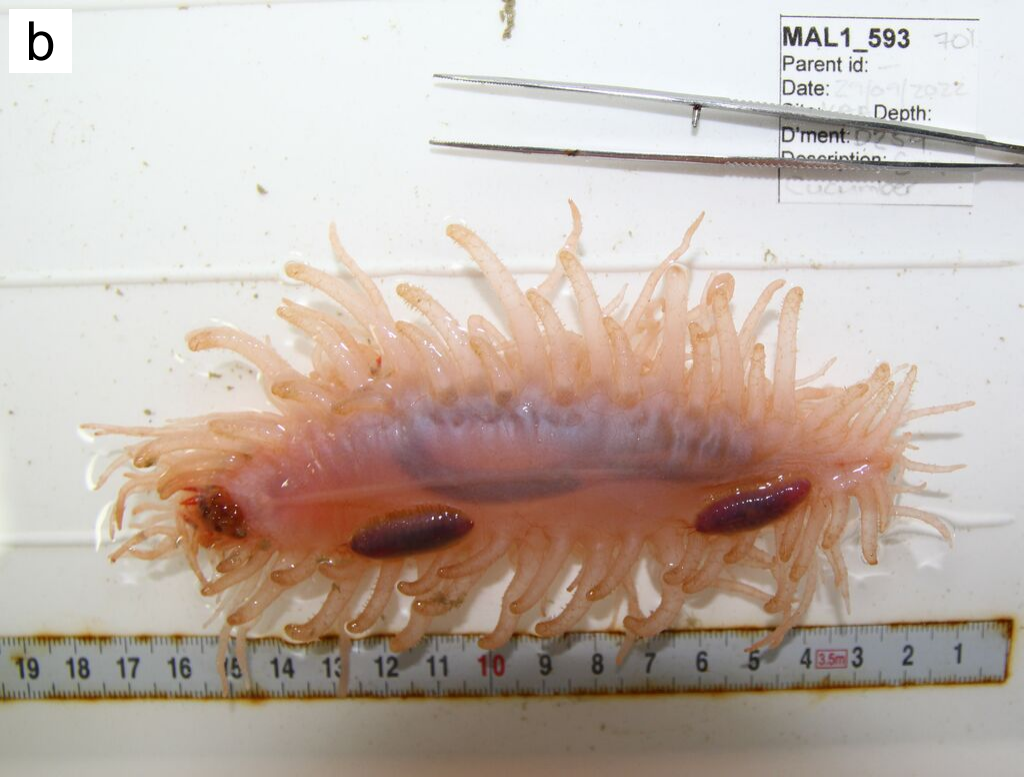
Vaavu, 481 m, collected specimen MAL1_593;

**Figure 277c.**
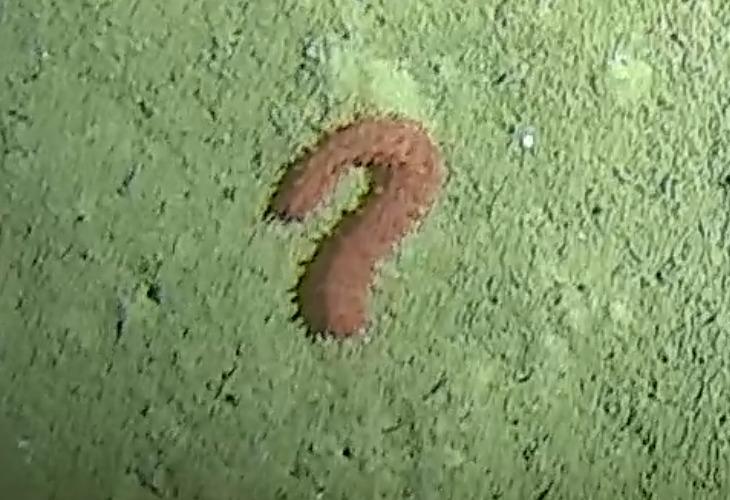
Vaavu, 490 m.

**Figure 278a.**
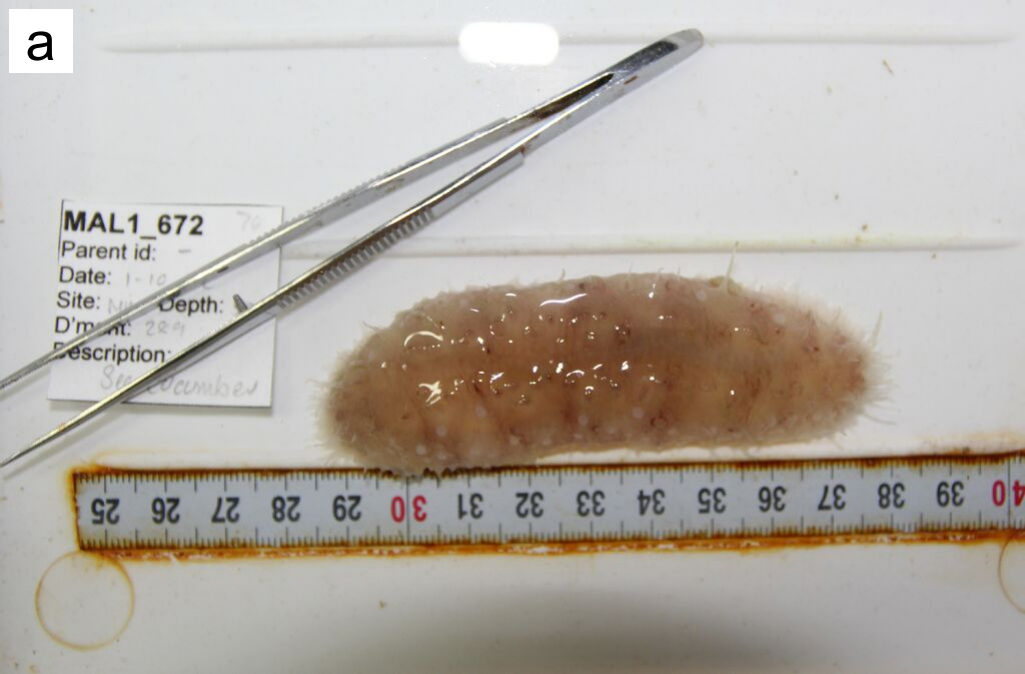
North Male’, collected specimen 490 m, MAL1_672;

**Figure 278b.**
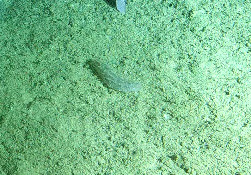
North Male’, 490 m *in situ* photo of collected specimen MAL1_672;

**Figure 278c.**
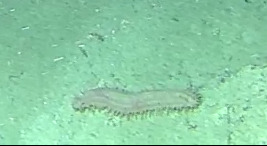
North Male’, 490 m.

**Figure 279.**
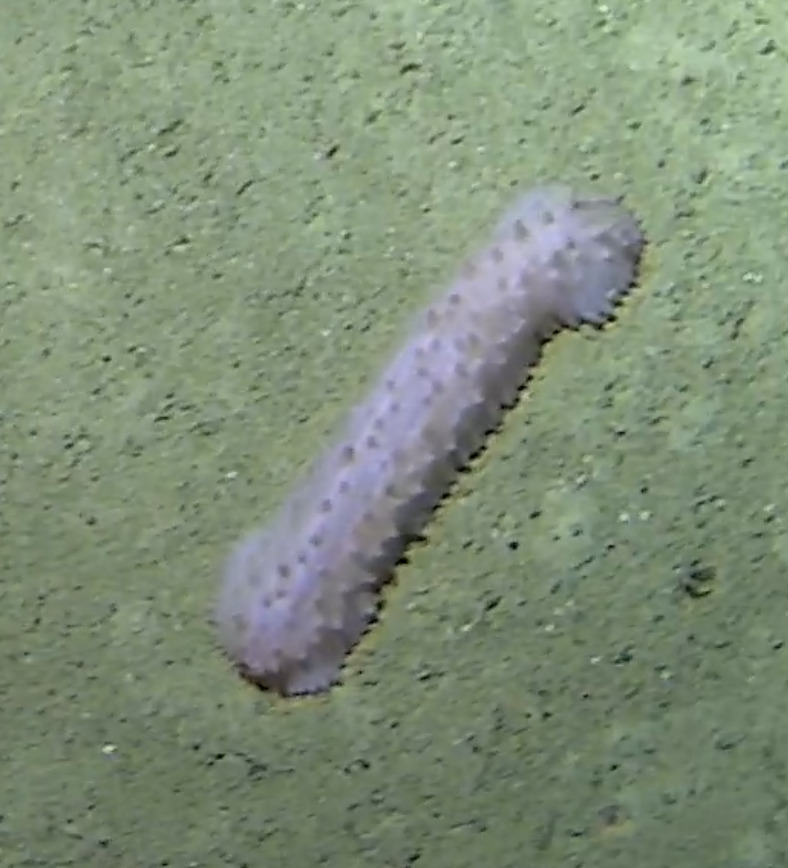
Holothuroidea ord. indet. sp. 8, Vaavu, 490 m.

**Figure 280.**
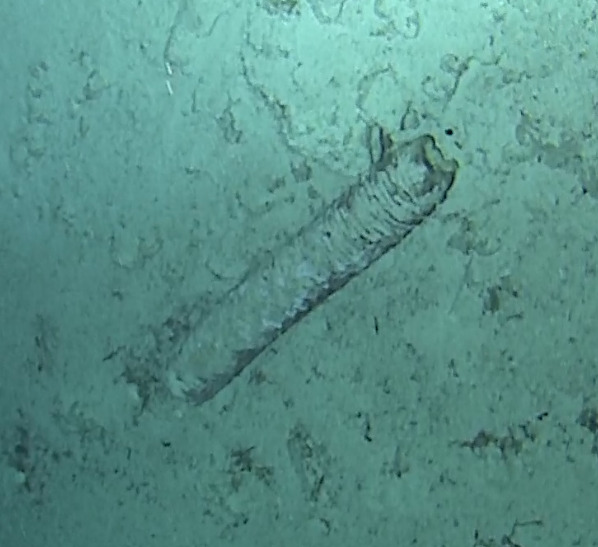
Holothuroidea ord. indet. sp. 9, Fuvahmulah, 490 m.

**Figure 281a.**
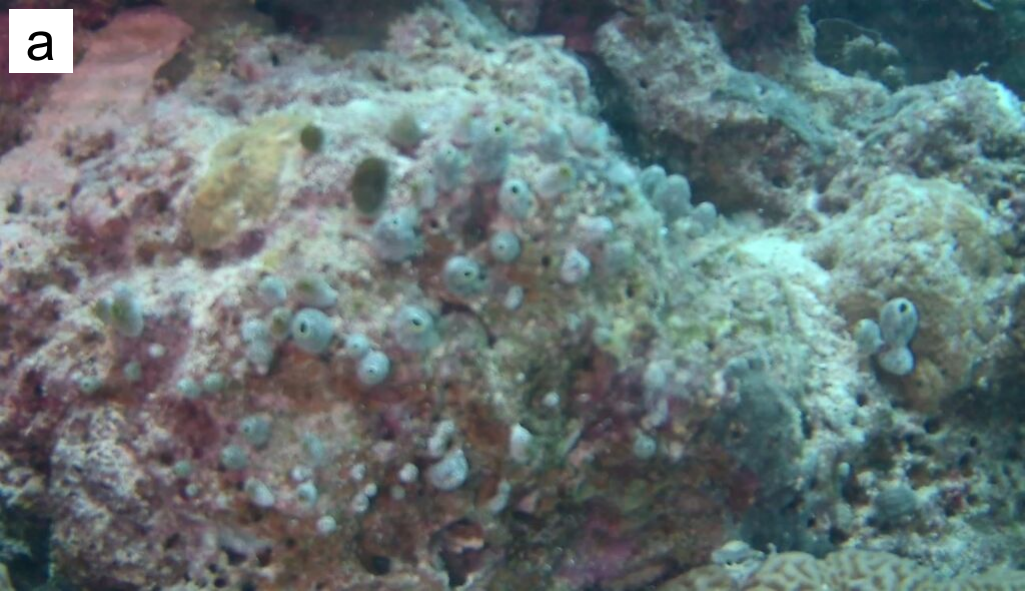
Vaavu, 10 m;

**Figure 281b.**
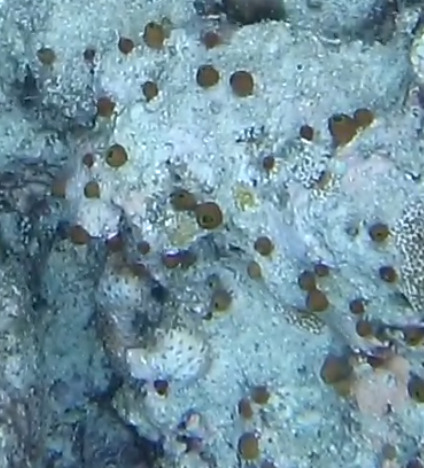
Vaavu, 2 m.

**Figure 282.**
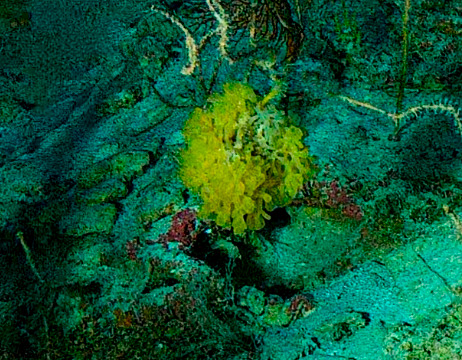
Ascidiacea ord. indet. sp. 1, Fuvahmulah, 120 m.

**Figure 283.**
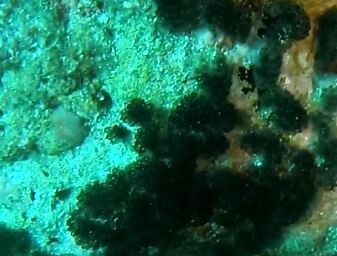
Ascidiacea ord. indet. sp. 2, Addu, 10 m.

**Figure 284a.**
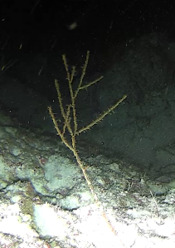
Huvadhu, 120 m;

**Figure 284b.**
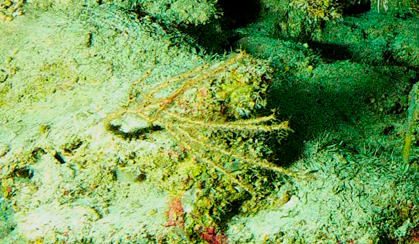
Vaavu, 120 m;

**Figure 284c.**
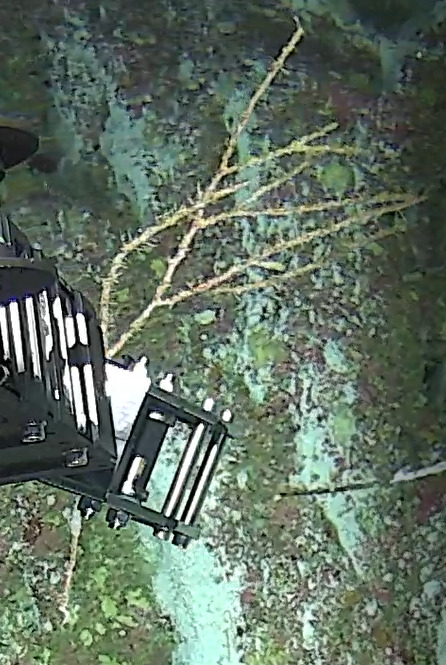
Addu, 120 m, *in situ* photo of collected specimen MAL1_261;

**Figure 284d.**
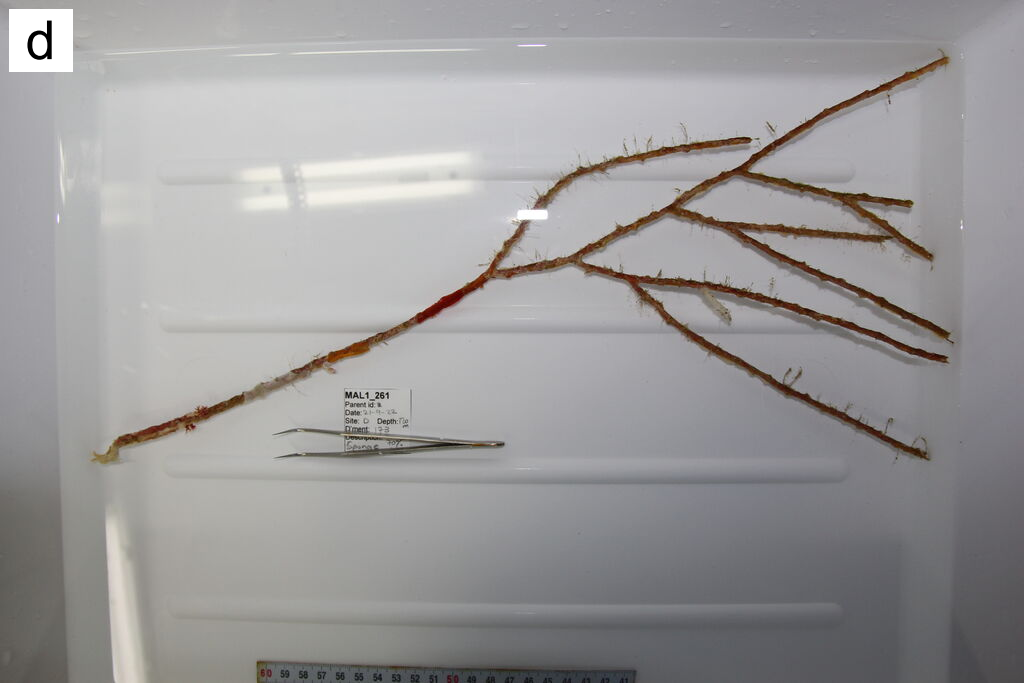
Addu, 120 m, *ex situ* photo of collected specimen MAL1_261.

**Table 1.** List of the 283 morphotypes observed in shallow and deeper reef habitats in the Seychelles during the Nekton Maldives Mission 2022 expedition. Open nomenclature (ON) signs applicable to image-based faunal analyses (e.g. indet., stet., inc.), as suggested by Horton et al. (2021), are also provided in the cases where species-level identification was not possible.

Phylum	Class	Order	Family	Genus	(Morpho)Species Scientific Name with ON signs
Cyanobacteria					Cyanobacteria stet. sp. 1
Cyanobacteria					Cyanobacteria stet. sp. 2
Chlorophyta	Ulvophyceae	Bryopsidales	Caulerpaceae	*Caulerpa*	*Caulerpa* sp. indet. 1
Chlorophyta	Ulvophyceae	Bryopsidales	Caulerpaceae	*Caulerpa*	*Caulerpaserrulata* sp. inc.
Chlorophyta	Ulvophyceae	Bryopsidales	Halimedaceae	*Halimeda*	*Halimeda* sp. indet.
Chlorophyta	Ulvophyceae	Bryopsidales	Halimedaceae	*Halimeda*	*Halimedamicronesica*
Chlorophyta	Ulvophyceae	Bryopsidales	Udoteaceae	*Tydemania*	*Tydemaniaexpeditionis*
Rhodophyta	Florideophyceae	Corallinales			Corallinales stet.
Porifera	Demospongiae	Clionaida	Clionaidae	*Spheciospongia*	*Spheciospongia* sp. indet. 4
Porifera	Demospongiae	Clionaida	Clionaidae	*Spheciospongia*	*Spheciospongia* sp. indet. 5
Porifera	Demospongiae	Clionaida	Clionaidae	*Spheciospongia*	*Spheciospongiaexcentrica*
Porifera	Demospongiae	Dictyoceratida	Thorectidae	*Phyllospongia*	*Phyllospongiafoliascens*
Porifera	Demospongiae	Haplosclerida	Callyspongiidae	*Callyspongia*	*Callyspongia* sp. indet. 1
Porifera	Demospongiae	Haplosclerida	Petrosiidae		Petrosiidae gen. indet. sp. 3
Porifera	Demospongiae	Petrosiidae	Petrosiidae	*Petrosia*	*Petrosia* sp. indet. 1
Porifera	Demospongiae	Petrosiidae	Petrosiidae	Petrosia (Strongylophora)	Petrosia (Strongylophora) sp. indet. 2
Porifera	Demospongiae	Haplosclerida	Chalinidae	*Haliclona*	*Haliclona* sp. indet. 15
Porifera	Demospongiae	Haplosclerida	Chalinidae	*Haliclona*	*Haliclona* sp. indet. 16
Porifera	Demospongiae	Haplosclerida	Chalinidae	*Haliclona*	*Haliclona* sp. indet. 17
Porifera	Demospongiae	Haplosclerida	Chalinidae	*Haliclona*	*Haliclona* sp. indet. 18
Porifera	Demospongiae	Haplosclerida	Chalinidae	*Haliclona*	*Haliclona* sp. indet. 19
Porifera	Demospongiae	Suberitida	Suberitidae	*Suberites*	*Suberites* sp. indet. 3
Porifera	Demospongiae	Suberitida	Suberitidae	*Rhizaxinella*	*Rhizaxinellaramulosa* sp. inc.
Porifera	Homoscleromorpha	Homosclerophorida	Plakinidae	*Plakortis*	*Plakortis* sp. indet. 3
Porifera	Demospongiae	Poecilosclerida	Iotrochotidae	*Iotrochota*	*Iotrochotanigra*
Porifera	Demospongiae	Poecilosclerida	Microcionidae	*Clathria*	*Clathria* sp. indet. 1
Porifera	Demospongiae	Scopalinida	Scopalinidae	*Stylissa*	*Stylissacarteri*
Porifera	Demospongiae	Tetractinellida	Ancorinidae	*Stelletta*	*Stelletta* sp. indet. 2
Porifera	Demospongiae	Tetractinellida	Corallistidae	*Corallistes*	*Corallistes* sp. indet. 2
Porifera	Demospongiae	Tetractinellida	Geodiidae	*Geodia*	*Geodia* sp. indet. 3
Porifera	Demospongiae	Tetractinellida	Geodiidae	*Geodia*	*Geodia* sp. indet. 4
Porifera	Demospongiae	Tetractinellida	Pachastrellidae	*Pachastrella*	*Pachastrella* sp. indet. 1
Porifera	Demospongiae				Demospongiae ord. indet. sp. 1
Porifera	Demospongiae				Demospongiae ord. indet. sp. 2
Porifera	Demospongiae				Demospongiae ord. indet. sp. 3
Porifera	Demospongiae				Demospongiae ord. indet. sp. 4
Porifera	Demospongiae				Demospongiae ord. sp. indet. 16
Porifera	Demospongiae				Demospongiae ord. sp. indet. 17
Porifera	Demospongiae				Demospongiae ord. sp. indet. 18
Porifera	Demospongiae				Demospongiae ord. sp. indet. 19
Porifera	Hexactinellida	Amphidiscosida	Hyalonematidae	*Hyalonema*	Hyalonema (Paradisconema) alcocki
Porifera	Hexactinellida	Amphidiscosida	Pheronematidae	*Semperella*	*Semperellacucumis*
Porifera	Hexactinellida	Sceptrulophora	Farreidae	*Farrea*	*Farrea* sp. indet. 1
Porifera	Hexactinellida	Sceptrulophora	Farreidae	*Farrea*	*Farrea* sp. indet. 2
Porifera	Hexactinellida	Sceptrulophora	Euretidae	*Pleurochorium*	*Pleurochoriumannandalei*
Porifera	Hexactinellida				Hexactinellida ord. indet. sp. 1
Cnidaria	Anthozoa-Hexacorallia	Actiniaria	Stichodactylidae	*Radianthus*	*Radianthusmagnifica*
Cnidaria	Anthozoa-Hexacorallia	Actiniaria	Heteractidae	*Heteractis*	*Heteractisaurora*
Cnidaria	Anthozoa-Hexacorallia	Actiniaria	Stichodactylidae	*Stichodactyla*	*Stichodactylamertensii*
Cnidaria	Anthozoa-Hexacorallia	Actiniaria			Actiniaria fam. indet. sp. 2
Cnidaria	Anthozoa-Hexacorallia	Actiniaria			Actiniaria fam. indet. sp. 4
Cnidaria	Anthozoa-Hexacorallia	Actiniaria			Actiniaria fam. indet. sp. 5
Cnidaria	Anthozoa-Hexacorallia	Actiniaria			Actiniaria fam. indet. sp. 6
Cnidaria	Anthozoa-Hexacorallia	Actiniaria			Actiniaria fam. indet. sp. 7
Cnidaria	Anthozoa-Hexacorallia	Actiniaria			Actiniaria fam. indet. sp. 8
Cnidaria	Anthozoa-Hexacorallia	Scleractinia	Acroporidae	*Acropora*	*Acropora* sp. indet. 1
Cnidaria	Anthozoa-Hexacorallia	Scleractinia	Acroporidae	*Acropora*	*Acropora* sp. indet. 2
Cnidaria	Anthozoa-Hexacorallia	Scleractinia	Acroporidae	*Acropora*	*Acropora* sp. indet. 3
Cnidaria	Anthozoa-Hexacorallia	Scleractinia	Acroporidae	*Acropora*	*Acropora* sp. indet. 4
Cnidaria	Anthozoa-Hexacorallia	Scleractinia	Acroporidae	*Acropora*	*Acropora* sp. indet. 5
Cnidaria	Anthozoa-Hexacorallia	Scleractinia	Acroporidae	*Acropora*	*Acropora* sp. indet. 6
Cnidaria	Anthozoa-Hexacorallia	Scleractinia	Acroporidae	*Acropora*	*Acropora* sp. indet. 7
Cnidaria	Anthozoa-Hexacorallia	Scleractinia	Acroporidae	*Astreopora*	*Astreopora* sp. indet.
Cnidaria	Anthozoa-Hexacorallia	Scleractinia	Acroporidae	*Montipora*	*Montipora* sp. indet. 1
Cnidaria	Anthozoa-Hexacorallia	Scleractinia	Acroporidae	*Montipora*	*Montipora* sp. indet. 2
Cnidaria	Anthozoa-Hexacorallia	Scleractinia	Agariciidae	*Gardineroseris*	*Gardineroserisplanulata*
Cnidaria	Anthozoa-Hexacorallia	Scleractinia	Agariciidae	*Leptoseris*	*Leptoseris* sp. indet.
Cnidaria	Anthozoa-Hexacorallia	Scleractinia	Agariciidae	*Pavona*	*Pavonavarians*
Cnidaria	Anthozoa-Hexacorallia	Scleractinia	Agariciidae	*Pavona*	*Pavona* sp. indet. 2
Cnidaria	Anthozoa-Hexacorallia	Scleractinia	Dendrophylliidae	*Dendrophyllia*	*Dendrophyllia* sp. indet.
Cnidaria	Anthozoa-Hexacorallia	Scleractinia	Dendrophylliidae	*Tubastraea*	*Tubastraea* sp. indet.
Cnidaria	Anthozoa-Hexacorallia	Scleractinia	Dendrophylliidae	*Turbinaria*	*Turbinaria* sp. indet.
Cnidaria	Anthozoa-Hexacorallia	Scleractinia	Dendrophylliidae	*Duncanopsammia*	*Duncanopsammiapeltata*
Cnidaria	Anthozoa-Hexacorallia	Scleractinia	Dendrophylliidae	*Balanophyllia*	*Balanophyllia* sp. indet.
Cnidaria	Anthozoa-Hexacorallia	Scleractinia	Diploastraeidae	*Diploastrea*	*Diploastreaheliopora*
Cnidaria	Anthozoa-Hexacorallia	Scleractinia	Euphyllidae	*Galaxea*	*Galaxea* sp. indet.
Cnidaria	Anthozoa-Hexacorallia	Scleractinia	Faviidae	*Dipsastraea*	*Dipsastraea* sp. indet.
Cnidaria	Anthozoa-Hexacorallia	Scleractinia	Fungiidae	*Heliofungiaactiniformis*	*Heliofungiaactiniformis*
Cnidaria	Anthozoa-Hexacorallia	Scleractinia	Fungiidae		Fungiidae sp. indet. 1
Cnidaria	Anthozoa-Hexacorallia	Scleractinia	Fungiidae	*Herpolitha*	*Herpolitha* sp. indet.
Cnidaria	Anthozoa-Hexacorallia	Scleractinia	Fungiidae	*Halomitra*	*Halomitra* sp. indet.
Cnidaria	Anthozoa-Hexacorallia	Scleractinia	Fungiidae	*Lithophyllon*	*Lithophyllonundulatum* sp. inc.
Cnidaria	Anthozoa-Hexacorallia	Scleractinia	Incerta saedis	*Pachyseris*	*Pachyserisrugosa*
Cnidaria	Anthozoa-Hexacorallia	Scleractinia	Incerta saedis	*Pachyseris*	*Pachyserisspeciosa*
Cnidaria	Anthozoa-Hexacorallia	Scleractinia	Incerta saedis	*Physogyra*	*Physogyralichtensteini*
Cnidaria	Anthozoa-Hexacorallia	Scleractinia	Incerta saedis	*Plerogyra*	*Plerogyrasinuosa*
Cnidaria	Anthozoa-Hexacorallia	Scleractinia	Incerta saedis	*Plesiastrea*	*Plesiastreaversipora*
Cnidaria	Anthozoa-Hexacorallia	Scleractinia	Lobophyllidae	*Echinophyllia*	*Echinophyllia* sp. indet.
Cnidaria	Anthozoa-Hexacorallia	Scleractinia	Lobophyllidae	*Lobophyllia*	*Lobophyllia* sp. indet.
Cnidaria	Anthozoa-Hexacorallia	Scleractinia	Lobophyllidae	*Oxypora*	*Oxyporacrassispinosa*
Cnidaria	Anthozoa-Hexacorallia	Scleractinia	Merulinidae	*Coelastrea*	*Coelastrea* sp. indet.
Cnidaria	Anthozoa-Hexacorallia	Scleractinia	Merulinidae	*Echinopora*	*Echinopora* sp. indet.
Cnidaria	Anthozoa-Hexacorallia	Scleractinia	Merulinidae	*Favites*	*Favites* sp. indet.
Cnidaria	Anthozoa-Hexacorallia	Scleractinia	Merulinidae	*Goniastrea*	*Goniastrea* sp. indet.
Cnidaria	Anthozoa-Hexacorallia	Scleractinia	Merulinidae	*Hydnophora*	*Hydnophora* sp. indet.
Cnidaria	Anthozoa-Hexacorallia	Scleractinia	Merulinidae	*Leptoria*	*Leptoria* sp. indet.
Cnidaria	Anthozoa-Hexacorallia	Scleractinia	Merulinidae	*Merulina*	*Merulina* sp. indet.
Cnidaria	Anthozoa-Hexacorallia	Scleractinia	Merulinidae	*Mycedium*	*Mycedium* sp. indet.
Cnidaria	Anthozoa-Hexacorallia	Scleractinia	Merulinidae	*Oulophyllia*	*Oulophyllia* sp. indet.
Cnidaria	Anthozoa-Hexacorallia	Scleractinia	Merulinidae	*Paragoniastrea*	*Paragoniastrearusselli* sp. inc.
Cnidaria	Anthozoa-Hexacorallia	Scleractinia	Merulinidae	*Platygyra*	*Platygyra* sp. indet.
Cnidaria	Anthozoa-Hexacorallia	Scleractinia	Pocilloporidae	*Madracis*	*Madracis* sp. indet.
Cnidaria	Anthozoa-Hexacorallia	Scleractinia	Pocilloporidae	*Pocillopora*	*Pocillopora* sp. indet. 1
Cnidaria	Anthozoa-Hexacorallia	Scleractinia	Pocilloporidae	*Pocillopora*	*Pocillopora* sp. indet. 2
Cnidaria	Anthozoa-Hexacorallia	Scleractinia	Pocilloporidae	*Pocillopora*	*Pocillopora* sp. indet. 3
Cnidaria	Anthozoa-Hexacorallia	Scleractinia	Pocilloporidae	*Pocillopora*	*Pocillopora* sp. indet. 4
Cnidaria	Anthozoa-Hexacorallia	Scleractinia	Poritidae	*Goniopora*	*Goniopora* sp. indet.
Cnidaria	Anthozoa-Hexacorallia	Scleractinia	Poritidae	*Porites*	*Poritesrus*
Cnidaria	Anthozoa-Hexacorallia	Scleractinia	Poritidae	*Porites*	*Porites* sp. indet. 1
Cnidaria	Anthozoa-Hexacorallia	Scleractinia	Poritidae	*Porites*	*Porites* sp. indet. 2
Cnidaria	Anthozoa-Hexacorallia	Scleractinia	Poritidae	*Porites*	*Porites* sp. indet. 3
Cnidaria	Anthozoa-Hexacorallia	Scleractinia	Psammocoridae	*Psammocora*	*Psammocora* sp. indet.
Cnidaria	Anthozoa-Hexacorallia	Zoantharia	Sphenopidae	*Palythoa*	*Palythoatuberculosa*
Cnidaria	Anthozoa-Hexacorallia	Zoantharia	Sphenopidae	*Palythoa*	*Palythoa* sp. indet. 2
Cnidaria	Anthozoa-Hexacorallia	Antipatharia	Antipathidae	*Arachnopathes*	*Arachnopathes* sp. indet.
Cnidaria	Anthozoa-Hexacorallia	Antipatharia	Antipathidae	*Antipathes*	*Antipathesnilanduensis* sp. inc.
Cnidaria	Anthozoa-Hexacorallia	Antipatharia	Antipathidae	*Antipathes*	*Antipathes* sp. indet. 2
Cnidaria	Anthozoa-Hexacorallia	Antipatharia	Antipathidae	*Antipathes*	*Antipathes* sp. indet. 3
Cnidaria	Anthozoa-Hexacorallia	Antipatharia	Antipathidae	*Antipathes*	*Antipathes* sp. indet. 4
Cnidaria	Anthozoa-Hexacorallia	Antipatharia	Aphanipathidae	*Asteriopathes*	*Asteriopathes* sp. indet.
Cnidaria	Anthozoa-Hexacorallia	Antipatharia	Aphanipathidae	*Tetrapathes*	*Tetrapathes* sp. indet.
Cnidaria	Anthozoa-Hexacorallia	Antipatharia	Schizopathidae	*Bathypathes*	*Bathypathes* sp. indet.
Cnidaria	Anthozoa-Hexacorallia	Antipatharia	Myriopathidae	*Cupressopathes*	*Cupressopathes* sp. indet.
Cnidaria	Anthozoa-Hexacorallia	Antipatharia	Myriopathidae	*Myriopathes*	*Myriopathes* sp. indet. 1
Cnidaria	Anthozoa-Hexacorallia	Antipatharia	Aphanipathidae	*Pteridopathes*	*Pteridopathes* sp. indet.
Cnidaria	Anthozoa-Hexacorallia	Antipatharia	Stylopathidae	*Stylopathes*	*Stylopathes* sp. indet.
Cnidaria	Anthozoa-Hexacorallia	Antipatharia	Schizopathidae	*Parantipathes*	*Parantipathes* sp. indet.
Cnidaria	Anthozoa-Hexacorallia	Antipatharia	Schizopathidae	*Umbellapathes*	*Umbellapathes* sp. indet.
Cnidaria	Anthozoa-Hexacorallia	Antipatharia			Antipatharia fam. indet. sp. 7
Cnidaria	Anthozoa-Hexacorallia	Corallimorpharia		*Rhodactis*	*Rhodactis* sp. indet.
Cnidaria	Anthozoa-Octocorallia	Malacalcyonacea	Astrogorgiidae	*Astrogorgia*	*Astrogorgia* sp. indet.
Cnidaria	Anthozoa-Octocorallia	Malacalcyonacea	Melithaeidae	*Melithaea*	*Melithaea* sp. indet. 1
Cnidaria	Anthozoa-Octocorallia	Malacalcyonacea	Melithaeidae	*Melithaea*	*Melithaea* sp. indet. 2
Cnidaria	Anthozoa-Octocorallia	Malacalcyonacea	Nephtheidae	*Dendronephthya*	*Dendronephthya* sp. indet. 1
Cnidaria	Anthozoa-Octocorallia	Malacalcyonacea	Nephtheidae	*Dendronephthya*	*Dendronephthya* sp. indet. 4
Cnidaria	Anthozoa-Octocorallia	Malacalcyonacea	Nephtheidae		Nephtheidae gen. indet. sp. 5
Cnidaria	Anthozoa-Octocorallia	Malacalcyonacea	Nephtheidae	*Scleronephythya*	*Scleronephythya* sp. indet. 2
Cnidaria	Anthozoa-Octocorallia	Malacalcyonacea	Nephtheidae	*Umbellulifera*	*Umbellulifera* sp. indet.
Cnidaria	Anthozoa-Octocorallia	Malacalcyonacea	Paramuriceidae		Paramuriceidae gen. indet. sp. 1
Cnidaria	Anthozoa-Octocorallia	Malacalcyonacea	Paramuriceidae	*Acanthogorgia*	*Acanthogorgia* sp. indet.
Cnidaria	Anthozoa-Octocorallia	Malacalcyonacea	Plexauridae		Plexauridae gen. indet. sp. 2
Cnidaria	Anthozoa-Octocorallia	Malacalcyonacea	Plexauridae		Plexauridae gen. indet. sp. 9
Cnidaria	Anthozoa-Octocorallia	Malacalcyonacea	Sarcophytidae	*Lobophytum*	*Lobophytum* sp. indet.
Cnidaria	Anthozoa-Octocorallia	Malacalcyonacea	Sarcophytidae	*Sarcophyton*	*Sarcophyton* sp. indet.
Cnidaria	Anthozoa-Octocorallia	Malacalcyonacea	Sinulariidae		Sinulariidae gen. indet. sp. 1
Cnidaria	Anthozoa-Octocorallia	Malacalcyonacea	Sinulariidae		Sinulariidae gen. indet. sp. 2
Cnidaria	Anthozoa-Octocorallia	Malacalcyonacea	Sinulariidae		Sinulariidae gen. indet. sp. 3
Cnidaria	Anthozoa-Octocorallia	Malacalcyonacea	Sinulariidae		Sinulariidae gen. indet. sp. 4
Cnidaria	Anthozoa-Octocorallia	Malacalcyonacea	Siphonogorgiidae	*Chironephthya*	*Chironephthya* sp. indet. 2
Cnidaria	Anthozoa-Octocorallia	Malacalcyonacea	Subergorgiidae		Subergorgiidae sp. indet. 1
Cnidaria	Anthozoa-Octocorallia	Malacalcyonacea	Subergorgiidae	*Annella*	*Annella* sp. indet.
Cnidaria	Anthozoa-Octocorallia	Malacalcyonacea	Subergorgiidae	*Subergorgia*	*Subergorgia* sp. indet. 2
Cnidaria	Anthozoa-Octocorallia	Malacalcyonacea			Malacalcyonacea fam. indet.
Cnidaria	Anthozoa-Octocorallia	Scleralcyonacea	Ellisellidae	*Ellisella*	*Ellisella* sp. indet.
Cnidaria	Anthozoa-Octocorallia	Scleralcyonacea	Ellisellidae	*Nicella*	*Nicella* sp. indet.
Cnidaria	Anthozoa-Octocorallia	Scleralcyonacea	Ellisellidae		Ellisellidae gen. indet. sp. 2
Cnidaria	Anthozoa-Octocorallia	Scleralcyonacea	Ellisellidae		Ellisellidae gen. indet. sp. 4
Cnidaria	Anthozoa-Octocorallia	Scleralcyonacea	Ellisellidae		Ellisellidae gen. indet. sp. 5
Cnidaria	Anthozoa-Octocorallia	Scleralcyonacea	Ellisellidae		Ellisellidae gen. indet. sp. 6
Cnidaria	Anthozoa-Octocorallia	Scleralcyonacea	Ellisellidae		Ellisellidae gen. indet. sp. 8
Cnidaria	Anthozoa-Octocorallia	Scleralcyonacea	Ellisellidae		Ellisellidae gen. indet. sp. 9
Cnidaria	Anthozoa-Octocorallia	Scleralcyonacea	Helioporidae	*Heliopora*	*Heliopora* sp. indet.
Cnidaria	Anthozoa-Octocorallia	Scleralcyonacea	Pennatuloidea		Pennatuloidea gen. indet. sp.
Cnidaria	Anthozoa-Octocorallia	Scleralcyonacea	Primnoidae		Primnoidae gen. indet. sp. 1
Cnidaria	Anthozoa-Octocorallia	Scleralcyonacea	Primnoidae		Primnoidae gen. indet. sp. 2
Cnidaria	Anthozoa-Octocorallia	Scleralcyonacea	Primnoidae		Primnoidae gen. indet. sp. 3
Cnidaria	Anthozoa-Octocorallia	Scleralcyonacea	Primnoidae		Primnoidae gen. indet. sp. 4
Cnidaria	Anthozoa-Octocorallia	Scleralcyonacea	Primnoidae		Primnoidae gen. indet. sp. 5
Cnidaria	Anthozoa-Octocorallia				Octocorallia ord. indet. sp. 3
Cnidaria	Anthozoa-Octocorallia				Octocorallia ord. indet. sp. 4
Cnidaria	Anthozoa-Octocorallia				Octocorallia ord. indet. sp. 5
Cnidaria	Anthozoa-Octocorallia				Octocorallia ord. indet. sp. 6
Cnidaria	Anthozoa-Octocorallia				Octocorallia ord. indet. sp. 7
Cnidaria	Anthozoa-Octocorallia				Octocorallia ord. indet. sp. 8
Cnidaria	Anthozoa-Octocorallia				Octocorallia ord. indet. sp. 9
Cnidaria	Anthozoa-Octocorallia				Octocorallia ord. indet. sp. 10
Cnidaria	Anthozoa-Octocorallia				Octocorallia ord. indet. sp. 11
Cnidaria	Anthozoa-Octocorallia				Octocorallia ord. indet. sp. 12
Cnidaria	Anthozoa-Octocorallia				Octocorallia ord. indet. sp. 13
Cnidaria	Anthozoa-Octocorallia				Octocorallia ord. indet. sp. 14
Cnidaria	Anthozoa-Octocorallia				Octocorallia ord. indet. sp. 15
Cnidaria	Anthozoa-Octocorallia				Octocorallia ord. indet. sp. 18
Cnidaria	Anthozoa-Octocorallia				Octocorallia ord. indet. sp. 19
Cnidaria	Anthozoa-Octocorallia				Octocorallia ord. indet. sp. 20
Cnidaria	Anthozoa-Octocorallia				Octocorallia ord. indet. sp. 21
Cnidaria	Anthozoa-Octocorallia				Octocorallia ord. indet. sp. 22
Cnidaria	Anthozoa-Octocorallia				Octocorallia ord. indet. sp. 24
Cnidaria	Anthozoa-Octocorallia				Octocorallia ord. indet. sp. 25
Cnidaria	Anthozoa-Octocorallia				Octocorallia ord. indet. sp. 27
Cnidaria	Anthozoa-Octocorallia				Octocorallia ord. indet. sp. 29
Cnidaria	Anthozoa-Octocorallia				Octocorallia ord. indet. sp. 31
Cnidaria	Anthozoa-Ceriantharia				Ceriantharia stet.
Cnidaria	Hydrozoa	Anthoathecata	Milleporidae	*Millepora*	*Millepora* sp. indet. 1
Cnidaria	Hydrozoa	Anthoathecata	Milleporidae	*Millepora*	*Millepora* sp. indet. 2
Cnidaria	Hydrozoa	Anthoathecata	Milleporidae	*Millepora*	*Millepora* sp. indet. 3
Cnidaria	Hydrozoa	Anthoathecata	Stylasteridae	*Crypthelia*	*Crypthelia* gen. indet. sp.
Cnidaria	Hydrozoa	Anthoathecata	Stylasteridae		Stylasteridae gen. indet. sp. 4
Cnidaria	Hydrozoa	Anthoathecata	Stylasteridae		Stylasteridae gen. indet. sp. 5
Cnidaria	Hydrozoa	Anthoathecata	Stylasteridae		Stylasteridae gen. indet. sp. 6
Cnidaria	Hydrozoa	Anthoathecata	Stylasteridae		Stylasteridae gen. indet. sp. 7
Cnidaria	Hydrozoa	Anthoathecata	Stylasteridae		Stylasteridae gen. indet. sp. 8
Cnidaria	Hydrozoa				Hydrozoa ord. indet. sp. 1
Cnidaria	Hydrozoa				Hydrozoa ord. indet. sp. 4
Cnidaria	Hydrozoa				Hydrozoa ord. indet. sp. 5
Ctenophora	Tentaculata	Platyctenida	Lyroctenidae	*Lyrocteis*	*Lyrocteis* sp. indet.
Mollusca	Bivalvia	Cardiida	Cardiidae	*Tridacna*	*Tridacna* sp. indet.
Mollusca	Bivalvia				Bivalvia ord. indet. sp. 1
Mollusca	Bivalvia				Bivalvia ord. indet. sp. 2
Mollusca	Bivalvia				Bivalvia ord. indet. sp. 3
Mollusca	Cephalopoda	Octopoda			Octopoda fam. indet. sp.
Mollusca	Gastropoda	Littorinimorpha	Strombidae		Strombidae gen. indet. sp. 1
Mollusca	Gastropoda	Littorinimorpha	Strombidae		Strombidae gen. indet. sp. 2
Mollusca	Gastropoda	Neogastropoda	Conoidea		Conoidea gen. indet. sp.
Mollusca	Gastropoda	Neogastropoda	Muricidae	*Drupella*	*Drupella* sp. indet.
Annelida	Polychaeta				Polychaeta ord. indet. sp. 1
Annelida	Polychaeta				Polychaeta ord. indet. sp. 2
Annelida	Polychaeta				Polychaeta ord. indet. sp. 3
Annelida	Polychaeta				Polychaeta ord. indet. sp. 4
Annelida	Polychaeta				Polychaeta ord. indet. sp. 5
Annelida	Polychaeta-Echiura				Echiura ord. indet. sp.
Arthropoda	Malacostraca	Decapoda	Galatheoidea		Galatheoidea gen. indet. sp.
Arthropoda	Malacostraca	Decapoda	Chirostyloidea		Chirostyloidea gen. indet. sp. 1
Arthropoda	Malacostraca	Decapoda	Chirostyloidea		Chirostyloidea gen. indet. sp. 2
Arthropoda	Malacostraca	Decapoda	Leucosiidae		Leucosiidae gen. indet. sp.
Arthropoda	Malacostraca	Decapoda	Munidopsidae		Munidopsidae gen. indet. sp.
Arthropoda	Malacostraca	Decapoda	Mithracidae		Mithracidae gen. indet. sp.
Arthropoda	Malacostraca	Decapoda	Homolidae		Homolidae gen. indet. sp.
Arthropoda	Malacostraca	Decapoda	Paguroidea	*Paguropsis*	*Paguropsisconfusa*
Arthropoda	Malacostraca	Decapoda	Aristeidae		Aristeidae gen. indet. sp.
Arthropoda	Malacostraca	Decapoda-Brachyura	Xanthidae		Xanthidae gen. indet. sp.
Arthropoda	Malacostraca	Decapoda-Brachyura	Calappidae		Calappidae gen. indet. sp.
Arthropoda	Malacostraca	Decapoda-Brachyura	Geryonidae		Geryonidae gen. indet. sp.
Arthropoda	Malacostraca	Decapoda-Caridea			Caridea fam. indet. sp.
Bryozoa					Bryozoa clas. indet. sp. 1
Bryozoa	Gymnolaemata	Cheilostomatida	Cellariidae	*Cellaria*	*Cellaria* sp. indet.
Echinodermata	Asteroidea	Brisingida	Brisingidae		Brisingidae gen. indet. sp.
Echinodermata	Asteroidea	Forcipulatida	Asteriidae	*Sclerasterias*	*Sclerasterias* sp. indet.
Echinodermata	Asteroidea	Paxillosida	Astropectinidae	*Persephonaster*	*Persephonaster* sp. indet.
Echinodermata	Asteroidea	Paxillosida	Astropectinidae		Astropectinidae gen. indet. sp. 1
Echinodermata	Asteroidea	Paxillosida	Astropectinidae		Astropectinidae gen. indet. sp. 2
Echinodermata	Asteroidea	Spinulosida	Echinasteridae	*Echinaster*	*Echinasterluzonicus*
Echinodermata	Asteroidea	Valvatida	Asterinidae	*Anseropoda*	*Anseropoda* sp. indet.
Echinodermata	Asteroidea	Valvatida	Asterinidae	*Paranepanthia*	*Paranepanthia* sp. indet.
Echinodermata	Asteroidea	Valvatida	Asterinidae	*Tremaster*	*Tremastermirabilis*
Echinodermata	Asteroidea	Valvatida	Asterinidae		Asterinidae gen. indet. sp.
Echinodermata	Asteroidea	Valvatida	Goniasteridae	*Mediaster*	*Mediaster* sp. indet.
Echinodermata	Asteroidea	Valvatida	Goniasteridae	*Ceramaster*	*Ceramaster* sp. indet.
Echinodermata	Asteroidea	Valvatida	Goniasteridae	*Sphaeriodiscus*	*Sphaeriodiscus* sp. indet.
Echinodermata	Asteroidea	Valvatida	Goniasteridae	*Nymphaster*	*Nymphaster* sp. indet.
Echinodermata	Asteroidea	Valvatida	Goniasteridae		Goniasteridae gen. indet. sp. 4
Echinodermata	Asteroidea	Valvatida	Goniasteridae	*Fromia*	*Fromiamonilis*
Echinodermata	Asteroidea	Valvatida	Oreasteridae	*Astrosarkus*	*Astrosarkusidipi*
Echinodermata	Asteroidea	Valvatida	Oreasteridae	*Choriaster*	*Choriastergranulatus*
Echinodermata	Asteroidea	Valvatida	Oreasteridae	*Culcita*	*Culcitaschmideliana*
Echinodermata	Asteroidea	Valvatida	Ophidiasteridae	*Linckia*	*Linckia* sp. indet.
Echinodermata	Asteroidea				Asteroidea ord. indet. sp. 5
Echinodermata	Asteroidea				Asteroidea ord. indet. sp. 6
Echinodermata	Ophiuroidea				Ophiuroidea stet.
Echinodermata	Crinoidea				Crinoidea ord. indet. sp. 1
Echinodermata	Crinoidea				Crinoidea ord. indet. sp. 2
Echinodermata	Crinoidea				Crinoidea ord. indet. sp. 3
Echinodermata	Echinoidea	Micropygoida	Micropygidae	*Micropyga*	*Micropyga* sp. indet.
Echinodermata	Echinoidea	Cidaroida			Cidaroida fam. indet. sp. 1
Echinodermata	Echinoidea	Cidaroida			Cidaroida fam. indet. sp. 2
Echinodermata	Echinoidea	Clypeasteroida	Clypeasteridae	*Clypeaster*	*Clypeaster* sp. indet.
Echinodermata	Echinoidea	Diadematoida	Diadematidae	*Echinothrix*	*Echinothrixdiadema*
Echinodermata	Echinoidea				Echinoidea ord. indet. sp. 1
Echinodermata	Echinoidea				Echinoidea ord. indet. sp. 2
Echinodermata	Echinoidea	Spatangoida			Spatangoida fam. indet. sp.
Echinodermata	Holothuroidea	Holothuriida	Holothuriidae	*Holothuria*	*Holothuriaatra*
Echinodermata	Holothuroidea	Holothuriida	Holothuriidae	*Holothuria*	*Holothuriaedulis*
Echinodermata	Holothuroidea	Holothuriida	Holothuriidae	*Pearsonothuria*	*Pearsonothuriagraeffei*
Echinodermata	Holothuroidea				Holothuroidea ord. indet. sp. 3
Echinodermata	Holothuroidea				Holothuroidea ord. indet. sp. 4
Echinodermata	Holothuroidea				Holothuroidea ord. indet. sp. 5
Echinodermata	Holothuroidea				Holothuroidea ord. indet. sp. 6
Echinodermata	Holothuroidea				Holothuroidea ord. indet. sp. 7
Echinodermata	Holothuroidea				Holothuroidea ord. indet. sp. 8
Echinodermata	Holothuroidea				Holothuroidea ord. indet. sp. 9
Chordata-Tunicata	Ascidiacea	Aplousobranchia	Didemnidae	*Didemnum*	*Didemnummolle*
Chordata-Tunicata	Ascidiacea				Ascidiacea ord. indet. sp. 1
Chordata-Tunicata	Ascidiacea				Ascidiacea ord. indet. sp. 2
Unknown					Unknown sp. indet. 1
